# InSituPREP enables 3D single-cell mapping of interaction-associated gene programs in the breast cancer tumor microenvironment

**DOI:** 10.1093/nar/gkag406

**Published:** 2026-05-11

**Authors:** Tal Goldberg, Michal Danino-Levi, Modi Safra, Yedaaya Copeland, Efrat R Weizman, Noa Konforti, Tal Ishon, Noga Ben-Ari, Yael Brand, Bareket Kruger, Gili Perry, Dana Morzaev-Sulzbach, Einav N Gal-Yam, Maya Dadiani, Shahar Alon

**Affiliations:** The Alexander Kofkin Faculty of Engineering, Bar-Ilan University, Ramat Gan, 5290002, Israel; Institute for Nanotechnology and Advanced Materials (BINA), Bar-Ilan University, Ramat Gan, 5290002, Israel; The Gonda Multidisciplinary Brain Research Center, Bar-Ilan University, Ramat Gan, 5290002, Israel; The Alexander Kofkin Faculty of Engineering, Bar-Ilan University, Ramat Gan, 5290002, Israel; Institute for Nanotechnology and Advanced Materials (BINA), Bar-Ilan University, Ramat Gan, 5290002, Israel; The Gonda Multidisciplinary Brain Research Center, Bar-Ilan University, Ramat Gan, 5290002, Israel; The Alexander Kofkin Faculty of Engineering, Bar-Ilan University, Ramat Gan, 5290002, Israel; Institute for Nanotechnology and Advanced Materials (BINA), Bar-Ilan University, Ramat Gan, 5290002, Israel; The Gonda Multidisciplinary Brain Research Center, Bar-Ilan University, Ramat Gan, 5290002, Israel; The Alexander Kofkin Faculty of Engineering, Bar-Ilan University, Ramat Gan, 5290002, Israel; The Alexander Kofkin Faculty of Engineering, Bar-Ilan University, Ramat Gan, 5290002, Israel; Institute for Nanotechnology and Advanced Materials (BINA), Bar-Ilan University, Ramat Gan, 5290002, Israel; The Gonda Multidisciplinary Brain Research Center, Bar-Ilan University, Ramat Gan, 5290002, Israel; The Alexander Kofkin Faculty of Engineering, Bar-Ilan University, Ramat Gan, 5290002, Israel; Institute for Nanotechnology and Advanced Materials (BINA), Bar-Ilan University, Ramat Gan, 5290002, Israel; The Gonda Multidisciplinary Brain Research Center, Bar-Ilan University, Ramat Gan, 5290002, Israel; The Alexander Kofkin Faculty of Engineering, Bar-Ilan University, Ramat Gan, 5290002, Israel; The Alexander Kofkin Faculty of Engineering, Bar-Ilan University, Ramat Gan, 5290002, Israel; The Alexander Kofkin Faculty of Engineering, Bar-Ilan University, Ramat Gan, 5290002, Israel; Institute for Nanotechnology and Advanced Materials (BINA), Bar-Ilan University, Ramat Gan, 5290002, Israel; The Gonda Multidisciplinary Brain Research Center, Bar-Ilan University, Ramat Gan, 5290002, Israel; The Alexander Kofkin Faculty of Engineering, Bar-Ilan University, Ramat Gan, 5290002, Israel; Cancer Research Center, Sheba Medical Center, Ramat Gan, 52621, Israel; Cancer Research Center, Sheba Medical Center, Ramat Gan, 52621, Israel; Institute of Breast Oncology, Jusidman Cancer Center, Sheba Medical Center, Ramat Gan, 52621, Israel; Cancer Research Center, Sheba Medical Center, Ramat Gan, 52621, Israel; The Alexander Kofkin Faculty of Engineering, Bar-Ilan University, Ramat Gan, 5290002, Israel; Institute for Nanotechnology and Advanced Materials (BINA), Bar-Ilan University, Ramat Gan, 5290002, Israel; The Gonda Multidisciplinary Brain Research Center, Bar-Ilan University, Ramat Gan, 5290002, Israel

## Abstract

Cell-cell interactions influence gene expression in the tumor microenvironment, yet despite advances in spatial transcriptomics that enable *in situ* expression profiling, current computational tools remain limited in quantitatively resolving single-cell and multicellular interaction effects across patient tissues. Here, we present InSituPREP (In Situ Proximity Expression Programs), a computational framework for quantifying how spatial context relates to transcriptional states in three-dimensional tissues at single-cell resolution. The framework quantifies proximity-associated gene programs across multiple analytical layers. We applied InSituPREP to Expansion Sequencing data from breast cancer biopsies of 10 patients, integrating newly generated and previously published datasets and profiling 299 cancer-related genes. This analysis revealed that a portion of within-cell type variability reflects proximity-associated transcriptional states, that proximity-dependent programs are reproducible across patients and measurement platforms, and that spatial RNA velocity links intercellular distance to future transcriptional trajectories. Linear modeling showed that gene expression in specific cell types varies with intercellular distance and the number of neighboring cells, while triplet configurations revealed additional interaction-associated programs. Moreover, simultaneous detection of bacterial signals and host mRNA enabled us to link bacterial proximity to distinct transcriptional responses. InSituPREP provides a framework for quantifying spatially associated gene expression and dissecting context-dependent transcriptional states at single-cell resolution.

## Graphical Abstract

**Graphical Abstract:**
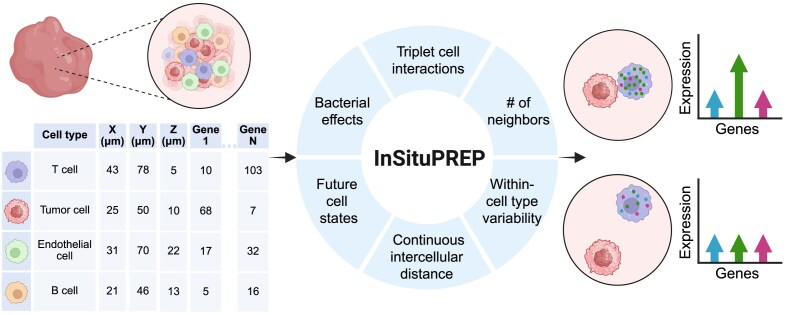

## Introduction

The tumor microenvironment (TME) is a complex ecosystem in which cancer cells interact with immune, stromal, and vascular lineages. These interactions shape tumor growth, immune evasion, and therapeutic response [1, 2]. Although tumor-infiltrating lymphocytes are generally associated with a favorable prognosis in breast cancer, their effects are heterogeneous [3], likely reflecting differences in local cell-cell interaction features. Prior studies have shown structured spatial organization within the TME, with immune and stromal populations exhibiting characteristic localization patterns [4], yet how such arrangements translate into proximity-associated transcriptional changes remains unclear [5]. Understanding how these interactions influence transcriptional states requires measuring gene expression within intact spatial contexts, where proximity-driven signals such as ligand-receptor, autocrine, and paracrine interactions occur, and developing computational methods capable of linking these expression programs to spatial proximity.

While single-cell transcriptomics has provided important insights into cell-intrinsic states, dissociation removes the spatial information needed to study how local neighborhoods influence gene expression [6, 7]. Emerging spatial transcriptomics technologies now enable gene-expression measurements within preserved tissue architecture [8]. Specifically, spatial approaches with single-cell resolution are particularly critical for detecting subtle, short-range transcriptional effects that lower-resolution methods often miss [9]. In situ fluorescence-based multiplexed platforms achieve single-cell or subcellular resolution, including MERFISH [10], SeqFISH, STARmap, ISS, and related techniques [11–15], as well as Expansion Sequencing (ExSeq) [16], a super-resolution *in situ* sequencing approach developed by us and others. These methods open the door to quantifying cell-cell interactions at the level of individual interacting cells within biopsies.

Extracting interpretable biological signals from these data remains challenging. Computational frameworks such as CellPhoneDB [17], CellChat [18], and NicheNet [19] have advanced the inference of ligand–receptor signaling from single-cell transcriptomics, but they lack spatial context. Spatially informed approaches, including Giotto [20], Squidpy [21], stLearn [22], Spacia [9], and COMMOT [23], incorporate positional information, yet most encode proximity in a binary or discrete manner and are largely restricted to pairwise interactions in two-dimensional sections. As a result, they cannot fully resolve gradual, distance-dependent transcriptional changes or higher-order, multicellular dependencies in three-dimensional tissue. MuSpAn [24] recently extended spatial analyses across multiple length scales, yet like other existing approaches, it does not infer the transcriptional programs underlying cell-cell interactions, leaving the molecular consequences of spatial proximity insufficiently characterized. Other previous attempts to relate spatial proximity to transcriptional changes have relied on coarse binning strategies. For example, tumor cells were aggregated into 100 × 100 µm bins and differential expression was performed between bins with and without immune cells (T cells or NK cells) [25]. While informative, such binning obscures cell-level interaction signals. Therefore, computational methods are needed to resolve interaction-associated gene programs at single-cell resolution while preserving full 3D tissue geometry and capturing complex spatial effects.

Here, we applied ExSeq to a cohort of 10 breast cancer biopsies, integrating newly generated and previously published data and profiling 299 cancer-related genes. To systematically interpret these data, we developed InSituPREP (*In Situ Proximity Expression Program*), a computational framework for quantifying how spatial context contributes to transcriptional variability. InSituPREP integrates multiple analytical layers spanning distinct spatial scales, from binary tumor-immune contacts and graded distance-dependent expression changes to local cellular density effects and higher-order (triplet) neighborhoods of cell types. This continuous, three-dimensional, and multicellular representation overcomes limitations of existing approaches that rely on binary, planar, or strictly pairwise interactions. The framework further incorporates spatial RNA velocity [26, 27] to evaluate how proximity relates to predicted future states. In addition, we expanded the ExSeq platform to detect bacterial 16S rRNA *in situ* and used InSituPREP to analyze host-microbiome interactions, correlating microbial presence with host transcriptional responses. Together, these components establish a single-cell framework for dissecting how local microenvironmental context is associated with cell states in human tumors.

## Materials and methods

### Generation of the dataset

We analyzed 10 breast cancer biopsies that were *in situ* sequenced using ExSeq.

Overall, eight samples were derived from metastatic breast cancer collected from different metastatic sites, mostly from the liver, seven of which were previously published and generated by us and others [25]: 313 (liver, HR⁻/HER2⁺), 330 (liver, HR⁺/HER2⁻), 364 (liver, HR⁺/HER2⁺), 514 (axilla, HR⁻/HER2⁻), 783 (breast, collected from the primary site following metastatic diagnosis, HR⁺/HER2⁻), 880 (liver, HR⁺/HER2⁻), and 982 (liver, HR⁺/HER2⁻). In addition, we analyzed sample 100 (liver, HR⁺; biospecimen identifier as in the original publication [28]), a metastatic breast cancer specimen previously published and generated by us and others [28]. Ethical approvals and informed consent procedures for these previously published samples are described in the respective publications [25, 28].

We performed ExSeq on two pretreatment, preoperative breast biopsies collected from patients with primary, non-metastatic breast cancer at Sheba Medical Center. Prior to any study procedures, both patients provided written informed consent for research biopsy and subsequent molecular analysis. The study was approved by Sheba Medical Center’s Helsinki Committee (approval 7509–09-SMC; Einav Nili Gal-Yam) and was conducted in accordance with the ethical standards of the Helsinki Declaration. The patients were female, both aged 50, and both diagnosed as HR⁺ breast cancer, with both estrogen receptor (ER)-positive and progesterone receptor (PR)-positive status, and HER2^−^ status. One patient (sample 58; biospecimen identifier from Sheba Medical Center) exhibited a partial response to therapy, whereas the other (sample 59) showed no response. Fresh tumor tissue was collected via core needle biopsy, snap-frozen in liquid nitrogen, molded with OCT compound, and preserved at −80°C until processing. Sections (10 µm) were prepared using a Leica Cryotome, mounted on Superfrost Plus slides, and immediately fixed in ice-cold 10% formalin in 1 × PBS for 12 min. Slides were then washed three times for 5 min each with ice-cold 1 × PBS and stored at 4°C in 70% ethanol until use. Both biopsies contained detectable bacterial content, as demonstrated by RNA sequencing of 16S rRNA using the method described in [29].

Processing followed established protocols [16]: the samples were gelled, digested, and physically expanded. Targeted *in situ* RNA sequencing was performed for bacterial 16S rRNA alongside 299 human breast cancer-related genes, spanning markers of epithelial, stromal, and immune cells. The full list of these 299 genes is described in [25, 28]. Bacteria were detected using specifically designed probes against 16S rRNA sequences conserved across multiple bacterial strains. Each probe consisted of three components: (i) a homology region specific to 16S rRNA without matches in the human genome (verified with BLAT, UCSC Genome Browser); (ii) a constant backbone region for *in situ* sequencing primer binding; and (iii) a constant seven-base barcode sequenced *in situ* to identify 16S rRNA. All barcodes, including those for mRNA and bacterial probes, were designed with a Hamming distance of three, allowing correction of a single substitution error during *in situ* sequencing. The resulting padlock probes were hybridized to RNA, circularized, and amplified by rolling-circle amplification. Iterative rounds of sequencing chemistry were then performed, during which cDNA amplicons were sequenced *in situ* using fluorescently labeled oligonucleotides (Supplementary Fig. S1). To facilitate repeated imaging, samples were attached to the bottom of a 24-well glass-bottom plate, ensuring stable positioning across sequencing cycles. For attachment, wells were pretreated with Bind-Silane, and samples were re-embedded in individual wells using a re-embedding gel, as described in [16].

### Segmentation of cells in ExSeq data

Cell segmentation was performed on each tissue to ensure accurate delineation of single-cell boundaries. For tissues 313, 330, 364, 880, 982, 783, 59, and 100, segmentation was carried out using InSituSeg [28], a tool specifically designed for multiplexed *in situ* transcriptomics data and optimized for 3D tissue architecture (Supplementary Fig. S1). For tissues 514 and 58, we used a refinement of InSituSeg, which we term OctopusSeg, based on similar principles as InSituSeg and yields comparable results. Outputs from both segmentation methods were consolidated into a unified cell-by-gene count matrix for downstream analysis.

### Cell type clustering of ExSeq data

As a first step toward cell-type clustering, tissue-specific marker genes were extracted from matched single-cell RNA-seq (scRNA-seq) data. For seven of the 10 tissues analyzed (313, 330, 364, 514, 783, 880, and 982), scRNA-seq data from adjacent sections were available [25]. For the remaining three tissues (58, 59, and 100), pooled scRNA-seq data from non-adjacent tissues in the cohort were used. For this pooled reference only, we reduced computation time by randomly sampling 3000 cells each from four tissues (313, 364, 880, and 982) while preserving cell-type proportions, and by including all 565 available cells from tissue 330. scRNA-seq data from tissues 514 and 783 were excluded from this pooled reference because we prioritized tissues for which the corresponding ExSeq datasets contained a large number of high-quality cells (≥50 transcripts per cell). In total, this pooled reference consisted of 12 565 scRNA-seq cells from five tissues and was used to identify marker genes for tissues lacking directly matched scRNA-seq references.

Cell typing for the scRNA-seq datasets was already available [25]; therefore, these data were used only to detect tissue-specific cell-type markers. For each tissue-specific or pooled scRNA-seq dataset, gene expression profiles were normalized and scaled across all genes. Differential expression analysis was performed using Seurat [30] to identify genes enriched in each cell type, retaining only those with average log₂ fold change > 5 and adjusted *p* ≈ 0 (in Seurat, the smallest *P*-values are reported as 0, corresponding in practice to adjusted *p* ≤ 1 × 10⁻^200^). To avoid ambiguity, genes detected as markers in more than one cluster were conservatively filtered: if a gene showed adjusted *p* ≈ 0 in one cluster but not in others, it was retained only for that cluster; in all other cases of duplication, the gene was excluded. These tissue-specific markers were complemented with a reference panel of canonical markers [28], including genes for T cells, B cells, macrophages, fibroblasts, and tumor epithelial cells. The union of tissue-specific and canonical markers constituted the final panel used for cell-type assignment in the ExSeq data.

For each tissue independently, ExSeq gene expression profiles were processed and clustered using Seurat. Low-quality cells (<50 detected transcripts) were removed. The remaining cells were normalized, scaled, and reduced in dimensionality using PCA. To ensure robustness, clustering was performed across a grid of parameter combinations varying the number of significant principal components and the clustering resolution. The clustering resolution, a parameter of the FindClusters function in the Seurat R package [30], influences the modularity optimization step of the Louvain algorithm (lower clustering resolution values yield fewer, broader clusters, whereas higher values produce a larger number of finer clusters). For each parameter combination, enrichment of marker genes was assessed by differential expression analysis across clusters, and clusters were assigned only if they showed significant overexpression of marker genes relative to all other clusters (*q* ≤ 1 × 10⁻^9^), where *q* denotes the Benjamini-Hochberg false discovery rate-adjusted *P*-value; this definition applies throughout the manuscript. If a cluster was supported by a single marker, it was assigned directly. When multiple candidate markers supported a cluster, two cases were distinguished: (i) if all markers mapped to the same cell type and the most significant marker satisfied *q* ≤ 1 × 10⁻^9^, the cluster was assigned to that cell type; (ii) if markers mapped to different cell types, stricter rules were applied: clusters were retained only when one candidate had adjusted *p* ≈ 0 while the others did not, or when the leading candidate substantially outperformed the next-best marker (log₁₀ (*q*₁/*q*₂) < 0.3·log₁₀ (*q*₂) and *q*₁ ≤ 1 × 10⁻^9^). If both competing markers had *q* ≈ 0, or if these criteria were not met, the cluster was left unassigned. This stringent filtering ensured that only clusters with unambiguous marker support were labeled, while ambiguous clusters were excluded from downstream analyses.

Because clustering results depend on parameter choices (i.e. number of significant principal components and clustering resolution), we adopted a consensus labeling strategy. First, only clustering solutions (i.e. parameter combinations that yielded cell type assignment) with more than three inferred cell types were retained. Each cell was then clustered across all retained parameter combinations, and its assigned labels were recorded. The final label for each cell was defined as the most frequent assignment, provided it appeared in at least 20% of the retained combinations and in at least 10 clustering solutions (out of ∼200 total per tissue), thereby ensuring a robust annotation.

To assess the robustness of the cell-typing procedure, we performed a permutation analysis. For each tissue, raw ExSeq profiles were permuted 20 times by shuffling transcript counts across cells within each gene, thereby preserving the overall expression distribution of each gene while destroying true co-expression and cell-type structure. Each permuted dataset was processed using the full clustering and annotation pipeline described above. Statistical significance was evaluated by comparing the number of cells assigned to a consensus cell type in the real data to the null distribution from permuted datasets. For each permutation, the number of cells passing quality control and receiving a valid label was recorded. Observed counts from the real dataset were standardized relative to this null distribution, and a one-tailed *P*-value was calculated as the probability of observing an equal or greater number of assigned cells under the null (hereafter referred to as the normal-approximation permutation procedure). Across all 10 tissues, *p* ≈ 0 was obtained, reflecting the near absence of valid cell annotations in the permuted data and confirming the statistical significance of the observed clustering.

**Figure 1.**
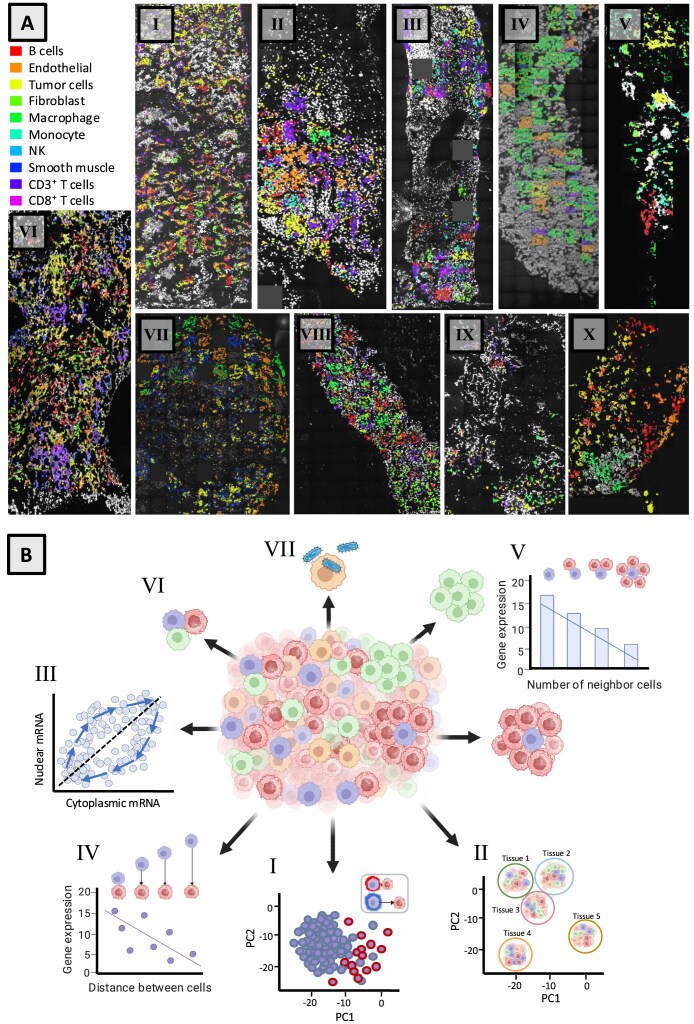
Breast cancer super-resolution dataset and InSituPREP workflow for analysis of cell states. (**A**) ExSeq profiling of single cells in 10 breast cancer tissues. A panel of 299 breast cancer-related genes was sequenced *in situ*, and segmented cells were annotated by cell type, shown in distinct colors. Biopsy/tissue identifiers: I, 330; II, 982; III, 364; IV, 514; V, 58; VI, 100; VII, 880; VIII, 313; IX, 783; X, 59. Details about these tissues, including their overall size, are provided in Supplementary Table S1. (**B**) Schematic of the InSituPREP workflow for detecting spatially associated transcriptional programs: (I) contribution of cell-cell proximity to within-cell type variability; (II) proximity-associated expression programs across datasets and patients; (III) influence of cell-cell proximity on future cell states; (IV) linear dependencies between gene expression and intercellular distance; (V) gene expression as a function of neighbor number; (VI) triplet cell interactions; (VII) bacterial presence and local effects on gene expression. Created in BioRender. Tal, G. (2026) https://BioRender.com/dgddkkj.

Across the 10 biopsies analyzed, we identified 10 major cell type clusters: B cells, endothelial cells, tumor cells, fibroblasts, macrophages, monocytes, NK T cells, smooth muscle cells, CD3⁺ T cells, and CD8⁺ T cells (Fig. 1 and Supplementary Fig. S2). Hepatocytes were not detected, even though six samples were liver metastases (Supplementary Table S1). Using MERFISH and scRNA-seq on consecutive sections of these same liver biopsies, Klughammer *et al*. [25] reported that hepatocytes constitute less than 4% of cells. Given this low abundance and the fact that ExSeq samples fewer cells per tissue compared with MERFISH and scRNA-seq, the absence of hepatocytes in our annotations is consistent with their low abundance.

### Dispersion in cell type explained by proximity-induced cell state

**Figure 2.**
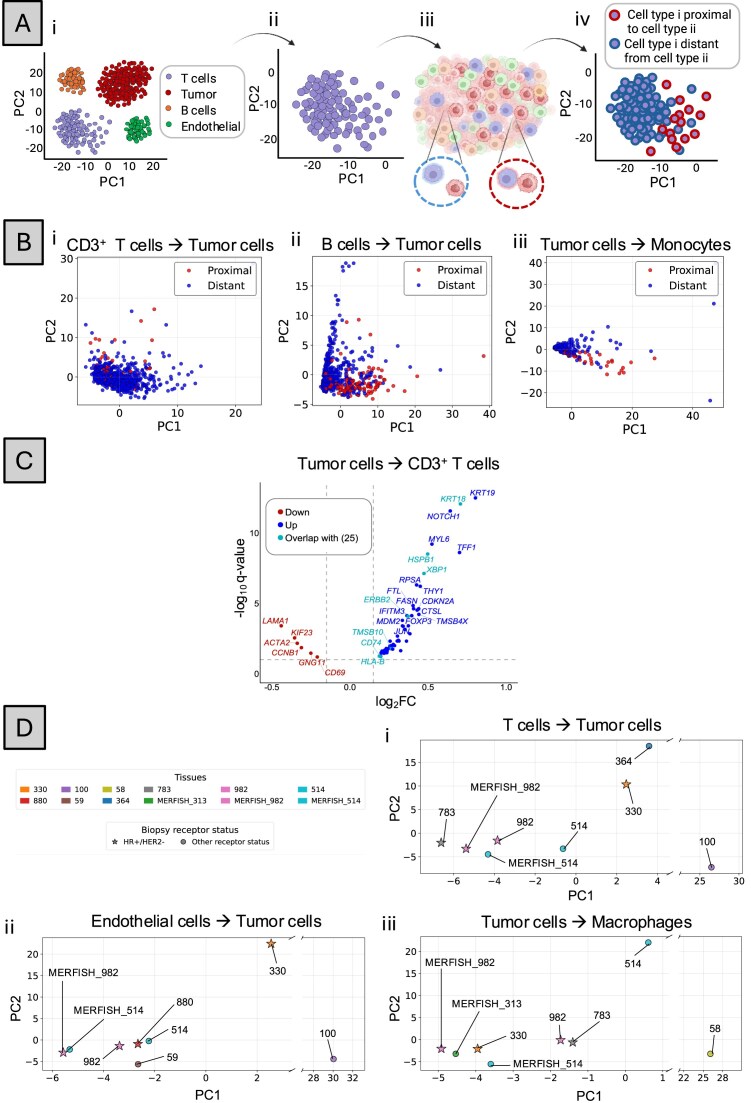
Proximity-related expression programs explain part of within-cell type variability and are shared across datasets and patients. (**A**) Schematic representation of cell types in expression-based PCA space (i). We reasoned that part of the variability observed within a cell type may reflect proximity-induced cell states (ii-iv). Created in BioRender. Tal, G. (2026) https://BioRender.com/kcyct53. (**B**) Examples of variability explained by proximity: (i) CD3⁺ T cells are more dispersed when adjacent to tumor cells (proximal, red) compared to distant cells (blue) (*q* = 2 × 10⁻^12^, permutation analysis); a similar effect is observed for B cells (*q* = 2 × 10⁻^95^) (ii), and for tumor cells in proximity to monocytes (*q* = 4 × 10⁻^16^) (iii). All cases of variability explained by proximity are in Supplementary Fig. S6 and Supplementary Table S2. (**C**) Volcano plots showing log2 fold change (FC) and significance (*q* < 0.1) for genes differentially expressed in tumor cells proximal versus distant from CD3⁺ T cells, shown here for tissue 100. Blue shows upregulated genes, red shows downregulated genes, and turquoise marks overlap with Klughammer *et al*. [25]. (**D**) Proximity-related expression programs are shared between ExSeq and MERFISH and show similarity across patients with similar receptor status (Supplementary Fig. S7 and Supplementary Table S5). Shown are three examples of proximity-induced programs: (i) T cells relative to tumor cells, (ii) endothelial cells relative to tumor cells, and (iii) tumor cells relative to macrophages. In each case, proximity-induced programs are displayed in PCA space for each tissue, with quantifications from consecutive sections profiled by ExSeq and MERFISH when available. Patients with HR⁺/HER2⁻ status are marked with a star; other receptor types are indicated by circles. ExSeq and MERFISH programs were significantly closer than expected by chance (e.g. *p *< 0.02 and *p *< 0.04 for biopsy 982 in i and ii, respectively; permutation analysis; overall *q* < 0.1 for all combinations excluding biopsy 514 in iii), as were programs from patients with HR⁺/HER2⁻ status (*p *< 0.004 in iii; permutation analysis).

As part of InSituPREP (Supplementary Fig. S3), we next introduce a module that quantifies how spatial proximity contributes to within-cell type heterogeneity. Following cell type clustering, several clusters exhibited notable dispersion in low-dimensional PCA space (Fig. 2A, B). We reasoned that part of this variability may reflect microenvironment-driven cell states, potentially induced by physical proximity between cell type *i* and cell type *ii*. Specifically, we asked whether type *i* cells proximal to type *ii* show a measurable shift in PCA embedding relative to those located farther away. To test this, type *i* cells were stratified by spatial distance to type *ii*, and their dispersion in PCA space was quantified as described below.

For each tissue and cell type *i*, cells were divided into two groups: ‘proximal’, defined as type *i* cells within ≤ 1 µm (cell-boundary distance) of type *ii* cells, and ‘distant’, defined as type *i* cells located > 1 µm from type *ii* cells (Supplementary Fig. S4; 3D proximity demonstration in Supplementary Fig. S5). Throughout this manuscript, cell-boundary distance is defined as the minimal Euclidean distance between RNA molecules belonging to the two cells, and all the reported distance measurements are expressed in pre-expansion units. For each cell type pair (type *i* and type *ii*) in each tissue, we standardized the expression levels for type *i* cells by z-scoring, and computed PCA.

Dispersion of type *i* cells was quantified in the PC1-PC2 embedding (Supplementary Fig. S6). An overall centroid for type *i* was defined from all cells, and the dispersion of the proximal subset was measured as the sum of Euclidean distances between proximal cells and this centroid. Significance was assessed by permutation testing with 10 000 shuffles of proximal-distant labels, preserving the observed proximal subset size. For each permutation, the analogous dispersion was calculated, and a normal distribution was fit to the permuted values. The z-score of the observed dispersion was subsequently used as one component of a combined test statistic, as described below. Proximity contribution to variance was defined as the ratio of the dispersion of proximal cells to the total dispersion.

As a complementary measure of proximity-induced cell state, we quantified the separation between proximal and distant subsets as the Euclidean distance between their centroids in PC1-PC2 space. As before, 10 000 shuffles of proximal-distant labels, preserving the observed proximal subset size, were performed. For each permutation, the centroid distance was recalculated, and a normal distribution was fit to the permuted values. The z-score of the observed centroid distance was subsequently used as another component of a combined test statistic, as described below.

For each tissue and cell-type pair, the two previously computed z-scores were combined into a unified test statistic. To account for testing two correlated measures, we defined Z_max _= max (Z_single-centroid_, Z_two-centroids_). A one-sided *P*-value for Z_max_ was computed using the normal-approximation permutation procedure. Results were considered significant at *q* ≤ 0.05.

The analysis was performed across all cell type combinations in each tissue. With 10 identified cell types and 10 tissues, 900 combinations were possible; however, because not all cell types were present in every tissue and we required at least one proximal and one distant cell for each type *i*–type *ii* pair, the final number of examined combinations was 232. Remarkably, proximity-induced cell states were statistically significant in 70 of the 232 examined combinations (Supplementary Table S2).

We validated these findings globally by permutation analysis. For each tissue–cell-type pair, proximal–distant labels were randomly permuted 10 000 times while preserving the observed proximal group size, and the full analysis was repeated for each shuffled dataset. Across 232 tissue–cell-type combinations, only 3 passed the significance threshold (*q* ≤ 0.05). Given this chance rate (3/232), the observed 70/232 significant cases are highly unlikely (binomial test, *p* = 2 × 10⁻^73^).

We performed two additional analyses: [1] a sensitivity check for potential segmentation artifacts and [2] a robustness assessment in an alternative embedding (*t*–SNE). These analyses are provided in the Supplementary Material.

### Detection of proximity-induced genes

InSituPREP also incorporates differential expression analysis to identify proximity-induced genes, enabling systematic detection of transcriptional changes associated with spatial context. For each of the 10 tissues and for each cell type, proximity-induced genes were detected using DESeq2 [31] differential expression analysis. Analyses were performed separately for tumor and non-tumor cell types. In each case, cells were stratified relative to the opposing compartment: cells within ≤1 µm cell-boundary distance were designated proximal, and all others distal.

To evaluate statistical significance, we combined DESeq2 tests with a permutation-based framework: proximity labels were randomly shuffled across cells (1000 iterations), and the DESeq2 pipeline was rerun for each permuted dataset. For each gene, an empirical permutation-derived *P*-value was computed. Genes were filtered based on their log₂ fold-change, and both nominal *P*-values (from DESeq2) and permutation-derived *P*-values were corrected for multiple testing as before (Supplementary Table S3).

To evaluate the consistency of spatially defined transcriptional responses across tissues, we compared proximity-induced genes identified in tumor versus non-tumor cells, as well as in the reciprocal direction, for each cell type. To assess whether overlaps of proximity-induced genes between tissues exceeded random expectation, we performed a permutation-based significance test. For each tissue and each list of proximity-induced genes for a given cell-type pair, random gene sets of equal size were sampled 100 000 times from the background of all expressed genes, and intersections were recalculated. A permutation-based *P*-value was then computed. For each tissue pair and cell-type pair, we reported: (i) the number of proximity-induced genes identified in each tissue, (ii) the intersecting gene set, (iii) the intersection size, (iv) the permutation-derived *P*-value, and (v) the corrected *q*-values (Supplementary Table S4).

Differential expression analysis was also performed on MERFISH gene expression data, which was available for four samples (Biopsy identifiers: 313, 514, 880, and 982). For each tissue and each tumor–non-tumor cell type pair, as well as the reciprocal non-tumor–tumor pair, proximity labels were defined using the same criteria as in the ExSeq analysis (cells within ≤ 1 µm considered proximal). DESeq2 was then applied to these data, yielding gene-level statistics including *P*-values.

### ExSeq-MERFISH comparison and hormone receptor-dependent clustering

InSituPREP incorporates a comparative module to assess proximity-induced signatures across hormone receptor subtypes and between ExSeq and MERFISH data. Several hormone receptor types were present across the 10 analyzed breast cancer tissues. For a subset of samples, ExSeq and MERFISH datasets were obtained from adjacent sections of the same specimen. These datasets were used to directly compare cell-cell proximity-induced genes between different hormone receptor types and across the two measurement platforms (Supplementary Fig. S7).

For each non-tumor cell type, differential expression was assessed between cells proximal and non-proximal to tumor cells using DESeq2. The reciprocal analysis was performed for tumor cells with respect to proximity to non-tumor cells. These analyses were applied across all tissues, incorporating both ExSeq and MERFISH datasets from adjacent sections of the same samples when available.

To ensure comparability across tissues, only genes expressed in all tissues for a given cell type were retained. *P* values were then transformed as − log_10_ (*p*). Exact zeros in DESeq2 *P* values (which make − log_10_ undefined) were replaced with a small value equal to 0.8 × the global minimum nonzero *P* value observed for that cell type. The resulting tissue-by-gene *P* values matrix was Z-score standardized per gene across tissues, and each tissue’s standardized *P* value profiles were then projected into principal component analysis (PCA) space.

After projecting the *P* values of the proximity-induced genes into PCA space, we evaluated whether PC1 and PC2 explained more variance than expected by chance. Significance was assessed by permutation analysis: 10 000 permutations were performed in which values within each gene were randomly shuffled across tissues, thereby preserving gene-wise distributions while destroying tissue-specific structure. PCA was recomputed for each permuted dataset, and for each PC, a permutation-based *P*-value was calculated.

To quantify ExSeq-MERFISH agreement, we computed Euclidean distances in PC1-PC2 space between ExSeq and MERFISH representations of the same tissue (e.g. ‘514’ vs. ‘MERFISH_514’). Significance was assessed by the normal-approximation permutation procedure. In each permutation, the *P*-value profile of each gene was independently shuffled across tissues, preserving gene-wise distributions while destroying tissue-specific structure. The permuted matrix was then Z-score standardized per gene, and PCA was recomputed. To ensure distances were numerically comparable across permutations, each permuted PC dimension was linearly rescaled to the scale of the corresponding observed PC dimension using an affine transformation, applied separately to PC1 and PC2. This rescaling preserved relative positions within each permuted configuration while aligning axis scales to those of the observed PCA.

For tissues with similar hormone receptor and HER2 status, we analogously assessed whether ExSeq samples clustered more tightly than expected. Specifically, we computed the mean pairwise Euclidean distance among HR⁺/HER2⁻ ExSeq tissues in PC1-PC2 space and compared it to a permuted null distribution generated by per-gene shuffling across tissues. This analysis was performed identically to the ExSeq-MERFISH comparison described above. We examined HR⁺/HER2⁻ clustering only in the metastasis samples, excluding the primary biopsies 58 and 59, because metastases and primary breast tumors may exhibit different characteristics that could influence clustering outcomes (these primary samples were therefore not marked as HR⁺/HER2⁻ in Fig. 2 and Supplementary Fig. S7 to clarify the analysis). As a control, we repeated the analysis after grouping the primary samples (58 and 59) with the HR⁺/HER2⁻ metastasis samples. This control did not yield significant clustering of HR⁺/HER2⁻ samples across any cell-type pair at a significance threshold of 0.05.

Significance values for ExSeq-MERFISH concordance, program similarity across HR⁺/HER2⁻ patients, and PCA embeddings are provided in Supplementary Table S5.

### RNA velocity

To capture dynamic transcriptional trajectories, InSituPREP includes a spatial RNA velocity module, implemented as follows:

Data preprocessing for RNA velocity analysis

Unspliced and spliced RNA read counts were extracted according to subcellular localization [27], nuclear or cytoplasmic, respectively, using 3D segmentation masks delineating nuclear and whole-cell boundaries. This spatial assignment enabled classification of RNA molecules for subsequent RNA velocity modeling. To account for variability in sequencing depth across cells, cytoplasmic read counts were normalized at the per-cell level. For each cell, a gene’s cytoplasmic expression was divided by the total cytoplasmic counts for that cell and multiplied by the mean total cytoplasmic count across all cells from all tissues. This procedure reduces artifacts caused by extreme total expression values in outlier cells.

Calculation of gamma (γ)

Following [26], for each gene in each cell, the spliced (cytoplasmic) RNA at a future time step S (T) was computed as S (0)+v × T, where S (0) is the initial spliced count, T is the time step (determined below), and v is the RNA velocity. Velocities were calculated as v = u−γs, where u and s are the unspliced (nuclear) and spliced (cytoplasmic) counts, respectively, and γ is the degradation rate of the spliced counts.

γ was estimated for each gene from the steady-state subset of cells by regressing spliced (cytoplasmic) counts against unspliced (nuclear) counts [26]. To reduce stochastic variability and measurement noise, cytoplasmic and nuclear counts for each cell were replaced with the mean counts of the cell and its K nearest neighbors in gene expression space [26]. Neighbors were identified by performing PCA on the normalized cytoplasmic expression matrix and defining proximity based on Euclidean distance in the first three principal components (PC1-PC3). We evaluated a range of K values for all 299 genes and found that γ estimates were stable around K = 30 (Supplementary Fig. S8B), which was used in all subsequent analyses.

In the gene phase portrait, i.e. plots showing the relationship between unspliced (nuclear) and spliced (cytoplasmic) transcript abundances across cells, only the upper and lower quantiles of cells were used for γ estimation [26]. This approach improves the linear regression fit, as cells with high or low total expression (nuclear + cytoplasmic) conform best to the steady-state line, whereas intermediate values often form an almond-shaped distribution (Supplementary Fig. S8A). Using K = 30, we evaluated quantile cutoffs of 5%, 7.5%, and 10%, and found that cutoffs between 5–10% produced similar γ values (≤20% variation among all genes). Based on these results, we used 7.5% quantile cutoffs in all subsequent analyses.

Selection of genes with reliable γ

We visually inspected the phase portrait of each gene and identified 62 genes with the expected shape and a clear linear trend (Supplementary Fig. S8A). These genes had higher expression levels than others, suggesting that lowly expressed genes cannot be reliably fitted to the RNA velocity production–degradation model due to increased stochastic noise. We used these 62 genes to determine an expression cutoff for gene selection. An expression threshold of 0.038 maximized the F-score (Supplementary Fig. S8C), which accounts for both precision and recall. Genes with a maximum normalized nuclear or cytoplasmic expression ≥ 0.038 were considered to have reliable γ estimates. In total, 84 genes met this criterion and were used for calculating future cell states.

Determine the time step (T)

Sensitivity analyses were performed to determine the time step (T). For each cell type, we predicted future states for each cell based on the calculated velocities using a range of T values. Following [26], T was constrained so that the total spliced (cytoplasmic) expression per cell at the future time point did not deviate by more than 10% from its initial value. We tested T values from 1 to 8, all within this constraint, and found that T = 3 produced robust results (Supplementary Fig. S8D). This value was used in all subsequent analyses.

Calculate future states (S (T = 3))

**Figure 3.**
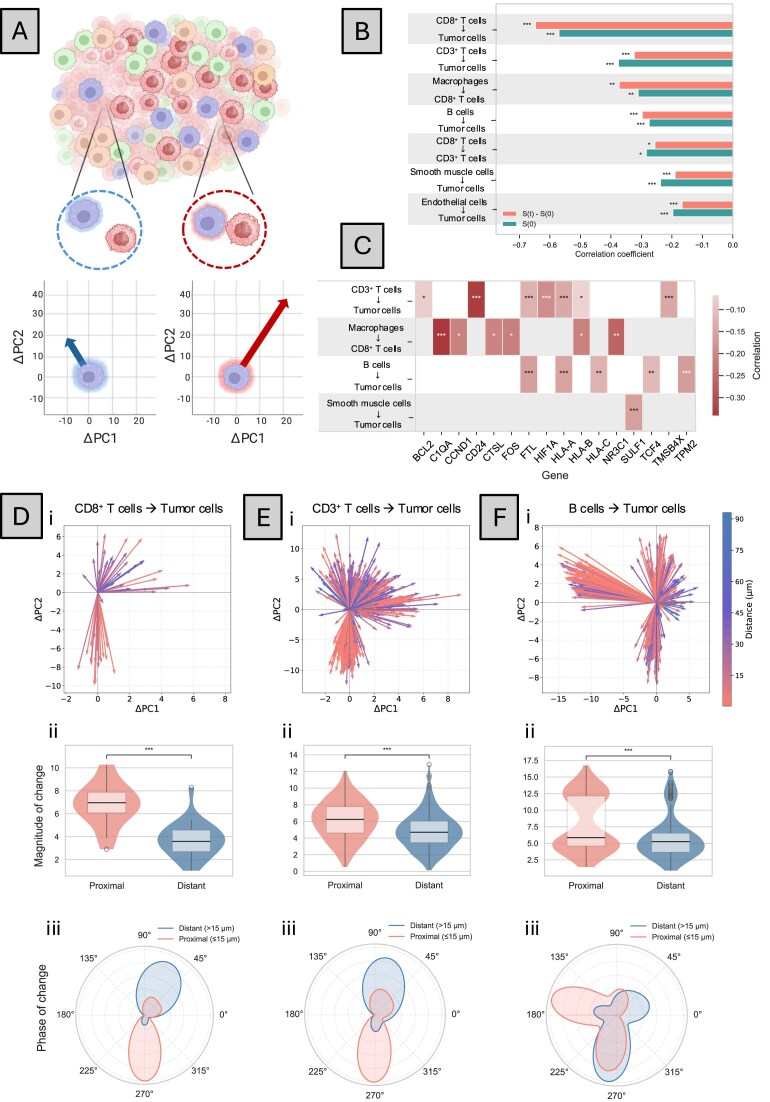
Current and future cell states correlate with distance to other cell types. (**A**) Schematic: proximity between two different cell types alters future states, illustrated by arrows in PCA space (red = proximal cells, blue = distal cells). Created in BioRender. Tal, G. (2026) https://BioRender.com/gz54aur. (**B**) Correlation of physical distance to other cell types with the magnitude of future state change (red) and current state (green; distance from origin in PCA space). In all examples, correlations are significant for both current and future states (*q < 0.05; **q < 0.01; ***q < 0.001; permutation-based test). Correlations for all cell type combinations are in Supplementary Fig. S9. (**C**) Genes in each cell type whose RNA velocities significantly correlated with distance to another cell type (Pearson correlation; permutation analysis; *q < 0.1; **q < 0.05; ***q < 0.01), potentially contributing to observed state differences. All detected genes are in Supplementary Fig. S12. (D–F) Three examples of the link between cell-cell proximity and future cell states (chosen from significant cell type combinations; Supplementary Figs S9, S10). (**D**) CD8⁺ T cells relative to tumor cells. (i) Quiver plots in PCA space colored by continuous distance (blue = farthest, red = closest; color bar indicates cell-cell distance). (ii) Box-violin plots of the magnitude of state changes in proximal (≤15 µm; red) and distant (>15 µm; blue) cells. (iii) Phase comparison shown as polar histograms for proximal (≤15 µm; red) and distant (>15 µm; blue) cells. Significant magnitude (*p* = 1 × 10⁻⁶; Mann-Whitney U (MWU)) and phase differences were observed. (**E**) Same analyses as in (**D**) but for CD3⁺ T cells relative to tumor cells (*p* = 1 × 10⁻¹²; MWU). (**F**) Same analyses as in (**D**) but for B cells relative to tumor cells (*p* = 5 × 10⁻⁶; MWU).

The velocity-predicted spliced (cytoplasmic) RNA abundance at T = 3, S (T = 3), was computed for each cell as S (0)+v × T. To ensure comparability across cells, S (T = 3) was scaled by the mean total S (T = 3) across all cells. This normalized expression matrix was subjected to PCA, and the first two principal components (PC1 and PC2) were used to visualize both current and projected future cell states (Fig. 3 Di, Ei, and Fi).

Correlating physical distance between cell types and the magnitude of change in cell state

For a given primary cell type X, we calculated the minimum cell-boundary distance from each cell to any cell of a different neighbor type Y. The magnitude of change in cell state was defined as the Euclidean distance in PCA space between the initial state S (0) and the projected future state S (T). Additionally, the magnitude of the initial state was computed as the Euclidean distance from S (0) to the origin in PCA space. For each primary–neighbor cell type pair (excluding self-pairs), Pearson’s correlation was calculated between the minimum physical distance and the magnitude of change in cell state for the primary cell type. Significance was assessed by permutation-derived *P*-values, requiring *q *< 0.05 across all tested pairs. The same procedure was applied to correlations involving the magnitude of the initial state.

The above analysis was performed both on all tissues combined (Supplementary Fig. S9, and phase analysis as described below in Supplementary Fig. S10) and on each tissue individually (Supplementary Fig. S11).

Correlating physical distance between cell types and the phase of change in cell state

The analysis described above was also performed on the phase, and not only the magnitude of the change in cell state. The phase of the change in cell state was defined as the angle between the initial and future positions of a cell in PCA space, projected onto the first two principal components (PC1 and PC2) (Supplementary Fig. S10).

Permutation testing

To assess the robustness of the observed correlations, we performed a permutation test (10 000 iterations) for each primary–neighbor pair. Distance values were shuffled among primary cells, preserving their distribution while disrupting spatial relationships. *P*-values were computed via a normal-approximation permutation procedure.

Overall (Supplementary Fig. S9), the spatial RNA velocity analysis identified 11 cell type combinations with significant negative correlations, where shorter distances were associated with larger future state changes, and 8 combinations with positive correlations, where greater distances were associated with larger changes, between physical distance to other cell types and the magnitude of *future* state change (*q* < 0.05). In parallel, 18 combinations showed significant negative correlations, and 9 showed positive correlations between distance and the magnitude of the *current* state (*q* < 0.05). Thus, both current and predicted future states are associated with proximity to other cell types.

Correlation between gene-specific RNA velocity and the physical distance

To assess whether gene-specific velocities in a given cell type were associated with physical proximity to other cell types, we computed correlations between gene-level velocity and spatial distance. For each primary–neighbor cell type pair and each gene, we extracted the gene’s velocity across all primary cells and correlated these values with the corresponding minimum distances to the neighbor cell type. Pearson’s correlation coefficients were calculated for all genes within each primary–neighbor pair, and *q*-values were calculated. This analysis identified genes whose transcriptional dynamics are spatially associated with the presence or proximity of specific cell types in the tissue microenvironment. Matching single-cell RNA-seq (scRNA-seq) data were used to exclude genes with very low expected expression in the primary cell type: genes detected in fewer than 20% of sequenced single cells of that type were removed from the analysis (Supplementary Fig. S12). An exception is *CD24*, which was retained due to its reported upregulation in activated T cells [32, 33]. The same scRNA-seq-based 20% expression filtering was applied in subsequent analyses unless otherwise stated.

### The effect of the distance between cells on gene expression

InSituPREP incorporates a continuous distance-response module to quantify how gene expression depends on intercellular distance. Specifically, we quantified how the distance of cells of type X from cells of type Y influenced gene expression in primary cells of type X. The following steps were performed:

Data preprocessing and detecting linear dependence

Cell-Cell distance calculation: To quantify how the distance of cells of type Y influenced gene expression in primary cells of type X, we calculated pairwise distances for each tumor sample and cell-type pair. Specifically, within each 40 × objective field of view (FOV; 100 × 100 µm), we computed the cell-boundary distance between every primary cell and its nearest neighbor of the other type. Each cell-type pair was processed bidirectionally (e.g. B cells to tumor cells and tumor cells to B cells). For downstream analyses, we retained only primary cells of type X that had at least one neighboring cell of the other type within the same FOV, i.e. cells for which a valid minimum distance to the other cell type could be calculated.

Gene filtering by low expression or sparse detection: To reduce noise from lowly expressed or sparsely detected genes, we applied two filtering criteria to genes in the primary cell type within each tissue: [1] 98th percentile of expression ≤ 10 counts, or [2] expression detected in ≤ 20 cells. Genes meeting either criterion were set to missing (NaN) and excluded from downstream analyses.

Linear regression of expression versus distance: For each retained gene in each cell type pair and tissue, we performed a linear regression to model the relationship between its expression in the X cells and the minimal distances of the X cells to the Y cells. In addition, we performed 100 random permutations per retained gene in each cell type pair and tissue. For each permutation, distance values were shuffled while maintaining the original expression. We performed a linear regression to model the relationship between the gene expression in the X cells and the shuffled distances of the X cells to the Y cells. We then calculated the average of the 100 permuted R² values for each gene. Regression outputs included the slope, original R², average permuted R², original *P*-value, number of contributing cells, 98th percentile of gene expression, and 98th percentile of cell-to-cell distance.

Statistical significance

Statistical significance of observed linear relationships was assessed by computing *P*-values for each gene’s regression coefficient using the statsmodels package in Python with default parameters. A gene was considered significant if its resulting *q*-value was less than 0.05.

Post-processing filter

Gaussian smoothing and R² filtering: To assess robustness of gene–distance relationships, we applied Gaussian smoothing (σ = 5) to gene expression vectors sorted by distance. Smoothed expression was re-regressed on distance, and a new R² was computed. Genes with Gaussian R² < 0.5 were excluded. Regression outputs included the Gaussian R².

Scoring and ranking of gene-distance associations

To prioritize gene – cell type – tissue associations with robust and interpretable spatial trends, we assigned a composite score to each association based on multiple features. This ranking was used solely for visualization and prioritization purposes and does not affect the statistical identification of significant gene-distance associations. For each feature, we computed its rank across all associations (*n* associations), using ascending or descending order based on biological desirability (e.g. lower FDR is better, higher original R² is better). Specifically:

- Features ranked in descending order (higher is better):
  - Absolute value of the regression slope
  - Original R² from unsmoothed expression
  - R² from Gaussian-smoothed expression
  - Number of cells contributing to the regression
  - 98th percentile of expression of the gene in question in cells from type X (effective dynamic range of the response variable)
  - 98th percentile of cell-to-cell distance between the cells from type X and Y (effective dynamic range of the predictor variable)
- Features ranked in ascending order (lower is better):
  - Mean R² across permutations
  - Global FDR-adjusted *P*-value

Each feature’s rank was reversed (e.g. *n *− rank) so that higher values always contributed positively to the total score. The final score for each association was calculated as the sum of the reversed ranks across all eight features. Associations were then sorted in descending order of score, with higher scores indicating more robust gene–distance relationships.

The resulting linear dependencies between gene expression and intercellular distance are summarized in Supplementary Table S6, with examples shown in Fig. 4 and Supplementary Figs S13–S27.

**Figure 4.**
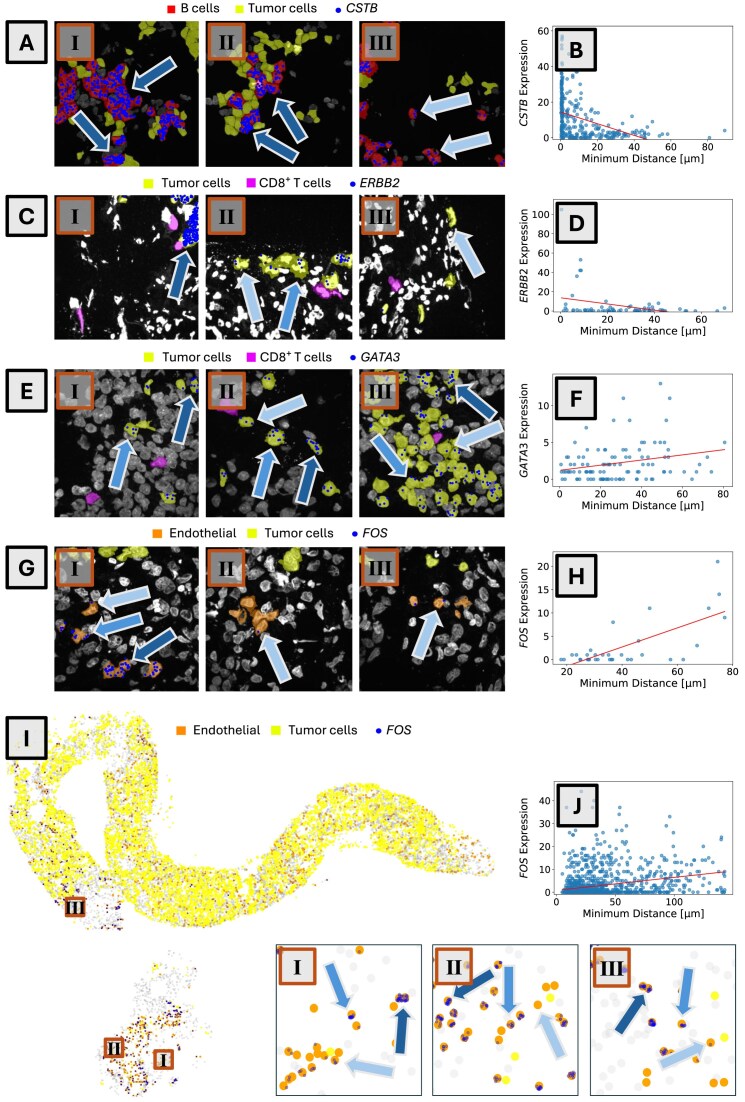
Proximity-dependent cell states. (**A**) *CSTB* expression in B cells relative to tumor cells in biopsy 59. Sequencing read locations for *CSTB* (blue dots) are overlaid on DAPI-stained nuclei, together with segmentations of B cells (red) and tumor cells (yellow). Only B-cell and tumor-cell segmentations are shown. Representative 40 × fields of view (100 × 100 µm) are displayed. B cells proximal to tumor cells show higher *CSTB* expression (solid blue arrows) compared with distal B cells (hollow blue arrows). (**B**) Linear dependency of *CSTB* expression in B cells on distance to tumor cells (*p* from regression coefficient using t-test, statsmodels; *q* = 2 × 10⁻¹¹, slope = –0.33). (**C–D**) Same analyses as in (A–B), for *ERBB2* expression in tumor cells relative to CD8⁺ T cells in biopsy 364. *ERBB2* expression decreases as a linear function of distance from CD8⁺ T cells (*q* = 3 × 10⁻^2^, slope = -0.32). (**E–F**) Same analyses as in (A–B), for *GATA3* expression in tumor cells relative to CD8⁺ T cells in biopsy 982. *GATA3* expression increases as a linear function of distance from CD8⁺ T cells (*q* = 4 × 10⁻^2^, slope = 0.03). (**G–H**) Same analyses as in (A–B), for *FOS* expression in endothelial cells relative to tumor cells in biopsy 313. *FOS* expression increases as a linear function of distance from tumor cells (*q* = 7 × 10⁻⁵, slope = 0.21). (**I–J**) Same analyses as in (G–H), i.e. *FOS* expression in endothelial cells relative to tumor cells, but using a consecutive slice section analyzed via MERFISH in biopsy 514. *FOS* again shows expression increases as a linear function of distance from tumor cells (*q* = 9 × 10^−24^, slope = 0.06). For panel (**I**) only, each subpanel measures 200 × 200 µm, and the overall imaged area is approximately 5400 × 6200 µm.

For four out of the 10 samples, matching MERFISH data from consecutive sections were available in the Human Tumor Atlas (Biopsy identifiers 313, 514, 880, 982). The same analysis pipeline used for ExSeq data was applied to the MERFISH datasets, with two key modifications. First, intercellular distances were computed as Euclidean distances between cell centroids rather than between the closest RNA molecules. This adjustment was necessary because MERFISH has lower spatial resolution than ExSeq, making centroid-based distances a more robust measure of cell-cell proximity. Second, to ensure comparability with the ExSeq analysis, which was limited to distances within a single 40 × field of view (FOV), we applied a maximum distance threshold of 145 µm to the MERFISH data. This value approximates the diagonal of a 100 µm square and thus corresponds to the upper bound of intercellular distances within one ExSeq FOV. All downstream steps, including gene filtering, linear regression, permutation testing, global FDR correction, Gaussian smoothing, and composite scoring, were performed identically to the ExSeq workflow. For MERFISH, analyses were run using two Gaussian-smoothed R² thresholds (0.5 and 0.3) to evaluate robustness.

The resulting linear dependencies between gene expression and intercellular distance using MERFISH data are summarized in Supplementary Table S7, with examples shown in Fig. 4 and Supplementary Figs S28, S29, and overlap with the ExSeq results presented in Supplementary Table S8.

### The effect of number of neighboring cells on gene expression

To complement the distance-based analyses, InSituPREP models gene expression as a function of the number of neighboring cells. We quantified how the number of neighboring cells of type Y influenced gene expression in primary cells of type X. The following steps were performed:

Data preprocessing and detecting linear dependence

For each tumor sample and cell type pair, genes with no expression in any primary cells were removed, followed by variance filtering to retain the top 20% most variable genes in the primary cells. For each primary cell type X, neighbor cell type Y, and tissue, we identified all Y cells within a 15 µm distance cutoff, which represents the distance between the closest pair of RNA molecules from the two cells. To mitigate data imbalance, neighbor-count bins (cells grouped by the same number of neighbors) containing fewer than 10 cells were excluded. In addition, cell type pairs with fewer than four valid bins were discarded to reduce noise. For each gene, we performed a linear regression to model the relationship between its expression in the X cells and the number of neighboring Y cells.

Statistical significance

Statistical significance of observed linear relationships was assessed by computing *P*-values for each gene’s regression coefficient using the statsmodels package in Python with default parameters. A gene was considered significant if its resulting *q*-value was less than 0.05.

Addressing data imbalance

Because most cells had few neighbors, the distribution of neighbor counts was skewed. This imbalance risked biasing the regression toward low-neighbor-count groups, potentially masking broader trends. To address this, we implemented an iterative sampling approach. In this method, we performed 1000 iterations where, for each iteration, a balanced dataset was constructed by sampling 10 cells from each valid neighbor-count bin. The final model’s slope was calculated as the average of the slopes from these 1000 regressions, with the intercept subsequently optimized to the original data. The iterative sampling approach yielded a more stringent set of 70 significant genes, compared to 109 detected by a standard (i.e. without accounting for data imbalance) linear regression, with 50 genes overlapping between the two methods. Note that the reported number of significant genes reflects detections within specific primary–neighbor cell type pairs in individual tissues, rather than the number of unique genes.

The analysis was repeated with distance cutoffs of 10 and 20 µm to assess the robustness of the 15 µm results. Using the iterative sampling approach, 51 significant genes were identified at the 10 µm cutoff, 50 of which overlapped with the 70 significant genes identified at 15 µm. At the 20 µm cutoff, 70 genes were identified, 56 of which overlapped with the 15 µm set.

Notably, the iterative sampling approach yielded results consistent with two alternative approaches we tested. The first alternative uses the 1000 slope-intercept pairs from the sampling iterations to generate 1000 distinct regression lines, and then performs a final, single linear regression on the collection of all these lines to determine an overall trend. This approach revealed 253 significant genes, out of which 70 overlapped with the 70 genes detected via iterative sampling.

The second alternative addresses the data imbalance using a Weighted Least Squares regression [34]. In this approach, each cell is assigned a weight based on the inverse probability of its neighbor-count group, which gives greater influence to cells from rarer, high-neighbor-count bins. We tested two variations of this: one using these raw inverse-probability weights and another using weights that were normalized to sum to one. These two variations each yielded 125 significant genes (identical between the sub-methods), 61 of which overlapped with the 70 genes detected using iterative sampling. Overall, the results of the iterative sampling approach yield robust results with respect to the distance cutoff and alternative approaches, and therefore, we proceeded with this approach as the primary analysis.

Post-processing filter and consensus gene set

The same scRNA-seq-based expression filtering was applied, with genes detected in fewer than 10% of cells excluded. To focus on the most robust findings, we defined a consensus gene set comprising genes that were consistently identified as significant across all analysis methods and distance cutoffs described above. Finally, we repeated the same analysis described above for cases where the primary and neighboring cells were of the same type. This analysis identified 48 significant associations spanning 8 different cell types.

**Figure 5.**
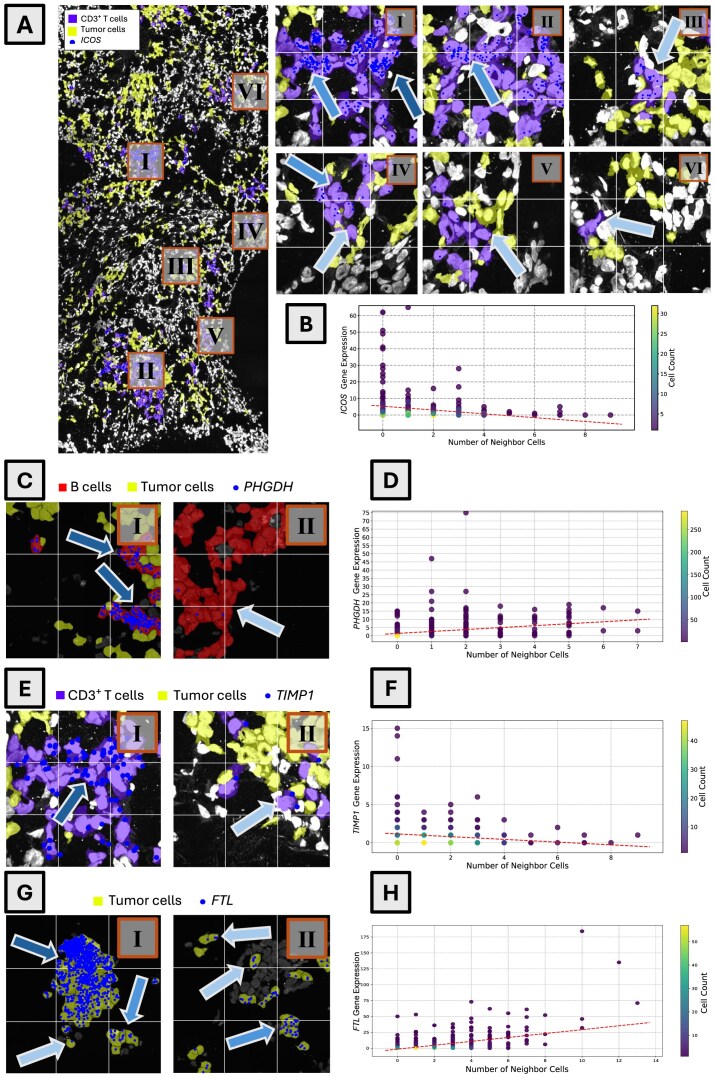
Neighboring cell-dependent cell states. Linear dependency between the number of neighboring cells and gene expression in a primary cell. (**A**) *ICOS* expression in CD3⁺ T cells relative to the number of neighboring tumor cells in biopsy 100. Sequencing read locations for *ICOS* (blue dots) are overlaid on DAPI-stained nuclei, together with segmentations of CD3⁺ T cells (magenta) and tumor cells (yellow). Only T-cell and tumor-cell segmentations are shown. A full tissue section and representative 40 × fields of view (100 × 100 µm) are displayed. CD3⁺ T cells with fewer neighboring tumor cells show higher *ICOS* expression (solid blue arrows) compared with T cells surrounded by many tumor cells, which show lower *ICOS* expression (hollow blue arrows). (**B**) Decreasing linear dependency of *ICOS* expression in CD3⁺ T cells on the number of neighboring tumor cells (*p* from regression coefficient using t-test, statsmodels; *q* = 5 × 10⁻³, slope = –1.15). (**C, D**) Same analyses as in (A–B), for *PHGDH* expression in B cells relative to tumor cells in biopsy 59, demonstrating an increasing linear dependency of *PHGDH* expression on the number of neighboring tumor cells (*q* = 2 × 10⁻^19^, slope = 1.18). (**E, F**) Same analyses as in (A–B), for *TIMP1* expression in CD3⁺ T cells relative to tumor cells in biopsy 100, demonstrating a decreasing linear dependency of *TIMP1* expression on the number of neighboring tumor cells (*q* = 2 × 10⁻², slope = –0.18). (**G, H**) Linear dependency between the number of neighboring cells and gene expression when the primary and neighboring cell types are the same. *FTL* expression in tumor cells increases with the number of neighboring tumor cells in biopsy 59 (*q* = 6 × 10^-24^, slope = 2.89).

The resulting linear dependencies between gene expression and the number of neighboring cells are summarized in Supplementary Table S9, with examples shown in Fig. 5 and Supplementary Figs S30, S31. Linear dependencies for same-type neighbor effects are summarized in Supplementary Table S10, with examples in Supplementary Figs S32, S33.

### Triplets analysis

**Figure 6.**
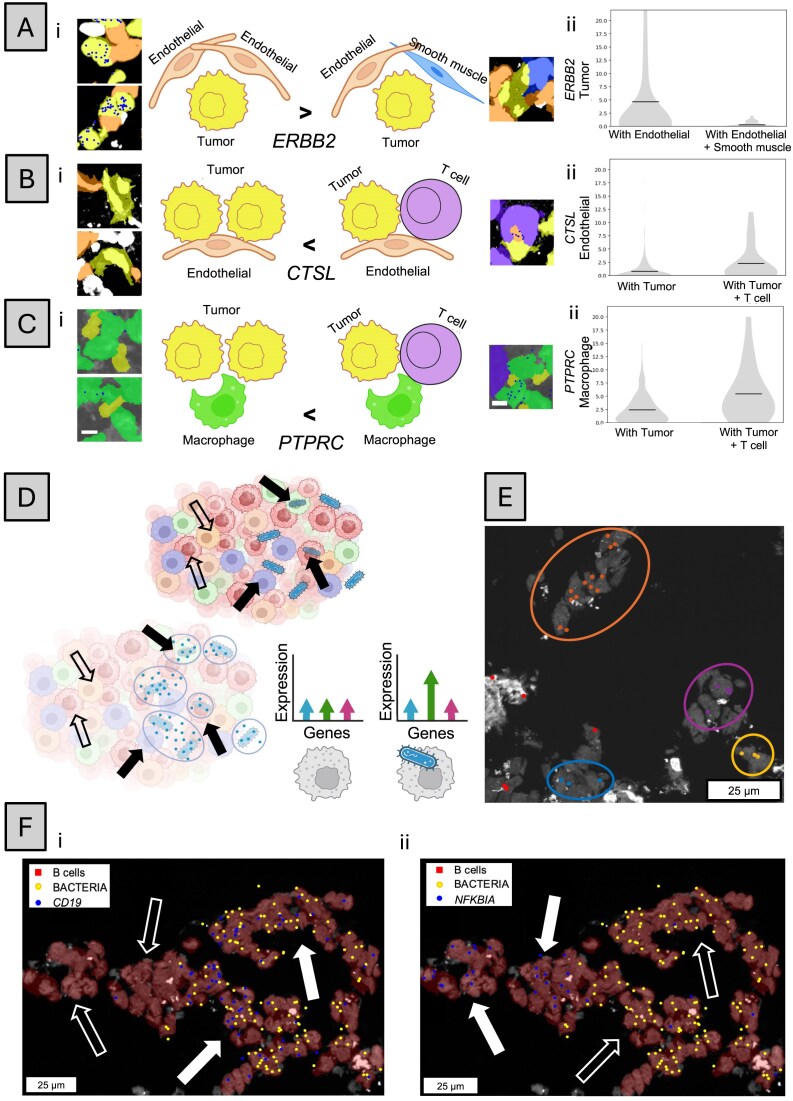
Local effects of cell triplets and bacteria on cell state. (A-C) Cell triplets influence gene expression. (**A**) *ERBB2* expression in tumor cells is higher when a tumor cell is surrounded by endothelial cells compared with both endothelial cells and smooth muscle cells. (i) Schematic and representative snippets of *ERBB2* expression (blue dots) in these triplets (cells colored as indicated). (ii) Violin plot (*p* from DESeq2 [31], *q* = 4 × 10^-4^, log₂ fold change = 3.84). (**B**) *CTSL* expression in endothelial cells is lower when surrounded by tumor cells compared with both tumor cells and T cells (i–ii as in A; *q* = 2.2 × 10^-2^, log₂ fold change = –1.51). (**C**) *PTPRC* expression in macrophages is lower when surrounded by tumor cells compared with both tumor cells and T cells (i, schematic; ii, violin plot; *q* = 3.3 × 10⁻², log₂ fold change = –1.15). Scale bar: 6 µm (applies to A–C). A–C were created in BioRender. Modi, S. (2026) https://BioRender.com/k540sel. (**D**) Schematic of bacteria detection within the biopsies via *in situ* sequencing of 16S rRNA in parallel to mRNA sequencing. Bacterial transcripts are clustered into objects, and then differentially expressed genes are detected in cells proximal to bacterial objects versus distant. Created in BioRender. Tal, G. (2026) https://BioRender.com/syosnel. (**E**) Four spatial regions in biopsy 58 are shown where clusters of 16S rRNA reads indicate the presence of bacteria in a statistically significant manner (*p* = 2 × 10^-14^, orange oval; *p* = 3 × 10⁻⁶, purple oval; *p* = 1 × 10⁻³, yellow oval; *p* = 3 × 10⁻³, blue oval; permutation analysis). (**F**) Because 16S rRNA detection in biopsy 58 was performed in parallel with gene sequencing, bacterial presence could be correlated with gene expression. (i) *CD19* expression in B cells tended to be higher when bacteria were present (*q *< 0.03, DESeq2), whereas (ii) *NFKBIA* expression in the same cells was lower under the same conditions (*q *< 0.01).

To extend the proximity framework to higher-order interactions, InSituPREP incorporates a triplets analysis that examines how three-cell configurations influence gene expression. This module assesses how the presence of a third cell type affects expression in a given cell-type pair. For each focal cell type (“A”), we identified cells that were either [1] in close proximity to a single other cell type (“B”) or [2] simultaneously in close proximity to two different cell types (“B” and “C”). We then compared the gene expression profiles of type A cells between conditions [1] and [2], pooling data across all samples. Proximal cells were defined as described above. We analyzed only cell types with at least five cells in both conditions [1] and [2] across all samples. Differential gene expression was assessed using the DESeq2 package in R. The resulting cell triplets that influence gene expression are summarized in Supplementary Table S11, with examples shown in Fig. 6A–C.

### Contamination control in bacteria-associated experiments

Fresh biopsies were immediately snap-frozen in liquid nitrogen (−196°C), embedded in OCT compound, and cryosectioned under contamination-controlled conditions. Sections were fixed in 4% paraformaldehyde and stored in 70% ethanol until further processing. Gel preparation, tissue transfer to the gel matrix, and all subsequent steps through imaging were conducted using nuclease-free reagents within a dedicated laboratory space restricted to fixed specimens and free of live biological work, under RNA-sensitive clean-workflow practices, including routine RNase- and DNase-decontamination procedures. These measures were implemented to avoid exogenous bacterial contamination and to minimize the likelihood that detected bacterial signals arose from the experimental process.

### Detection of significant bacterial transcript objects

To characterize host–microbe interactions within the tissue microenvironment, InSituPREP includes a workflow that detects significant bacterial transcript objects and profiles differential gene expression in bacteria-associated cells.

We first developed and applied an ExSeq-based strategy to detect bacteria in breast cancer biopsies by targeting conserved regions of bacterial 16S rRNA. Probe design and construction for 16S rRNA are described in the Methods section “Generation of the dataset”. To establish assay performance, we validated the probes in hydrogels spiked with serial dilutions of *E. coli*, and ExSeq imaging showed that the number of detected 16S rRNA molecules scaled with bacterial concentration (Supplementary Fig. S34). To enable simultaneous profiling of bacterial transcripts and host cell states, a unique barcode was incorporated into the 16S probes, as detailed in the Methods section “Generation of the dataset”.

To detect spatially localized bacterial transcripts, we first extracted the three-dimensional positions of bacterial transcripts in relation to the segmented cells. The three-dimensional positions of bacterial transcripts were clustered using the DBSCAN algorithm [35], across a range of neighborhood radii (ε = 1–150, min_samples = 3). For each field of view (FOV) and ε value, we quantified (i) the number of bacterial transcripts grouped into clusters, (ii) the recall, defined as the fraction of bacterial transcripts included in clusters, and (iii) the number of distinct bacterial objects (clusters).

To assess whether detected clusters represented significant bacterial objects, we performed a permutation-based null test. For each bacterial cluster, the sum of all pairwise Euclidean distances between transcripts was calculated. This statistic was compared to a null distribution generated by repeatedly sampling (10 000 permutations) random sets of transcripts from the most frequent host gene expressed in the same FOV, matched for cluster size. For each cluster, significance for increased spatial compactness relative to the null was assessed using the normal-approximation permutation procedure.

Precision, defined as the fraction of permutation-validated significant clusters among all detected clusters, and recall values were summarized per FOV and across ε values. We further calculated the F1 score (harmonic mean of precision and recall) and identified the ε value that maximized performance, which was consistently ε ≈ 29 pixels (Supplementary Fig. S35A). Bacterial objects were visualized on maximum-projected DAPI images, with transcripts colored by cluster assignment and annotated with their group-specific *P*-values (Fig. 6E). This pipeline provided both a quantitative measure of bacterial clustering and a visual confirmation of bacterial objects in tissue sections.

### Cell classification relative to bacteria

We next assessed which host cells were directly associated with bacterial transcripts. In biopsy 58, where bacterial clusters were detected, we quantified for each cell two metrics: (i) the number of significant bacterial transcripts (i.e. transcripts belonging to a bacterial cluster identified at *p* ≤ 0.05) located within the segmented cell boundary, and (ii) the minimum 3D Euclidean distance between any transcript in the cell and the nearest significant bacterial transcript located outside the cell. Based on these metrics, cells were classified into three categories: (i) ‘Include’ bacteria: cells containing ≥ 1 significant bacterial transcript; (ii) ‘Proximal’ to bacteria: cells without internal bacteria but located ≤ 1 µm from a bacterial transcript; and (iii) ‘Distant’ from bacteria: cells neither containing nor proximal to bacterial transcripts. This classification was then used to stratify cells for downstream differential expression analyses; because no cells were classified as ‘proximal’ in this dataset, analyses focused on the ‘include’ and ‘distant’ groups.

### Differential expression analysis of bacteria-associated cells

To identify host genes differentially expressed in association with bacterial proximity, we compared gene expression in cells that included bacterial transcripts to cells distant from bacteria, analyzing each major cell type separately. For general cell types (e.g. B cells, tumor cells), all specific subtypes were pooled. Binary labels (‘Include’ vs. ‘Distant’) were generated for matched sets of cells, and only these cells were retained for differential expression testing. Statistical significance was evaluated using DESeq2 combined with a permutation-based framework: proximity labels were randomly shuffled across cells (1000 iterations), and the DESeq2 pipeline was rerun for each permuted dataset. For each gene, an empirical permutation-derived *P*-value was calculated. Genes were filtered by log₂ fold change, and both nominal *P*-values (from DESeq2) and permutation-derived *P*-values were corrected for multiple testing. The resulting significant gene sets represent cell-type-specific signatures of bacteria-associated transcriptional responses, capturing effects of direct bacterial inclusion (Fig. 6F, Supplementary Fig. S36 and Supplementary Table S12).

### Applying InSituPREP modules to Xenium and STARmap data

To evaluate the robustness and generalizability of the InSituPREP framework, we applied it to datasets from two additional single-cell spatial transcriptomics technologies: Xenium and STARmap. For Xenium, human breast cancer tissue data were used, with 313 genes interrogated [36]. The dataset contained three samples: two serial sections from the same biopsy and one section from a biopsy of a different patient. Six annotated cell types were analyzed: tumor cells, macrophages, stromal cells, T cells, endothelial cells, and B cells. For STARmap, mouse visual cortex data were used, with 160 genes measured [13]. This dataset includes two samples from mice housed under two distinct physiological conditions: standard light exposure (Light) and 4-day dark adaptation (Dark). Twelve annotated cell types were analyzed: eL4, VIP, Smc, eL5, PV, eL6, Endo, eL2/3, Astro, NPY, Oligo, and SST, corresponding to excitatory and inhibitory neurons, glial cells, and vascular cells. Cell type annotations for both datasets were taken from the original publications.

Effect of intercellular distance on gene expression: The same analysis pipeline used for the MERFISH data was applied to the Xenium and STARmap datasets. The resulting linear dependencies between gene expression and intercellular distance using Xenium data are summarized in Supplementary Tables S13, S14, with examples shown in Supplementary Figs S37–S41. Corresponding results for the STARmap data are summarized in Supplementary Table S18, with examples shown in Supplementary Figs S43–S48.

Effect of the number of neighboring cells on gene expression: The same analysis pipeline described above was applied to the Xenium datasets, using a 40 µm centroid-based distance cutoff to define neighboring cells. The resulting linear dependencies between gene expression and the number of neighboring cells are summarized in Supplementary Tables S13 and S15, with examples shown in Supplementary Fig. S42.

Triplet analysis: The same analysis was applied to the Xenium datasets, using a 10 µm centroid-based distance to define proximal cells. The resulting cell triplets associated with gene expression changes are summarized in Supplementary Tables S16, S17.

## Results

### Multi-patient ExSeq profiling of breast cancer tissues

We applied Expansion Sequencing (ExSeq) to breast cancer biopsies from 10 patients, integrating newly generated and previously published datasets [25] into a unified multi-patient resource (Fig. 1A, Supplementary Fig. S1 and Materials and methods). Eight biopsies originated from metastatic breast cancer, allowing analysis of tumor-microenvironment interactions in advanced disease, which remains poorly characterized [25]. A targeted panel of 299 breast cancer-related genes was sequenced *in situ*, allowing direct measurement of gene expression at nanoscale resolution. The panel was designed based on prior knowledge of cancer biology and is therefore likely enriched for cancer-regulated genes. Matched single-cell RNA-seq (scRNA-seq) data for seven samples and MERFISH data for four samples, all generated from consecutive sections of the same biopsy [25], were incorporated into specific analyses as described below. Segmentation was performed using the InSituSeg [28] algorithm for eight samples and a refined version of InSituSeg for two (Materials and methods and Supplementary Fig. S1C), and outputs from both pipelines were consolidated into unified cell-by-gene count matrices. After quality-control filtering, 3 293 946 sequencing reads were obtained in 19 531 segmented cells across the 10 biopsies (Supplementary Table S1).

To assign cell types, we combined ExSeq profiles with tissue-specific markers derived from the matched scRNA-seq references (Materials and methods). Candidate marker genes were identified using Seurat [30] and complemented with canonical reference markers, with stringent filtering applied to exclude ambiguous cases. Cell clustering was performed using multiple parameter combinations, and a consensus labeling strategy was used to assign robust cell-type identities. Major lineages, including tumor epithelial cells, T cells, B cells, macrophages, fibroblasts, and endothelial cells, were consistently detected across tissues (Fig. 1A, Supplementary Fig. S2 and Supplementary Table S1).

To validate the robustness of this annotation procedure, we permuted raw expression profiles while preserving gene-wise distributions and re-ran the full clustering pipeline. Across 20 permutations per tissue, almost no cells could be assigned valid labels, yielding permutation-based *p* ≈ 0 for all 10 samples. This confirmed the statistical significance of the observed clustering and established confidence in the resulting cell-type annotations (Methods).

With this multi-patient ExSeq dataset established, we next developed a computational workflow to detect spatially associated transcriptional programs (Fig. 1B and Supplementary Fig. S3). This framework, termed InSituPREP, integrates spatial distances, cell-type annotations, and expression measurements to identify transcriptional states that are specifically associated with cell-cell proximity. The methods and results of these analyses are described in subsequent figures.

### Proximity contributes to within-cell type variability

During cell typing, we observed substantial variability among cells within the same cluster, as visualized in low-dimensional embeddings of gene expression (Fig. 2). We hypothesized that part of this variability reflects spatial context, that is, shifts in cell state driven by physical proximity to other cell types. To test this, we stratified cells into proximal (≤1 µm) and distant (>1 µm) groups relative to neighboring cell types. This 1 µm cell-boundary threshold is close to the smallest reliably measurable distance in diffraction-limited spatial transcriptomics methods and approximates potential soma-soma contact (Supplementary Fig. S4), with cell-cell distances calculated in 3D (Supplementary Fig. S5). Dispersion was quantified in principal component analysis (PCA) space (Supplementary Fig. S6). Significance was assessed by permutation testing with 10 000 shuffles while preserving the observed proximal subset size, and multiple comparisons were controlled using the Benjamini–Hochberg procedure (Materials and methods).

Across 10 breast cancer tissues and 232 evaluable cell type-pair combinations, 70 showed statistically significant proximity-associated dispersion (*q* ≤ 0.05) (Supplementary Table S2). For example, 12% of the variability in CD3⁺ T cells was explained by direct contact with tumor cells, 26% of the variability in B cells was attributable to contact with tumor cells, and 23% of the variability in tumor cells was associated with proximity to monocytes (Fig. 2A, B). When validating this analysis on proximity-shuffled data, across all 232 tissue–cell-type combinations, only 3 passed the significance threshold (*q* ≤ 0.05). These findings were robust to potential segmentation artifacts, as removing marker genes of the neighboring cell type did not affect the 70 significant associations (Methods). Results were also consistent across embeddings: 38 significant associations were detected in *t-*SNE space, all of which overlapped with the original 70, whereas none were significant in shuffled controls (0/232; Supplementary Table S2).

### Shared proximity-associated expression programs across datasets and patients

We next asked whether proximity-induced transcriptional programs were reproducible across technologies and consistent across patients with similar clinical features. Using DESeq2 [31], we identified proximity-induced genes for each tumor-non-tumor cell type pair and its reciprocal (Supplementary Table S3, and overlapping genes between biopsies in Supplementary Table S4). Comparing our identified proximity-induced genes (Fig. 2C and Supplementary Table S3) with those reported by Klughammer *et al*. [25] using consecutive sections from overlapping samples revealed that 8 of the 18 genes identified in that study were shared. Klughammer *et al*. [25] reported upregulated genes in tumor cells located near T/NK cells using MERFISH data and a 100 × 100 µm binning approach, so the level of overlap with our single-cell ExSeq analysis is substantial, given the biological, technical, and analytical differences. In tissue 100, tumor cells located near CD3⁺ T cells showed the strongest concordance with Klughammer *et al*., sharing seven upregulated genes: *CD74* and *HLA-B* (MHC class I, B), *KRT18* (a luminal epithelial marker), *ERBB2, HSPB1, TMSB10*, and *XBP1* (Fig. 2C). Several of these genes recurred across additional tissues: *KRT18* in tissue 330, *TMSB10* in tissue 364, and *ERBB2* in tissue 364 (tumor cells adjacent to CD8⁺ T cells). *HLA-E*, another MHC class I-related gene, was also observed in tumor cells close to CD3⁺ T cells in tissues 514 and 982, and in tissue 514, it appeared in both our ExSeq data and the MERFISH dataset from a consecutive section analyzed in [25]. Together, these findings highlight recurrent genes, including *HLA-E, KRT18, TMSB10*, and *ERBB2*, that emerge across distinct tissue-neighbor contexts, suggesting shared transcriptional programs activated in tumor cells adjacent to T cells.

To enable systematic cross-tissue comparisons, *P* values quantifying proximity-related expression for all genes were converted into standardized profiles and projected into PCA space (Supplementary Fig. S7 and Methods). Comparisons between ExSeq and MERFISH datasets generated from consecutive sections of the same biopsies showed that proximity-related expression programs were significantly more similar than expected under permutation (Fig. 2D, Supplementary Fig. S7, Supplementary Table S5). Moreover, patients with similar hormone receptor status (HR⁺/HER2⁻) showed tighter clustering of proximity-induced programs than patients with differing receptor types (Fig. 2D, Supplementary Fig. S7, Supplementary Table S5). This focus is particularly relevant, as the role of tumor-immune interactions in HR⁺/HER2⁻ breast cancer remains less defined than in triple-negative breast cancer or HER2⁺ subtypes [37]. Together, these results demonstrate that a portion of within-cell type variability reflects proximity-induced states, and that these transcriptional programs show concordance across measurement platforms and across patients with similar disease features.

### Proximity to other cell types correlates with both current and future cell states

We performed spatial RNA velocity analysis [26, 27] to examine whether the local tissue microenvironment is associated with transcriptional dynamics (Methods). Leveraging the high resolution of ExSeq, we unambiguously distinguished nuclear and cytoplasmic compartments within each cell, a prerequisite for accurate velocity estimation (Supplementary Fig. S8). Based on transcript abundances from these compartments, we predicted future cell states and projected them into PCA space, enabling quantification of both the magnitude and phase of state changes (Fig. 3).

Across tissues and cell-type pairs, proximity to other cell types significantly correlated with transcriptional trajectories (Fig. 3A–C and Supplementary Fig. S9-S12). Cells in closer proximity to other cell types exhibited larger shifts between current and projected future states, with the correlation between intercellular distance and these shifts consistently exceeding chance expectations (*q* < 0.05, permutation test, Methods). In total, 19 cell-type combinations showed significant correlations between intercellular distance and the magnitude of predicted future state change, and 27 with the magnitude of the current state (Supplementary Fig. S9).

For example, CD8⁺ T cells adjacent to tumor cells showed pronounced state changes compared to distant cells, as visualized by quiver plots and phase analyses (Fig. 3D). Similar proximity-dependent effects were observed for CD3⁺ T cells and B cells relative to tumor cells (Fig. 3E–F).

At the gene level, RNA velocity analysis identified transcripts whose velocities were sensitive to intercellular distance, with several genes showing significant velocity-distance correlations that may contribute to the observed cell-state differences (Fig. 3C, Supplementary Fig. S12, and Methods). Together, these results demonstrate that proximity between different cell types is significantly associated with both current and future cell states, highlighting the importance of spatial context in transcriptional dynamics.

### Gene expression levels exhibit linear dependencies on physical distance to neighboring cell types

We next tested whether transcriptional states in tumor and non-tumor cell types varied as a function of their physical proximity to one another. Using ExSeq data, we quantified the minimum distances between tumor cells and their nearest non-tumor neighbors, and modeled the relationship between gene expression in tumor or non-tumor cells and these distances by linear regression (Methods). Stringent filtering ensured that only robust associations were retained, including removal of lowly expressed genes, false discovery rate (FDR) correction across all gene–cell-type–tissue combinations, and confirmation of fit quality with Gaussian smoothing. A composite scoring scheme further prioritized associations with strong and interpretable spatial trends (Methods).

Several examples highlight the diversity of proximity-dependent gene-distance relationships (Fig. 4, Supplementary Fig. S13-S29, and Supplementary Table S6). *CSTB*, a ubiquitous cysteine-protease inhibitor that protects cells from protease leakage and oxidative stress, decreased linearly with distance in B cells, such that those closest to tumor cells showed the highest *CSTB* expression (Fig. 4A, B and Supplementary Fig. S13-S15). Similarly, *MYLK*, a key regulator of actin-myosin contractility in fibroblasts, showed elevated expression in fibroblasts located near tumor cells (Supplementary Fig. S16-S17). *CTSL* (*Cathepsin L*) expression in tumor cells was elevated when near CD3⁺ T cells (Supplementary Fig. S18-S19), again demonstrating proximity-dependent upregulation. *CTSL* expression was reported by Klughammer *et al*. [25] to be elevated in a subpopulation of tumor cells within metastatic breast cancer tissues, yet its association with proximity to T cells has not been examined.

Notably, *ERBB2 (HER2)*, which Klughammer *et al*. [25] found to be elevated in tumor cells adjacent to T cells, displayed the same trend in our data, with higher expression in tumor cells near CD8⁺ T cells (Fig. 4C, D, Supplementary Fig. S20-S21). Importantly, *ERBB2* overexpression has been associated with more aggressive tumor phenotypes and poorer prognosis in breast cancer [38]. Similarly, *GATA3*, previously identified by Klughammer *et al*. [25] as highly expressed in tumor cells distant from T cells in tissue 982 using MERFISH, likewise increased with greater distance from CD8⁺ T cells in our ExSeq analysis of the same tissue (Fig. 4E–F and Supplementary Fig. S22-S23). GATA3 is a key luminal-lineage transcription factor that reinforces epithelial identity and suppresses epithelial-mesenchymal transition, and recent studies suggest that its expression in breast cancer may be indicative of tumor response to immunotherapy [39].

Overall, we identified 34 genes whose expression was linearly dependent on distance to another cell type (Supplementary Table S6 and Supplementary Figs S24, S25). Analysis of the MERFISH datasets from four samples revealed 3 or 23 genes (depending on the stringent cutoff applied; see Methods) with distance-dependent expression, including one overlapping case: *FOS*, an AP-1 transcription factor involved in regulating cell growth and differentiation, exhibited higher expression in endothelial cells located farther from tumor cells in our ExSeq analysis (Fig. 4G–H and Supplementary Figs S26, S27). This spatial trend was consistent with the pattern observed in the MERFISH dataset (Fig. 4I–J, Supplementary Figs S28, S29, and Supplementary Tables S7, S8).

Together, these results demonstrate that linear dependencies between intercellular distance and gene expression can be detected across multiple cell types, and that the directionality of these relationships varies across genes. Importantly, we observed similar linear trends in the MERFISH datasets, providing orthogonal support for our approach. This analysis highlights how the transcriptional state of a given cell type can be systematically associated with its spatial relationship to neighboring cells in the tumor microenvironment.

### Local cellular density modulates gene expression

We next asked whether the influence of neighboring cells is cumulative, that is, whether tumor or non-tumor cells exhibit greater transcriptional changes when surrounded by larger numbers of cells from another type. Using ExSeq profiles from the 10 breast cancer tissues, we quantified for each cell the number of neighboring cells of a different type within a 15 µm cell-boundary distance cutoff and modeled gene expression as a function of neighbor count (Methods). To mitigate data imbalance, which arises because most cells have relatively few neighbors, we employed an iterative sampling approach in which balanced datasets were generated across neighbor-count bins and regression slopes averaged over 1000 iterations. This method identified 34 significant neighbor-associated genes (*q* < 0.05) across 10 distinct cell-type pairs (Supplementary Table S9). Roughly half of these associations were positive (47.1%, 16/34), indicating increasing expression with higher neighbor counts, and half were negative (52.9%, 18/34), indicating suppression with crowding. Results were robust to alternative distance cutoffs (10 and 20 µm) and alternative regression schemes (weighted least squares, slope-aggregation methods), and later filtered to a consensus set of 15 genes supported across approaches (Supplementary Table S9).

Examples of both activation and suppression are shown in Fig. 5 (and Supplementary Figs S30, S31). *ICOS* expression in CD3⁺ T cells decreased with increasing numbers of neighboring tumor cells (Fig. 5A, B). In contrast, *PHGDH* expression in B cells increased with the number of neighboring tumor cells (Fig. 5C, D and Supplementary Fig. S30). A second suppressive example was *TIMP1* in CD3⁺ T cells, which showed reduced expression in cells surrounded by many tumor cells (Fig. 5E, F and Supplementary Fig. S31). These findings support the idea that local cellular density systematically modulates gene expression. Moreover, the identified genes are biologically interpretable: *ICOS* is a co-stimulatory receptor important for T-cell activation [40], *PHGDH* encodes a metabolic enzyme known to be upregulated in activated B cells [41], and *TIMP1* encodes a matrix regulator that also signals through CD74 to modulate T-cell function [42, 43].

We next extended the neighbor analysis to cases where the primary and neighboring cells were of the same type. Across all tissues, this analysis revealed 48 significant associations spanning eight cell types, including 32 genes supported across approaches (Supplementary Table S10). Interestingly, tumor cells showed a clear intra-type dependency (Supplementary Fig. S32, S33). For example, *FTL* (ferritin light chain), an iron-storage protein whose overexpression has been reported to be associated with breast cancer progression [44], showed increased expression with the number of neighboring tumor cells (Fig. 5G, H and Supplementary Fig. S32). These findings demonstrate that cell density, whether from same-type or different-type neighbors, is associated with the transcriptional states of cells within the tumor microenvironment.

### Triplet cell interactions

Beyond pairwise proximities, we examined whether the joint presence of two different neighboring cell types influenced transcriptional states in a focal cell. For each triplet configuration, cells of type A were compared between contexts where they neighbored only cells of type B versus cells of both type B and type C (≤1 µm cell boundaries distance; Methods). DESeq2 [31] analysis identified genes whose expression differed significantly between these conditions after multiple testing correction. Overall, 25 genes were detected in 7 cell triplets (Supplementary Table S11).

Representative examples are shown in Fig. 6A–C. Among these, *ERBB2* (*HER2*) showed higher expression in tumor cells surrounded by two endothelial neighbors compared with those adjacent to one endothelial and one smooth muscle cell. *CTSL*, a lysosomal cysteine protease, showed reduced expression in endothelial cells adjacent to tumor cells compared with those neighboring both tumor cells and T cells. Similarly, *PTPRC (CD45)*, a protein tyrosine phosphatase, showed lower expression in macrophages surrounded only by tumor cells compared with macrophages adjacent to both tumor cells and T cells. These results indicate that higher-order neighborhood configurations, not just pairwise proximities, can modulate gene expression programs.

### Bacterial presence correlates with local cell states

In addition to host-host interactions, we developed and applied an ExSeq-based strategy to detect bacteria in breast cancer biopsies (Fig. 6D, Supplementary Figs S34–S36 and Materials and methods). Biopsies 58 and 59 were selected for bacterial richness, which was independently confirmed by bulk RNA sequencing of 16S rRNA [29]. We constructed and validated probes targeting conserved regions of bacterial 16S rRNA, enabling *in situ* detection of diverse bacterial strains. Validation experiments in hydrogels spiked with serial dilutions of *E. coli* confirmed that the number of detected 16S rRNA molecules correlated with bacterial concentration (Supplementary Fig. S34). To enable parallel profiling of bacteria and host cell states, we incorporated a unique barcode into the 16S probes, allowing bacterial rRNA to be distinguished from mRNA during the ExSeq workflow. This approach allowed precise spatial localization of bacterial clusters in tumor tissue while simultaneously quantifying gene expression in adjacent human cells. Permutation testing identified statistically significant clusters of 16S rRNA reads within tumor sections, confirming the presence of bacteria (Fig. 6E, Supplementary Fig. S35, and Materials and methods). In total, we detected 37 significant bacterial clusters in biopsy 58, whereas no significant clusters were found in biopsy 59, suggesting that detection sensitivity may vary across tissues.

Because 16S profiling was performed in parallel with host mRNA sequencing, we could directly correlate bacterial presence with transcriptional states of neighboring cells. Overall, we identified 59 genes with significantly altered expression in association with bacterial clusters, spanning four cell types (Supplementary Fig. S36 and Supplementary Table S12). Notably, *CD19*, which is known as enhanced in B cells, tended to be higher in B cells located within bacteria-rich regions. An opposite trend was observed for *NFKBIA*, a prognostic marker in breast cancer whose higher expression is linked to better outcomes according to the Human Protein Atlas, suggesting that microbial cues may be associated with differences in immune states in the tumor microenvironment (Fig. 6F).

Together, these results demonstrate that both higher-order cellular neighborhoods and local bacterial presence contribute to variation in cell states, adding new layers of complexity to the tumor ecosystem that can be resolved with single-cell spatial transcriptomics.

### Application of InSituPREP to Xenium and STARmap datasets

To evaluate the robustness and generalizability of InSituPREP, we applied the framework to spatial transcriptomics datasets generated using Xenium [36] and STARmap [13] technologies (Methods). The Xenium dataset consisted of human breast cancer tissue with 313 targeted genes and three samples: two consecutive sections from the same biopsy and one section from a biopsy of a different patient, spanning six annotated cell types. The STARmap dataset consisted of the mouse primary visual cortex with 160 measured genes across two physiological conditions (light and dark adaptation) and included 12 annotated cell types. In the Xenium data, multiple gene-cell type associations were detected in which gene expression depended on intercellular distance, the number of neighboring cells, and other spatial relationships captured by InSituPREP (Supplementary Figs S37–S42 and Supplementary Tables S13–S17). Comparable numbers of associations were detected in the two consecutive sections from the same biopsy, and the overlap between these sections was up to several-fold higher than the overlap observed with the biopsy from a different patient, indicating reproducible spatial patterns within the same tissue (Supplementary Table S13).

Representative examples of proximity-dependent expression include increased *SCD* expression in tumor cells with increasing distance from T cells and decreased *APOC1* expression in macrophages with increasing distance from tumor cells, with consistent spatial patterns observed across the two consecutive sections (Supplementary Fig. S37). Neighbor-dependent relationships were also detected, including a negative association between stromal cell *ADH1B* expression and the number of neighboring tumor cells and a positive association between endothelial cell *CENPF* expression and tumor cell density, again showing consistent spatial patterns between the two serial sections (Supplementary Fig. S42). Application of the Proximity module to the STARmap dataset identified proximity-associated gene expression relationships in mouse visual cortex (Supplementary Figs S43–S48 and Supplementary Tables S18, S19), including decreased *Egr1* and *Nrn1* expression in L2/3 neurons with increasing distance from parvalbumin (PV) interneurons, highlighting proximity-associated transcriptional patterns between excitatory and inhibitory neuronal populations (Supplementary Fig. S43). Together, these analyses demonstrate that InSituPREP identifies spatially associated gene expression patterns across multiple tissues and spatial transcriptomics platforms.

## Discussion

In this study, we developed InSituPREP (*In Situ Proximity Expression Program*), a computational framework that integrates multiple analytical modules to quantify how spatial context influences single-cell transcriptional variability in three-dimensional tissues. Applied to super-resolved ExSeq data from 10 breast cancer biopsies, the framework demonstrates how single-cell spatial transcriptomics can uncover proximity-associated gene programs across cellular scales.

Unlike previous spatial analysis approaches that rely on planar (2D) proximity definitions, InSituPREP performs spatial analyses in 3D. However, only contributions from cells captured within the imaging volume can be quantified. Therefore, when thicker tissue volumes are imaged, the completeness of the spatial context improves. For highly non-compact cells such as neurons with extended processes, proximity inference based on cell-boundary distance may be less precise when RNA coverage in fine projections is sparse, although soma-level proximity between cells can still be detected.

In addition, whereas many previous approaches focus on binary (proximal or distant) distance classification between cells, InSituPREP models continuous and higher-order spatial relationships at single-cell resolution through distance-dependent regression, neighborhood-density modeling, and triplet interaction analyses. This enables the detection of gradual and subtle proximity effects that are missed by discrete or low-resolution frameworks. Incorporating spatial RNA velocity extends the analysis to dynamic trajectories, showing that spatial context is associated with both current and predicted future transcriptional states. Moreover, because most single-cell spatial transcriptomics technologies rely on targeted gene panels with limited gene coverage, InSituPREP identifies genes associated with spatial context without requiring that they form predefined co-expression programs.

Applied to breast cancer tissues, InSituPREP revealed multiple spatial associations with gene expression. Pairwise tumor-immune and tumor-stromal interactions accounted for substantial fractions of within-cell type variability, while triplet configurations indicated that combinations of neighboring cell types are associated with distinct transcriptional programs. Continuous models uncovered linear dependencies between intercellular distance and expression of specific genes, illustrating that proximity effects can manifest in graded rather than strictly binary patterns. Local cell density, whether involving tumor-immune, tumor-stromal, or same-type neighbors, was associated with changes in expression in a manner consistent with tissue crowding effects. Spatial RNA velocity analysis further showed that proximity to particular neighbors, especially in tumor and T cells, was associated with larger predicted transcriptional transitions.

Although initially developed and benchmarked on ExSeq data, InSituPREP was applied here across four single-cell spatial transcriptomics technologies: ExSeq, MERFISH, Xenium, and STARmap. These platforms rely on distinct experimental implementations of spatially resolved transcript detection, including amplification-based *in situ* readouts (ExSeq and Xenium) and combinatorial barcoding strategies (MERFISH and STARmap). Despite these methodological differences, the same analytical framework identified spatially associated transcriptional patterns across all datasets analyzed. In addition, extending ExSeq with barcoded 16S rRNA probes enabled joint detection of microbial and host transcripts, and InSituPREP captured corresponding host-microbiome transcriptional associations *in situ*, illustrating its adaptability to additional data modalities.

Together, these analyses illustrate how modeling spatial context at single-cell resolution can reveal distinct spatial associations with cell states, including effects related to proximity, local density, and microbial presence. Applying InSituPREP to larger patient cohorts, additional tumor types, and broader gene panels may enable identification of conserved and patient-specific spatial programs with relevance for tumor biology and therapeutic response.

## Supplementary Material

gkag406_Supplemental_File

## Data Availability

The InSituPREP Python package is publicly available on GitHub (https://github.com/Tal-Goldberg/InSituPREP), including complete documentation and installation instructions, and is linked to the corresponding Zenodo archive (https://doi.org/10.5281/zenodo.17450066). The Zenodo deposit also contains the spatial sequencing data generated in this study, together with test datasets for running the code and the corresponding example outputs.
