# Supplementary material for: InSituPREP enables 3D single-cell mapping of interaction-associated gene programs in the breast cancer tumor microenvironment
Source: Nucleic Acids Res. 2026 May 11;54(9):gkag406. doi: 10.1093/nar/gkag406 (PMC13158666; doi:10.1093/nar/gkag406)
Supplement: gkag406_Supplemental_File [file gkag406_supplemental_file.pdf]

Supplementary Data

InSituPREP enables 3D single-cell mapping of interaction-associated gene programs in the breast cancer tumor microenvironment

Tal Goldberg[1,2,3]*, Michal Danino-Levi[1,2,3]*, Modi Safra[1,2,3], Yedaaya Copeland[1], Efrat Roth Weizman[1,2,3], Noa Konforti[1,2,3], Tal Ishon[1], Noga Ben-Ari[1], Yael Brand[1,2,3], Bareket Kruger[1], Gili Perry[4], Dana Morzaev-Sulzbach[4], Einav Nili Gal-Yam[5], Maya Dadiani[4] †, Shahar Alon[1,2,3] †

[1]The Alexander Kofkin Faculty of Engineering, [2]Institute for Nanotechnology and Advanced Materials (BINA), [3]The Gonda Multidisciplinary Brain Research Center, Bar-Ilan University, Israel. [4]Cancer Research Center, [5]Institute of Breast Oncology, Jusidman Cancer Center, Sheba Medical Center, Ramat Gan, Israel.
*Equal contribution.
† Corresponding: **maya.dadiani@sheba.health.gov.il**; **shahar.alon@biu.ac.il**

This PDF file includes:

Supplementary Information

Figs. S1 to S48

Tables S1 to S19

References

Supplementary Information

In the context of the Methods section entitled *“Dispersion in cell type explained by proximity-induced cell state”*, we performed two additional analyses to further validate the robustness of our conclusions: (1) a sensitivity analysis addressing potential segmentation artifacts, and (2) a robustness assessment using an alternative low-dimensional embedding (*t*-SNE).

(1) Sensitivity to potential segmentation artifacts.
For each tissue and cell-type pair (type *i* = primary; type *ii* = neighbor), we mitigated potential misassignment of transcripts from type *ii* to adjacent type *i* cells by filtering the type *i* expression matrix to exclude all genes designated as markers of the corresponding type *ii* cells (marker definition as in Methods section “Cell type clustering of ExSeq data”). Using the filtered profiles, we reran the full proximity-state pipeline exactly as for the original data. Significance was reassessed for all tissue–cell-type–pair combinations using 10,000 permutations per pair ($q \leq 0.05$). In this analysis, the same 70 pairs previously identified as significant remained significant, confirming that results were unaffected by potential segmentation artifacts (Table S2).

(2) Robustness to embedding choice (*t*-SNE analysis).
To assess the robustness of proximity-induced cell state signals to the choice of low-dimensional embedding, we used 2D *t*-SNE instead of PCA. Expression profiles of type *i* cells were standardized by z-scoring, and a *t*-SNE embedding was computed. Using the resulting coordinates, we repeated the full analysis as described above. Statistical significance was assessed by permuting proximal–distant labels 10,000 times within each tissue-cell-type pair while preserving the observed proximal group size ($q \leq 0.05$). Overall, 38 of the 232 examined tissue–cell-type combinations remained significant, all of which were contained within the 70 combinations identified in the PCA-based analysis of the same data, indicating that proximity-associated signals were largely robust to the choice of low-dimensional embedding. Applying the same *t*-SNE-based procedure to proximity-shuffled data yielded 0 significant combinations ($q \leq 0.05$), confirming that the observed signals were not attributable to random chance (Table S2).

Supplementary Figures

A

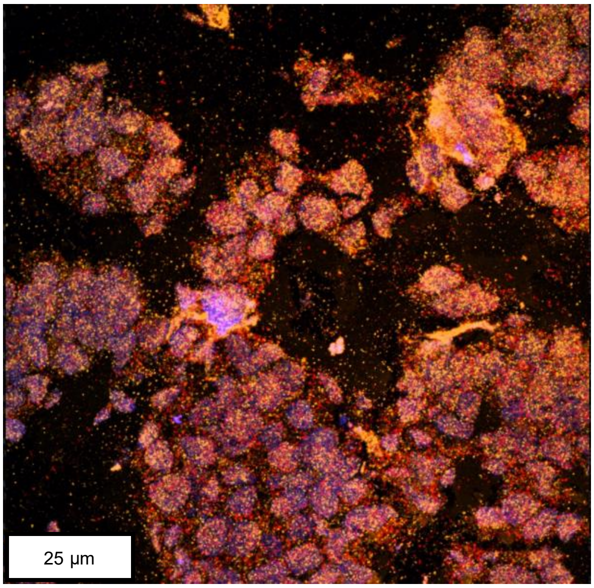

B

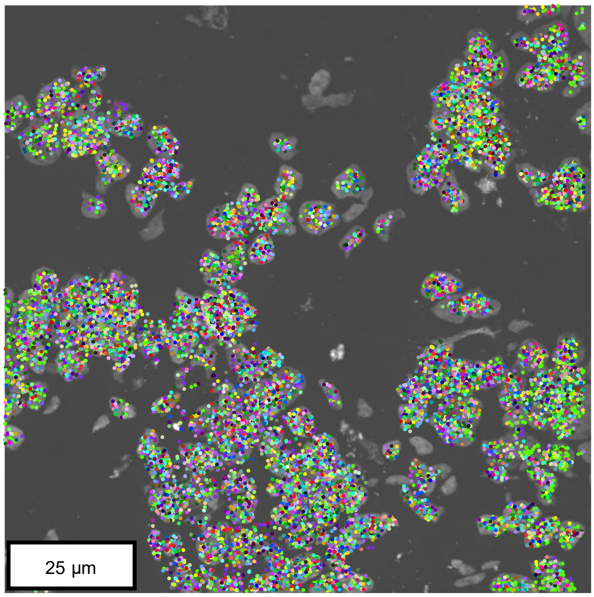

C

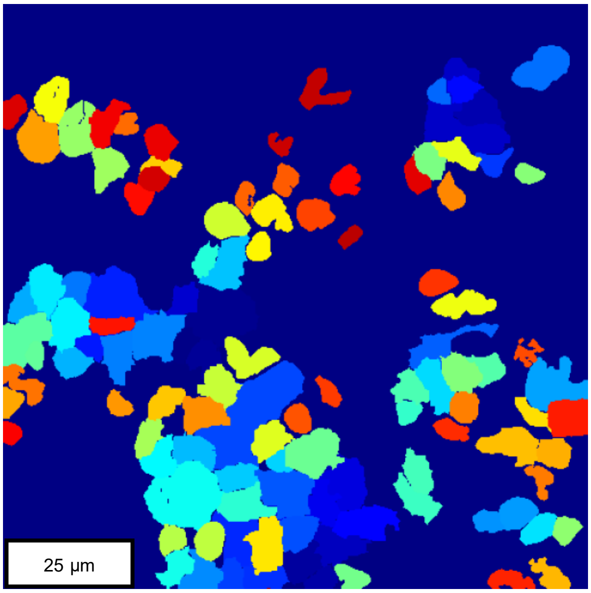

Fig. S1. ExSeq imaging, base calling, and segmentation in a representative field of view of breast cancer biopsy 59. **(A)** ExSeq raw data image showing DAPI-stained nuclei and the first round of sequenced bases in a single 40× field of view (FOV) within the tissue. Shown is a 2D maximum-intensity projection of the 3D dataset. **(B)** Detected transcripts in the same FOV after base calling and barcode alignment. Each colored spot represents a transcript corresponding to one of the 299 sequenced genes. **(C)** Segmentation results for the same FOV obtained using the *InSituSeg* tool(1).

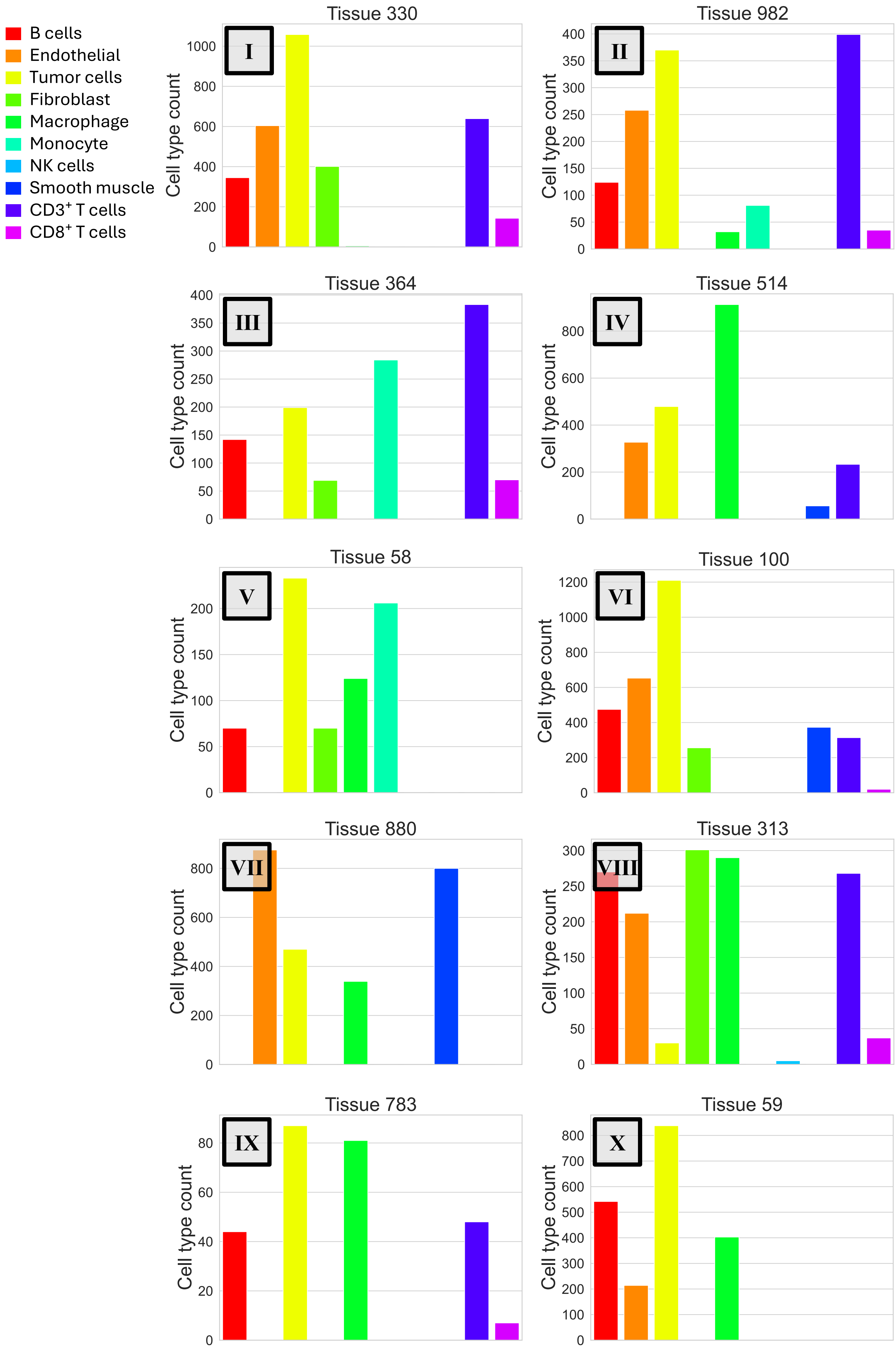

B cells
Endothelial
Tumor cells
Fibroblast
Macrophage
Monocyte
NK cells
Smooth muscle
CD3+ T cells
CD8+ T cells
Tissue 330
I
Tissue 982
II
Tissue 364
III
Tissue 514
IV
Tissue 58
V
Tissue 100
VI
Tissue 880
VII
Tissue 313
VIII
Tissue 783
IX
Tissue 59
X
Cell type count

Fig. S2. Cell type composition across tissues. Bar plots show the total number of classified cells per cell type for each of the ten analyzed tissues. Colors correspond to distinct cell types, as indicated in the legend (top left). Each panel (I–X) represents a single tissue sample.

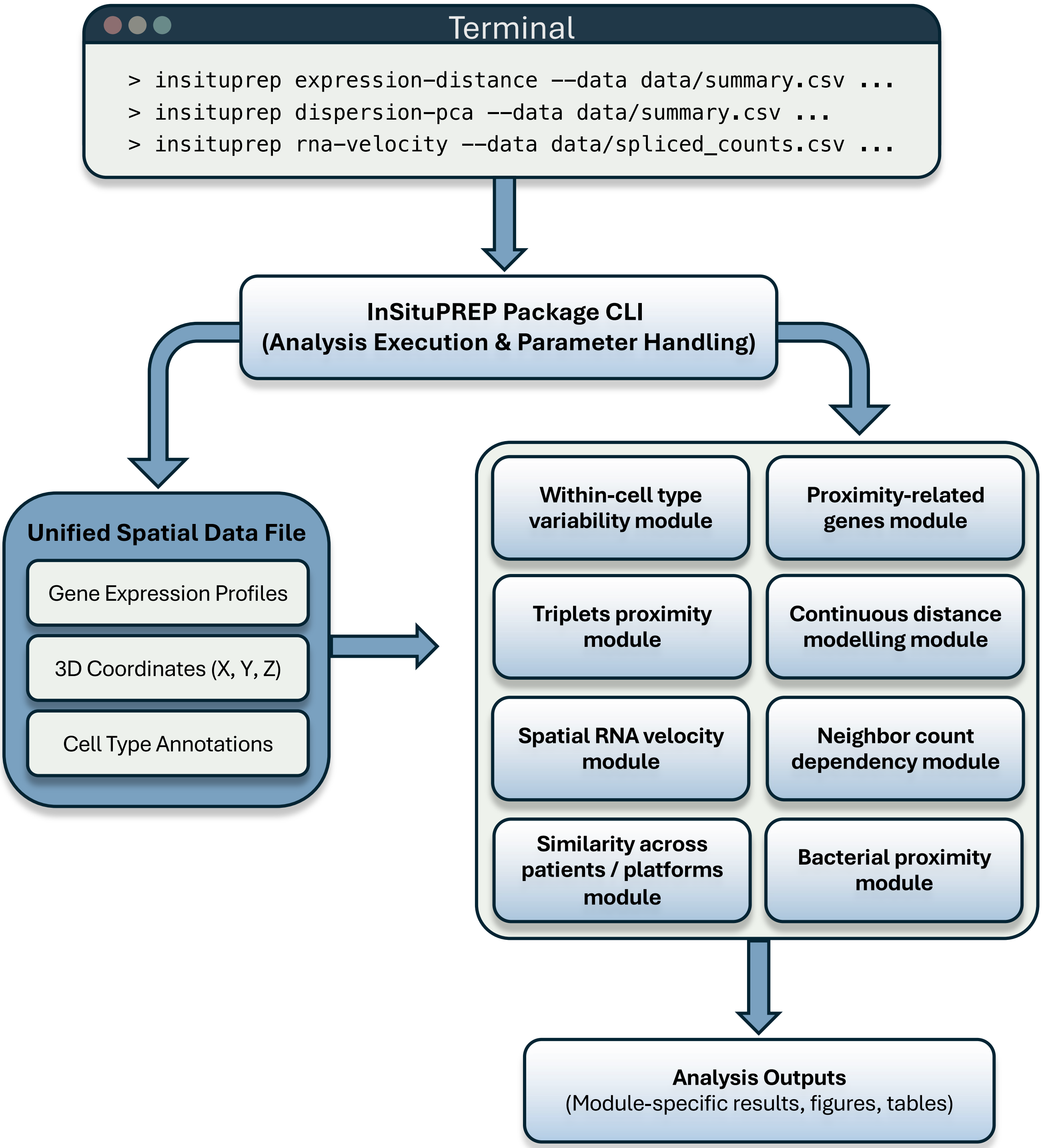
Terminal
> insituprep expression-distance --data data/summary.csv ...
> insituprep dispersion-pca --data data/summary.csv ...
> insituprep rna-velocity --data data/spliced_counts.csv ...
InSituPREP Package CLI
(Analysis Execution & Parameter Handling)
Unified Spatial Data File
Gene Expression Profiles
3D Coordinates (X, Y, Z)
Cell Type Annotations
Within-cell type variability module
Proximity-related genes module
Triplets proximity module
Continuous distance modelling module
Spatial RNA velocity module
Neighbor count dependency module
Similarity across patients / platforms module
Bacterial proximity module
Analysis Outputs
(Module-specific results, figures, tables)

Fig. S3. Architecture of the InSituPREP Python package for single-cell spatial analysis. Schematic overview of the InSituPREP software architecture. The framework is operated through a command-line interface (CLI), where each spatial analysis is executed as a dedicated command (e.g., expression-distance, dispersion-PCA, RNA velocity, triplet proximity, neighbor-count regression, bacterial proximity). The package operates on single-cell spatial transcriptomics datasets provided in a compatible per-cell format. For each command, users supply the required input files and parameters, which may include per-cell spatial summary tables (gene expression, spatial coordinates in 2D or 3D, and cell-type annotations), gene lists, distance matrices, transcript-level tables, and other module-specific inputs. The CLI layer performs argument parsing, parameter validation, and dataset filtering before passing the validated inputs to the corresponding analysis pipeline, where spatial effects on gene expression are quantified using module-specific statistical models.

The framework includes: Continuous distance modeling (regression of gene expression as a function of Euclidean distance to neighboring cell types); Dispersion in PCA space (contribution of cell-cell proximity to within-cell type variability). Triplet proximity analysis (identification of higher-order spatial cell configurations); Neighbor-count dependency analysis (regression of gene expression as a function of the number of neighboring cells within a defined spatial radius); Spatial RNA velocity analysis (multi-stage modeling integrating velocity-derived spatial features and their correlation to physical proximity between cells); Proximity-based differential expression (identification of genes enriched in spatially proximal versus distal cell subsets); Cross-sample similarity analysis (comparison of spatial gene programs across tissues or platforms); Bacterial proximity module (spatial clustering of target transcripts and proximity-dependent downstream gene expression analysis).

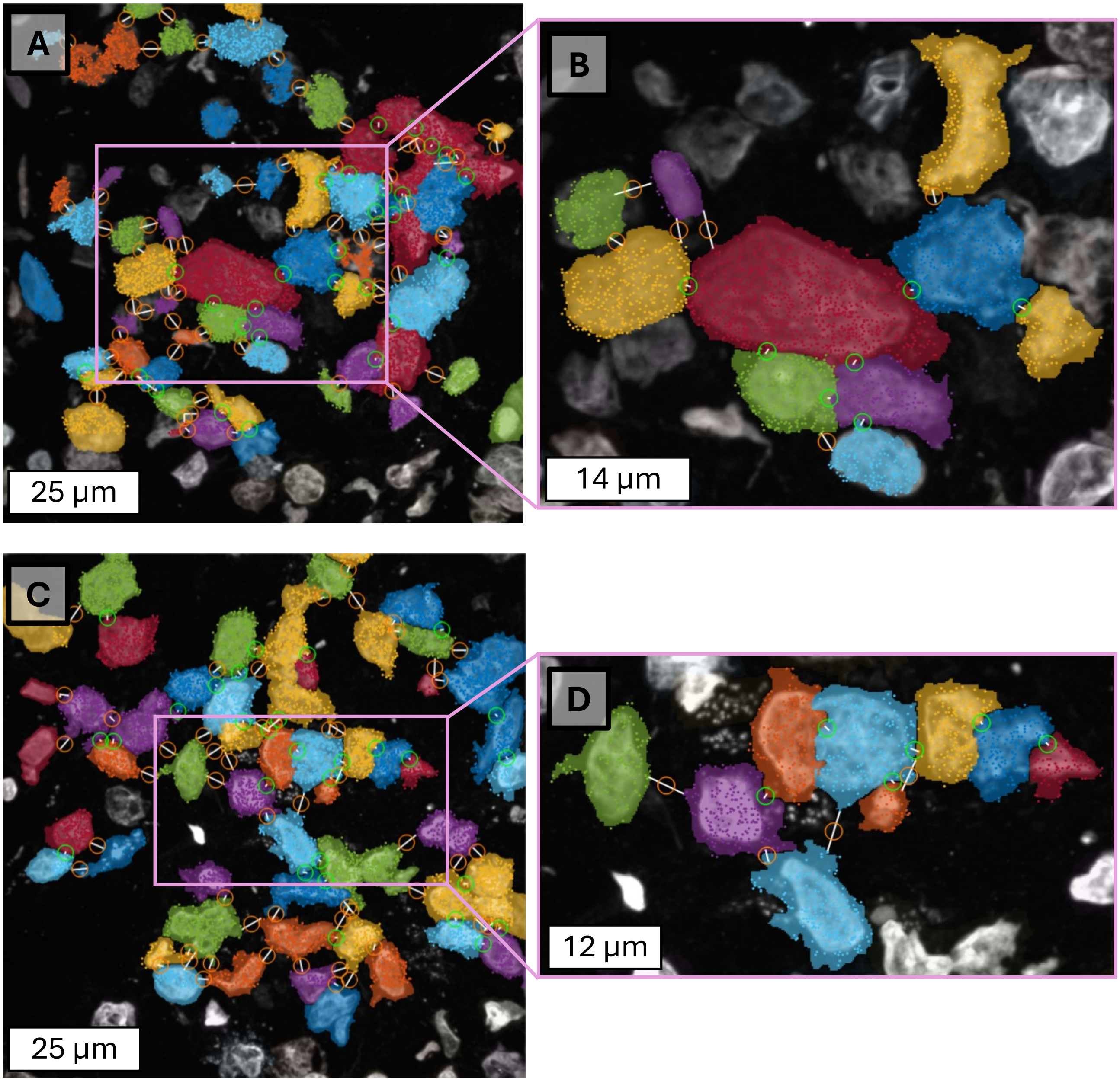
A
B
25 µm
14 µm
C
D
25 µm
12 µm

Fig. S4. Potential soma-soma contact with ≤1 μm cell-boundary distance. Two fields of view selected at random from a randomly chosen breast cancer biopsy (sample ID 313) are shown. The sample was analyzed using ExSeq. **(A, C)** Overlay of DAPI nuclear staining (grey) and cell segmentation masks (colored regions). Colored dots represent individual RNA transcripts assigned to specific cells. Solid white lines connect the pair of transcripts with the minimal 3D Euclidean distance between adjacent cells. The minimal-distance pairs are highlighted with circles: green circles indicate ≤1 μm cell-boundary distance, whereas orange circles indicate >1 μm cell-boundary distance. **(B, D)** Higher magnification views corresponding to the regions marked by pink rectangles in (A) and (C), respectively. In the examples shown, cells with ≤1 μm cell-boundary distance exhibit apparent soma-soma contact.

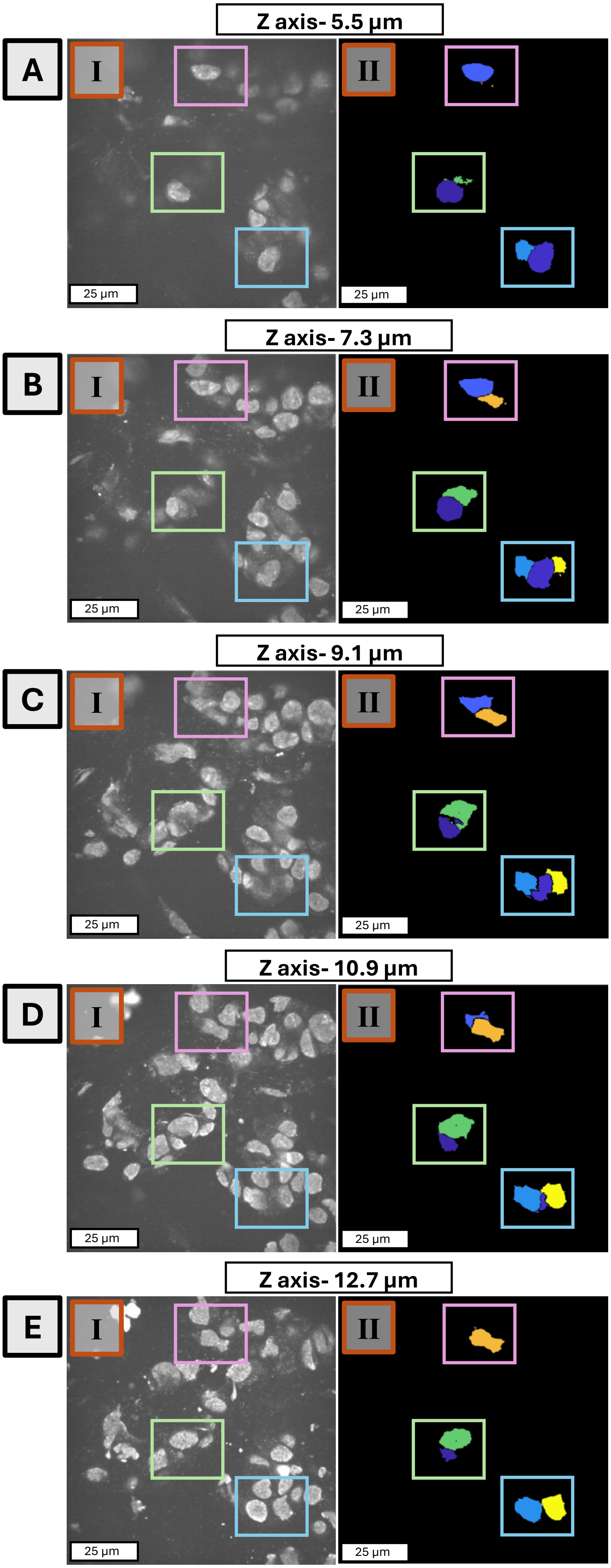

Z axis- 5.5 µm
A
I
II
25 µm
Z axis- 7.3 µm
B
Z axis- 9.1 µm
C
Z axis- 10.9 µm
D
Z axis- 12.7 µm
E

Fig. S5. Detection of proximal cells across z-planes using 3D distance calculations. Shown is one field of view from a breast cancer biopsy (sample ID 880) analyzed using ExSeq. **(A-E)** Selected z-planes spanning the imaging volume. (I) DAPI nuclear staining. Colored boxes highlight example proximal cell pairs and triplets. (II) Corresponding segmented cells within the boxed regions in (I). Cells that appear separated in individual imaging planes can nonetheless be identified as proximal because InSituPREP calculates cell-cell distances in 3D across the full imaging volume.

Fig. S6 – part 1

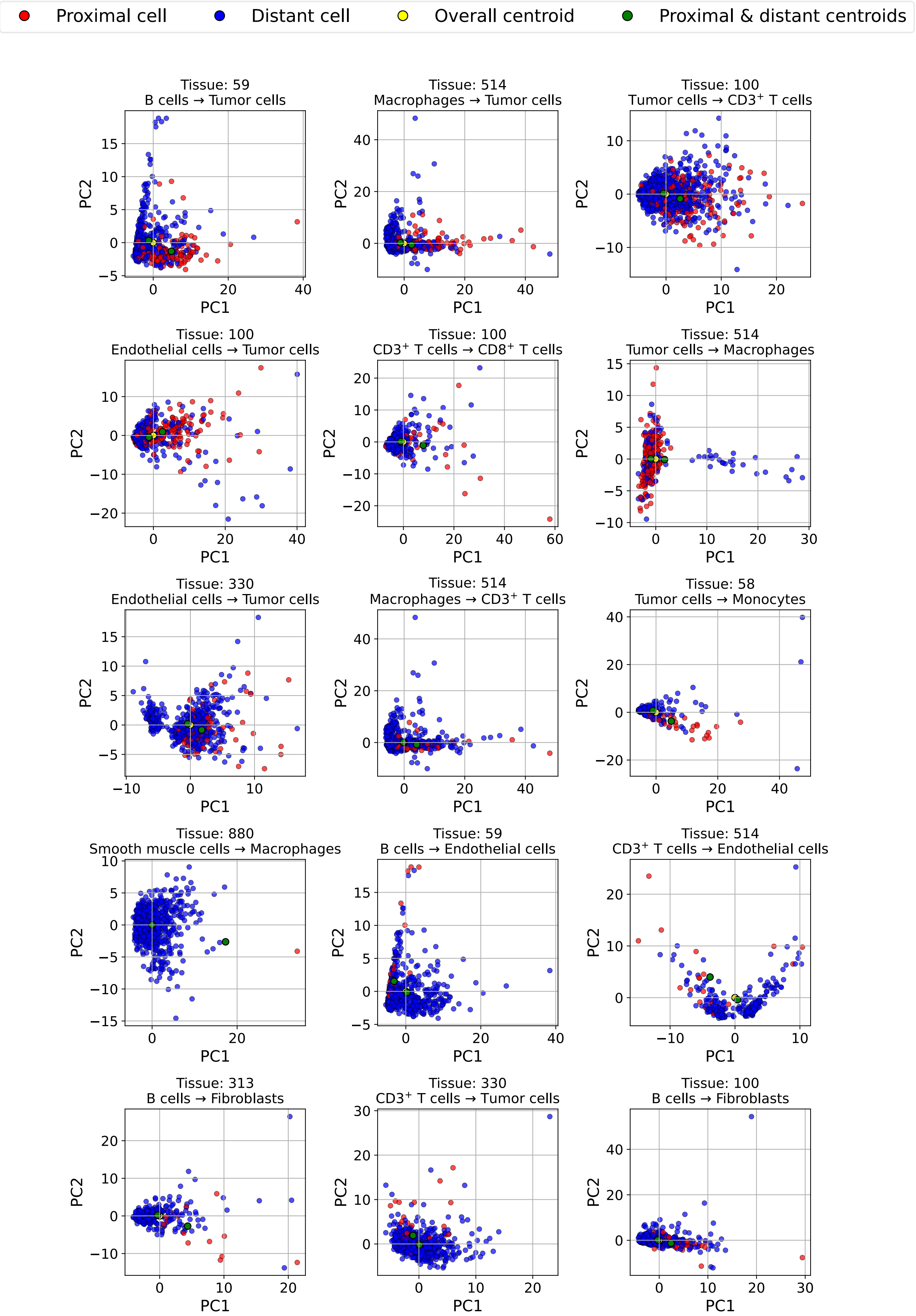
Proximal cell
Distant cell
Overall centroid
Proximal & distant centroids
Tissue: 59
B cells → Tumor cells
Tissue: 514
Macrophages → Tumor cells
Tissue: 100
Tumor cells → $CD3^+$ T cells
Tissue: 100
Endothelial cells → Tumor cells
Tissue: 100
$CD3^+$ T cells → $CD8^+$ T cells
Tissue: 514
Tumor cells → Macrophages
Tissue: 330
Endothelial cells → Tumor cells
Tissue: 514
Macrophages → $CD3^+$ T cells
Tissue: 58
Tumor cells → Monocytes
Tissue: 880
Smooth muscle cells → Macrophages
Tissue: 59
B cells → Endothelial cells
Tissue: 514
$CD3^+$ T cells → Endothelial cells
Tissue: 313
B cells → Fibroblasts
Tissue: 330
$CD3^+$ T cells → Tumor cells
Tissue: 100
B cells → Fibroblasts
PC1
PC2

Fig. S6 – part 2

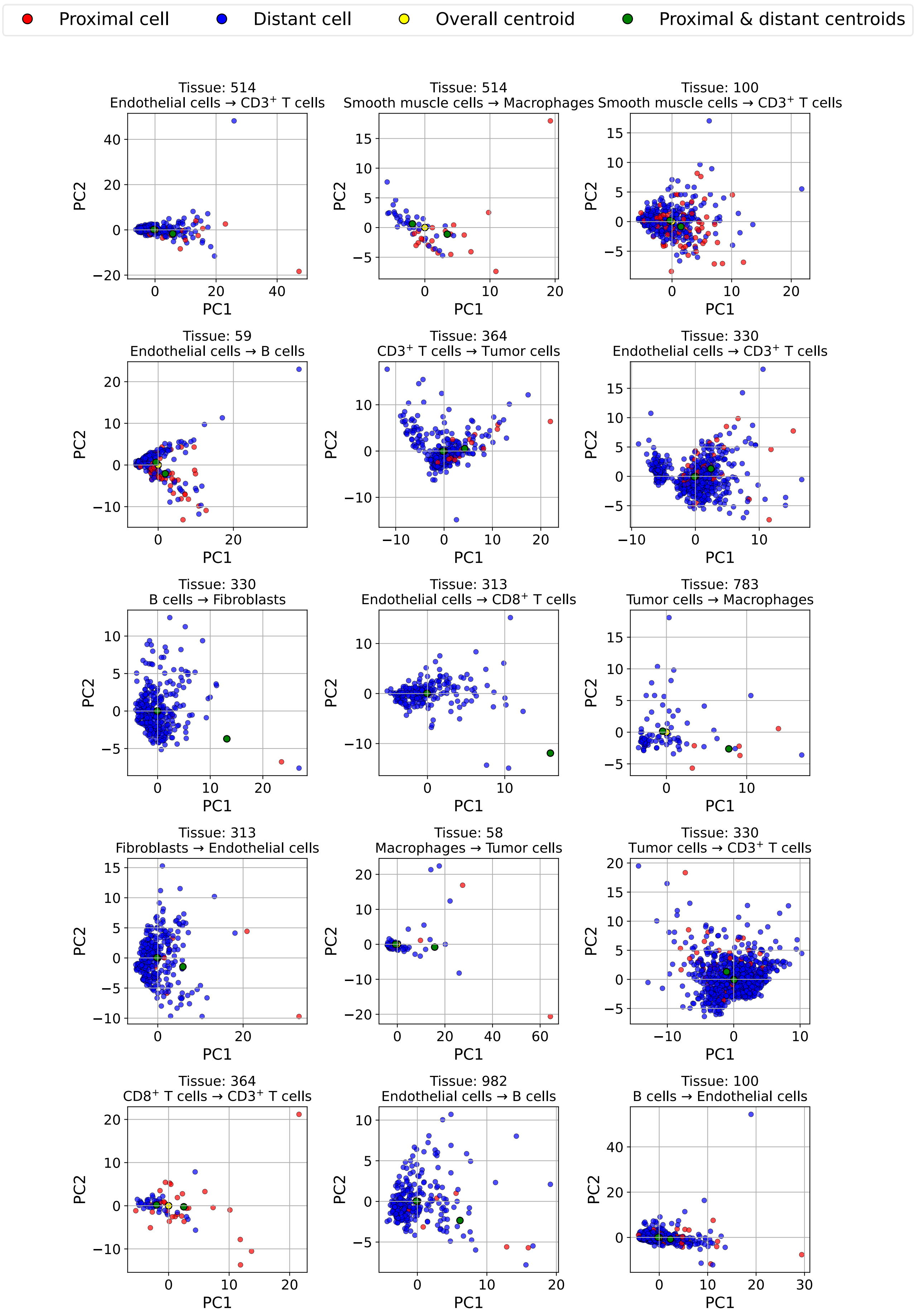

Proximal cell
Distant cell
Overall centroid
Proximal & distant centroids
Tissue: 514
Endothelial cells → CD3$^+$ T cells
Tissue: 514
Smooth muscle cells → Macrophages
Tissue: 100
Smooth muscle cells → CD3$^+$ T cells
Tissue: 59
Endothelial cells → B cells
Tissue: 364
CD3$^+$ T cells → Tumor cells
Tissue: 330
Endothelial cells → CD3$^+$ T cells
Tissue: 330
B cells → Fibroblasts
Tissue: 313
Endothelial cells → CD8$^+$ T cells
Tissue: 783
Tumor cells → Macrophages
Tissue: 313
Fibroblasts → Endothelial cells
Tissue: 58
Macrophages → Tumor cells
Tissue: 330
Tumor cells → CD3$^+$ T cells
Tissue: 364
CD8$^+$ T cells → CD3$^+$ T cells
Tissue: 982
Endothelial cells → B cells
Tissue: 100
B cells → Endothelial cells
PC1
PC2

Fig. S6 – part 3

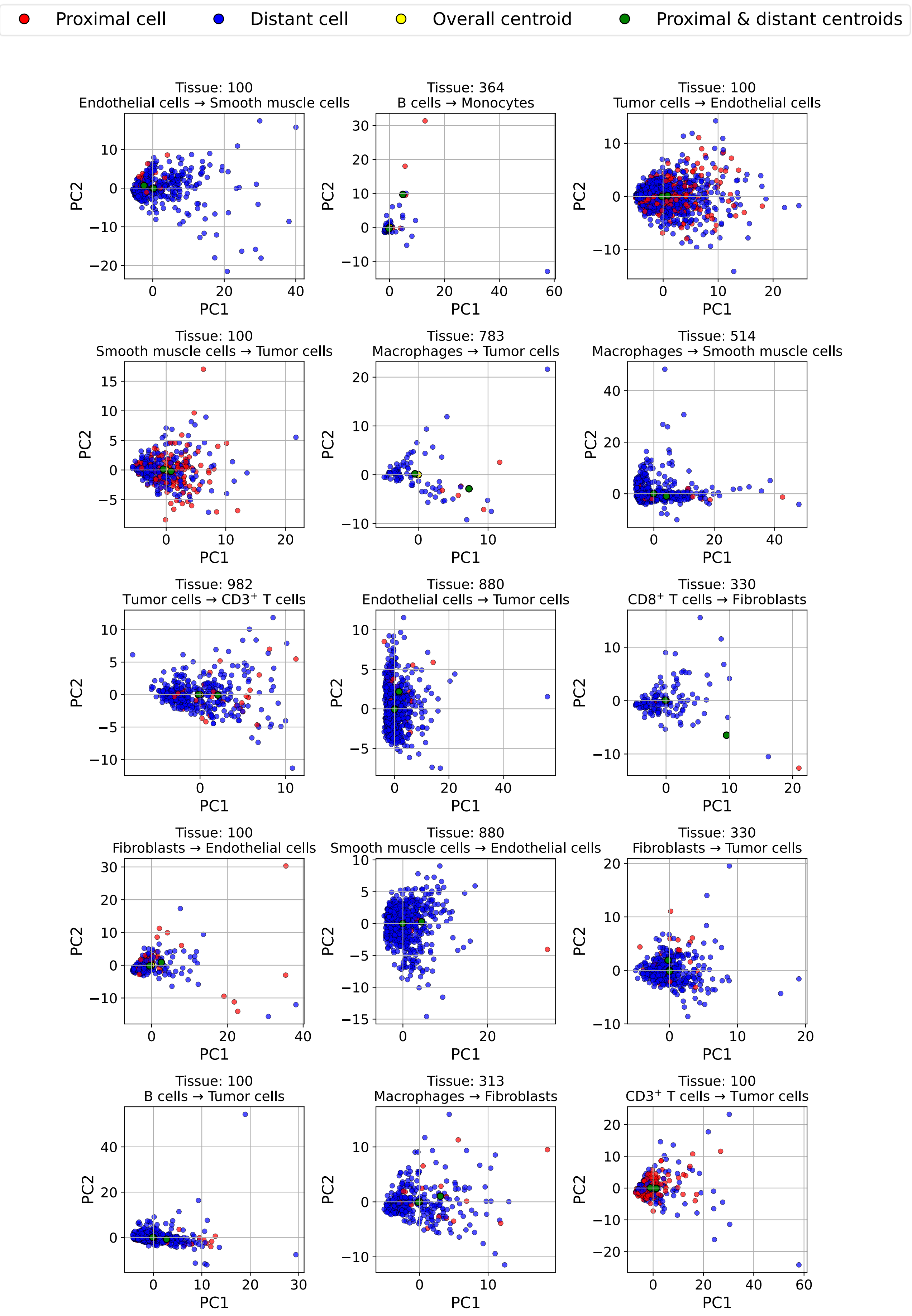
Proximal cell
Distant cell
Overall centroid
Proximal & distant centroids
Tissue: 100
Endothelial cells → Smooth muscle cells
Tissue: 364
B cells → Monocytes
Tissue: 100
Tumor cells → Endothelial cells
Tissue: 100
Smooth muscle cells → Tumor cells
Tissue: 783
Macrophages → Tumor cells
Tissue: 514
Macrophages → Smooth muscle cells
Tissue: 982
Tumor cells → CD3+ T cells
Tissue: 880
Endothelial cells → Tumor cells
Tissue: 330
CD8+ T cells → Fibroblasts
Tissue: 100
Fibroblasts → Endothelial cells
Tissue: 880
Smooth muscle cells → Endothelial cells
Tissue: 330
Fibroblasts → Tumor cells
Tissue: 100
B cells → Tumor cells
Tissue: 313
Macrophages → Fibroblasts
Tissue: 100
CD3+ T cells → Tumor cells
PC1
PC2

Fig. S6 – part 4

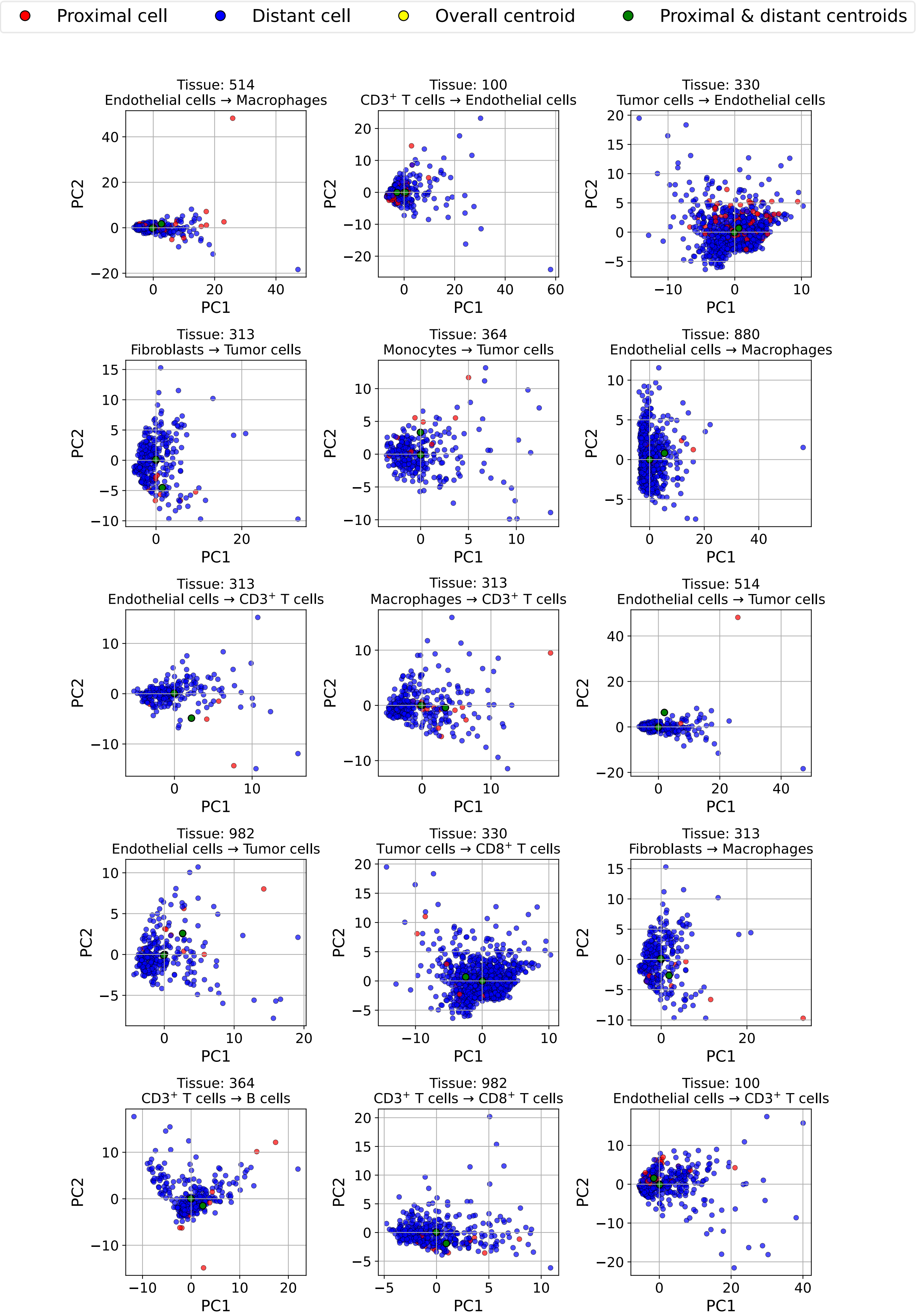

Proximal cell
Distant cell
Overall centroid
Proximal & distant centroids
Tissue: 514
Endothelial cells → Macrophages
Tissue: 100
CD3+ T cells → Endothelial cells
Tissue: 330
Tumor cells → Endothelial cells
Tissue: 313
Fibroblasts → Tumor cells
Tissue: 364
Monocytes → Tumor cells
Tissue: 880
Endothelial cells → Macrophages
Tissue: 313
Endothelial cells → CD3+ T cells
Tissue: 313
Macrophages → CD3+ T cells
Tissue: 514
Endothelial cells → Tumor cells
Tissue: 982
Endothelial cells → Tumor cells
Tissue: 330
Tumor cells → CD8+ T cells
Tissue: 313
Fibroblasts → Macrophages
Tissue: 364
CD3+ T cells → B cells
Tissue: 982
CD3+ T cells → CD8+ T cells
Tissue: 100
Endothelial cells → CD3+ T cells
PC1
PC2

Fig. S6 – part 5

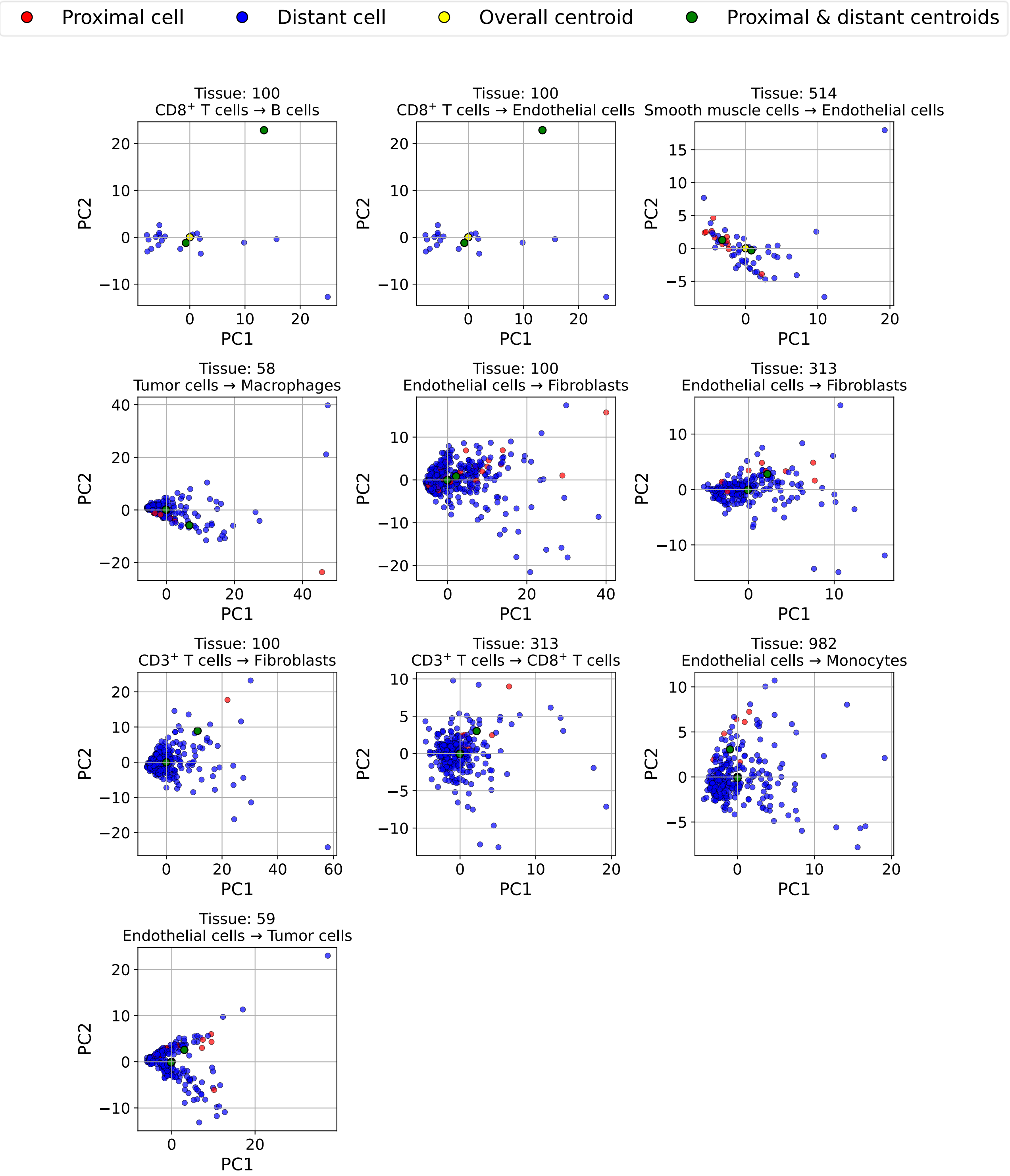
Proximal cell
Distant cell
Overall centroid
Proximal & distant centroids
Tissue: 100
CD8+ T cells → B cells
Tissue: 100
CD8+ T cells → Endothelial cells
Tissue: 514
Smooth muscle cells → Endothelial cells
Tissue: 58
Tumor cells → Macrophages
Tissue: 100
Endothelial cells → Fibroblasts
Tissue: 313
Endothelial cells → Fibroblasts
Tissue: 100
CD3+ T cells → Fibroblasts
Tissue: 313
CD3+ T cells → CD8+ T cells
Tissue: 982
Endothelial cells → Monocytes
Tissue: 59
Endothelial cells → Tumor cells
PC1
PC2

Fig. S6 – part 6

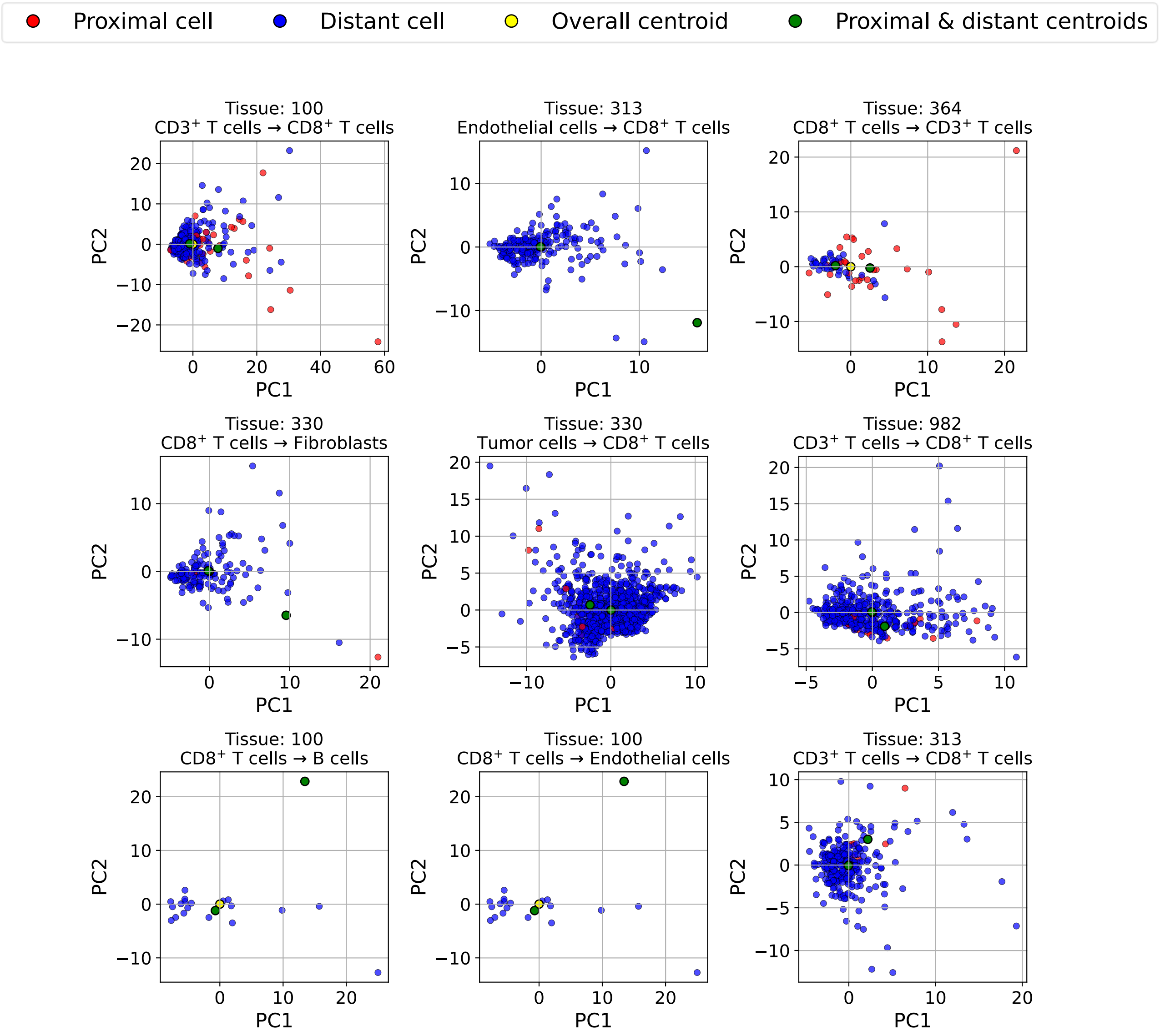

Proximal cell
Distant cell
Overall centroid
Proximal & distant centroids
Tissue: 100
CD3⁺ T cells → CD8⁺ T cells
Tissue: 313
Endothelial cells → CD8⁺ T cells
Tissue: 364
CD8⁺ T cells → CD3⁺ T cells
Tissue: 330
CD8⁺ T cells → Fibroblasts
Tissue: 330
Tumor cells → CD8⁺ T cells
Tissue: 982
CD3⁺ T cells → CD8⁺ T cells
Tissue: 100
CD8⁺ T cells → B cells
Tissue: 100
CD8⁺ T cells → Endothelial cells
Tissue: 313
CD3⁺ T cells → CD8⁺ T cells
PC1
PC2

Fig. S6. Proximity-related expression programs explain part of within–cell type variability. For each tissue and cell-type pair, with cell type *i* designated as the primary (first cell type in the subplot title) and cell type *ii* as the neighbor (second cell type in the subplot title), cells from the primary type were projected into two-dimensional PCA space based on their gene expression profiles. The projected type *i*-cells were partitioned into proximal (≤1 μm from neighbor type *ii*-cell) and distant subsets, and were colored in red and blue colors respectively. An overall centroid defined by all type *i*-cells was presented as a yellow dot, and the two centroids of 'proximal' and 'distant' type *i*-subsets were presented as green dots. Details regarding dispersion values and their statistical significance for each tissue and cell types combination are reported in Table S2. Note that part 6 displays only cell-type pairs involving $CD8^+$ T cells.

Fig. S7 – part 1

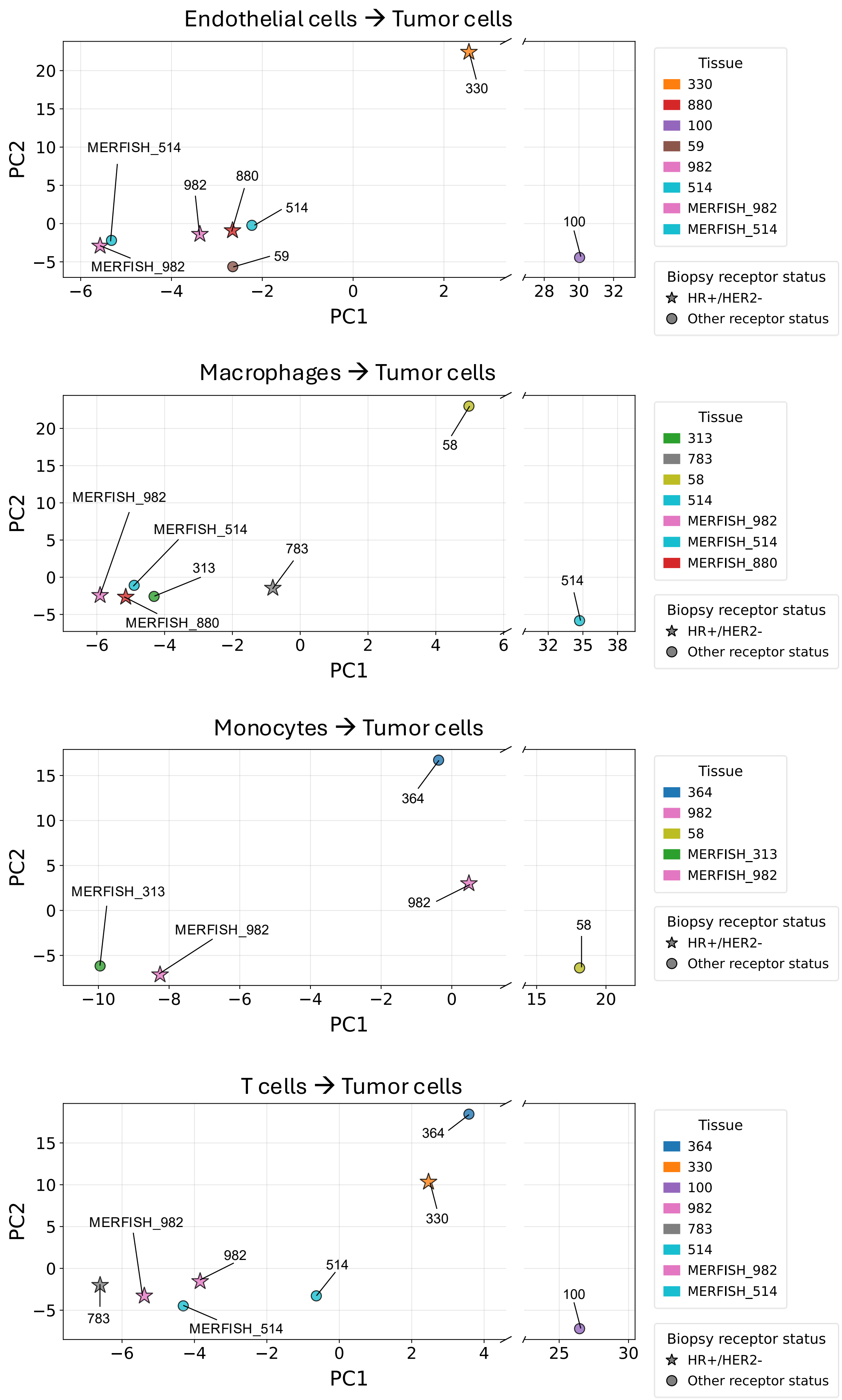
Endothelial cells → Tumor cells
PC2
PC1
330
MERFISH_514
982
880
514
MERFISH_982
59
100
Tissue
330
880
100
59
982
514
MERFISH_982
MERFISH_514
Biopsy receptor status
HR+/HER2-
Other receptor status
Macrophages → Tumor cells
58
MERFISH_982
MERFISH_514
783
313
MERFISH_880
514
Tissue
313
783
58
514
MERFISH_982
MERFISH_514
MERFISH_880
Biopsy receptor status
HR+/HER2-
Other receptor status
Monocytes → Tumor cells
364
982
MERFISH_313
MERFISH_982
58
Tissue
364
982
58
MERFISH_313
MERFISH_982
Biopsy receptor status
HR+/HER2-
Other receptor status
T cells → Tumor cells
364
330
MERFISH_982
982
514
783
MERFISH_514
100
Tissue
364
330
100
982
783
514
MERFISH_982
MERFISH_514
Biopsy receptor status
HR+/HER2-
Other receptor status

Fig. S7 – part 2

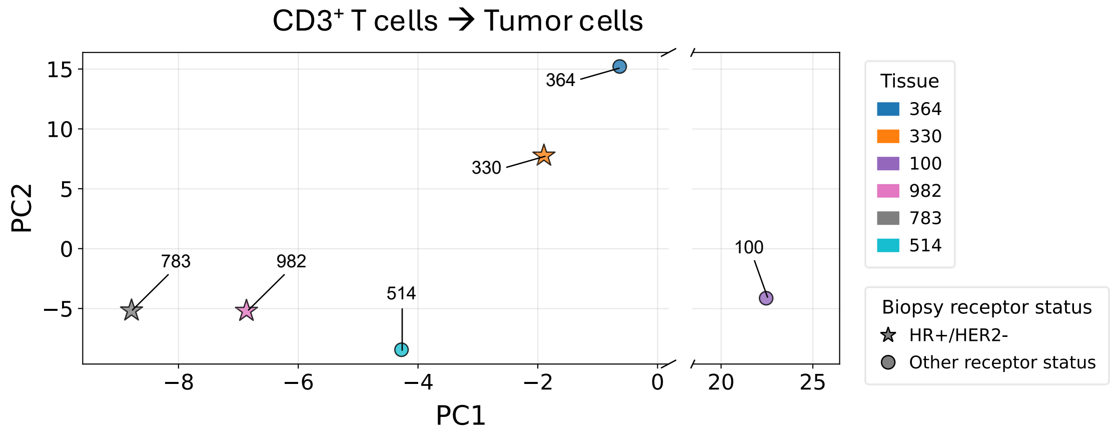
CD3+ T cells → Tumor cells
PC1
PC2
364
330
100
783
982
514
Tissue
364
330
100
982
783
514
Biopsy receptor status
HR+/HER2-
Other receptor status

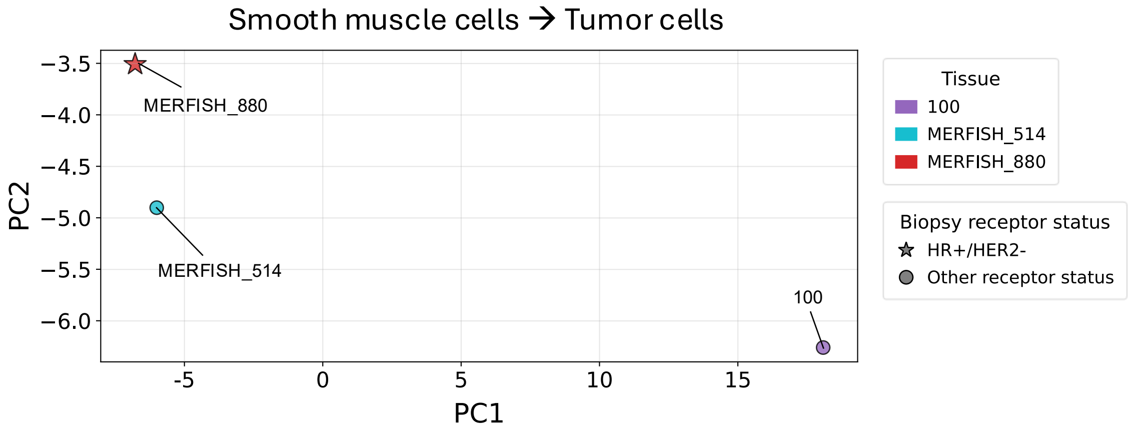
Smooth muscle cells → Tumor cells
PC1
PC2
MERFISH_880
MERFISH_514
100
Tissue
100
MERFISH_514
MERFISH_880
Biopsy receptor status
HR+/HER2-
Other receptor status

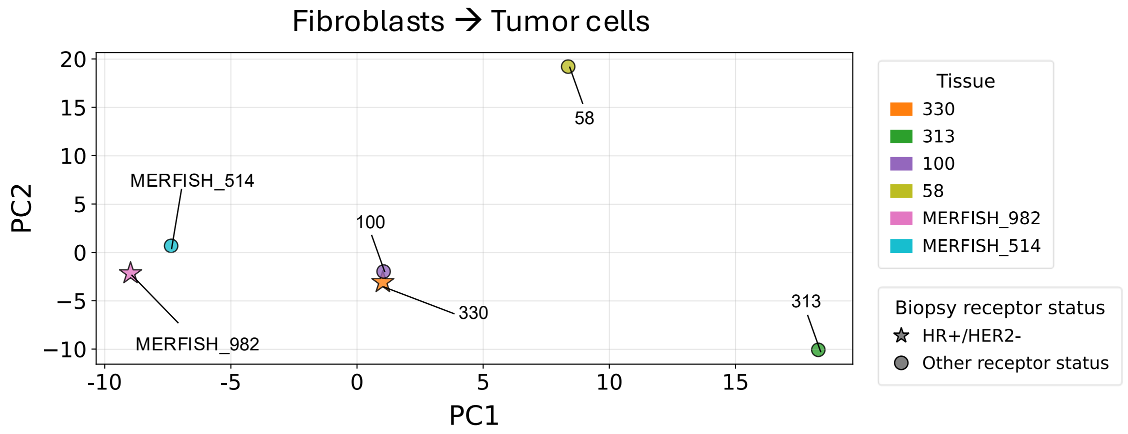
Fibroblasts → Tumor cells
PC1
PC2
58
MERFISH_514
100
330
313
MERFISH_982
Tissue
330
313
100
58
MERFISH_982
MERFISH_514
Biopsy receptor status
HR+/HER2-
Other receptor status

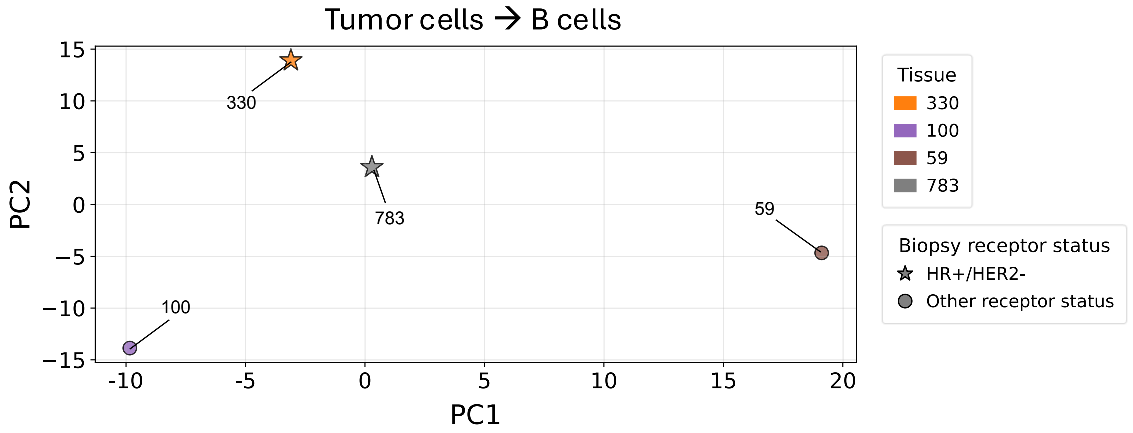
Tumor cells → B cells
PC1
PC2
330
783
59
100
Tissue
330
100
59
783
Biopsy receptor status
HR+/HER2-
Other receptor status

Fig. S7 – part 3

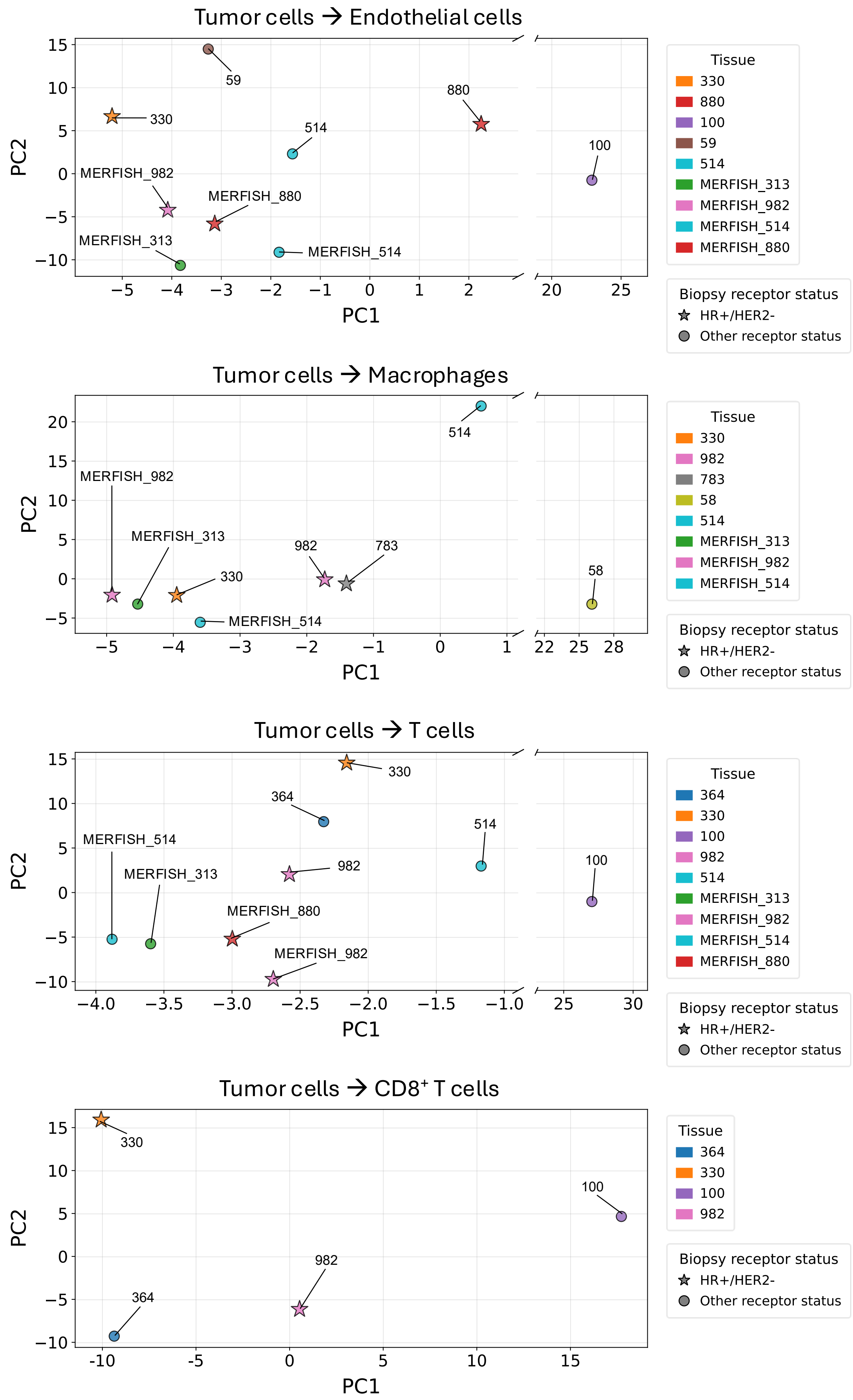
Tumor cells → Endothelial cells
PC2
PC1
59
330
880
514
100
MERFISH_982
MERFISH_880
MERFISH_313
MERFISH_514
Tissue
330
880
100
59
514
MERFISH_313
MERFISH_982
MERFISH_514
MERFISH_880
Biopsy receptor status
HR+/HER2-
Other receptor status
Tumor cells → Macrophages
PC2
PC1
514
MERFISH_982
MERFISH_313
982
783
330
58
MERFISH_514
Tissue
330
982
783
58
514
MERFISH_313
MERFISH_982
MERFISH_514
Biopsy receptor status
HR+/HER2-
Other receptor status
Tumor cells → T cells
PC2
PC1
330
364
514
MERFISH_514
MERFISH_313
982
100
MERFISH_880
MERFISH_982
Tissue
364
330
100
982
514
MERFISH_313
MERFISH_982
MERFISH_514
MERFISH_880
Biopsy receptor status
HR+/HER2-
Other receptor status
Tumor cells → CD8+ T cells
PC2
PC1
330
100
982
364
Tissue
364
330
100
982
Biopsy receptor status
HR+/HER2-
Other receptor status

Fig. S7 – part 4

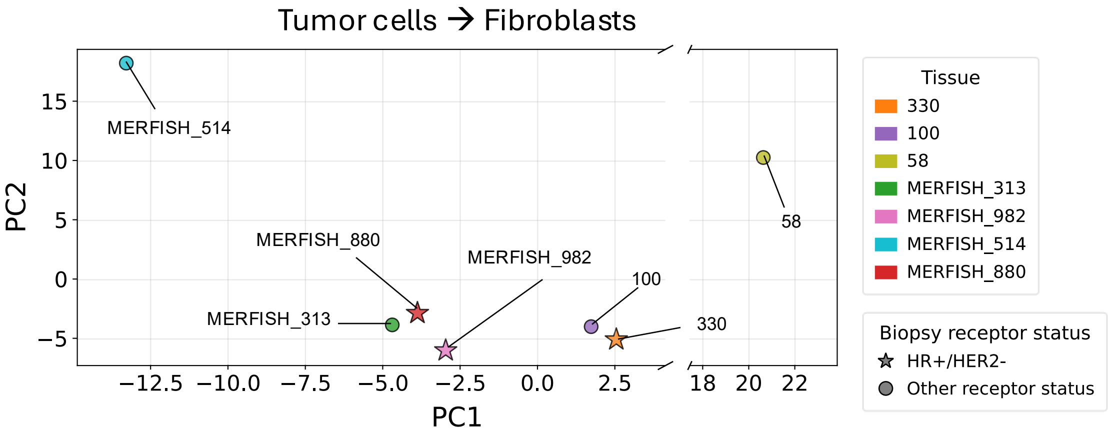

Tumor cells → Fibroblasts
PC2
PC1
15
10
5
0
−5
−12.5
−10.0
−7.5
−5.0
−2.5
0.0
2.5
18
20
22
MERFISH_514
MERFISH_880
MERFISH_982
MERFISH_313
100
330
58
Tissue
330
100
58
MERFISH_313
MERFISH_982
MERFISH_514
MERFISH_880
Biopsy receptor status
HR+/HER2-
Other receptor status

Fig. S7. Comparison of proximity-related expression programs across platforms and patients. For each cell-type pair, with cell type *i* designated as the primary (first cell type in the subplot title) and cell type *ii* as the neighbor (second cell type in the subplot title), differential expression was assessed between primary-type cells located proximal (≤1 μm) versus distant from neighbor-type cells. Analyses were performed across all tissues, incorporating both ExSeq and MERFISH datasets from adjacent sections of the same samples when available. For each dataset, DESeq2 *p*-values of proximity-induced genes were extracted and projected into principal component analysis (PCA) space, positioning each patient–platform combination (ExSeq or MERFISH) in the two-dimensional embedding. Individual patients are distinguished by color; $HR^{+}/HER2^{-}$ cases are marked with a star, and other receptor subtypes are shown as circles. Concordance of proximity-related programs between ExSeq and MERFISH and across patients with similar receptor status is summarized in Table S5.

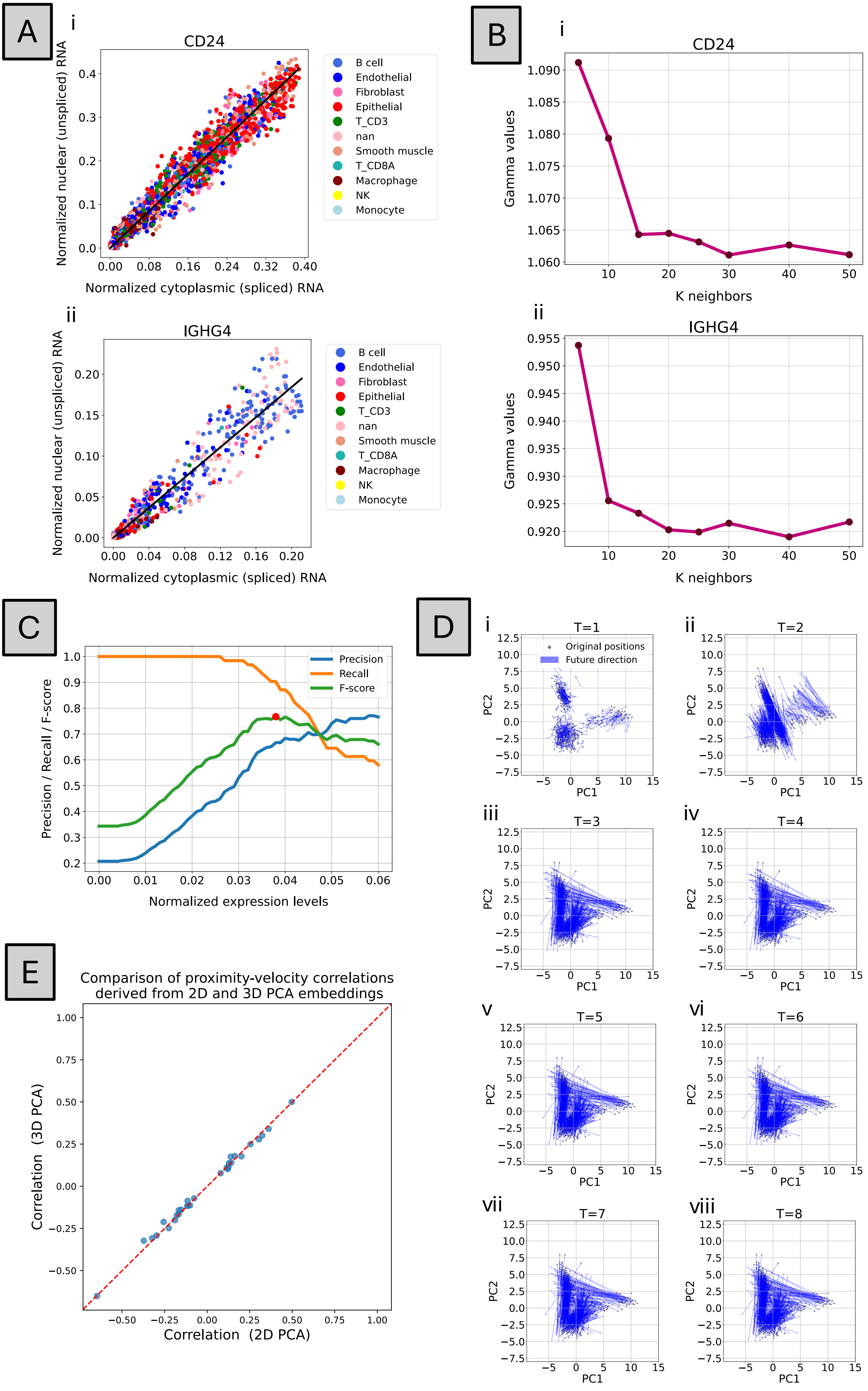
A
i
CD24
Normalized nuclear (unspliced) RNA
Normalized cytoplasmic (spliced) RNA
B cell
Endothelial
Fibroblast
Epithelial
T_CD3
nan
Smooth muscle
T_CD8A
Macrophage
NK
Monocyte
ii
IGHG4
Normalized nuclear (unspliced) RNA
Normalized cytoplasmic (spliced) RNA
B
i
CD24
Gamma values
K neighbors
ii
IGHG4
Gamma values
K neighbors
C
Precision / Recall / F-score
Normalized expression levels
Precision
Recall
F-score
D
i
T=1
Original positions
Future direction
PC1
PC2
ii
T=2
iii
T=3
iv
T=4
v
T=5
vi
T=6
vii
T=7
viii
T=8
E
Comparison of proximity-velocity correlations
derived from 2D and 3D PCA embeddings
Correlation (3D PCA)
Correlation (2D PCA)

Fig. S8. RNA velocity parameters selection. (A) (i) An example of a phase portrait, showing the *CD24* gene. *CD24* is a known marker for tumor cells and accordingly this gene exhibits high expression levels in tumor cells (red dots in the top right corner). (ii) Another example of a phase portrait, showing the *IGHG4* gene. *IGHG4* is a known marker for B cells, and accordingly this gene exhibits high expression levels in B cells (royal blue dots in the top right corner). **(B)** Evaluation of γ stability across K-nearest neighbor smoothing parameters. We evaluated a range of K values for all 299 genes and found that γ estimates were stable around K = 30. K represents the number of nearest neighbors: each cell's cytoplasmic and nuclear counts were replaced with the mean counts of the cell and its K nearest neighbors in gene-expression space. Neighbors were identified by performing PCA on the normalized cytoplasmic expression matrix and using Euclidean distance in the first three principal components (PC1–PC3). (i) γ values for the *CD24* gene as a function of K. (ii) γ values for the *IGHG4* gene as a function of K. **(C)** Evaluation of minimum expression cutoff for gene inclusion in future cell-state calculations. We evaluated a range of expression thresholds to identify genes whose phase portraits would yield reliable γ values (Methods). An expression cutoff of 0.038 maximized the F-score, balancing precision and recall. Genes with a maximum normalized nuclear or cytoplasmic expression ≥ 0.038 were considered to have robust γ values. In total, 84 genes met this criterion and were used for subsequent future cell-state analyses. **(D)** Sensitivity analysis for determining the optimal time-step parameter (*T*). Sensitivity analyses were performed to identify the maximum time step that yielded robust future-state projections while maintaining stability in total cytoplasmic expression. *T* = 3 showed robust velocity estimates while also ensuring that total cytoplasmic expression per cell at the projected time point deviated by no more than 10% from its initial value. This parameter was used in all subsequent analyses. **(E)** Consistency of proximity-state correlations between 2D and 3D PCA embeddings. Correlations between inter-cell-type physical proximity and the magnitude of change in cell-state were calculated using either a 2D or 3D PCA embedding. Each point corresponds to a primary-neighbor cell type pair. The near-identity between the two estimates indicates that inclusion of the third principal component does not materially affect the inferred spatial-dynamic relationships (Pearson correlation r = 0.99, $p = 3 \times 10^{-33}$).

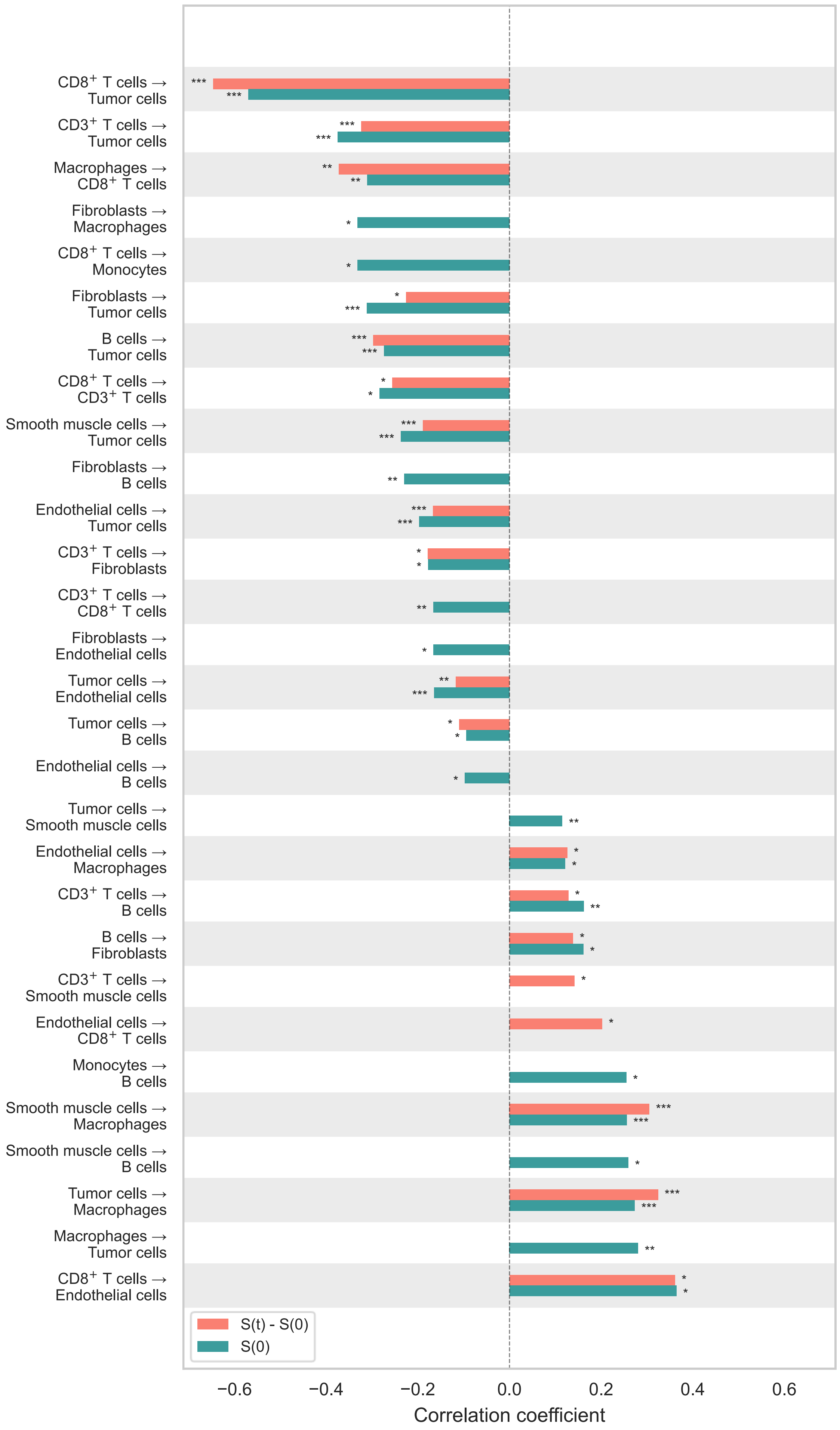
CD8+ T cells → Tumor cells
CD3+ T cells → Tumor cells
Macrophages → CD8+ T cells
Fibroblasts → Macrophages
CD8+ T cells → Monocytes
Fibroblasts → Tumor cells
B cells → Tumor cells
CD8+ T cells → CD3+ T cells
Smooth muscle cells → Tumor cells
Fibroblasts → B cells
Endothelial cells → Tumor cells
CD3+ T cells → Fibroblasts
CD3+ T cells → CD8+ T cells
Fibroblasts → Endothelial cells
Tumor cells → Endothelial cells
Tumor cells → B cells
Endothelial cells → B cells
Tumor cells → Smooth muscle cells
Endothelial cells → Macrophages
CD3+ T cells → B cells
B cells → Fibroblasts
CD3+ T cells → Smooth muscle cells
Endothelial cells → CD8+ T cells
Monocytes → B cells
Smooth muscle cells → Macrophages
Smooth muscle cells → B cells
Tumor cells → Macrophages
Macrophages → Tumor cells
CD8+ T cells → Endothelial cells
S(t) - S(0)
S(0)
−0.6
−0.4
−0.2
0.0
0.2
0.4
0.6
Correlation coefficient

Fig. S9. Correlation between physical distance and magnitude of cell-state change across cell types. For each primary-neighbor cell-type pair shown in the rows (primary before the arrow, neighbor after), the minimum physical distance was calculated between cells of the primary type and cells of the neighbor type. For each primary cell, the magnitude of change in cell state was quantified as the Euclidean distance in PCA space between its initial state $S(0)$ and projected future state $S(\mathrm{T})$. The magnitude of the current state was defined as the distance between $S(0)$ and the origin of the PCA space. Shown are Pearson correlation coefficients between physical distance and the magnitude of the future-state change (red) or the magnitude of the current state (green)**.** Asterisks indicate Benjamini-Hochberg-corrected permutation-based *p*-values (*$q < 0.05$; **$q < 0.01$; ***$q < 0.001$).

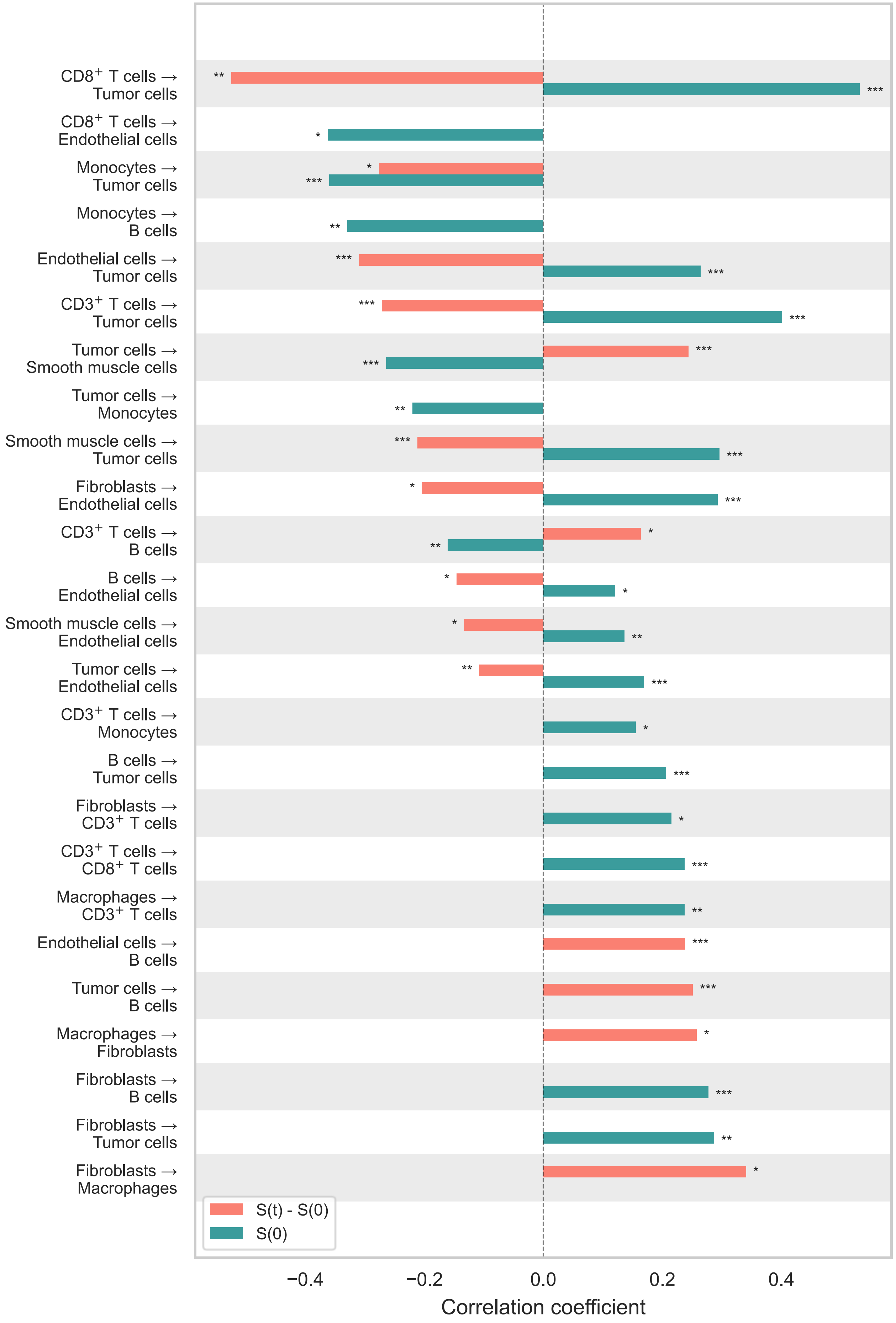

CD8+ T cells → Tumor cells
CD8+ T cells → Endothelial cells
Monocytes → Tumor cells
Monocytes → B cells
Endothelial cells → Tumor cells
CD3+ T cells → Tumor cells
Tumor cells → Smooth muscle cells
Tumor cells → Monocytes
Smooth muscle cells → Tumor cells
Fibroblasts → Endothelial cells
CD3+ T cells → B cells
B cells → Endothelial cells
Smooth muscle cells → Endothelial cells
Tumor cells → Endothelial cells
CD3+ T cells → Monocytes
B cells → Tumor cells
Fibroblasts → CD3+ T cells
CD3+ T cells → CD8+ T cells
Macrophages → CD3+ T cells
Endothelial cells → B cells
Tumor cells → B cells
Macrophages → Fibroblasts
Fibroblasts → B cells
Fibroblasts → Tumor cells
Fibroblasts → Macrophages
S(t) - S(0)
S(0)
−0.4
−0.2
0.0
0.2
0.4
Correlation coefficient

Fig. S10. Correlation between physical distance and phase of cell-state change across cell types. For each primary-neighbor cell-type pair shown in the rows (primary before the arrow, neighbor after), the minimum physical distance was calculated between cells of the primary type and cells of the neighbor type. For each primary cell, the phase of the change in cell state was defined as the angle between its initial position $S(0)$ and projected future position $S(T)$ in PCA space, projected onto the first two principal components (PC1 and PC2). The phase of the current state was defined as the angle between the initial position and the origin of the PCA space. Shown are Pearson correlation coefficients between physical distance and the phase of the future-state change (red) or the phase of the current state (green). Asterisks indicate Benjamini-Hochberg-corrected permutation-based p-values (*$q < 0.05$; **$q < 0.01$; ***$q < 0.001$).

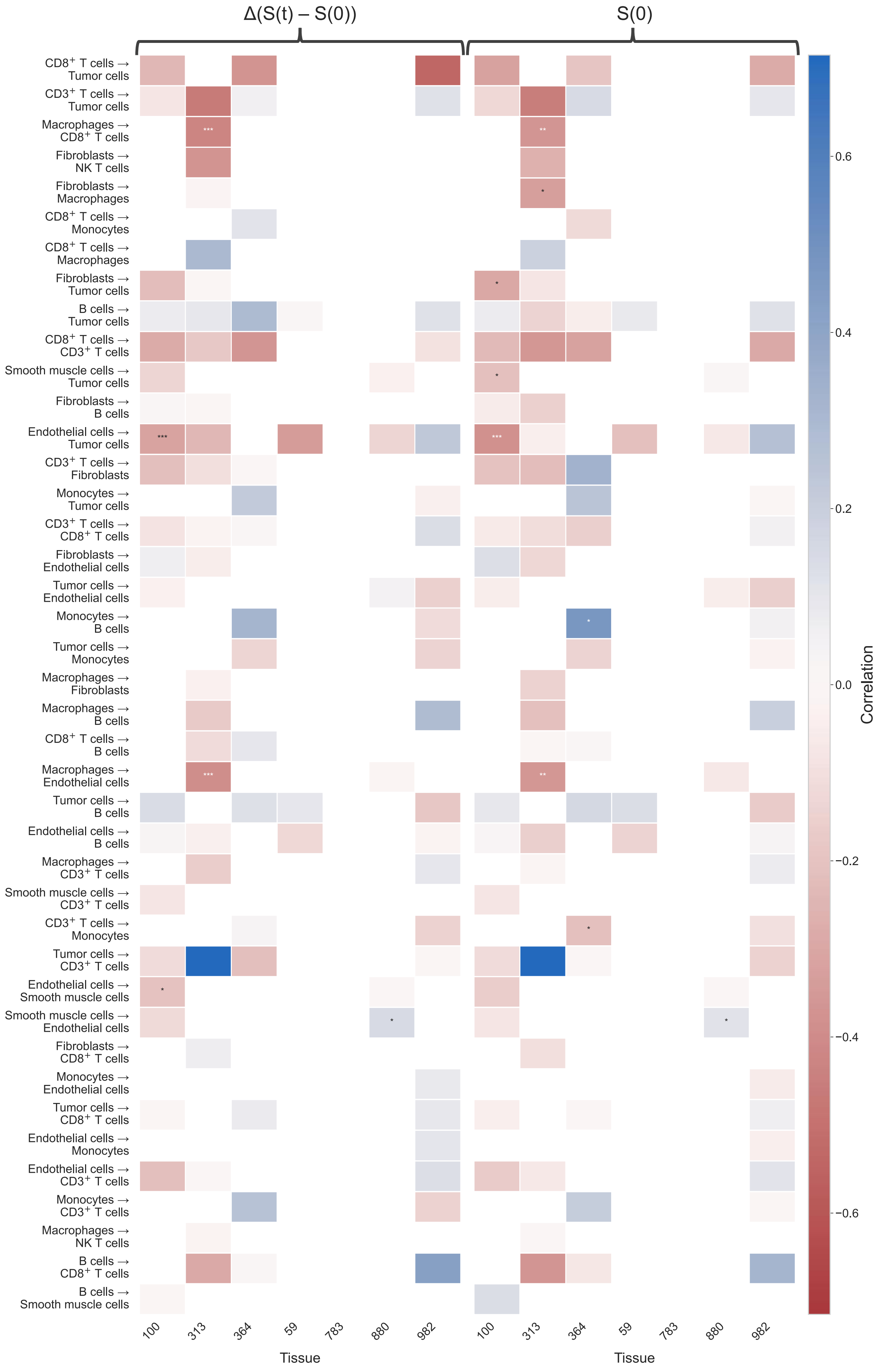

Δ(S(t) – S(0))
S(0)
CD8+ T cells → Tumor cells
CD3+ T cells → Tumor cells
Macrophages → CD8+ T cells
Fibroblasts → NK T cells
Fibroblasts → Macrophages
CD8+ T cells → Monocytes
CD8+ T cells → Macrophages
Fibroblasts → Tumor cells
B cells → Tumor cells
CD8+ T cells → CD3+ T cells
Smooth muscle cells → Tumor cells
Fibroblasts → B cells
Endothelial cells → Tumor cells
CD3+ T cells → Fibroblasts
Monocytes → Tumor cells
CD3+ T cells → CD8+ T cells
Fibroblasts → Endothelial cells
Tumor cells → Endothelial cells
Monocytes → B cells
Tumor cells → Monocytes
Macrophages → Fibroblasts
Macrophages → B cells
CD8+ T cells → B cells
Macrophages → Endothelial cells
Tumor cells → B cells
Endothelial cells → B cells
Macrophages → CD3+ T cells
Smooth muscle cells → CD3+ T cells
CD3+ T cells → Monocytes
Tumor cells → CD3+ T cells
Endothelial cells → Smooth muscle cells
Smooth muscle cells → Endothelial cells
Fibroblasts → CD8+ T cells
Monocytes → Endothelial cells
Tumor cells → CD8+ T cells
Endothelial cells → Monocytes
Endothelial cells → CD3+ T cells
Monocytes → CD3+ T cells
Macrophages → NK T cells
B cells → CD8+ T cells
B cells → Smooth muscle cells
100
313
364
59
783
880
982
Tissue
0.6
0.4
0.2
0.0
−0.2
−0.4
−0.6
Correlation

Fig. S11. Correlating physical distance between cell types and magnitude of change in cell state per tissue. The same as Fig. S9, but displaying correlation values for each tissue individually rather than combining all data. Asterisks indicate Benjamini-Hochberg-corrected permutation-based *p*-values (*$q < 0.05$; **$q < 0.01$; ***$q < 0.001$).

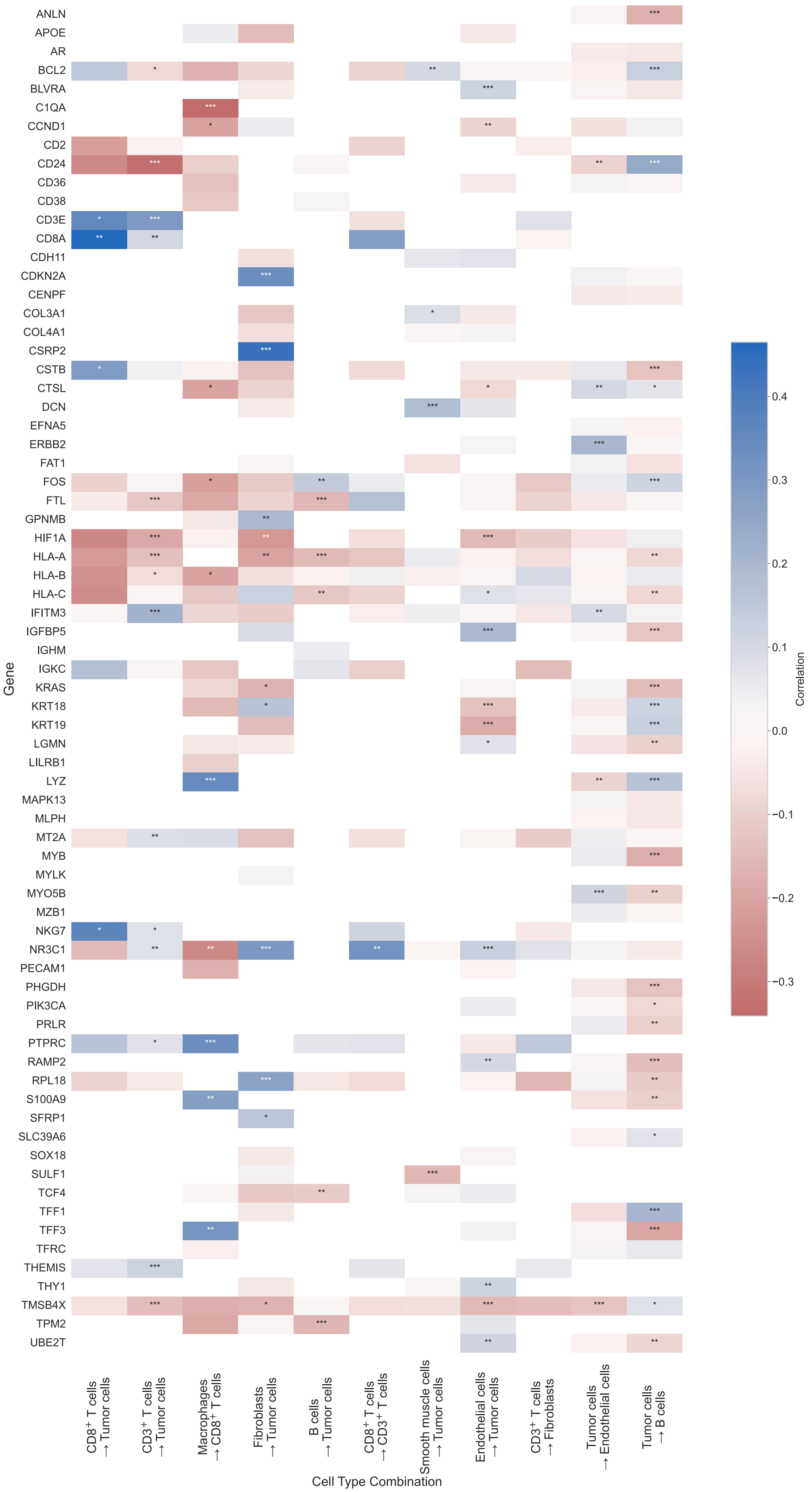

Gene
ANLN
APOE
AR
BCL2
BLVRA
C1QA
CCND1
CD2
CD24
CD36
CD38
CD3E
CD8A
CDH11
CDKN2A
CENPF
COL3A1
COL4A1
CSRP2
CSTB
CTSL
DCN
EFNA5
ERBB2
FAT1
FOS
FTL
GPNMB
HIF1A
HLA-A
HLA-B
HLA-C
IFITM3
IGFBP5
IGHM
IGKC
KRAS
KRT18
KRT19
LGMN
LILRB1
LYZ
MAPK13
MLPH
MT2A
MYB
MYLK
MYO5B
MZB1
NKG7
NR3C1
PECAM1
PHGDH
PIK3CA
PRLR
PTPRC
RAMP2
RPL18
S100A9
SFRP1
SLC39A6
SOX18
SULF1
TCF4
TFF1
TFF3
TFRC
THEMIS
THY1
TMSB4X
TPM2
UBE2T
CD8⁺ T cells → Tumor cells
CD3⁺ T cells → Tumor cells
Macrophages → CD8⁺ T cells
Fibroblasts → Tumor cells
B cells → Tumor cells
CD8⁺ T cells → CD3⁺ T cells
Smooth muscle cells → Tumor cells
Endothelial cells → Tumor cells
CD3⁺ T cells → Fibroblasts
Tumor cells → Endothelial cells
Tumor cells → B cells
Cell Type Combination
Correlation
0.4
0.3
0.2
0.1
0.0
−0.1
−0.2
−0.3

Fig. S12. Correlation between gene-specific RNA velocity and physical distance between cell types. For each primary-neighbor cell-type pair shown in the columns (primary before the arrow, neighbor after), the minimum physical distance was calculated between cells of the primary and neighbor types. For each primary-neighbor pair and each gene shown in the rows, the gene's RNA velocity across all primary cells was correlated with the corresponding minimum distances to the neighbor cell type, and the resulting Pearson correlation coefficients are shown. Only genes expressed in more than 20% of cells within the primary type (based on scRNA-seq data) are included. An exception is *CD24*, which was retained due to its reported upregulation in activated T cells(2, 3). Only cell-type pairs with significant correlations between physical distance and the magnitude of change in cell state are shown (see Fig. S9). Asterisks indicate Benjamini-Hochberg-corrected permutation-based *p*-values (*$q < 0.1$; **$q < 0.05$; ***$q < 0.01$).

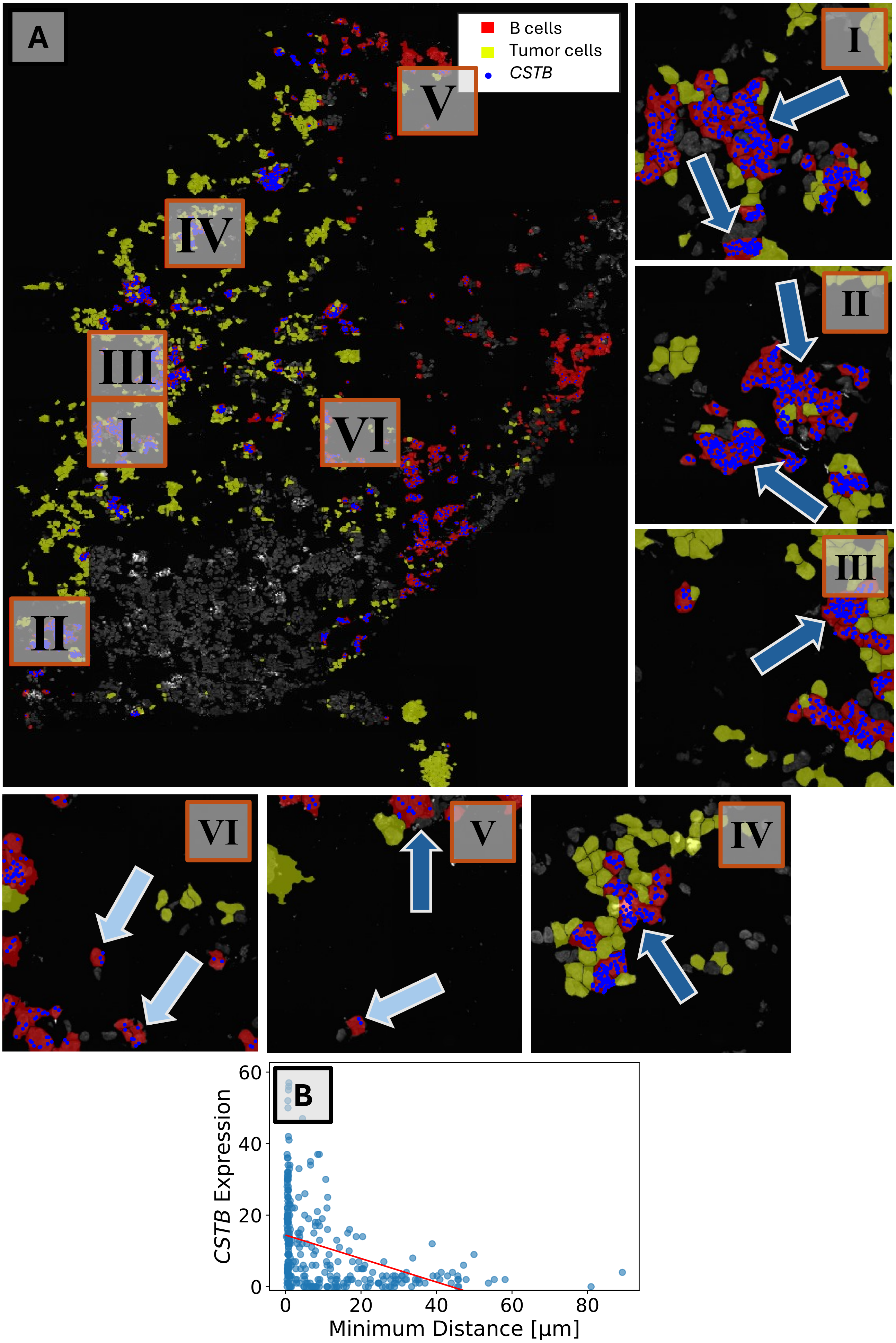
A
B cells
Tumor cells
CSTB
I
II
III
IV
V
VI
B
60
40
20
0
CSTB Expression
0
20
40
60
80
Minimum Distance [µm]

Fig. S13. Example of proximity-dependent gene expression: *CSTB* expression in B cells relative to tumor cells in biopsy 59. (A) Sequencing read locations for *CSTB* (blue dots) are overlaid on DAPI-stained nuclei, together with segmentations of B cells (red) and tumor cells (yellow). Only B-cell and tumor-cell segmentations are shown. A full tissue section and representative 40× fields of view (100 × 100 µm) are displayed. B cells proximal to tumor cells exhibit higher *CSTB* expression (solid blue arrows) compared with distal B cells (hollow blue arrows). **(B)** Linear dependency of *CSTB* expression in B cells on distance to tumor cells (p from regression coefficient; t-test, *statsmodels*; $q = 2 \times 10^{-11}$, slope = −0.33).

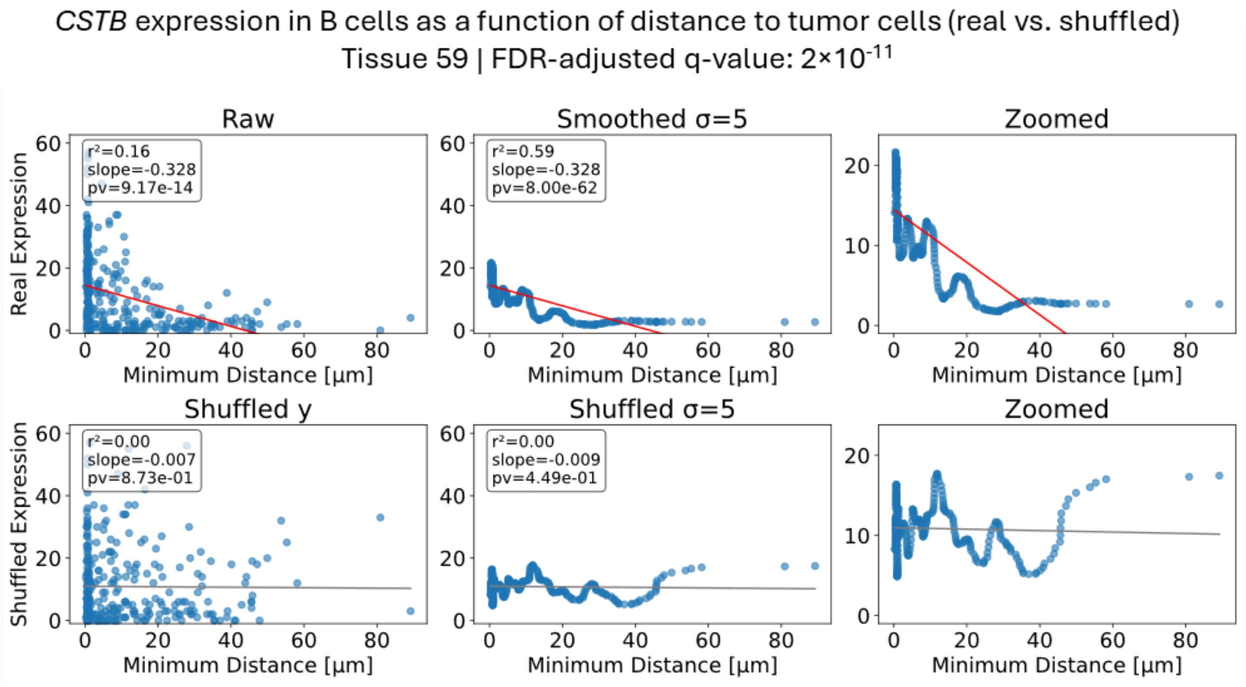

Fig. S14. *CSTB* expression in B cells as a function of distance to tumor cells in biopsy 59. A linear dependency of *CSTB* expression in B cells was observed as a function of the distance to tumor cells (top row), which was not present when expression values were shuffled (bottom row). For each B cell, the minimum distance to the nearest tumor cell was calculated. The left column shows the raw data with linear regression, the middle column shows smoothed expression using a Gaussian filter (σ = 5) with linear regression, and the right column shows a zoomed view of the smoothed data. Each subplot includes the R^2, slope, and p-value (t-test, *statsmodels*) of the linear regression. The top row demonstrates a statistically significant negative relationship between *CSTB* expression and proximity to tumor cells ($q = 2 \times 10^{-11}$, slope = −0.33, smoothed $R^2 = 0.59$), which is absent in the shuffled controls (slope = −0.01, $R^2 \approx 0$).

CSTB expression in B cells as a function of distance to tumor cells across smoothing levels
Tissue 59 | FDR-adjusted q-value: 2×10^{-11}

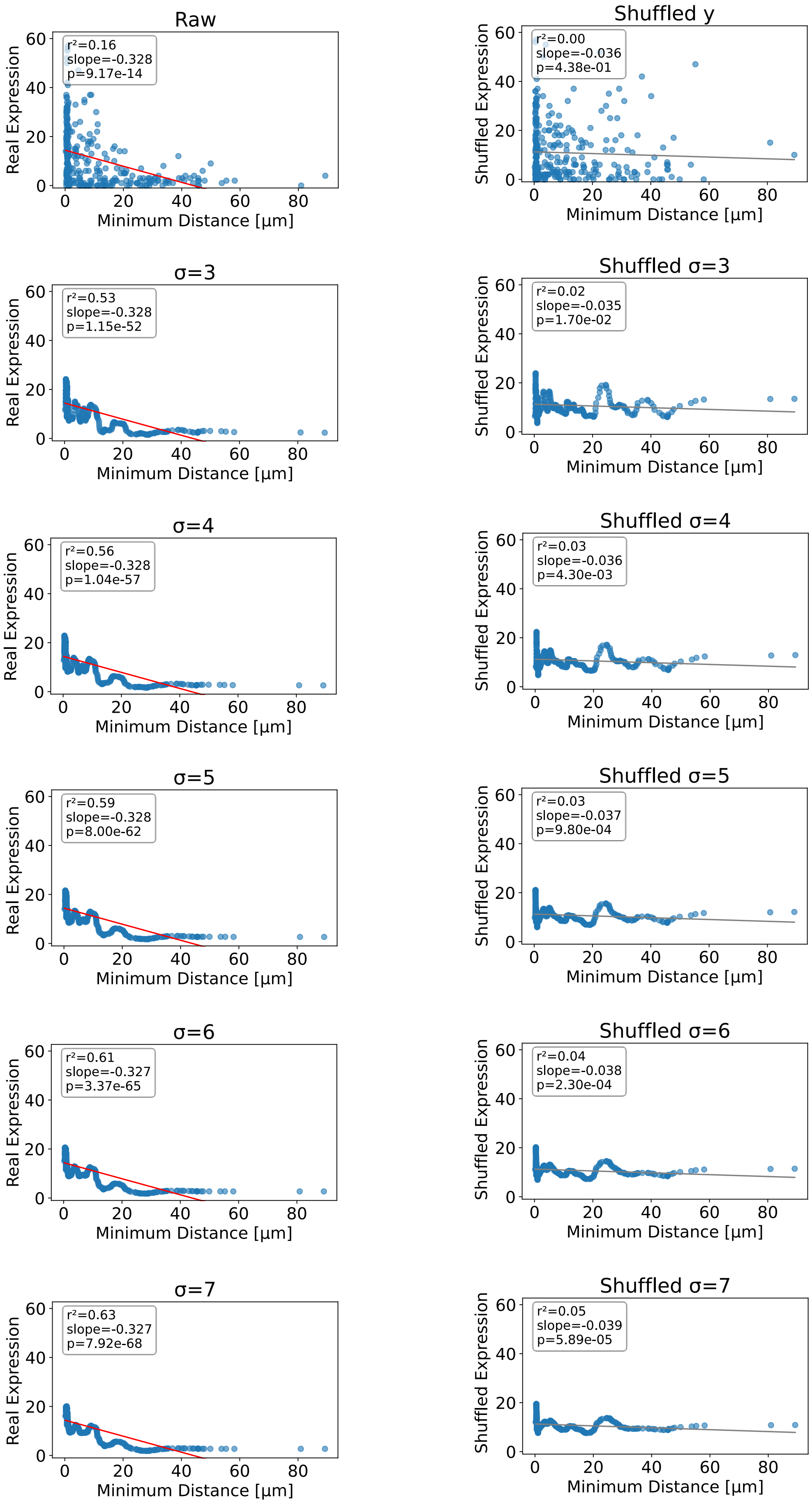

Fig. S15. *CSTB* expression in B cells relative to tumor cells in biopsy 59 across Gaussian smoothing levels. Same analysis as in Fig. S14, but with increasing levels of Gaussian smoothing applied to the expression vector ($\sigma = 3$ to 7). A strong negative distance-dependent trend is observed in the real data, which becomes more pronounced with greater smoothing (e.g., $R^2 = 0.63$, slope = −0.33, *p*-value = 8×10^{-68} at $\sigma = 7$), whereas shuffled controls consistently show no association. The observed trend is statistically significant ($q = 2 \times 10^{-11}$).

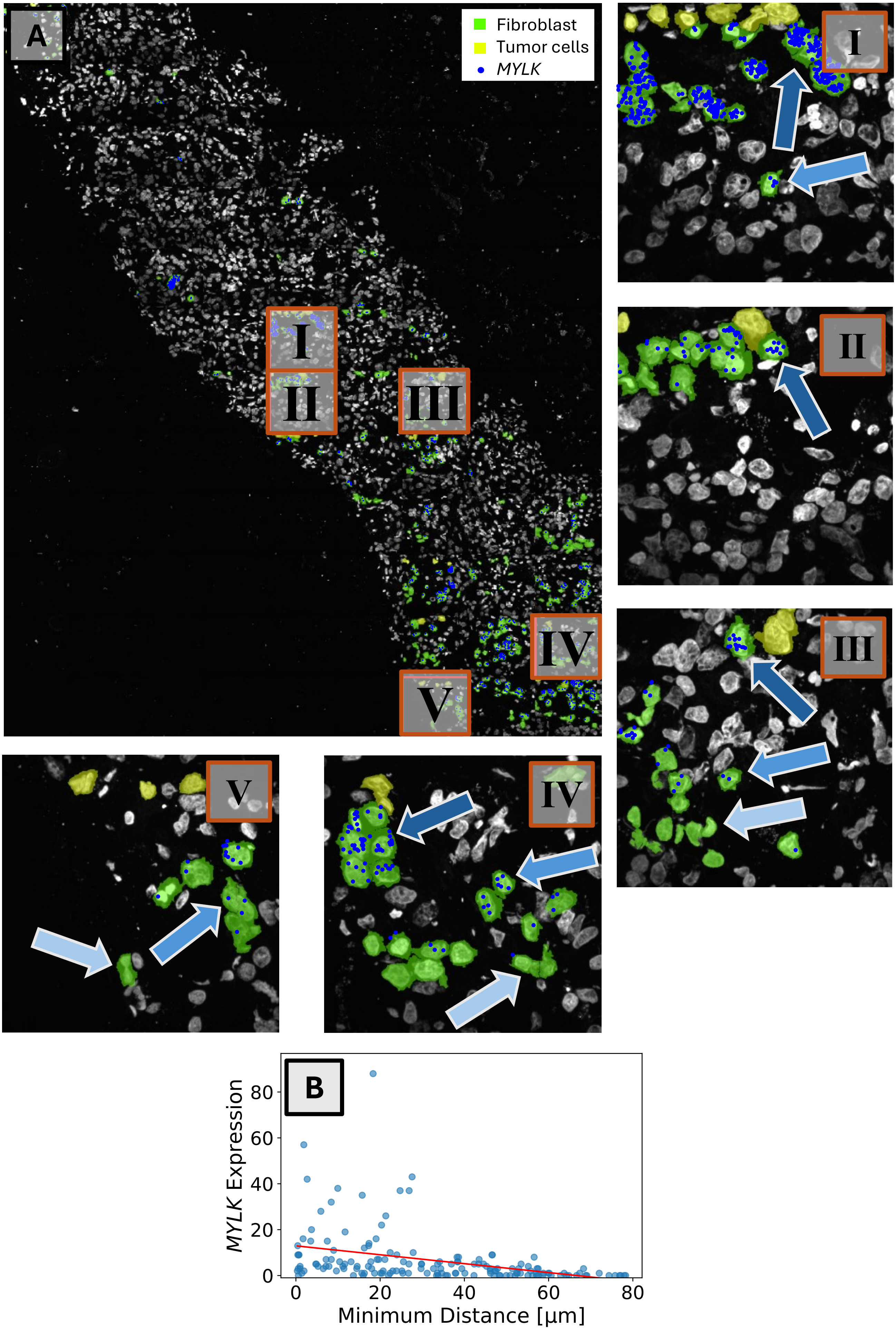

A
Fibroblast
Tumor cells
MYLK
I
II
III
IV
V
B
MYLK Expression
80
60
40
20
0
0
20
40
60
80
Minimum Distance [µm]

Fig. S16. Example of proximity-dependent gene expression: *MYLK* expression in fibroblasts relative to tumor cells in biopsy 313. (A) Sequencing read locations for *MYLK* (blue dots) are overlaid on DAPI-stained nuclei, together with segmentations of fibroblasts (green) and tumor cells (yellow). Only fibroblast and tumor-cell segmentations are shown. A full tissue section and representative 40× fields of view (100 × 100 µm) are displayed. Fibroblasts proximal to tumor cells exhibit higher *MYLK* expression (solid blue arrows) compared with distal fibroblasts (hollow blue arrows). **(B)** Linear dependency of *MYLK* expression in fibroblasts on distance to tumor cells (p from regression coefficient; t-test, *statsmodels*; $q = 6 \times 10^{-5}$, slope = −0.19).

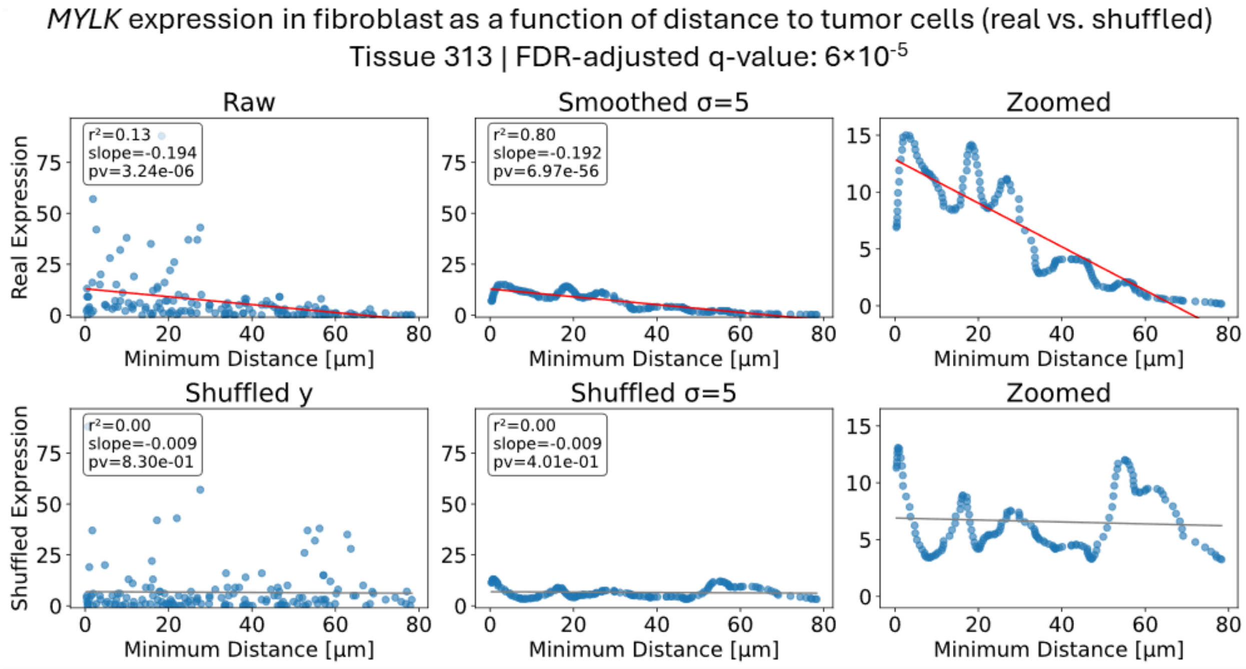

Fig. S17. *MYLK* expression in fibroblasts as a function of distance to tumor cells in biopsy 313. The data of Fig. S16, but with the permutation analysis as in Fig. S14. The top row demonstrates a statistically significant negative relationship between *MYLK* expression and proximity to tumor cells ($q = 6 \times 10^{-5}$, slope = -0.19, smoothed $R^2 = 0.8$), which is absent in the shuffled controls (slope = -0.01, $R^2 \approx 0$).

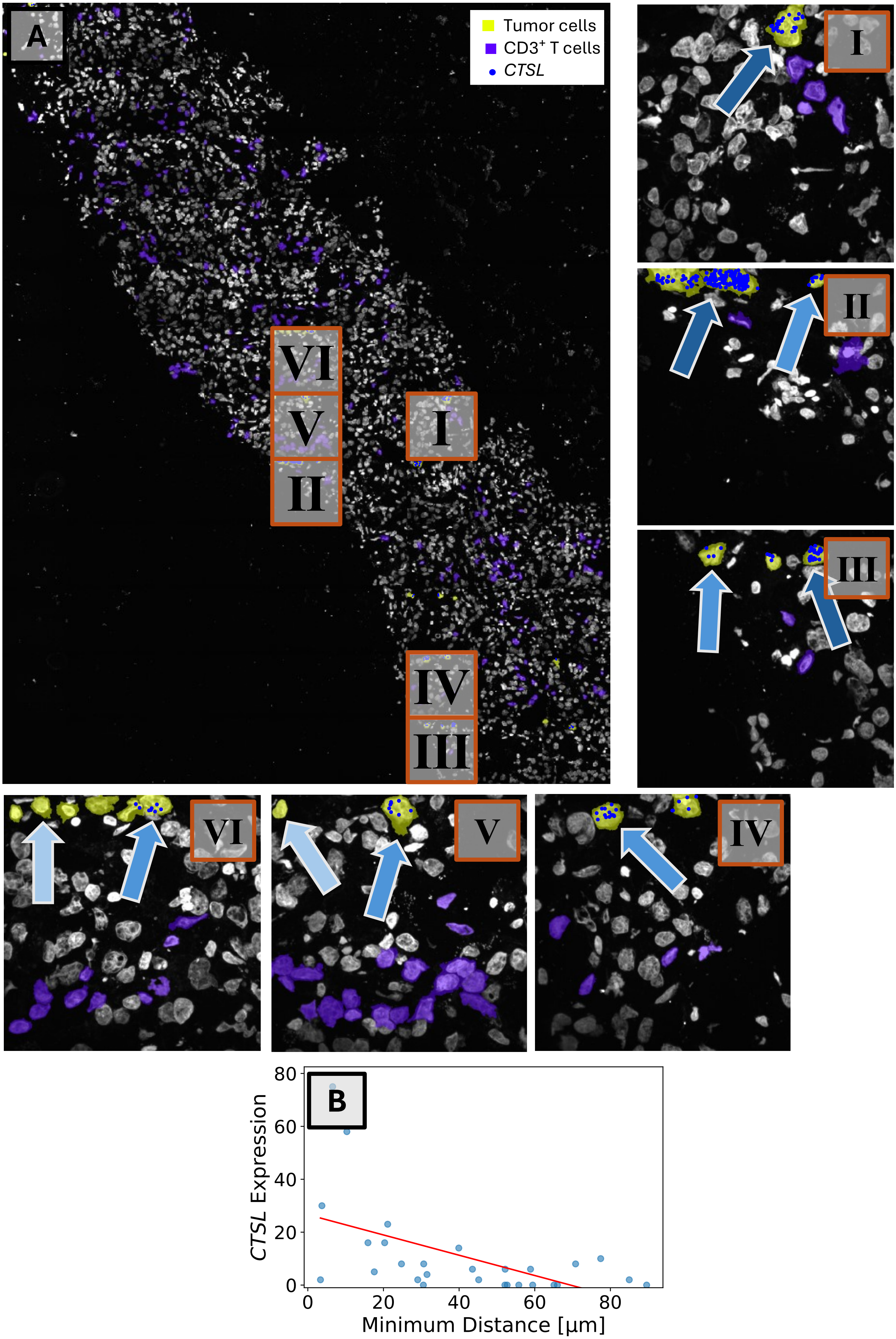

A
Tumor cells
CD3+ T cells
CTSL
VI
V
I
II
IV
III
B
CTSL Expression
Minimum Distance [µm]
0
20
40
60
80

Fig. S18. Example of proximity-dependent gene expression: *CTSL* expression in tumor cells relative to CD3$^+$ T cells in biopsy 313. (A) Sequencing read locations for *CTSL* (blue dots) are overlaid on DAPI-stained nuclei, together with segmentations of tumor cells (yellow) and CD3$^+$ T cells (magenta). Only tumor-cell and CD3$^+$ T-cell segmentations are shown. A full tissue section and representative 40× fields of view (100 × 100 µm) are displayed. Tumor cells proximal to CD3$^+$ T cells exhibit higher *CTSL* expression (solid blue arrows) compared with distal tumor cells (hollow blue arrows). **(B)** Linear dependency of *CTSL* expression in tumor cells on distance to CD3$^+$ T cells (p from regression coefficient; t-test, *statsmodels*; $q = 2 \times 10^{-2}$, slope = −0.38).

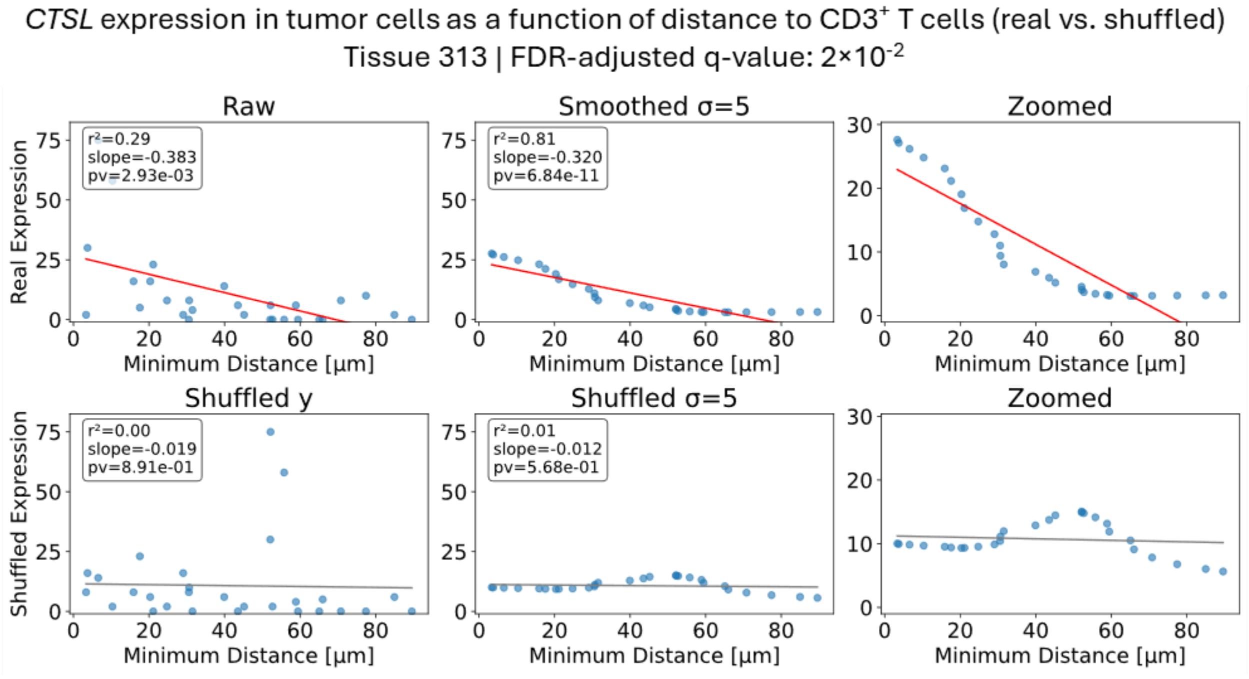

Fig. S19. *CTSL* expression in tumor cells as a function of distance to CD3+ T cells in biopsy 313. The data of Fig. S18, but with the permutation analysis as in Fig. S14. The top row demonstrates a statistically significant negative relationship between *CTSL* expression and proximity to CD3+ T cells ($q = 2 \times 10^{-2}$, slope = -0.38, smoothed $R^2 = 0.81$), which is absent in the shuffled controls (slope = -0.02, $R^2 \approx 0$).

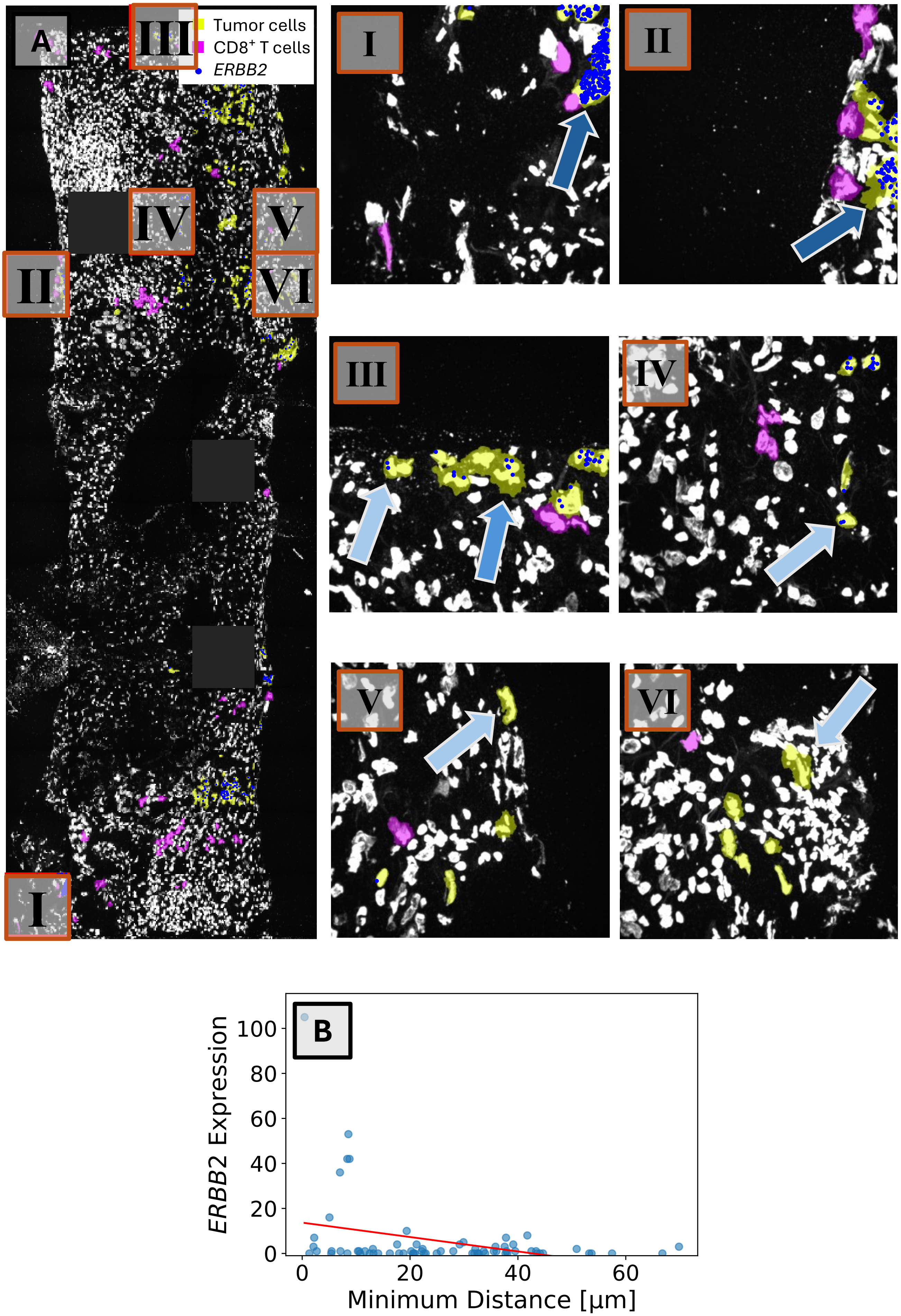

A
Tumor cells
CD8⁺ T cells
ERBB2
I
II
III
IV
V
VI
B
ERBB2 Expression
Minimum Distance [µm]
0
20
40
60
80
100

Fig. S20. Example of proximity-dependent gene expression: *ERBB2* expression in tumor cells relative to CD8$^+$ T cells in biopsy 364. (A) Sequencing read locations for *ERBB2* (blue dots) are overlaid on DAPI-stained nuclei, together with segmentations of tumor cells (yellow) and CD8$^+$ T cells (magenta). Only tumor-cell and CD8$^+$ T-cell segmentations are shown. A full tissue section and representative 40× fields of view (100 × 100 μm) are displayed. Tumor cells proximal to CD8$^+$ T cells exhibit higher *ERBB2* expression (solid blue arrows) compared with distal tumor cells (hollow blue arrows). **(B)** Linear dependency of *ERBB2* expression in tumor cells on distance to CD8$^+$ T cells (p from regression coefficient; t-test, *statsmodels*; $q = 3 \times 10^{-2}$, slope = -0.32).

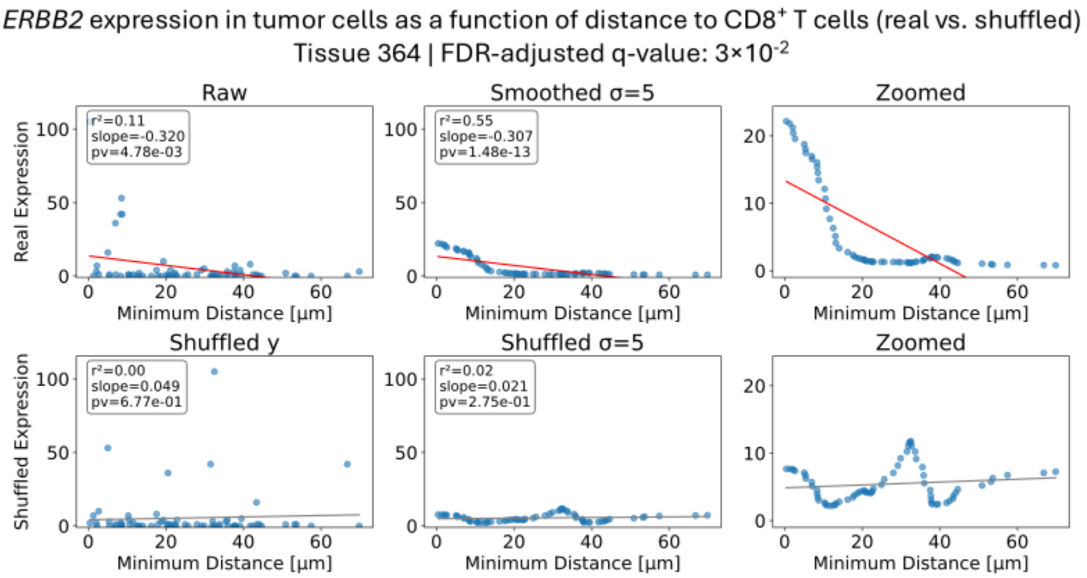

Fig. S21. *ERBB2* expression in tumor cells as a function of distance to CD8⁺ T cells in biopsy 364. The data of Fig. S20, but with the permutation analysis as in Fig. S14. The top row demonstrates a statistically significant negative relationship between *ERBB2* expression and proximity to CD8⁺ T cells ($q = 3 \times 10^{-2}$, slope = -0.32, smoothed $R^2 = 0.55$), which is absent in the shuffled controls (slope = 0.05, $R^2 \approx 0$).

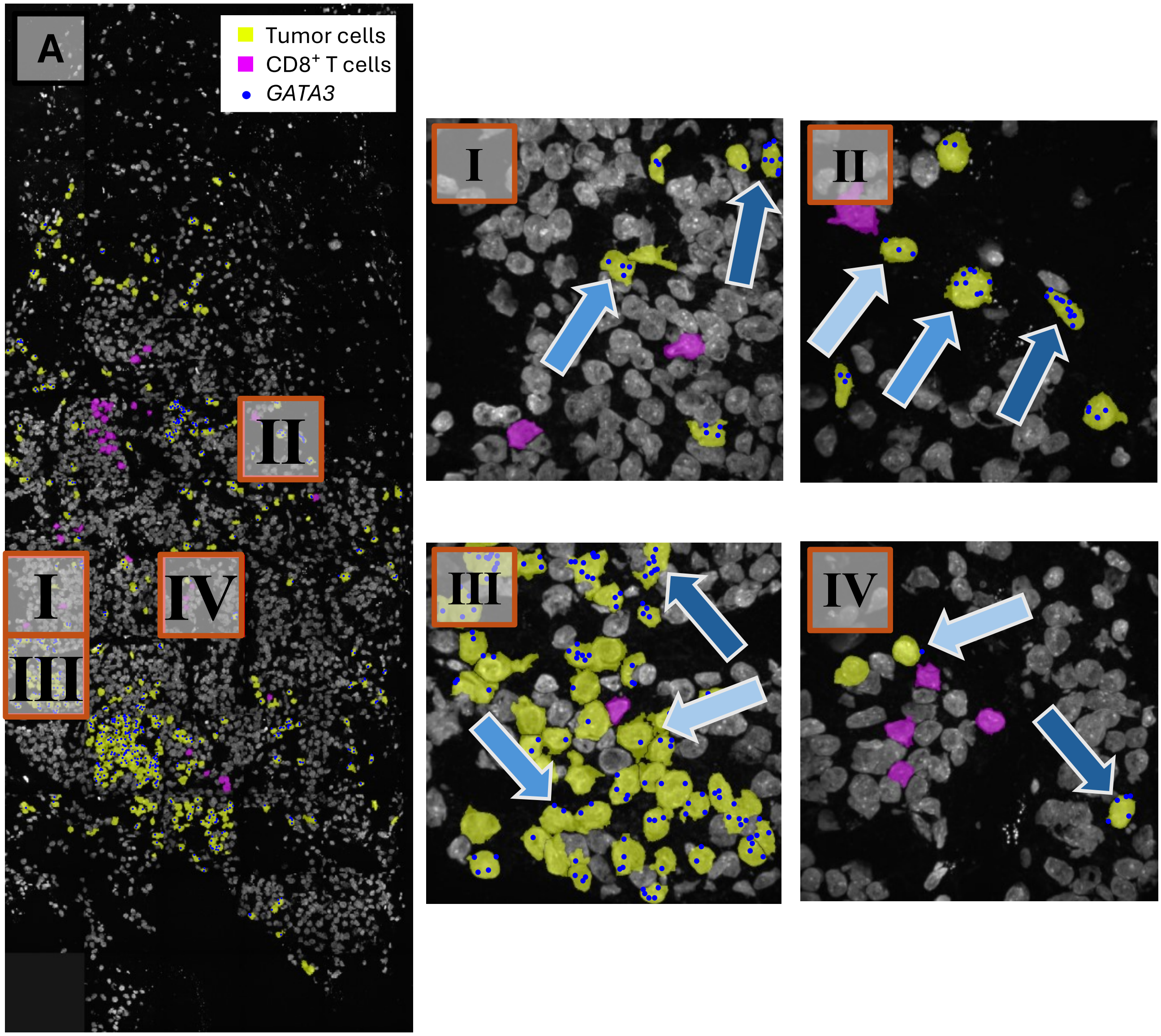
A
Tumor cells
CD8+ T cells
GATA3
I
II
III
IV

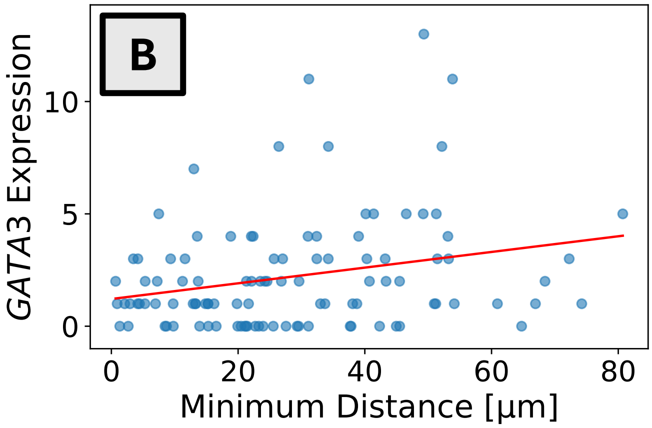
B
GATA3 Expression
Minimum Distance [μm]
0
5
10
0
20
40
60
80

Fig. S22. Example of proximity-dependent gene expression: *GATA3* expression in tumor cells relative to CD8$^+$ T cells in biopsy 982. (A) Sequencing read locations for *GATA3* (blue dots) are overlaid on DAPI-stained nuclei, together with segmentations of tumor cells (yellow) and CD8$^+$ T cells (magenta). Only tumor-cell and CD8$^+$ T-cell segmentations are shown. A full tissue section and representative 40× fields of view (100 × 100 μm) are displayed. Tumor cells distal to CD8$^+$ T cells exhibit higher *GATA3* expression (solid blue arrows) compared with proximal tumor cells (hollow blue arrows). **(B)** Linear dependency of *GATA3* expression in tumor cells on distance to CD8$^+$ T cells (p from regression coefficient; t-test, *statsmodels*; $q = 4 \times 10^{-2}$, slope = 0.03).

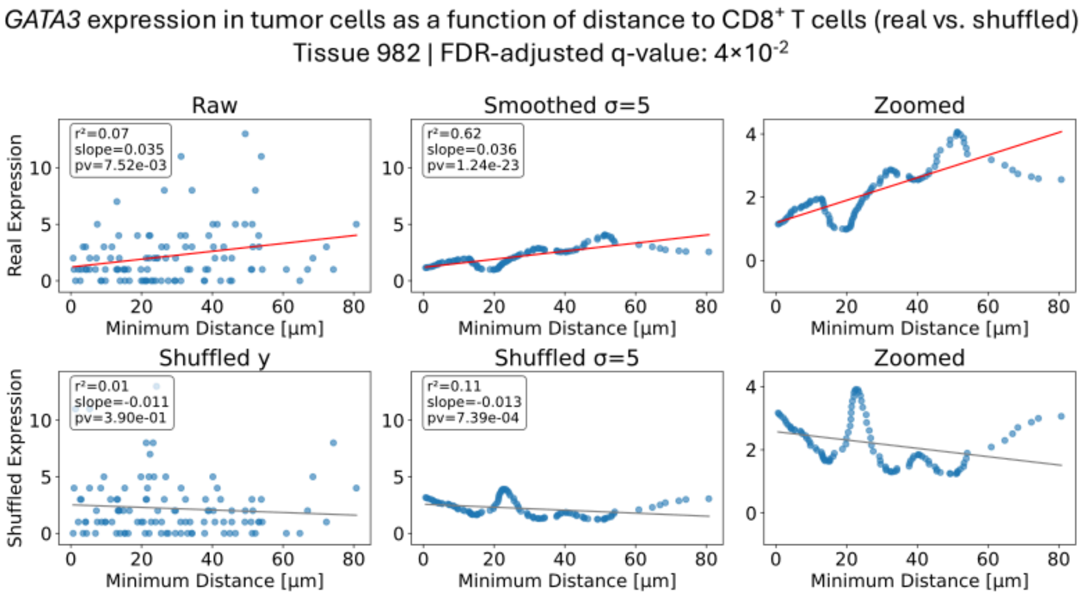

Fig. S23. ***GATA3* expression in tumor cells as a function of distance to CD8⁺ T cells in biopsy 982.** The data of Fig. S22, but with the permutation analysis as in Fig. S14. The top row demonstrates a statistically significant positive relationship between *GATA3* expression and proximity to CD8⁺ T cells ($q = 4 \times 10^{-2}$, slope = 0.03, smoothed $R^2 = 0.62$), which is absent in the shuffled controls (slope = -0.01, $R^2 \approx 0.01$).

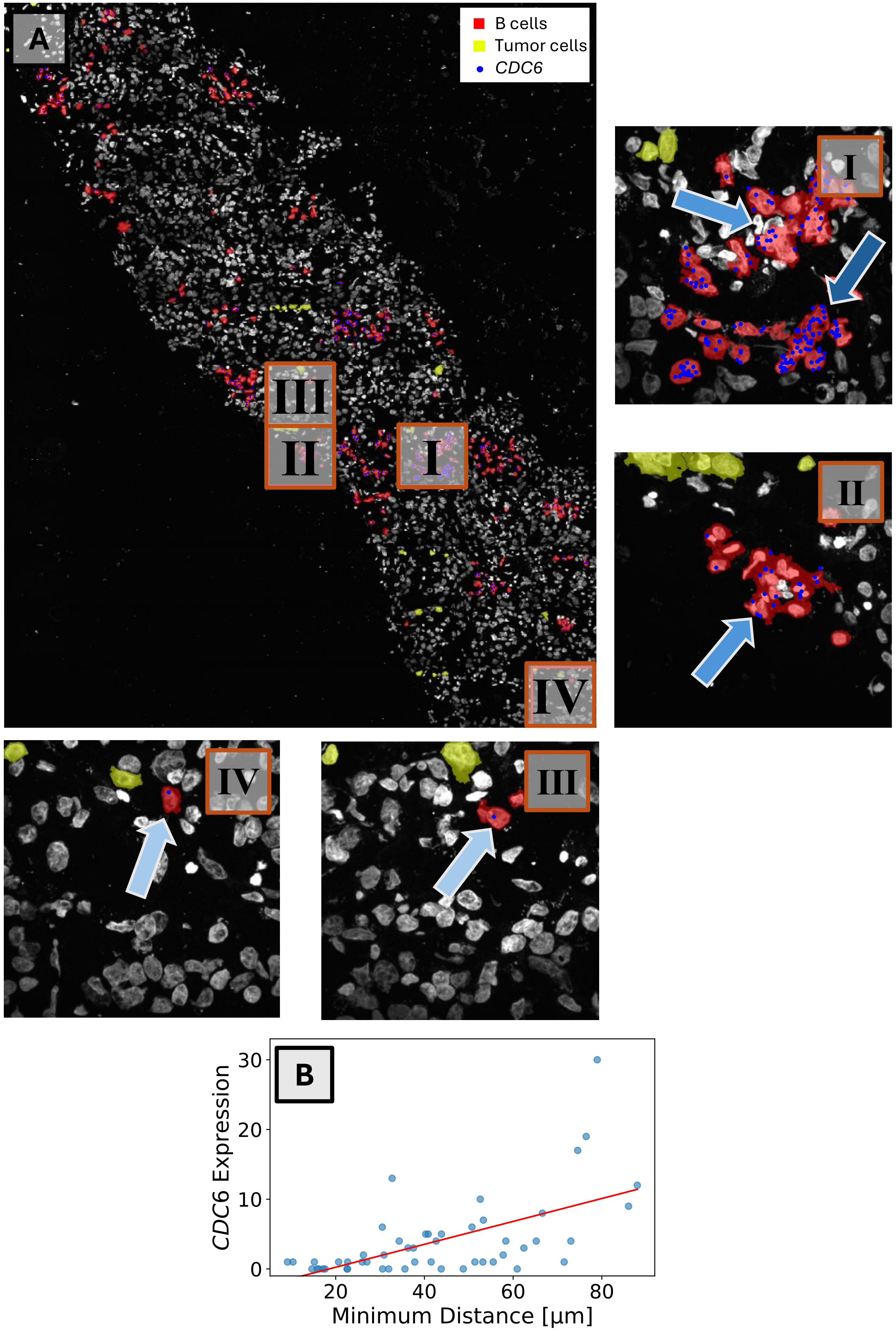

A
B cells
Tumor cells
CDC6
I
II
III
IV
B
CDC6 Expression
Minimum Distance [µm]
0
10
20
30
20
40
60
80

Fig. S24. Example of proximity-dependent gene expression: *CDC6* expression in B cells relative to tumor cells in biopsy 313. (A) Sequencing read locations for *CDC6* (blue dots) are overlaid on DAPI-stained nuclei, together with segmentations of B cells (red) and tumor cells (yellow). Only B-cell and tumor-cell segmentations are shown. A full tissue section and representative 40× fields of view (100 × 100 µm) are displayed. B cells distal to tumor cells exhibit higher *CDC6* expression (solid blue arrows) compared with proximal B cells (hollow blue arrows). **(B)** Linear dependency of *CDC6* expression in B cells on distance to tumor cells (p from regression coefficient; t-test, *statsmodels*; $q = 6 \times 10^{-5}$, slope = 0.16).

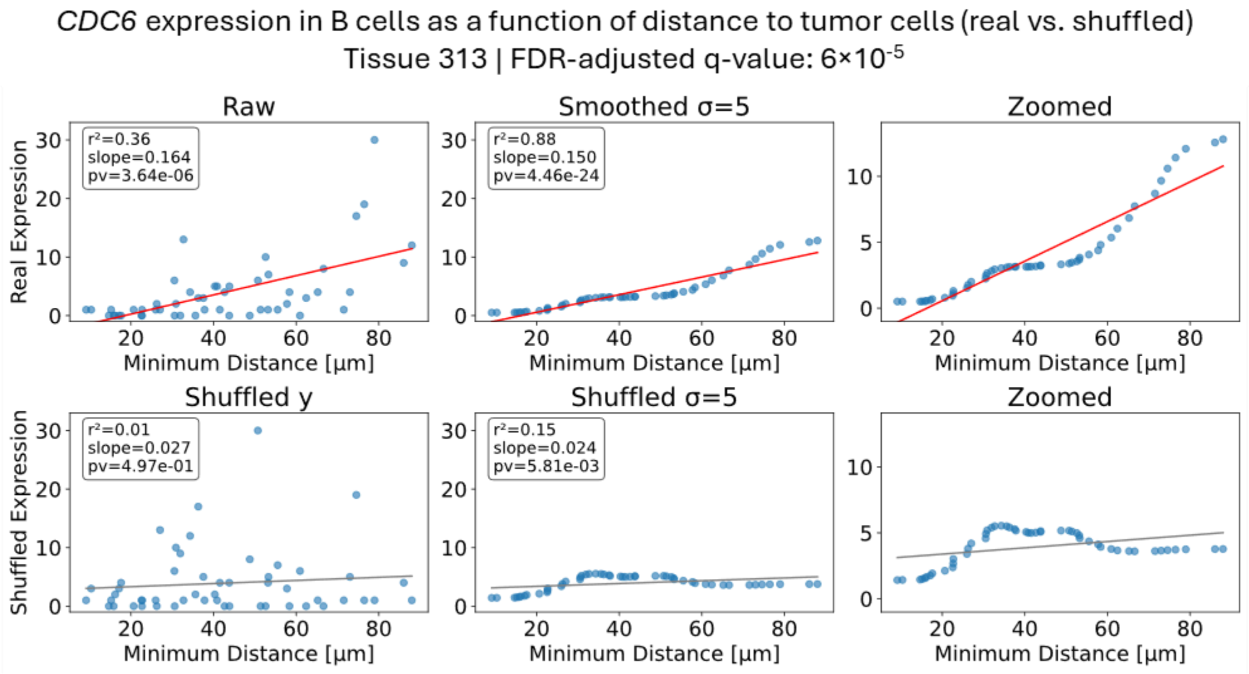

Fig. S25. *CDC6* expression in B cells as a function of distance to tumor cells in biopsy 313. The data of Fig. S24, but with the permutation analysis as in Fig. S14. The top row demonstrates a statistically significant positive relationship between *CDC6* expression and proximity to tumor cells ($q = 6 \times 10^{-5}$, slope = 0.16, smoothed $R^2 = 0.88$), which is absent in the shuffled controls (slope = 0.03, $R^2 \approx 0.01$).

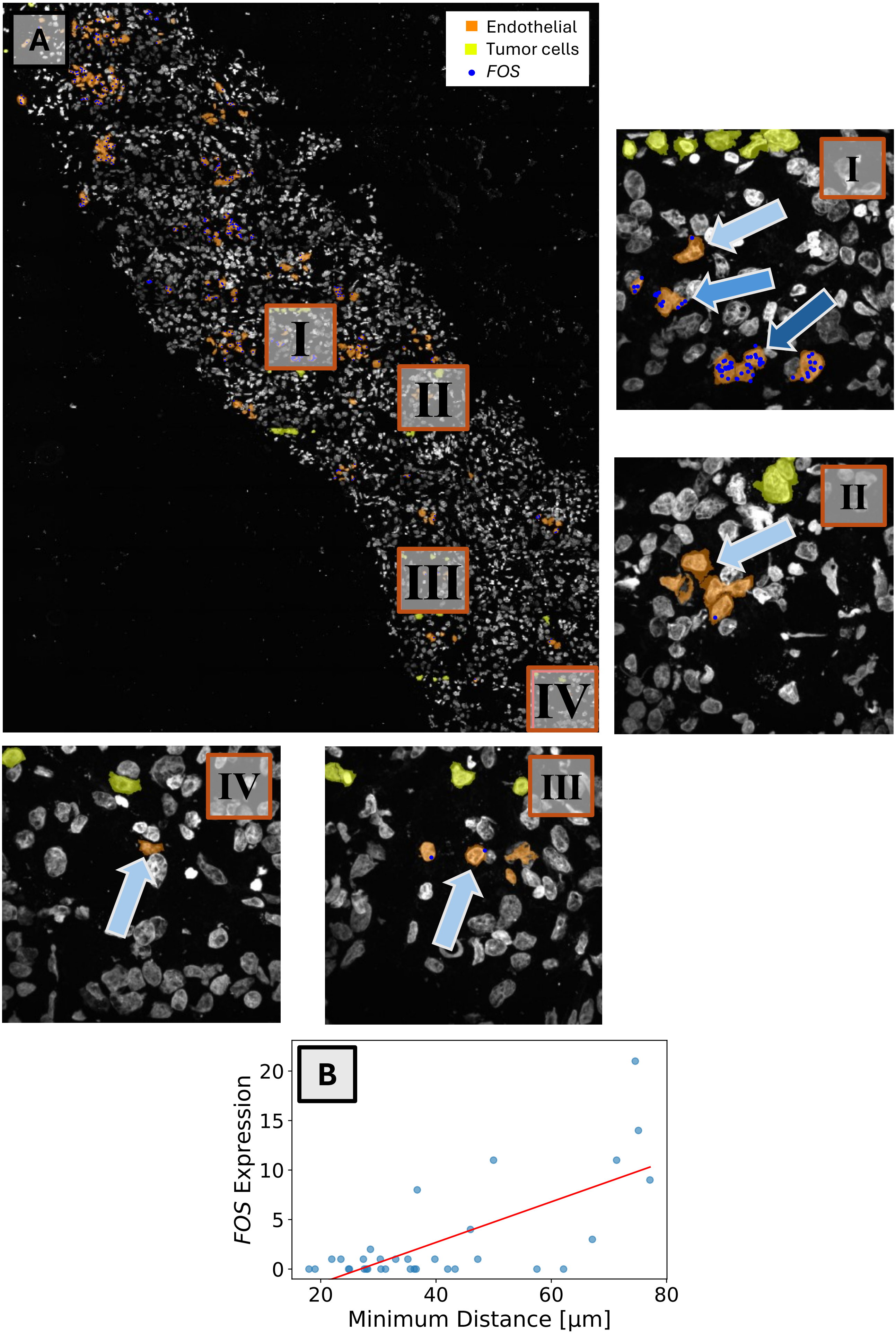

A
Endothelial
Tumor cells
FOS
I
II
III
IV
B
FOS Expression
Minimum Distance [µm]

Fig. S26. Example of proximity-dependent gene expression: *FOS* expression in endothelial cells relative to tumor cells in biopsy 313. (A) Sequencing read locations for *FOS* (blue dots) are overlaid on DAPI-stained nuclei, together with segmentations of endothelial cells (orange) and tumor cells (yellow). Only endothelial-cell and tumor-cell segmentations are shown. A full tissue section and representative 40× fields of view (100 × 100 µm) are displayed. Endothelial cells distal to tumor cells exhibit higher *FOS* expression (solid blue arrows) compared with proximal endothelial cells (hollow blue arrows). **(B)** Linear dependency of *FOS* expression in endothelial cells on distance to tumor cells (p from regression coefficient; t-test, *statsmodels*; $q = 7 \times 10^{-5}$, slope = 0.21).

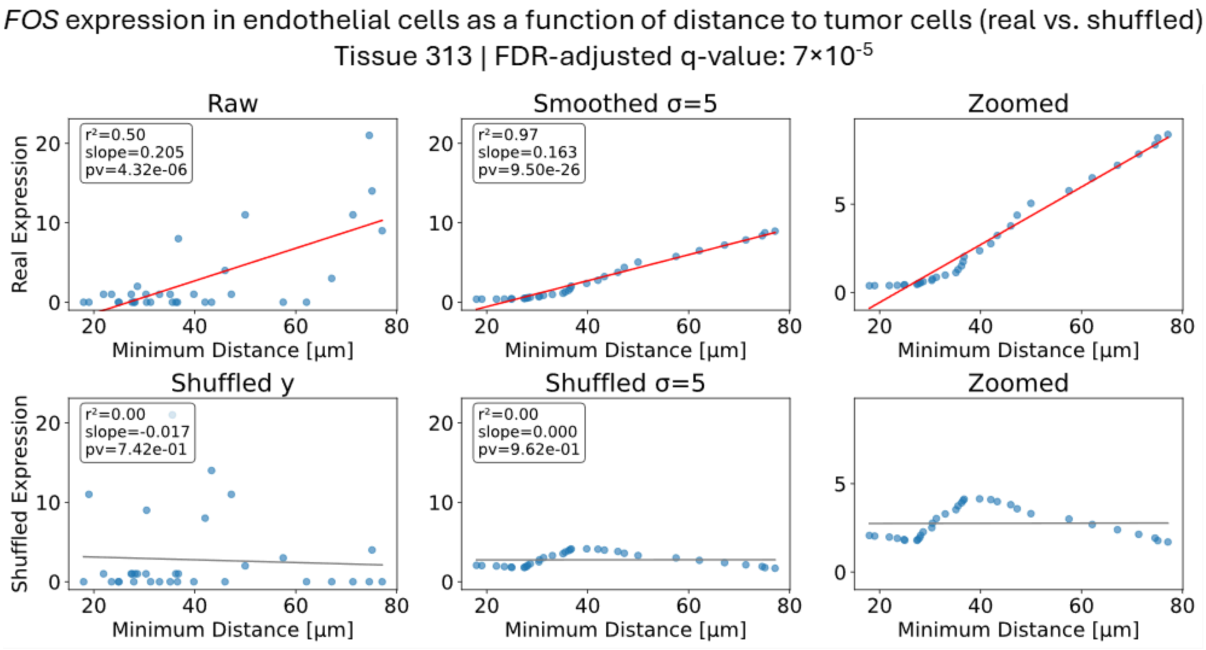

Fig. S27. *FOS* expression in endothelial cells as a function of distance to tumor cells in biopsy 313. The data of Fig. S26, but with the permutation analysis as in Fig. S14. The top row demonstrates a statistically significant positive relationship between *FOS* expression and proximity to tumor cells ($q = 7 \times 10^{-5}$, slope = 0.21, smoothed $R^2 = 0.97$), which is absent in the shuffled controls (slope = -0.02, $R^2 \approx 0$).

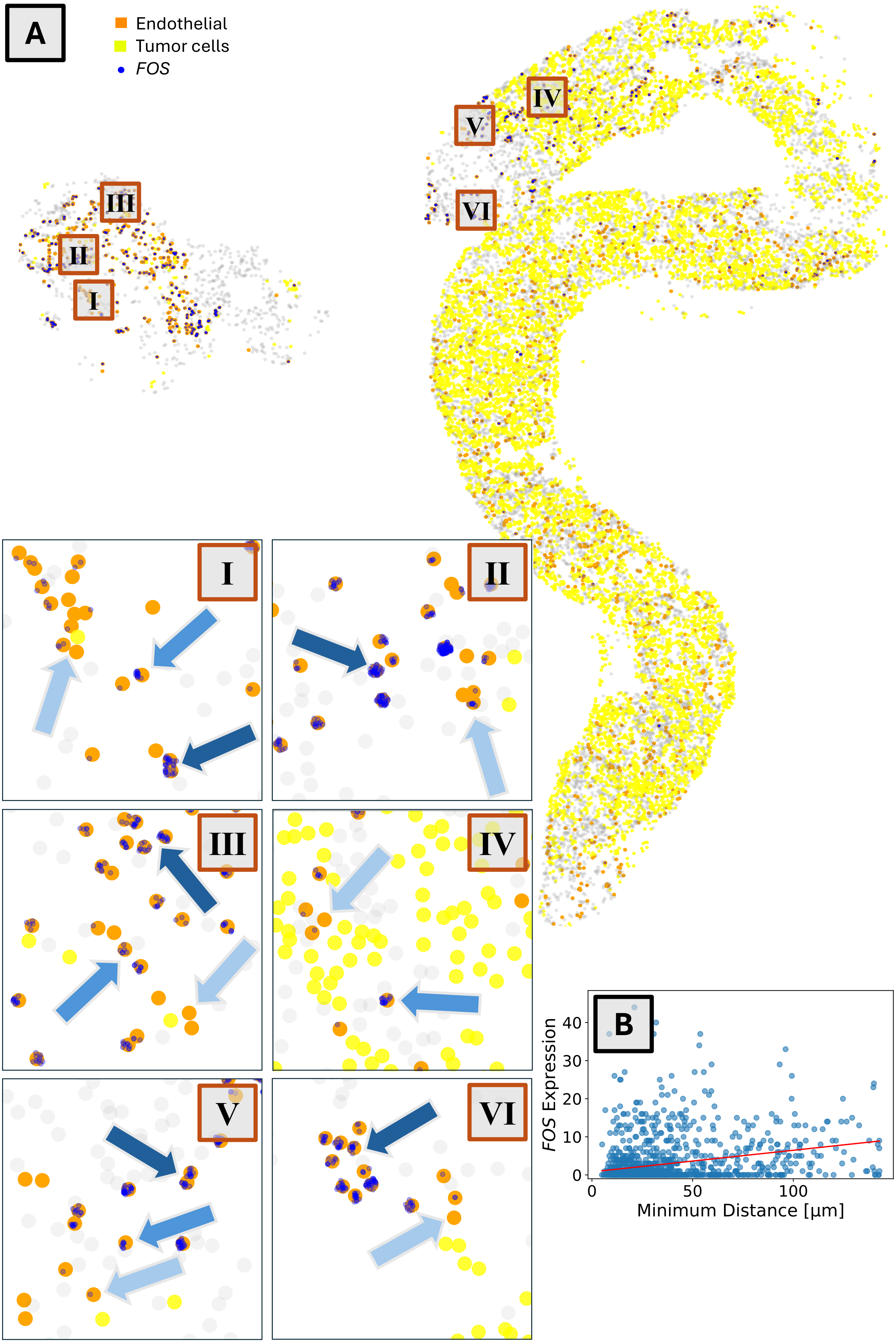
A
Endothelial
Tumor cells
FOS
I
II
III
IV
V
VI
B
FOS Expression
Minimum Distance [µm]
0
10
20
30
40
0
50
100

Fig. S28. Example of proximity-dependent gene expression using MERFISH data: *FOS* expression in endothelial cells relative to tumor cells in biopsy 514. **(A)** Sequencing read locations for *FOS* (blue dots) are overlaid on endothelial cells (orange) and tumor cells (yellow). Only endothelial-cell and tumor-cell segmentations are shown. Each subpanel measures 200 × 200 µm, and the overall imaged tissue is approximately 5400 × 6200 µm. Endothelial cells distal to tumor cells exhibit higher *FOS* expression (solid blue arrows) compared with proximal endothelial cells (hollow blue arrows). **(B)** Linear dependency of *FOS* expression in endothelial cells on distance to tumor cells (p from regression coefficient; t-test, *statsmodels*; $q = 9 \times 10^{-24}$, slope = 0.06).

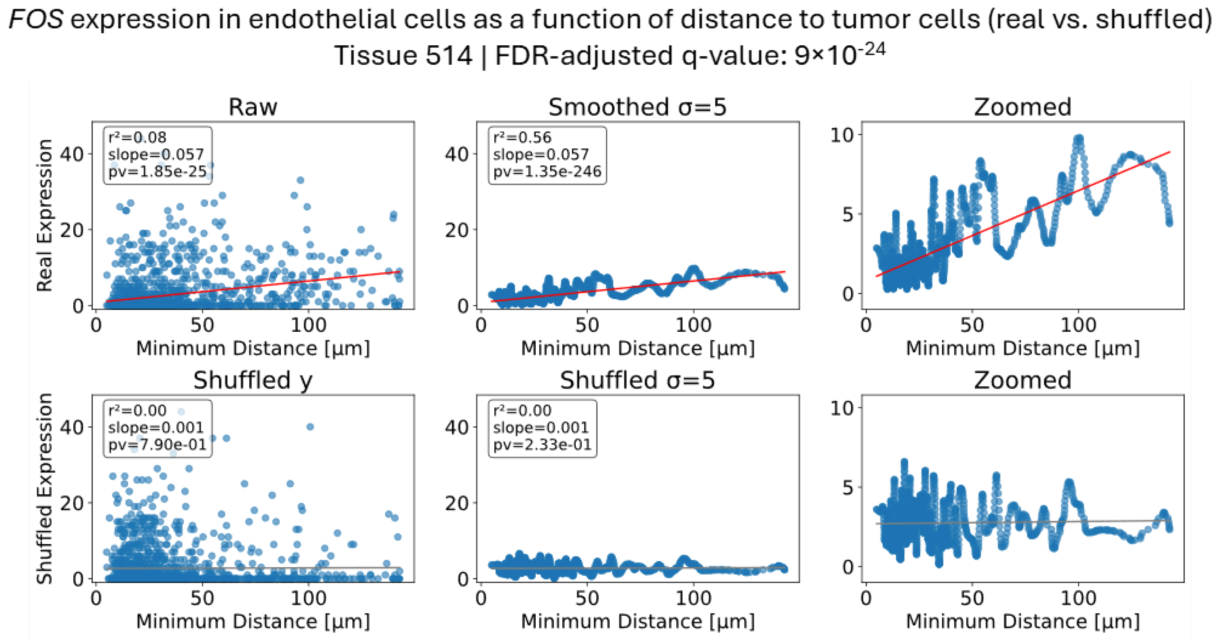

Fig. S29. *FOS* expression in endothelial cells as a function of distance to tumor cells in biopsy 514. The data in this figure is of Fig. S28, but with the permutation analysis as in Fig. S14. The top row demonstrates a statistically significant positive relationship between *FOS* expression and proximity to tumor cells ($q = 9 \times 10^{-24}$, slope = 0.06, smoothed $R^2 = 0.56$), which is absent in the shuffled controls (slope ≈ 0, $R^2 \approx 0$).

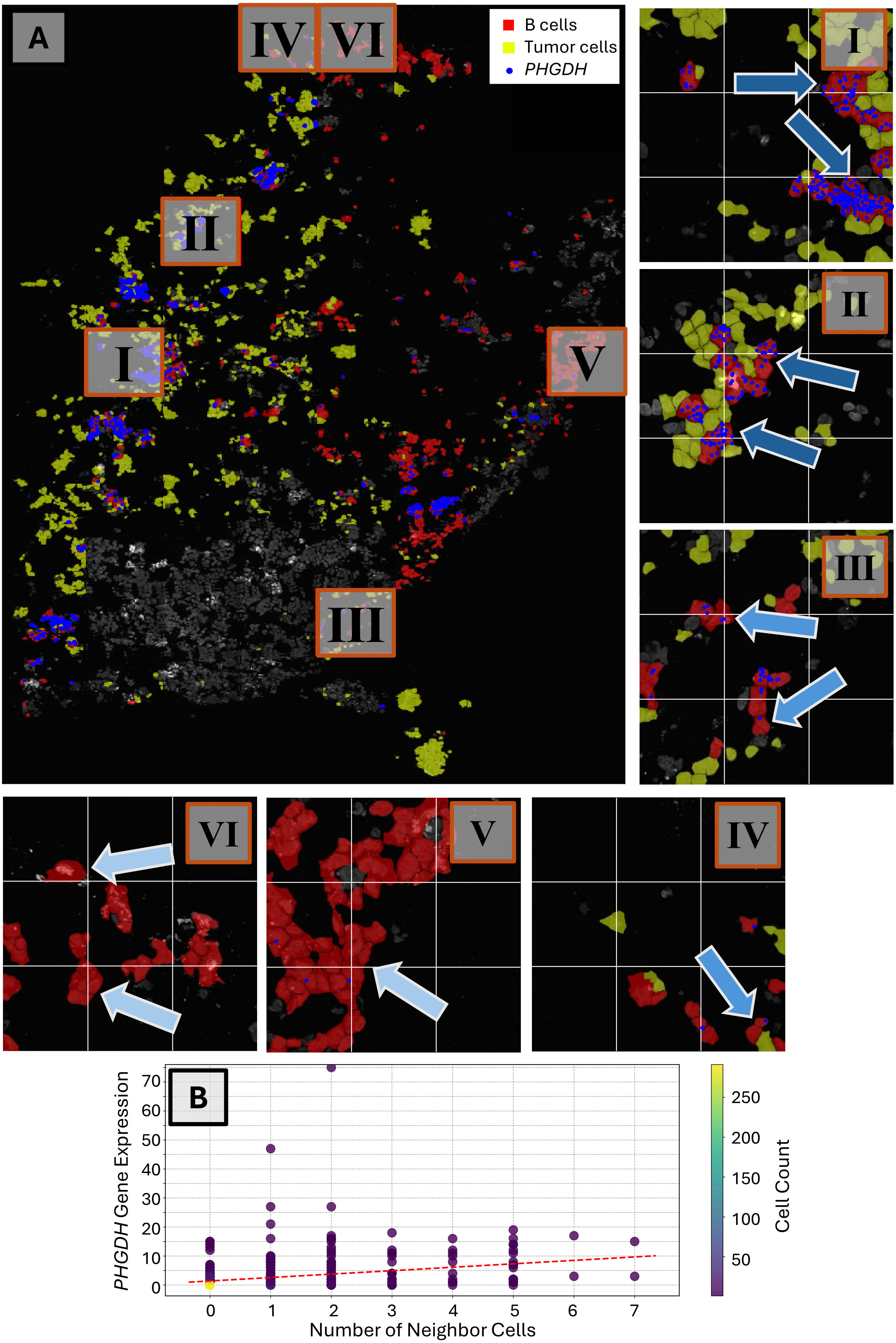
A
IV
VI
B cells
Tumor cells
PHGDH
I
II
III
V
B
PHGDH Gene Expression
Number of Neighbor Cells
Cell Count
0
10
20
30
40
50
60
70
1
2
3
4
5
6
7
50
100
150
200
250

Fig. S30. Example of neighboring-dependent gene expression: *PHGDH* expression in B cells relative to the number of neighboring tumor cells in biopsy 59. (A) Sequencing read locations for *PHGDH* (blue dots) are overlaid on DAPI-stained nuclei, together with segmentations of B cells (red) and tumor cells (yellow). Only B-cell and tumor-cell segmentations are shown. A full tissue section and representative 40× fields of view (100 × 100 µm) are displayed. B cells with fewer neighboring tumor cells show lower *PHGDH* expression (hollow blue arrows) compared with B cells surrounded by many tumor cells, which show higher *PHGDH* expression (solid blue arrows). **(B)** Increasing linear dependency of *PHGDH* expression in B cells on the number of neighboring tumor cells (p from regression coefficient; t-test, *statsmodels*; $q = 2 \times 10^{-19}$, slope = 1.18).

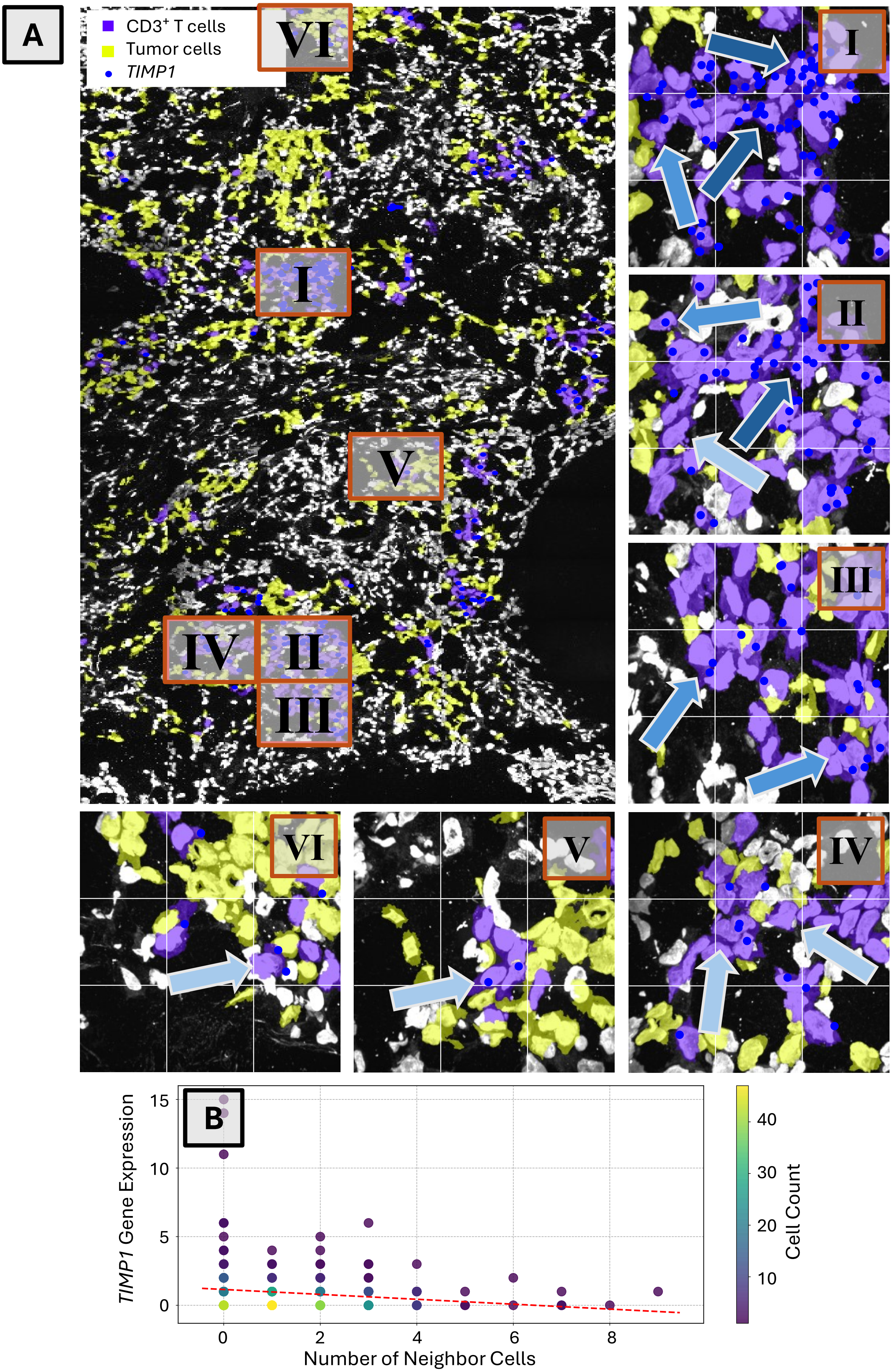
A
CD3+ T cells
Tumor cells
TIMP1
VI
I
V
IV
II
III
B
TIMP1 Gene Expression
Number of Neighbor Cells
Cell Count
0
2
4
6
8
5
10
15
20
30
40

Fig. S31. Example of neighboring-dependent gene expression: *TIMP1* expression in CD3$^+$ T cells relative to the number of neighboring tumor cells in biopsy 100. (A) Sequencing read locations for *TIMP1* (blue dots) are overlaid on DAPI-stained nuclei, together with segmentations of CD3$^+$ T cells (magenta) and tumor cells (yellow). Only CD3$^+$ T-cell and tumor-cell segmentations are shown. A full tissue section and representative 40× fields of view (100 × 100 µm) are displayed. CD3$^+$ T cells with fewer neighboring tumor cells show higher *TIMP1* expression (solid blue arrows) compared with CD3$^+$ T cells surrounded by many tumor cells, which show lower *TIMP1* expression (hollow blue arrows). **(B)** Negative linear dependency of *TIMP1* expression in CD3$^+$ T cells on the number of neighboring tumor cells (p from regression coefficient; t-test, *statsmodels*; $q = 2 \times 10^{-2}$, slope = −0.18).

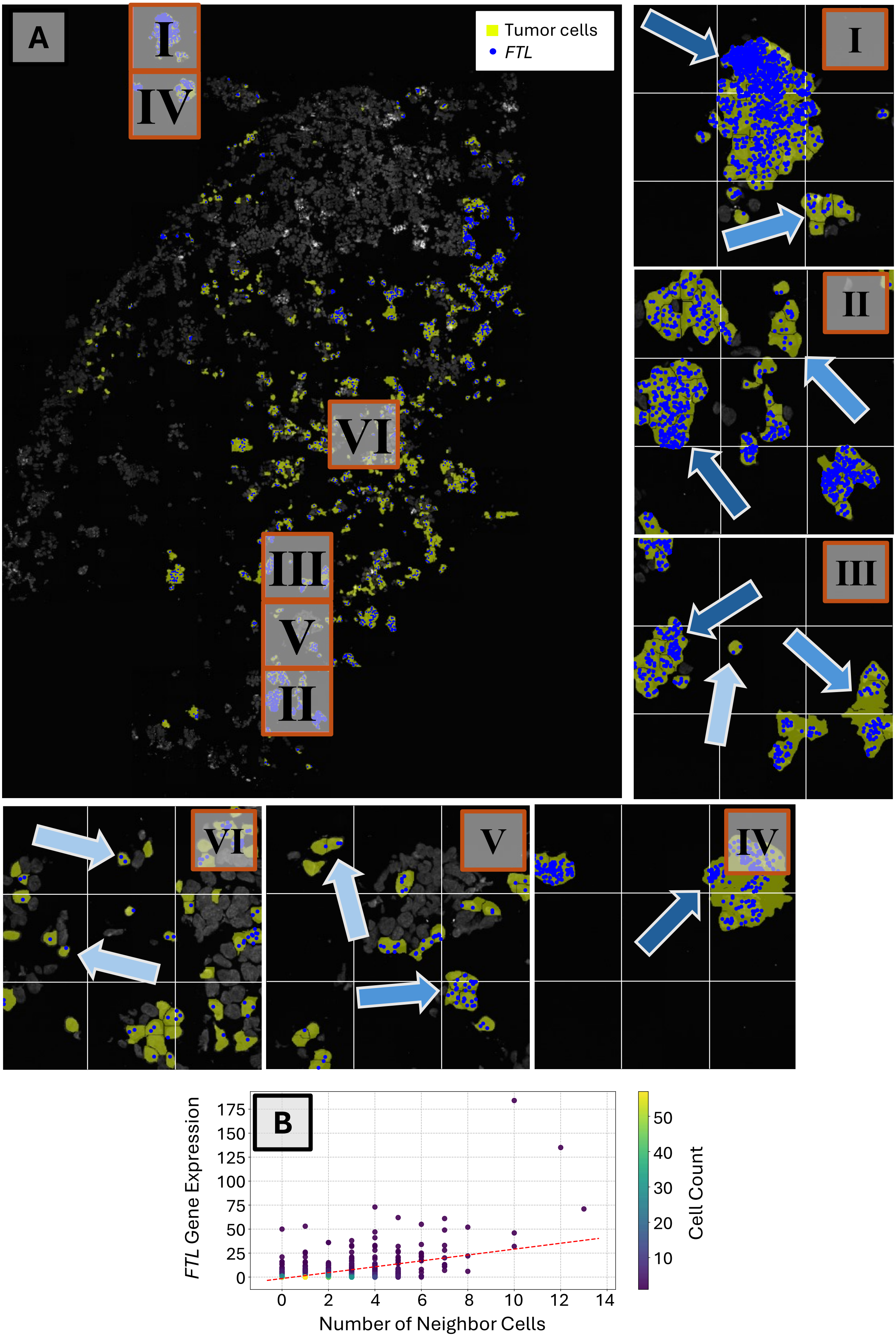
A
I
IV
VI
III
V
II
Tumor cells
FTL
B
FTL Gene Expression
Number of Neighbor Cells
Cell Count
0
25
50
75
100
125
150
175
0
2
4
6
8
10
12
14
10
20
30
40
50

Fig. S32. Example of self neighboring-dependent gene expression: *FTL* expression in tumor cells relative to the number of neighboring tumor cells in biopsy 59. (A) Sequencing read locations for *FTL* (blue dots) are overlaid on DAPI-stained nuclei, together with segmentations of tumor cells (yellow). Only tumor-cell segmentations are shown. A full tissue section and representative 40× fields of view (100 × 100 µm) are displayed. Tumor cells with fewer neighboring tumor cells show lower *FTL* expression (hollow blue arrows) compared with tumor cells surrounded by many tumor cells, which show higher *FTL* expression (solid blue arrows). **(B)** Positive linear dependency of *FTL* expression in tumor cells on the number of neighboring tumor cells (p from regression coefficient; t-test, *statsmodels*; $q = 6 \times 10^{-24}$, slope = 2.89).

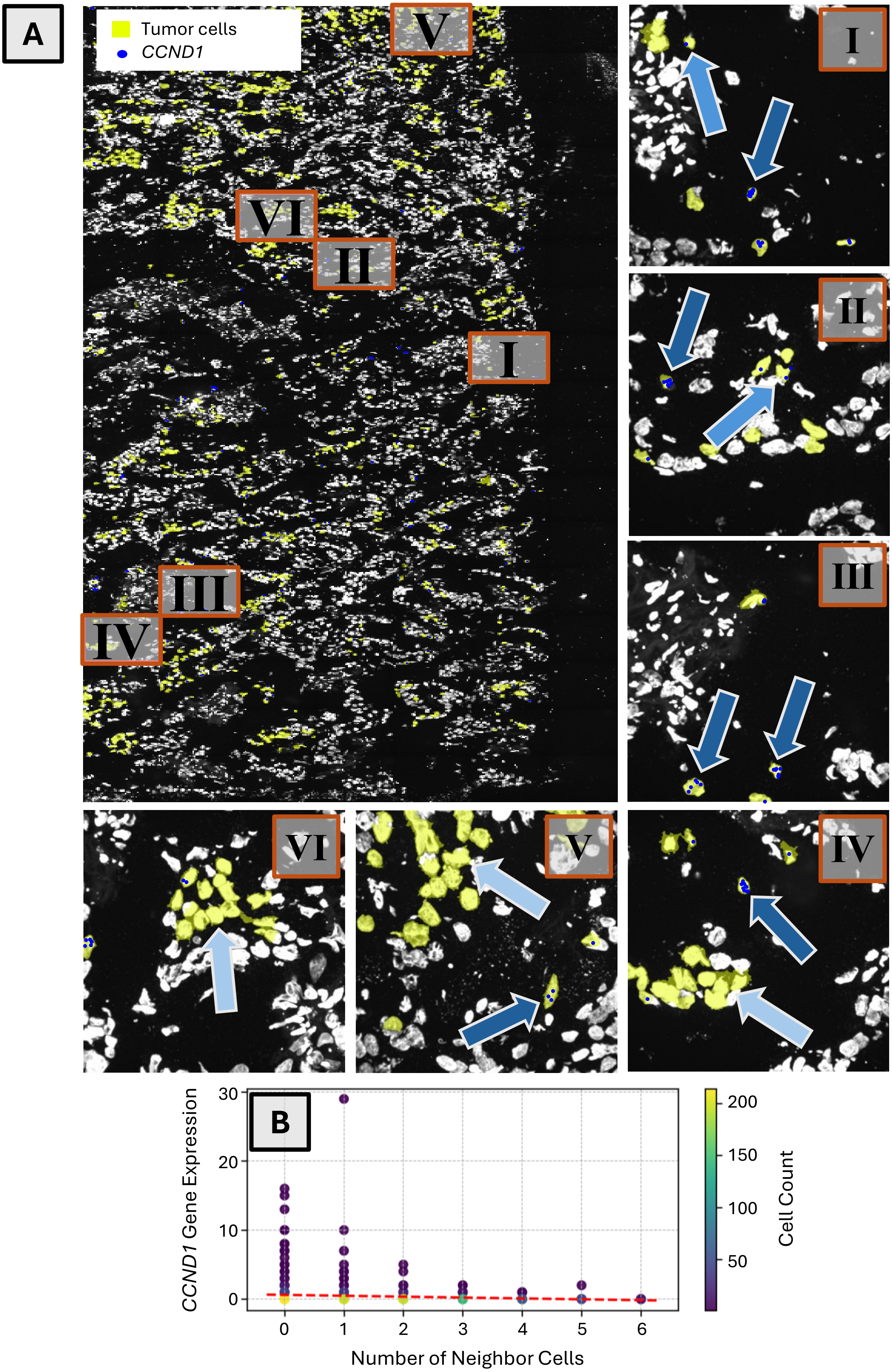

A
Tumor cells
CCND1
V
VI
II
I
III
IV
B
CCND1 Gene Expression
Number of Neighbor Cells
Cell Count
0
10
20
30
0
1
2
3
4
5
6
50
100
150
200

Fig. S33. Example of self neighboring-dependent gene expression: *CCND1* expression in tumor cells relative to the number of neighboring tumor cells in biopsy 330. (A) Sequencing read locations for *CCND1* (blue dots) are overlaid on DAPI-stained nuclei, together with segmentations of tumor cells (yellow). Only tumor-cell segmentations are shown. A full tissue section and representative 40× fields of view (100 × 100 μm) are displayed. Tumor cells with fewer neighboring tumor cells show higher *CCND1* expression (solid blue arrows) compared with tumor cells surrounded by many tumor cells, which show lower *CCND1* expression (hollow blue arrows). **(B)** Negative linear dependency of *CCND1* expression in tumor cells on the number of neighboring tumor cells (p from regression coefficient; t-test, *statsmodels*; $q = 6 \times 10^{-7}$, slope = −0.13).

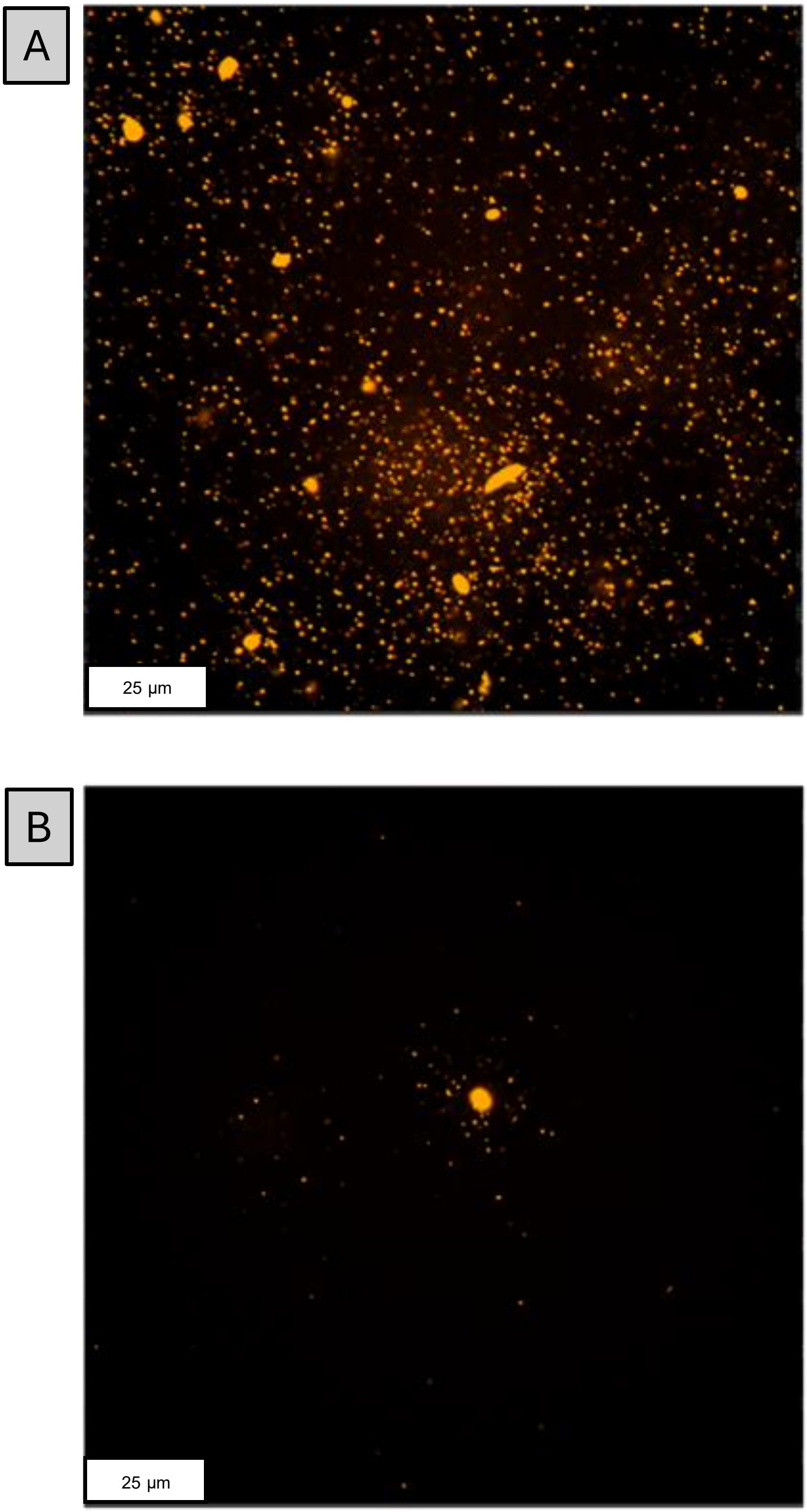
A
25 µm
B
25 µm

Fig. S34. Validation of 16S rRNA ExSeq detection in bacteria-spiked hydrogels. Validation experiments in hydrogels spiked with serial dilutions of *E. coli* confirmed that the number of detected 16S rRNA molecules correlated with bacterial concentration. ExSeq of bacteria 16S ribosomal RNA (rRNA) visualized as yellow spots. **(A)** a hydrogel with high concentration of bacteria. **(B)** hydrogel with concentration of bacteria which is 100 fold lower compared to (A). The small round points correspond to rRNA molecules. Using higher magnification, the large spots (several can be seen in (A) and one in (B)) were validated to be in the shape of individual E. coli, as expected from autofluorescence of the bacteria cell bodies (but note that not all the bacteria cells had autofluorescence).

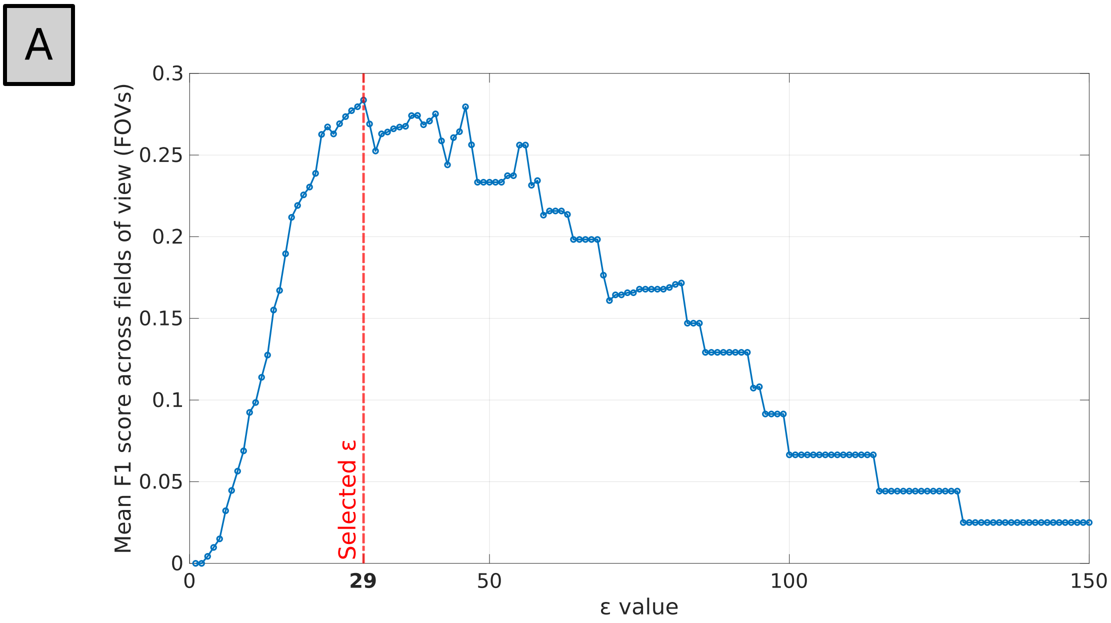
A
Mean F1 score across fields of view (FOVs)
Selected ε
ε value
0.3
0.25
0.2
0.15
0.1
0.05
0
0
29
50
100
150

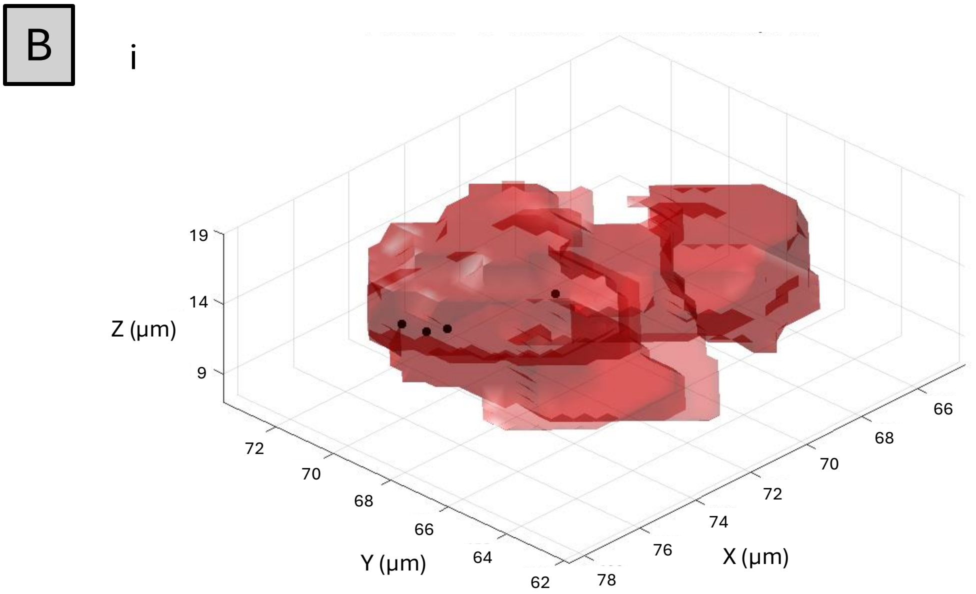
B
i
Z (μm)
Y (μm)
X (μm)
19
14
9
72
70
68
66
64
62
66
68
70
72
74
76
78

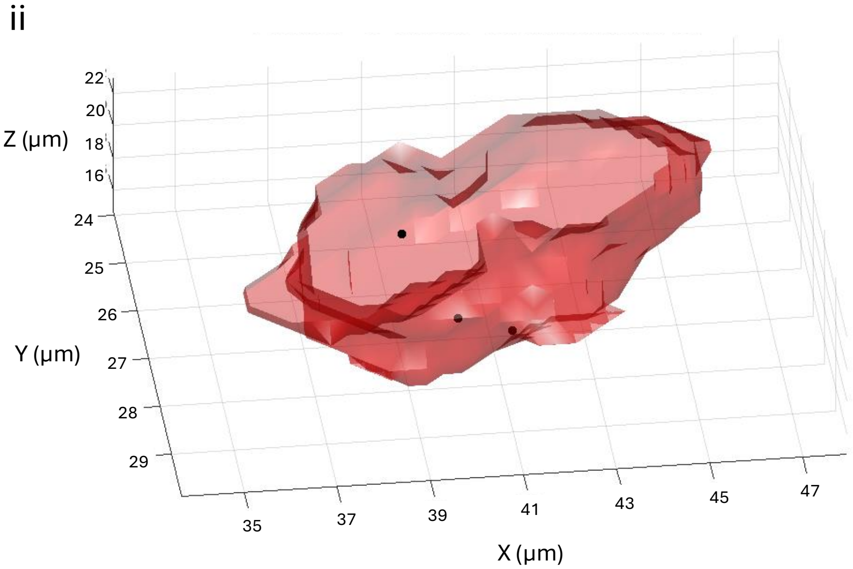
ii
Z (μm)
Y (μm)
X (μm)
22
20
18
16
24
25
26
27
28
29
35
37
39
41
43
45
47

Fig. S35. Detection of spatially localized 16S bacterial transcripts in biopsy 58 using ExSeq. (A) Mean F_1 score across fields of view (FOVs) as a function of the DBSCAN algorithm ε parameter (see Methods). The three-dimensional positions of bacterial transcripts were clustered using DBSCAN across a range of neighborhood radii (ε = 1–150; *min_samples* = 3). Precision (fraction of permutation-validated significant clusters among all detected clusters), recall (fraction of transcripts included in clusters), and F_1 score (harmonic mean of precision and recall) were calculated per FOV and summarized across ε values. The ε value that maximized performance was consistently ε ≈ 29, yielding the highest mean F_1 scores across all FOVs. **(B)** Zoomed 3D visualization of two segmented B cells (i–ii) containing five and three 16S bacterial transcripts, respectively (black dots), all belonging to significant bacterial clusters. The 16S transcripts are clearly localized within the segmented cells.

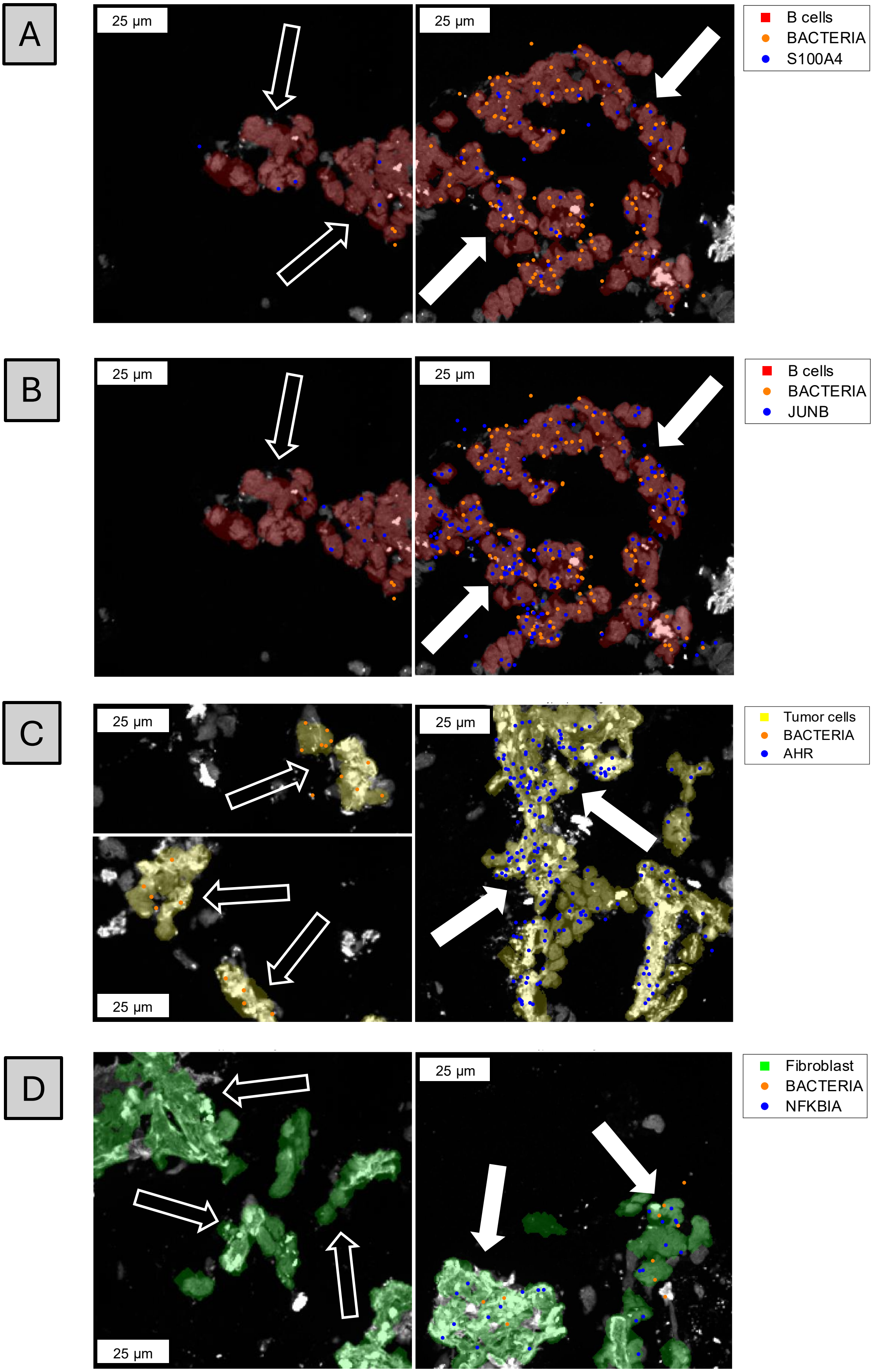
A
25 µm
25 µm
B cells
BACTERIA
S100A4
B
25 µm
25 µm
B cells
BACTERIA
JUNB
C
25 µm
25 µm
25 µm
Tumor cells
BACTERIA
AHR
D
25 µm
25 µm
Fibroblast
BACTERIA
NFKBIA

Fig. S36. Differential expression of host genes in specific cell types associated with bacterial proximity in biopsy 58. For each cell type, gene expression was compared between cells containing bacterial transcripts and cells distant from bacteria (>1 µm; see Methods). Shown are four genes that reached statistical significance (DESeq2; $q < 0.05$) and were expressed in at least 10% of cells of the respective type in the scRNA-seq dataset: **(A)** *S100A4* in B cells, higher in bacteria-associated cells (solid white arrows) compared with non-bacteria-associated cells (hollow arrows; $q < 0.03$); **(B)** *JUNB* in B cells, higher in bacteria-associated cells ($q < 0.003$); **(C)** *AHR* in tumor cells, lower in bacteria-associated cells (solid white arrows) compared with non-bacteria-associated cells (hollow arrows; $q < 0.05$); and **(D)** *NFKBIA* in fibroblasts, higher in bacteria-associated cells (solid white arrows) compared with non-bacteria-associated cells (hollow arrows; $q < 0.05$).

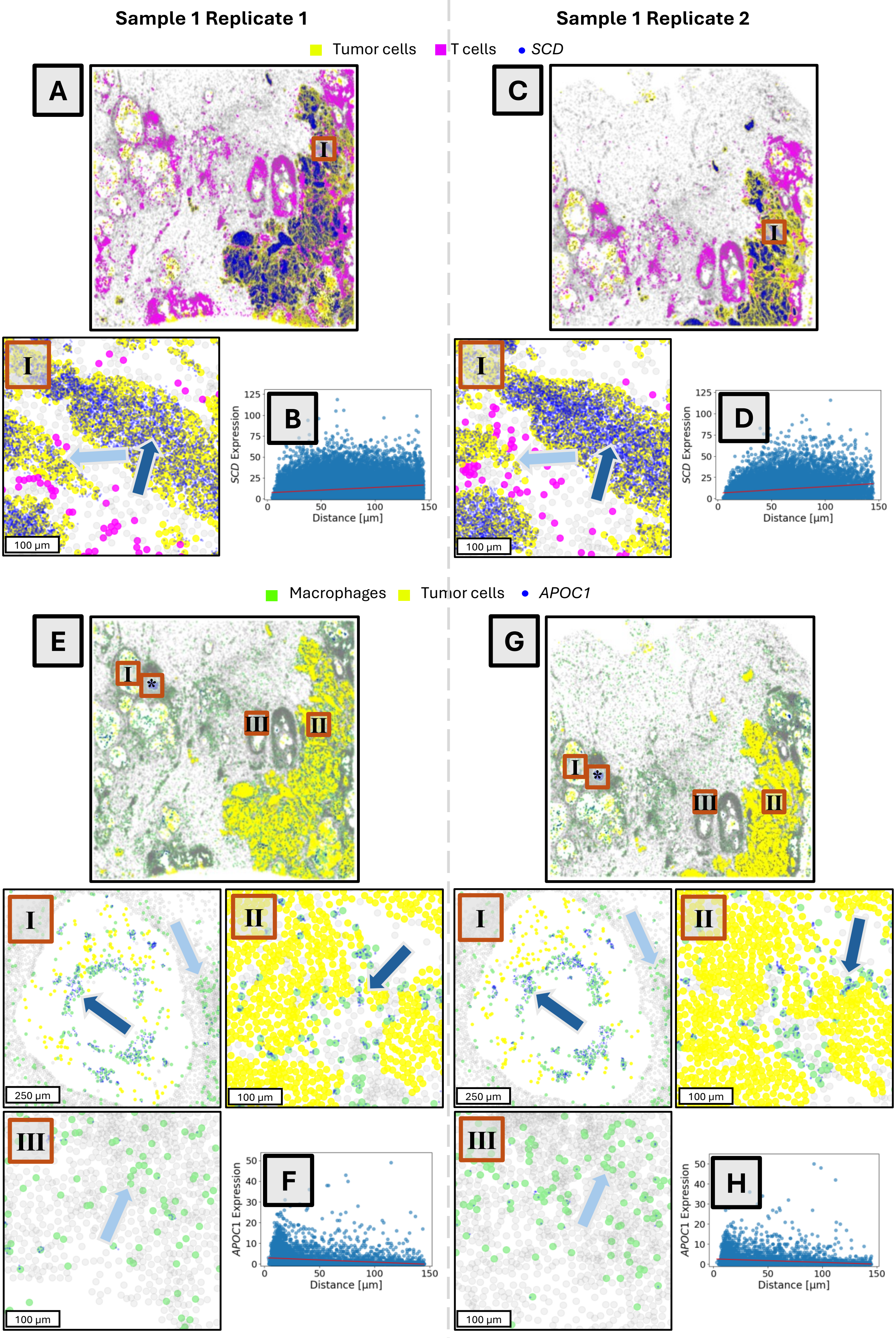
Sample 1 Replicate 1
Sample 1 Replicate 2
Tumor cells
T cells
SCD
A
C
I
B
D
SCD Expression
Distance [µm]
100 µm
Macrophages
Tumor cells
APOC1
E
G
II
III
F
H
APOC1 Expression
250 µm

Fig. S37. Examples of proximity-dependent gene expression in two replicates of a breast cancer biopsy using Xenium data. Sequencing reads for the indicated genes are shown as colored dots and overlaid on selected cell types, while all other cell types are displayed in gray with high transparency. The overall imaged tissue area is approximately 7500 × 5500 µm. **(A–D)** Spatial distribution and distance-dependent expression of *SCD* in tumor cells relative to T cells. **(A, C)** Spatial distribution of tumor cells (yellow), T cells (magenta), and *SCD* sequencing reads (blue dots) in sample 1 replicate 1 and replicate 2, respectively. The boxed region labeled I indicates the area shown at higher magnification below. Tumor cells distal to T cells (solid blue arrows) exhibit higher SCD expression than tumor cells proximal to T cells (hollow blue arrows). **(B, D)** Scatter plots showing the positive linear dependency between SCD expression in tumor cells and distance to T cells in replicates 1 and 2. Statistical significance was determined from the regression coefficient using a *t*-test (statsmodels). Replicate 1: $q < 1 \times 10^{-302}$, slope = 0.06. Replicate 2: $q = 1 \times 10^{-174}$, slope = 0.08. **(E–H)** Spatial distribution and distance-dependent expression of *APOC1* in macrophages relative to tumor cells. **(E, G)** Spatial distribution of macrophages (green), tumor cells (yellow), and *APOC1* sequencing reads (blue dots) in sample 1 replicate 1 and replicate 2. Three regions are highlighted and shown at higher magnification below (I–III). **(F, H)** Scatter plots showing the negative linear dependency between *APOC1* expression in macrophages and distance to tumor cells in replicates 1 and 2 (Replicate 1: $q = 1 \times 10^{-98}$, slope = −0.02; Replicate 2: $q = 3 \times 10^{-52}$, slope = −0.02). In panels E and G, a square marked with (*) highlights a region with unusually high *APOC1* expression in macrophage cells that may represent a potential technical artifact.

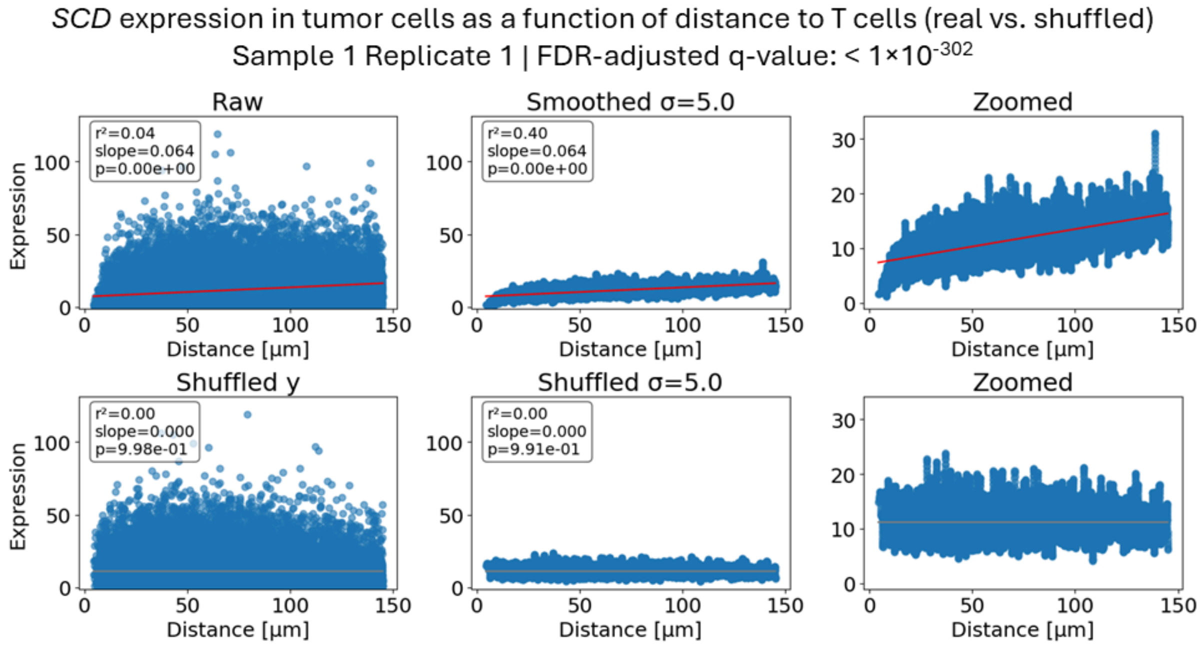

Fig. S38. *SCD* expression in tumor cells as a function of distance to T cells in Xenium sample 1 replicate 1 biopsy. The data in this figure is of Fig. S37, but with the permutation analysis as in Fig. S14. The top row demonstrates a statistically significant positive relationship between *SCD* expression and proximity to T cells ($q < 1 \times 10^{-302}$, slope = 0.06, smoothed $R^2 = 0.4$), which is absent in the shuffled controls (slope ≈ 0, $R^2 \approx 0$).

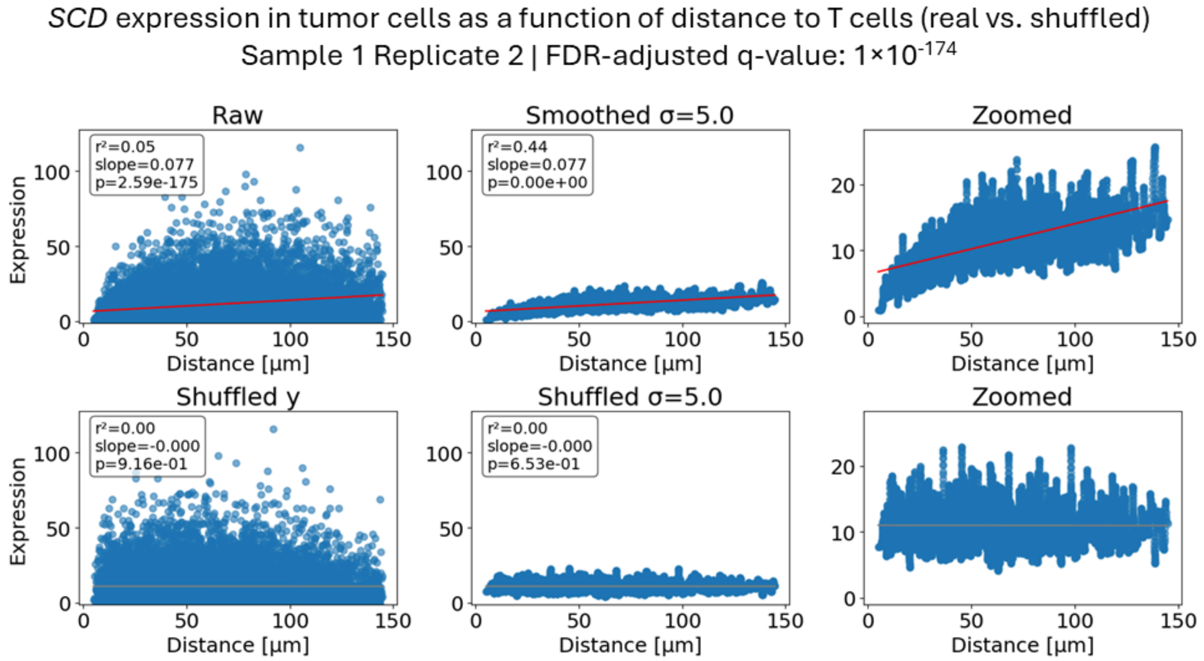

Fig. S39. *SCD* expression in tumor cells as a function of distance to T cells in Xenium sample 1 replicate 2 biopsy. The data in this figure is of Fig. S37, but with the permutation analysis as in Fig. S14. The top row demonstrates a statistically significant positive relationship between *SCD* expression and proximity to T cells ($q = 1 \times 10^{-174}$, slope = 0.08, smoothed $R^2 = 0.44$), which is absent in the shuffled controls (slope ≈ 0, $R^2 \approx 0$).

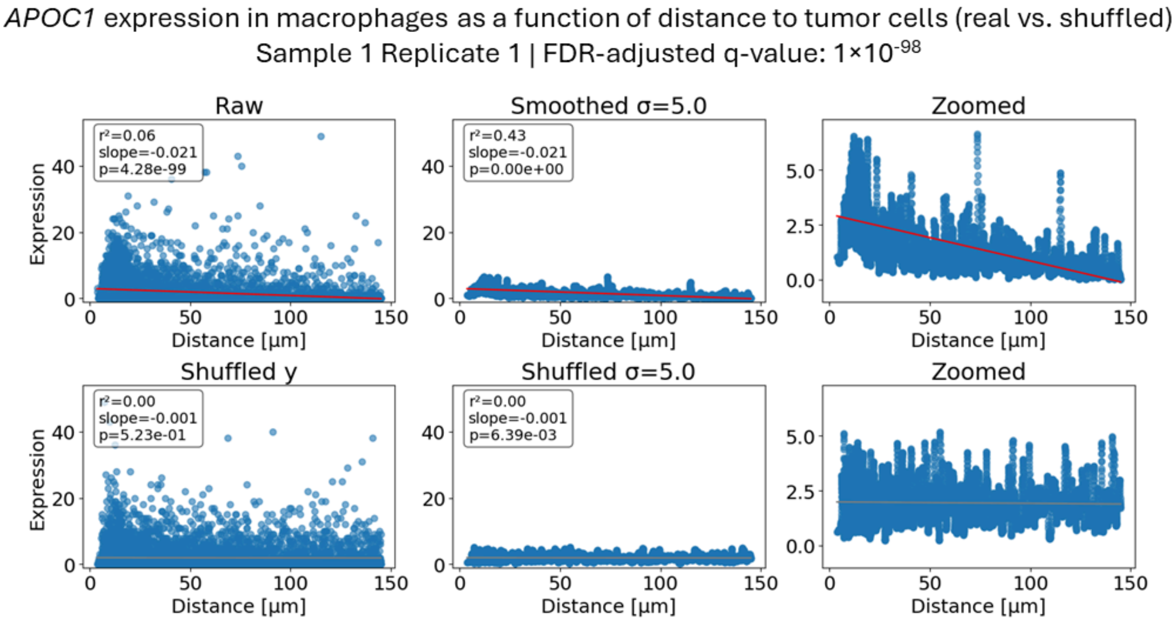

Fig. S40. *APOC1* expression in macrophages as a function of distance to tumor cells in Xenium sample 1 replicate 1 biopsy. The data in this figure is of Fig. S37, but with the permutation analysis as in Fig. S14. The top row demonstrates a statistically significant negative relationship between *APOC1* expression and proximity to tumor cells (q = 1 × 10^{-98}, slope = -0.02, smoothed R^2 = 0.43), which is absent in the shuffled controls (slope ≈ 0, R^2 ≈ 0).

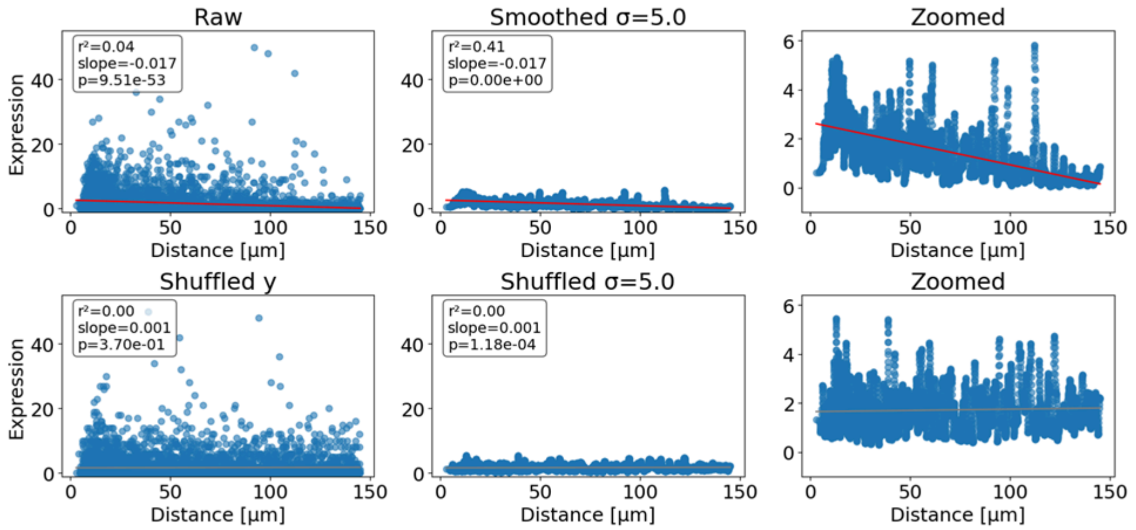

Fig. S41. *APOC1* expression in macrophages as a function of distance to tumor cells in Xenium sample 1 replicate 2 biopsy. Same as Fig. S40, but for replicate 2. Significant negative relationship between *APOC1* expression and proximity to tumor cells (q = 3 × 10^{-52}, slope = -0.02, smoothed R^2 = 0.41, top row), which is absent in the shuffled controls (slope ≈ 0, R^2 ≈ 0, bottom row).

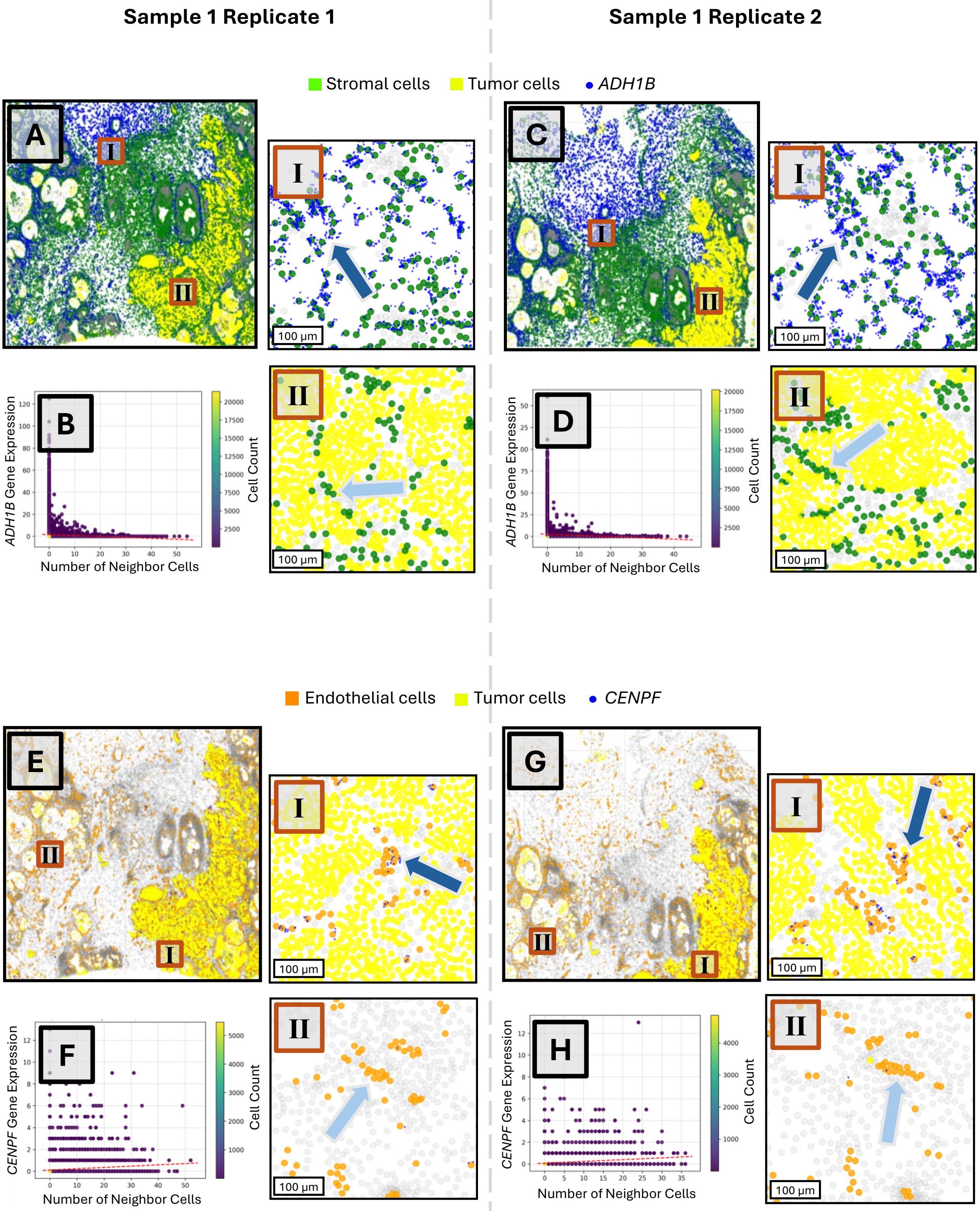

Sample 1 Replicate 1
Sample 1 Replicate 2
Stromal cells
Tumor cells
ADH1B
A
B
C
D
I
II
100 µm
ADH1B Gene Expression
Number of Neighbor Cells
Cell Count
Endothelial cells
Tumor cells
CENPF
E
F
G
H
CENPF Gene Expression

Fig. S42. Examples of neighbor-dependent gene expression in two replicates of a breast cancer biopsy using Xenium data. Sequencing reads for the indicated genes are shown as colored dots and overlaid on selected cell types, while all other cell types are displayed in gray with high transparency. The overall imaged tissue area is approximately 7500 × 5500 µm. Each zoomed subpanel measures approximately 400 × 400 µm. **(A–D)** Spatial distribution and neighbor-dependent expression of *ADH1B* in stromal cells relative to the number of neighboring tumor cells. **(A, C)** Spatial distribution of stromal cells (green), tumor cells (yellow), and *ADH1B* sequencing reads (blue dots) in sample 1 replicates 1 and 2, respectively. Regions labeled I and II indicate areas shown at higher magnification. Stromal cells with fewer neighboring tumor cells exhibit higher *ADH1B* expression (solid blue arrows), whereas stromal cells surrounded by many tumor cells show lower expression (hollow blue arrows). **(B, D)** Scatter plots showing the negative linear dependency between *ADH1B* expression in stromal cells and the number of neighboring tumor cells in replicates 1 and 2. Statistical significance was determined from the regression coefficient using a *t*-test (statsmodels). Replicate 1: $q = 4 \times 10^{-103}$, slope = −0.09. Replicate 2: $q = 9 \times 10^{-65}$, slope = −0.10. **(E–H)** Spatial distribution and neighbor-dependent expression of *CENPF* in endothelial cells relative to the number of neighboring tumor cells. **(E, G)** Spatial distribution of endothelial cells (orange), tumor cells (yellow), and *CENPF* sequencing reads (blue dots) in sample 1 replicates 1 and 2. Regions labeled I and II indicate areas shown at higher magnification. Endothelial cells surrounded by many tumor cells exhibit higher *CENPF* expression (solid blue arrows), whereas endothelial cells with fewer neighboring tumor cells show lower expression (hollow blue arrows). **(F, H)** Scatter plots showing the positive linear dependency between *CENPF* expression in endothelial cells and the number of neighboring tumor cells in replicates 1 and 2 (Replicate 1: $q = 7 \times 10^{-37}$, slope = 0.01; Replicate 2: $q = 2 \times 10^{-54}$, slope = 0.02).

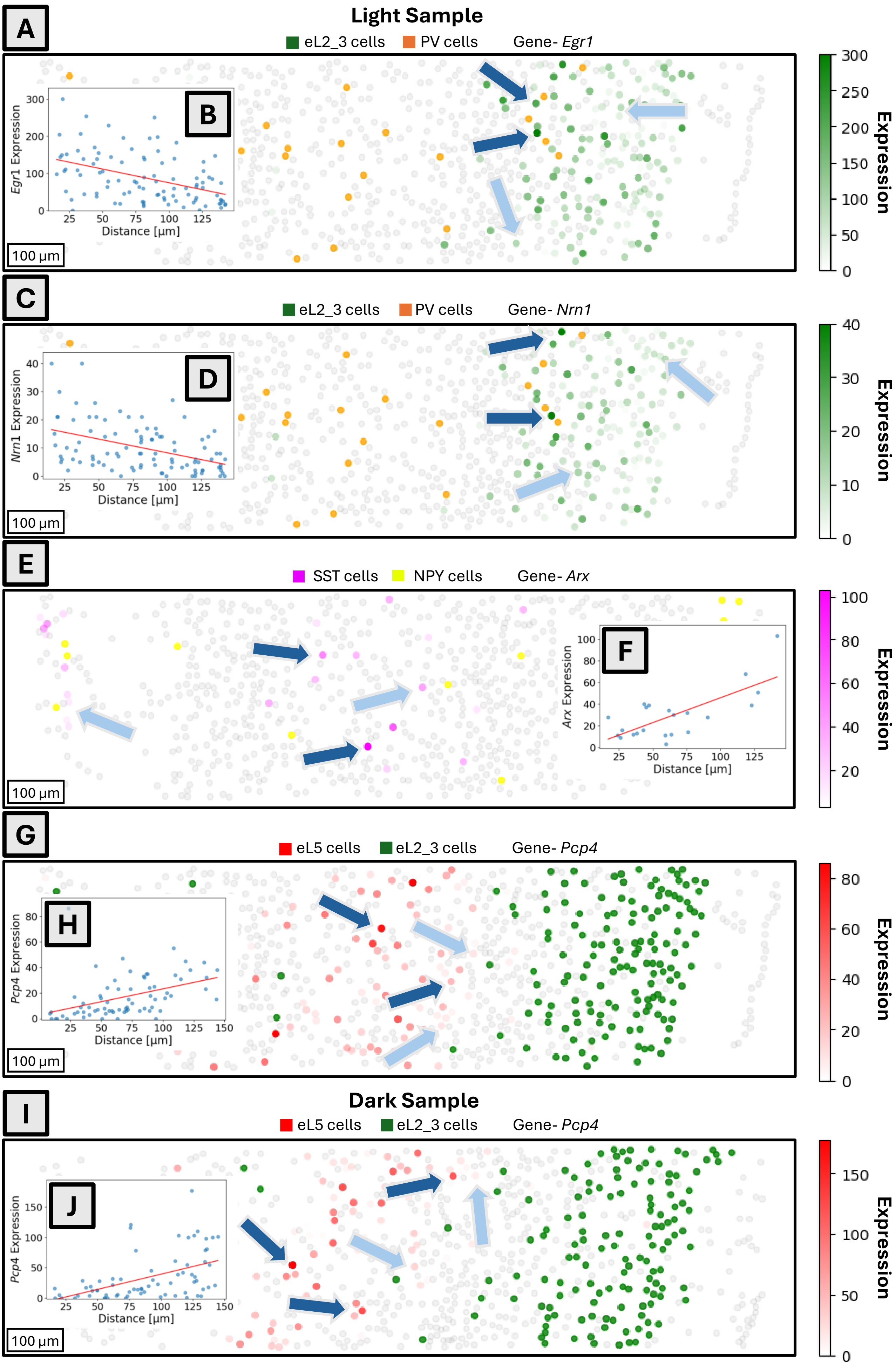

A
Light Sample
eL2_3 cells
PV cells
Gene- Egr1
B
Egr1 Expression
Distance [µm]
100 µm
Expression
C
eL2_3 cells
PV cells
Gene- Nrn1
D
Nrn1 Expression
Distance [µm]
100 µm
Expression
E
SST cells
NPY cells
Gene- Arx
F
Arx Expression
Distance [µm]
100 µm
Expression
G
eL5 cells
eL2_3 cells
Gene- Pcp4
H
Pcp4 Expression
Distance [µm]
100 µm
Expression
I
Dark Sample
eL5 cells
eL2_3 cells
Gene- Pcp4
J
Pcp4 Expression
Distance [µm]
100 µm
Expression

Fig. S43. Examples of proximity-dependent gene expression in mouse primary visual cortex using STARmap data. Spatial maps show examples of gene expression in a primary cell type as a function of distance to a neighboring cell type. Primary cells are color-coded according to transcript counts of the indicated gene (color scale at right), neighboring cells are shown in a distinct solid color, and all other cell types are displayed in gray with high transparency. **(A–B)** Spatial distribution and distance-dependent expression of *Egr1* in eL2_3 cells relative to PV cells under the light condition. **(A)** Spatial distribution of eL2_3 cells (green) and PV cells (orange), with *Egr1* expression in eL2_3 cells shown by the green intensity scale. eL2_3 cells proximal to PV cells (solid blue arrows) exhibit higher *Egr1* expression than distal cells (hollow blue arrows). **(B)** Scatter plot showing the negative linear dependency between *Egr1* expression in eL2_3 cells and distance to PV cells. Statistical significance was determined from the regression coefficient using a *t*-test (statsmodels); $q < 9 \times 10^{-3}$, slope = −0.74. **(C–D)** Spatial distribution and distance-dependent expression of *Nrn1* in eL2_3 cells relative to PV cells under the light condition. **(C)** Spatial distribution of eL2_3 cells (green) and PV cells (orange), with *Nrn1* expression in eL2_3 cells shown by the green intensity scale. eL2_3 cells proximal to PV cells (solid blue arrows) exhibit higher *Nrn1* expression than distal cells (hollow blue arrows). **(D)** Scatter plot showing the negative linear dependency between *Nrn1* expression in eL2_3 cells and distance to PV cells ($q = 9 \times 10^{-3}$, slope = −0.1). **(E–F)** Spatial distribution and distance-dependent expression of *Arx* in SST cells relative to NPY cells under the light condition. **(E)** Spatial distribution of SST cells (magenta) and NPY cells (yellow), with *Arx* expression in SST cells shown by the magenta intensity scale. SST cells distal to NPY cells (solid blue arrows) exhibit higher *Arx* expression than proximal cells (hollow blue arrows). **(F)** Scatter plot showing the positive linear dependency between *Arx* expression in SST cells and distance to NPY cells ($q = 3 \times 10^{-2}$, slope = 0.46). **(G–H)** Spatial distribution and distance-dependent expression of *Pcp4* in eL5 cells relative to eL2_3 cells under the light condition. **(G)** Spatial distribution of eL5 cells (red) and eL2_3 cells (green), with *Pcp4* expression in eL5 cells shown by the red intensity scale. eL5 cells distal to eL2_3 cells (solid blue arrows) exhibit higher *Pcp4* expression than proximal cells (hollow blue arrows). **(H)** Scatter plot showing the positive linear dependency between *Pcp4* expression in eL5 cells and distance to eL2_3 cells ($q = 4 \times 10^{-2}$, slope = 0.2). **(I–J)** Same analysis as in (G–H) for the dark condition. **(I)** Spatial distribution of eL5 cells (red) and eL2_3 cells (green) with *Pcp4* expression in eL5 cells. **(J)** Scatter plot showing the positive linear dependency between *Pcp4* expression in eL5 cells and distance to eL2_3 cells ($q = 2 \times 10^{-2}$, slope = 0.5).

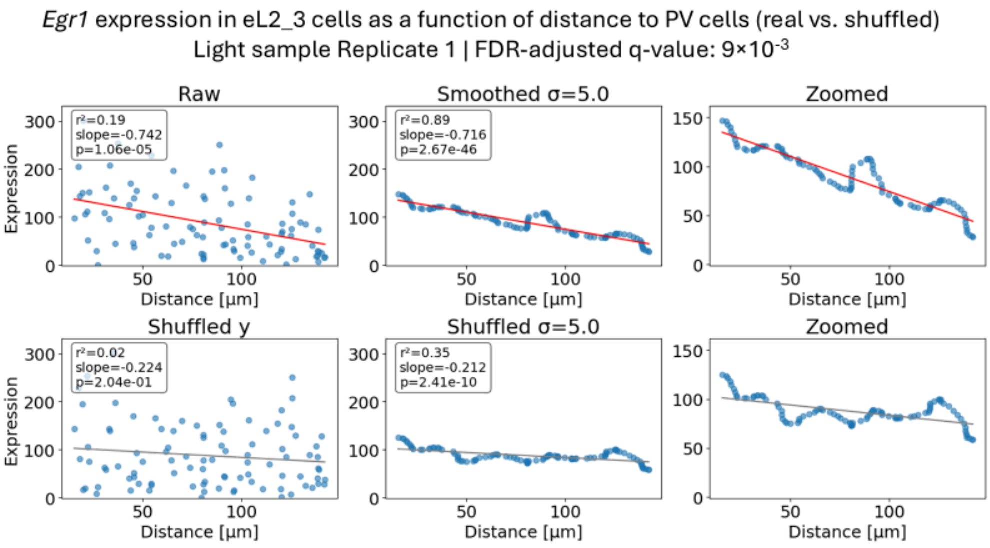

Fig. S44. *Egr1* expression in eL2_3 cells as a function of distance to PV cells in the mouse primary visual cortex in the light condition. The data in this figure is of Fig. S43, but with the permutation analysis as in Fig. S14. The top row demonstrates a statistically significant negative relationship between *Egr1* expression in eL2_3 cells and proximity to PV cells (q = 9 × 10^{-3}, slope = -0.74, smoothed R^2 = 0.89), which is lower in the shuffled controls (slope = -0.2, smoothed R^2 = 0.35).

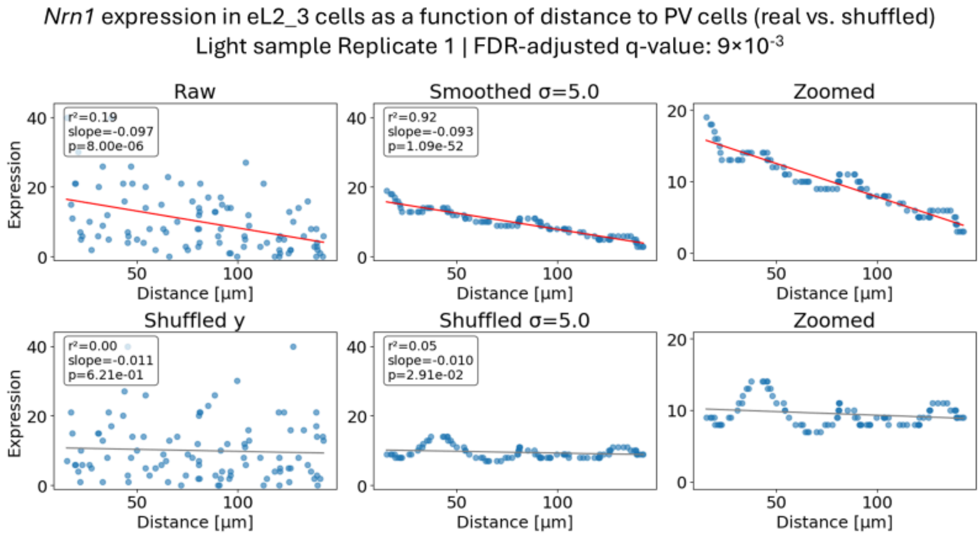

Fig. S45. *Nrn1* expression in eL2_3 cells as a function of distance to PV cells in the mouse primary visual cortex in the light condition. The data in this figure is of Fig. S43, but with the permutation analysis as in Fig. S14. The top row demonstrates a statistically significant negative relationship between *Nrn1* expression in eL2_3 cells and proximity to PV cells (q = 9 × 10^{-3}, slope = -0.1, smoothed R^2 = 0.92), which is lower in the shuffled controls (slope = -0.01, smoothed R^2 = 0.05).

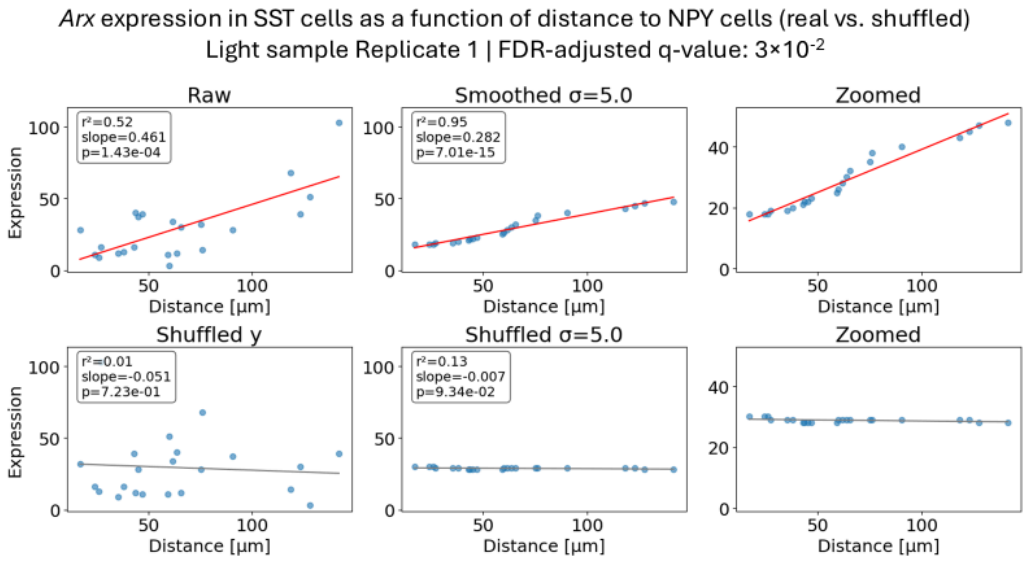

Fig. S46. *Arx* expression in SST cells as a function of distance to NPY cells in the mouse primary visual cortex in the light condition. The data in this figure is of Fig. S43, but with the permutation analysis as in Fig. S14. The top row demonstrates a statistically significant positive relationship between *Arx* expression in SST cells and proximity to NPY cells (q = 3 × 10^{-2}, slope = 0.46, smoothed R^2 = 0.95), which is lower in the shuffled controls (slope = -0.05, smoothed R^2 = 0.13).

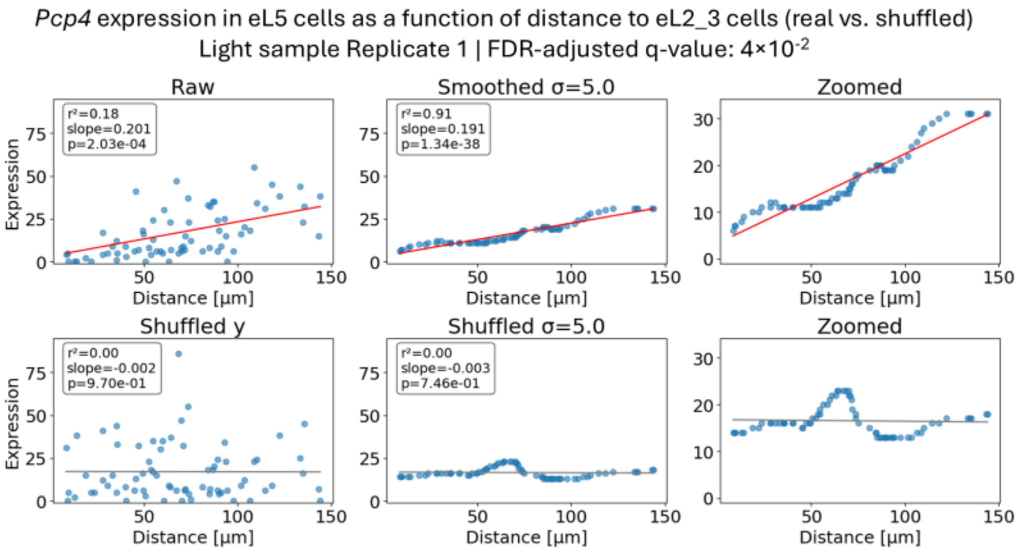

Fig. S47. *Pcp4* expression in eL5 cells as a function of distance to eL2_3 cells in the mouse primary visual cortex in the light condition. The data in this figure is of Fig. S43, but with the permutation analysis as in Fig. S14. The top row demonstrates a statistically significant positive relationship between *Pcp4* expression in eL5 cells and proximity to eL2_3 cells (q = 4 × 10^{-2}, slope = 0.2, smoothed R^2 = 0.91), which is lower in the shuffled controls (slope ≈ 0, smoothed R^2 ≈ 0).

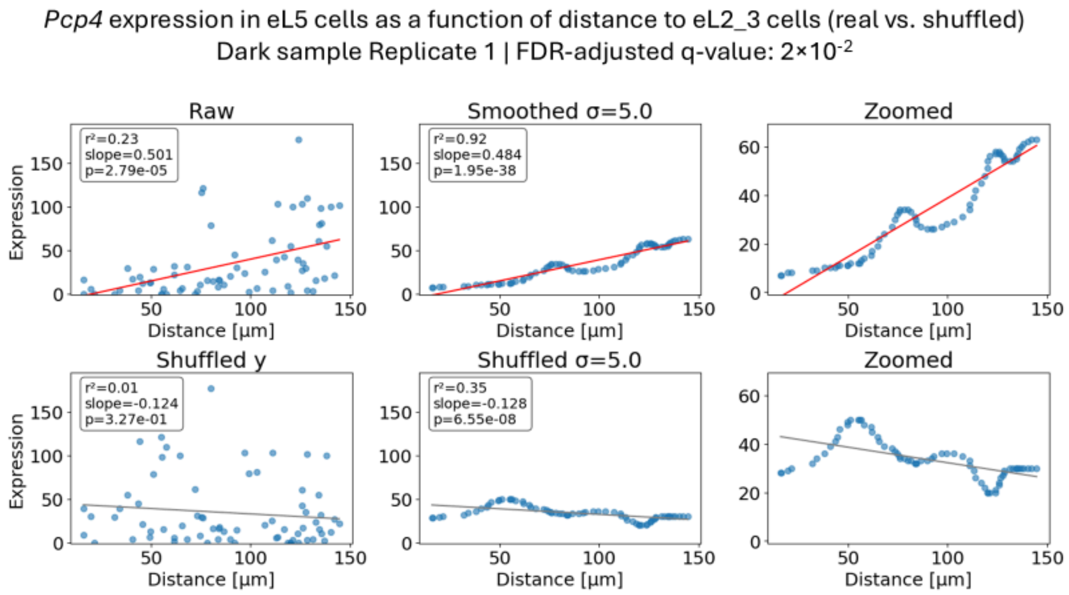

Fig. S48. *Pcp4* expression in eL5 cells as a function of distance to eL2_3 cells in the mouse primary visual cortex in the dark condition. The data in this figure is of Fig. S43, but with the permutation analysis as in Fig. S14. The top row demonstrates a statistically significant positive relationship between *Pcp4* expression in eL5 cells and proximity to eL2_3 cells ($q = 2 \times 10^{-2}$, slope = 0.5, smoothed R^2 = 0.92), which is lower in the shuffled controls (slope = -0.12, smoothed R^2 = 0.35).

Supplementary Tables

Tissue / Cell type	58	59	100	313	330	364	514	783	880	982	Mean values	Minimum values	Maximum values	Total
B cells	70	542	475	270	345	142	0	44	0	124	201.2	0	542	2012
Endothelial cells	0	214	653	212	604	0	327	0	875	258	314.3	0	875	3143
Tumor cells	233	838	1210	30	1058	199	479	87	470	370	497.4	30	1210	4974
Fibroblast	70	0	256	301	401	69	0	0	0	0	109.7	0	401	1097
Macrophage	124	403	0	290	5	0	913	81	339	32	218.7	0	913	2187
Monocyte	206	0	0	0	0	284	0	0	0	81	57.1	0	284	571
NK cells	0	0	0	5	0	0	0	0	0	0	0.5	0	5	5
Smooth muscle	0	0	373	0	0	0	56	0	800	0	122.9	0	800	1229
CD3+ T cells	0	0	314	268	639	383	233	48	0	399	228.4	0	639	2284
CD8+ T cells	0	0	20	37	143	70	1	7	0	35	31.3	0	143	313
Total reads	325329	499560	996719	191373	370950	133471	304666	36135	309789	125954	329394.6	36135	996719	3293946
# of cells	975	2636	3518	1506	3232	1178	2111	386	2575	1414	1953.1	386	3518	19531
Reads per cell	333.67	189.51	283.32	127.07	114.77	113.3	144.32	93.61	120.31	89.08	168.65	89.08	333.67	1608.98
# of FOVs in X axis	11	8	6	9	7	5	6	9	13	6	8	5	13	80
# of FOVs in Y axis	18	12	13	12	17	16	19	10	10	13	14	10	19	140
Sample type (M/P) and tissue source	P breast	P breast	M liver	M liver	M liver	M liver	M axilla	M breast	M liver	M liver	-	-	-	-

Table S1. Cell type distribution and sequencing metrics across tissues (biopsies). The number of cells classified by cell type for each of the ten biopsies analyzed, along with total read counts, the number of cells, and the average reads per cell. Columns correspond to individual biopsies (IDs: 58–982), while rows represent major annotated cell types. The rows also provide the total number of sequencing reads, the number of detected cells, and the average reads per cell (Reads per cell) for each tissue. Two rows report the overall biopsy dimensions along the X and Y axes, expressed in units of 40× fields of view (each 100 × 100 μm). The last columns report: Mean values- Average number of cells per cell type across tissues. Minimum values- Lowest observed cell count per cell type. Maximum values- Highest observed cell count per cell type. Total- Total number of cells per cell type and cumulative metrics across all tissues. Only cells included in the downstream spatial gene expression analysis are shown. The last row provides general information about the biopsies, all obtained from breast cancer patients, including patient disease status (metastatic [M] or non-metastatic [P]) and tissue of collection (predominantly liver metastases).

	tissue	Primary cell type	Neighbor cell type	Original dispersion	Mean permutations dispersion	STD permutations dispersion	z-score	p-value	q-value	Excluding marker genes q-value	tSNE q-value	Shuffled data q-value
1	59	B cells	Tumor cells	0.26	0.20	0.01	21.19	5.3E-100	1.2E-97	3.2E-96	1.9E-18	9.2E-01
2	514	Macrophages	Tumor cells	0.34	0.33	0.02	16.99	4.5E-65	5.3E-63	1.9E-66	1.3E-61	9.2E-01
3	100	Tumor cells	CD3+ T cells	0.19	0.15	0.01	16.04	3.5E-58	2.7E-56	6.9E-55	4.6E-01	9.8E-01
4	100	Endothelial cells	Tumor cells	0.37	0.33	0.02	13.85	6.3E-44	3.6E-42	1.1E-36	1.8E-21	9.7E-01
5	514	Tumor cells	Macrophages	0.48	0.64	0.03	11.64	1.3E-31	5.9E-30	8.0E-31	7.7E-06	9.2E-01
6	100	CD3+ T cells	CD8+ T cells	0.22	0.12	0.02	11.17	3.0E-29	1.1E-27	1.8E-28	7.6E-08	9.6E-01
7	330	Endothelial cells	Tumor cells	0.18	0.20	0.01	9.18	2.1E-20	7.0E-19	2.4E-18	1.7E-04	9.2E-01
8	514	Macrophages	CD3+ T cells	0.08	0.06	0.01	8.64	2.9E-18	8.4E-17	1.1E-17	1.3E-01	9.2E-01
9	880	Smooth muscle cells	Macrophages	0.01	0.00	0.00	8.43	1.7E-17	4.4E-16	4.8E-17	3.2E-01	9.2E-01
10	58	Tumor cells	Monocytes	0.23	0.17	0.03	8.38	2.7E-17	6.2E-16	7.4E-16	4.7E-24	9.2E-01
11	59	B cells	Endothelial cells	0.11	0.10	0.01	7.98	7.3E-16	1.5E-14	2.6E-15	2.4E-12	9.2E-01
12	514	CD3+ T cells	Endothelial cells	0.15	0.09	0.01	7.91	1.3E-15	2.5E-14	1.9E-14	1.1E-03	9.2E-01
13	313	B cells	Fibroblasts	0.14	0.07	0.02	7.48	3.7E-14	6.7E-13	3.2E-12	1.2E-03	9.2E-01
14	330	CD3+ T cells	Tumor cells	0.12	0.10	0.01	7.43	5.3E-14	8.9E-13	2.7E-11	1.5E-05	9.2E-01
15	100	B cells	Fibroblasts	0.14	0.11	0.02	6.69	1.1E-11	1.7E-10	1.9E-10	7.8E-07	9.7E-01
16	514	Endothelial cells	CD3+ T cells	0.12	0.07	0.01	6.64	1.6E-11	2.4E-10	1.9E-10	2.2E-03	9.2E-01
17	514	Smooth muscle cells	Macrophages	0.45	0.36	0.06	6.49	4.2E-11	5.7E-10	2.5E-10	3.9E-04	9.2E-01
18	330	B cells	Fibroblasts	0.02	0.01	0.00	6.18	3.2E-10	4.1E-09	6.9E-10	1.3E-01	9.2E-01
19	364	CD3+ T cells	Tumor cells	0.07	0.05	0.01	6.10	5.3E-10	6.5E-09	2.0E-09	9.0E-03	9.7E-01
20	59	Endothelial cells	B cells	0.25	0.24	0.02	5.98	1.1E-09	1.3E-08	8.0E-09	1.0E-05	9.2E-01
21	100	Smooth muscle cells	CD3+ T cells	0.22	0.19	0.01	5.95	1.3E-09	1.5E-08	2.2E-09	4.1E-02	9.2E-01
22	330	Endothelial cells	CD3+ T cells	0.07	0.06	0.01	5.90	1.8E-09	1.9E-08	7.6E-09	2.5E-02	9.2E-01
23	783	Tumor cells	Macrophages	0.12	0.06	0.02	5.71	5.7E-09	5.8E-08	1.8E-07	2.2E-04	6.6E-01
24	313	Endothelial cells	CD8+ T cells	0.03	0.00	0.00	5.48	2.1E-08	2.0E-07	2.8E-08	3.8E-02	9.2E-01
25	313	Fibroblasts	Endothelial cells	0.06	0.03	0.01	5.43	2.8E-08	2.6E-07	1.4E-07	1.4E-02	9.2E-01
26	58	Macrophages	Tumor cells	0.19	0.05	0.03	5.39	3.6E-08	3.2E-07	5.0E-08	8.1E-02	9.2E-01
27	330	Tumor cells	CD3+ T cells	0.07	0.06	0.00	5.27	6.9E-08	5.9E-07	1.4E-07	3.7E-01	9.2E-01
28	100	B cells	Endothelial cells	0.13	0.10	0.01	5.20	1.0E-07	8.3E-07	1.2E-06	1.4E-01	9.2E-01
29	364	CD8+ T cells	CD3+ T cells	0.58	0.44	0.06	5.19	1.1E-07	8.6E-07	1.4E-07	2.3E-03	9.2E-01
30	982	Endothelial cells	B cells	0.05	0.02	0.01	5.12	1.6E-07	1.2E-06	1.5E-07	1.3E-01	9.2E-01
31	100	Endothelial cells	Smooth muscle cells	0.07	0.10	0.01	4.62	1.9E-06	1.4E-05	3.5E-06	1.7E-10	6.0E-01
32	364	B cells	Monocytes	0.18	0.04	0.04	4.50	3.4E-06	2.5E-05	6.0E-06	1.6E-02	9.2E-01
33	100	Tumor cells	Endothelial cells	0.23	0.22	0.01	4.37	6.2E-06	4.4E-05	5.1E-11	9.4E-05	9.2E-01
34	100	Smooth muscle cells	Tumor cells	0.37	0.38	0.02	4.24	1.1E-05	7.7E-05	5.0E-03	6.5E-02	6.9E-01
35	783	Macrophages	Tumor cells	0.11	0.06	0.02	3.99	3.3E-05	2.1E-04	2.8E-05	4.3E-02	9.2E-01
36	982	Tumor cells	CD3+ T cells	0.10	0.08	0.01	3.99	3.3E-05	2.1E-04	5.2E-05	1.3E-02	9.2E-01
37	330	CD8+ T cells	Fibroblasts	0.05	0.01	0.01	3.95	3.8E-05	2.4E-04	1.5E-04	4.6E-01	9.2E-01
38	880	Endothelial cells	Tumor cells	0.04	0.03	0.00	3.93	4.2E-05	2.6E-04	4.6E-05	3.5E-01	9.2E-01
39	880	Smooth muscle cells	Endothelial cells	0.02	0.01	0.00	3.88	5.1E-05	2.9E-04	9.4E-05	2.1E-01	9.2E-01
40	514	Macrophages	Smooth muscle cells	0.04	0.02	0.01	3.89	5.0E-05	2.9E-04	3.6E-05	9.0E-02	9.2E-01
41	100	Fibroblasts	Endothelial cells	0.25	0.16	0.03	3.89	5.0E-05	2.9E-04	2.0E-04	9.0E-03	9.2E-01
42	330	Fibroblasts	Tumor cells	0.08	0.07	0.01	3.81	7.0E-05	3.9E-04	8.8E-04	1.1E-03	6.6E-01
43	100	CD3+ T cells	Tumor cells	0.43	0.52	0.03	3.72	9.9E-05	5.3E-04	2.3E-04	2.8E-03	9.2E-01
44	514	Endothelial cells	Macrophages	0.15	0.11	0.02	3.63	1.4E-04	7.4E-04	3.8E-04	1.1E-01	9.2E-01
45	100	B cells	Tumor cells	0.08	0.05	0.01	3.60	1.6E-04	8.3E-04	3.7E-04	3.3E-02	9.2E-01
46	313	Macrophages	Fibroblasts	0.08	0.06	0.01	3.53	2.1E-04	1.0E-03	1.5E-04	1.6E-01	9.2E-01
47	100	CD3+ T cells	Endothelial cells	0.13	0.16	0.02	3.51	2.2E-04	1.1E-03	3.3E-04	1.1E-04	1.3E-01
48	364	Monocytes	Tumor cells	0.05	0.04	0.01	3.38	3.6E-04	1.7E-03	5.2E-04	9.6E-02	9.2E-01
49	313	Fibroblasts	Tumor cells	0.04	0.03	0.01	3.33	4.4E-04	2.1E-03	1.7E-03	1.6E-02	4.5E-03
50	330	Tumor cells	Endothelial cells	0.10	0.12	0.01	3.23	6.2E-04	2.9E-03	1.5E-03	8.1E-01	9.2E-01
51	313	Endothelial cells	CD3+ T cells	0.05	0.02	0.01	3.12	9.0E-04	4.1E-03	1.2E-03	2.8E-01	9.2E-01
52	982	Endothelial cells	Tumor cells	0.05	0.04	0.01	3.08	1.0E-03	4.6E-03	1.4E-03	4.1E-02	9.2E-01

	tissue	Primary cell type	Neighbor cell type	Original dispersion	Mean permutations dispersion	STD permutations dispersion	z-score	p-value	q-value	Excluding marker genes q-value	tSNE q-value	Shuffled data q-value
53	313	Macrophages	CD3+ T cells	0.05	0.04	0.01	3.07	1.1E-03	4.7E-03	1.2E-03	6.1E-01	9.2E-01
54	880	Endothelial cells	Macrophages	0.01	0.01	0.00	3.03	1.2E-03	5.2E-03	1.7E-03	1.8E-01	9.2E-01
55	514	Endothelial cells	Tumor cells	0.05	0.02	0.01	2.90	1.8E-03	7.8E-03	5.0E-03	1.3E-01	9.2E-01
56	330	Tumor cells	CD8+ T cells	0.02	0.01	0.00	2.84	2.3E-03	9.4E-03	2.2E-03	3.9E-01	9.2E-01
57	313	Fibroblasts	Macrophages	0.07	0.05	0.01	2.81	2.5E-03	1.0E-02	4.2E-03	7.9E-01	9.2E-01
58	364	CD3+ T cells	B cells	0.06	0.04	0.01	2.76	2.9E-03	1.2E-02	4.5E-03	8.8E-03	9.2E-01
59	982	CD3+ T cells	CD8+ T cells	0.04	0.04	0.01	2.68	3.7E-03	1.5E-02	4.6E-03	2.1E-01	9.2E-01
60	514	Smooth muscle cells	Endothelial cells	0.19	0.20	0.05	2.66	3.9E-03	1.5E-02	1.1E-02	8.1E-01	9.2E-01
61	100	Endothelial cells	CD3+ T cells	0.06	0.08	0.01	2.64	4.1E-03	1.6E-02	4.8E-03	2.2E-09	9.2E-01
62	100	CD8+ T cells	Endothelial cells	0.16	0.05	0.04	2.59	4.8E-03	1.8E-02	4.9E-03	9.8E-01	9.2E-01
63	100	CD8+ T cells	B cells	0.16	0.05	0.04	2.54	5.6E-03	2.0E-02	8.5E-03	9.8E-01	9.2E-01
64	58	Tumor cells	Macrophages	0.05	0.03	0.01	2.51	6.0E-03	2.2E-02	1.2E-02	8.1E-01	9.2E-01
65	100	Endothelial cells	Fibroblasts	0.08	0.07	0.01	2.51	6.1E-03	2.2E-02	1.7E-02	1.0E-03	9.2E-01
66	100	CD3+ T cells	Fibroblasts	0.02	0.01	0.00	2.46	6.9E-03	2.4E-02	1.2E-02	1.6E-01	9.2E-01
67	313	Endothelial cells	Fibroblasts	0.05	0.04	0.01	2.46	7.0E-03	2.4E-02	7.9E-03	8.3E-02	9.2E-01
68	59	Endothelial cells	Tumor cells	0.07	0.06	0.01	2.44	7.4E-03	2.5E-02	8.5E-03	3.3E-06	9.3E-01
69	313	CD3+ T cells	CD8+ T cells	0.03	0.02	0.01	2.44	7.4E-03	2.5E-02	6.4E-03	4.4E-01	9.2E-01
70	982	Endothelial cells	Monocytes	0.04	0.03	0.01	2.42	7.7E-03	2.6E-02	1.1E-02	5.5E-01	9.2E-01

Table S2. Proximity-related cell dispersion in low-dimensional embeddings. For each biopsy (tissue) and each cell-type pair (any combination of primary and neighbor cell types), primary-type cells were projected into a low-dimensional expression embedding, and the dispersion of cells spatially proximal (≤1 μm) to neighbor-type cells was quantified (see Methods). For each biopsy and primary-neighbor cell-type pair, we report: (i) the ratio of dispersion in the PCA embedding of proximal primary-type cells divided by the total dispersion of all primary-type cells ("original dispersion"); (ii) the corresponding ratio after permutation shuffling ("mean permutations dispersion"); (iii) the standard deviation of the ratios from permutation shuffling ("STD permutations dispersion"); (iv) the final proximity z-score, defined using a max-type statistic that combines two complementary proximity metrics (proximal cells-dispersion shift from overall centroid and two centroids separation); (v) the corresponding one-sided *p*-value derived from the Gaussian-standardized z-score of (iv); (vi) *q*-values. This analysis identified 70 significant ($q < 0.05$) proximity-induced cell states among 232 tested combinations. Columns (viii)–(x) provide robustness and sensitivity checks: (viii) *q*-values from a sensitivity analysis (after excluding neighbor cell-type marker genes from the primary-type expression profiles to control for segmentation artifacts), which confirmed the same 70 significant pairs; (ix) *q*-values obtained by repeating the analysis using an alternative embedding (*t*-SNE), where 38 of the 70 pairs remained significant; and (x) *q*-values from a global permutation test (full-pipeline shuffling), in which only 3 of 232 combinations reached significance, and none of the 70 originally significant pairs did so, confirming robustness against chance (see Methods).

Primary cell type	Neighbor cell type	Tissue	Log_2 fold change cutoff	# Detected genes	Detected genes	# Genes with positive log_2 fold change	Genes with positive log_2 fold change	# Genes with negative log_2 fold change	Genes with negative log_2 fold change
Macrophages	Tumor cells	514	0.15	117	*ACTA2, ADGRL4, AGR2, AKT1, APOE, AR, BLVRA, C1QA, CALCRL, CCL2, CCL4, CCND1, CCNE1, CD163, CD19, CD3E, CD40, CD40LG, CD5, CD69, CDC6, CDH11, CDK6, CDKN2A, CLDN4, COL4A1, COL4A2, CSRP2, CSTB, CTCF, CTSL, CXCR5, CXXC5, DCN, DERL3, EFNA5, EIF3E, ELF5, ERBB3, ESR1, FABP7, FAP, FASN, FAT1, FOS, FOXC1, FTL, GATA3, GNLY, GSN, GZMB, HLA.C, HLA.E, IGF1R, IGFBP5, IGHG1, IGHM, IL3RA, ITGA6, KIF23, KIT, KRAS, KRT14, KRT17, KRT5, KRT8, LAMA1, LAMC1, LYPD6B, LYZ, MAPK13, MIA, MLPH, MMP11, MMP12, MMP9, MS4A1, MSR1, MT2A, MYB, MYLK, NCAM1, NDC80, NDRG2, NFKBIA, NOTCH1, NR3C1, PABPC1, PHGDH, PLEK, PLVAP, POU2AF1, PRLR, S100A14, S100A4, S100A8, S100A9, SDC1, SFRP1, SKAP1, SLC2A1, SNAI2, TAGLN, TCF4, TCL1A, TFF3, TFRC, THY1, TMEM45B, TMSB10, TPM2, TSPAN1, TTYH1, TYMS, UBE2C, UBE2T, XCL1*	96	*ACTA2, AGR2, AKT1, AR, BLVRA, C1QA, CALCRL, CCL4, CCND1, CCNE1, CD3E, CD40LG, CD5, CD69, CDC6, CDH11, CDK6, CDKN2A, CLDN4, CSRP2, CSTB, CTCF, CTSL, DCN, EFNA5, EIF3E, ELF5, ERBB3, ESR1, FABP7, FAP, FASN, FAT1, FOS, FOXC1, FTL, GATA3, GNLY, GSN, GZMB, HLA.C, HLA.E, IGF1R, IGFBP5, IGHG1, IGHM, ITGA6, KIF23, KIT, KRAS, KRT14, KRT5, KRT8, LAMC1, LYPD6B, LYZ, MAPK13, MLPH, MMP12, MMP9, MS4A1, MSR1, MT2A, MYB, MYLK, NDRG2, NFKBIA, NOTCH1, NR3C1, PABPC1, PHGDH, PLEK, PLVAP, POU2AF1, PRLR, S100A14, S100A9, SDC1, SFRP1, SKAP1, SLC2A1, SNAI2, TAGLN, TCF4, TCL1A, TFF3, TFRC, THY1, TMEM45B, TMSB10, TPM2, TSPAN1, TYMS, UBE2C, UBE2T, XCL1*	21	*ADGRL4, APOE, CCL2, CD163, CD19, CD40, COL4A1, COL4A2, CXCR5, CXXC5, DERL3, IL3RA, KRT17, LAMA1, MIA, MMP11, NCAM1, NDC80, S100A4, S100A8, TTYH1*
Tumor cells	Monocytes	58	0.4	99	*ACTG2, ANLN, AR, BICC1, C10orf54, C1QA, C1QB, CCL3, CCND1, CD14, CD36, CD38, CD3G, CD7, CD9, CDK7, CDKN2A, COL3A1, COL4A5, CSTB, CTCF, CXXC5, DCN, EIF3E, FABP7, FASN, FAT1, FCRL5, FOS, GNLY, GZMB, HIF1A, HLA.C, HLA.DPA1, HLA.DRA, HLA.DRB1, HSPG2, IGF1R, IGHG4, KIF23, KIT, KRAS, KRT17, KRT5, KRT8, LAMA1, LTF, LUM, LYPD6B, LYZ, MAPK13, MAPK3, MMP12, MS4A1, MSR1, MT2A, MUC1, MYB, MYBL2, MYLK, MYO5B, MZB1, NAT1, NDRG2, NFKBIA, NOTCH1, NR3C1, NUF2, PABPC1, PBK, PDCD1, PECAM1, PI3, PLEK, PRLR, PTPRC, RAMP2, RPL13, RPSA, S100A14, SDC1, SFRP1, SKAP1, SLC2A1, SOX10, SPDEF, STAT5A, TCF4, TCL1A, TFF1, TFF3, TFRC, THY1, TPM2, TRAC, TSPAN1, TYMS, UBE2C, BACTERIA*	90	*ANLN, AR, BICC1, C1QA, C1QB, CCL3, CCND1, CD14, CD36, CD38, CD3G, CD9, CDK7, CDKN2A, COL3A1, COL4A5, CSTB, CTCF, CXXC5, EIF3E, FABP7, FASN, FAT1, FCRL5, FOS, GNLY, GZMB, HIF1A, HLA.C, HLA.DPA1, HLA.DRA, HLA.DRB1, IGF1R, IGHG4, KIF23, KIT, KRAS, KRT17, KRT5, KRT8, LAMA1, LTF, LUM, LYPD6B, LYZ, MAPK13, MAPK3, MMP12, MS4A1, MSR1, MT2A, MUC1, MYB, MYBL2, MYLK, MYO5B, NAT1, NDRG2, NFKBIA, NOTCH1, NR3C1, NUF2, PABPC1, PBK, PDCD1, PECAM1, PI3, PLEK, PRLR, PTPRC, RAMP2, RPL13, RPSA, S100A14, SDC1, SFRP1, SKAP1, SOX10, STAT5A, TCF4, TCL1A, TFF1, TFF3, TFRC, THY1, TPM2, TRAC, TSPAN1, UBE2C, BACTERIA*	9	*ACTG2, C10orf54, CD7, DCN, HSPG2, MZB1, SLC2A1, SPDEF, TYMS*

Primary cell type	Neighbor cell type	Tissue	Log_2 fold change cutoff	# Detected genes	Detected genes	# Genes with positive log_2 fold change	Genes with positive log_2 fold change	# Genes with negative log_2 fold change	Genes with negative log_2 fold change
Macrophages	Tumor cells	58	0.25	79	*ANLN, AR, BICC1, C1QA, C1QB, CCL3, CCNE1, CD14, CD19, CD38, CD3E, CD3G, CDK6, CDK7, CDKN2A, COL3A1, CSTB, CXXC5, EFNA5, EIF3E, ELF5, FABP7, FASN, FAT1, FOS, GNLY, HIF1A, HLA.A, HLA.DPA1, HLA.DRA, IGF1R, IGKC, IL7R, ISG15, KIF23, KRAS, KRT15, KRT17, KRT5, KRT8, LAMA1, LGALS1, LTF, LUM, MAPK3, MIA, MMP11, MMP12, MS4A1, MSR1, MT2A, MYBL2, MYO5B, NAT1, NOTCH1, NR3C1, PABPC1, PDCD1, PECAM1, PLEK, PLVAP, PTPRC, PTTG1, RAMP2, RPL13, RPSA, S100A9, SDC1, SFRP1, SKAP1, SOX10, SPP1, TCF4, TCL1A, TFRC, THY1, TMEM45B, TPM2, ZEB1*	79	*ANLN, AR, BICC1, C1QA, C1QB, CCL3, CCNE1, CD14, CD19, CD38, CD3E, CD3G, CDK6, CDK7, CDKN2A, COL3A1, CSTB, CXXC5, EFNA5, EIF3E, ELF5, FABP7, FASN, FAT1, FOS, GNLY, HIF1A, HLA.A, HLA.DPA1, HLA.DRA, IGF1R, IGKC, IL7R, ISG15, KIF23, KRAS, KRT15, KRT17, KRT5, KRT8, LAMA1, LGALS1, LTF, LUM, MAPK3, MIA, MMP11, MMP12, MS4A1, MSR1, MT2A, MYBL2, MYO5B, NAT1, NOTCH1, NR3C1, PABPC1, PDCD1, PECAM1, PLEK, PLVAP, PTPRC, PTTG1, RAMP2, RPL13, RPSA, S100A9, SDC1, SFRP1, SKAP1, SOX10, SPP1, TCF4, TCL1A, TFRC, THY1, TMEM45B, TPM2, ZEB1*	0	
Tumor cells	CD3+ T cells	100	0.15	64	*ACTA2, AKT1, APOE, AURKA, BICC1, CCNB1, CCND1, CD36, CD3E, CD3G, CD63, CD69, CD74, CD9, CDKN2A, CRABP2, CTSL, EPCAM, ERBB2, FAP, FASN, FOS, FOXP3, FTL, FXYD3, GNG11, HIF1A, HLA.A, HLA.B, HSPB1, HSPG2, IFITM3, JUN, KIF23, KRT18, KRT19, LAMA1, LST1, LTF, LYZ, MAPK3, MDM2, MKI67, MT2A, MYL6, NOTCH1, PABPC1, RPL13, RPL18, RPSA, RRM2, S100A14, SLC39A6, SOX10, STMN1, SULF1, TFF1, TFF3, THY1, TMSB10, TMSB4X, TYMS, XBP1, ZNF571*	58	*AKT1, APOE, AURKA, BICC1, CCND1, CD36, CD3E, CD3G, CD63, CD74, CD9, CDKN2A, CRABP2, CTSL, EPCAM, ERBB2, FAP, FASN, FOS, FOXP3, FTL, FXYD3, HIF1A, HLA.A, HLA.B, HSPB1, HSPG2, IFITM3, JUN, KRT18, KRT19, LST1, LTF, LYZ, MAPK3, MDM2, MKI67, MT2A, MYL6, NOTCH1, PABPC1, RPL13, RPL18, RPSA, RRM2, S100A14, SLC39A6, SOX10, STMN1, SULF1, TFF1, TFF3, THY1, TMSB10, TMSB4X, TYMS, XBP1, ZNF571*	6	*ACTA2, CCNB1, CD69, GNG11, KIF23, LAMA1*
B cells	Tumor cells	59	0.2	61	*ANLN, APOC1, AR, BANK1, BGN, CAPN13, CCNB1, CD2, CD38, CD68, CD74, CD79A, CDK4, CRABP2, CSTB, CTCF, CTLA4, FABP7, FOXC1, FOXP3, FTL, GATA3, GNLY, HLA.A, HLA.C, HLA.DRB5, IGF1R, IGKC, ITGAX, KRAS, LGMN, LILRB1, LYZ, MAPK13, MMP12, MS4A1, NF1, NKG7, NOTCH1, OBP2B, ORC6, PABPC1, PDPN, PHGDH, PI3, PIK3CA, PLEK, POU2AF1, RPL18, S100A9, SFRP1, SIAH2, SLC39A6, SNAI2, TCF4, THY1, TMEM45B, TMSB4X, TPM2, TRAC, XCL1*	52	*ANLN, APOC1, AR, BANK1, BGN, CAPN13, CD38, CD79A, CDK4, CRABP2, CSTB, CTCF, CTLA4, FABP7, FOXC1, FOXP3, FTL, GATA3, GNLY, HLA.A, HLA.C, HLA.DRB5, IGF1R, ITGAX, KRAS, LGMN, LILRB1, LYZ, MAPK13, MMP12, MS4A1, NF1, NKG7, OBP2B, ORC6, PABPC1, PDPN, PHGDH, PI3, PLEK, S100A9, SFRP1, SIAH2, SLC39A6, SNAI2, TCF4, THY1, TMEM45B, TMSB4X, TPM2, TRAC, XCL1*	9	*CCNB1, CD2, CD68, CD74, IGKC, NOTCH1, PIK3CA, POU2AF1, RPL18*
Tumor cells	T cells	100	0.15	60	*ACTA2, AKT1, APOE, AURKA, BICC1, CCND1, CD36, CD63, CD69, CD74, CD9, CDKN2A, CRABP2, CTSL, EPCAM, ERBB2, FAP, FASN, FOS, FTL, FXYD3, GNG11, HIF1A, HLA.A, HLA.B, HSPB1, HSPG2, IFITM3, JUN, KIF23, KRT18, KRT19, LAMA1, LST1, LTF, LYZ, MAPK3, MDM2, MKI67, MT2A, MYL6, NOTCH1, PABPC1, RPL13, RPL18, RPSA, RRM2, S100A14, SLC39A6, SOX10, STMN1, SULF1, TFF1, TFF3, THY1, TMSB10, TMSB4X, TYMS, XBP1, ZNF571*	55	*AKT1, APOE, AURKA, BICC1, CCND1, CD36, CD63, CD74, CD9, CDKN2A, CRABP2, CTSL, EPCAM, ERBB2, FAP, FASN, FOS, FTL, FXYD3, HIF1A, HLA.A, HLA.B, HSPB1, HSPG2, IFITM3, JUN, KRT18, KRT19, LST1, LTF, LYZ, MAPK3, MDM2, MKI67, MT2A, MYL6, NOTCH1, PABPC1, RPL13, RPL18, RPSA, RRM2, S100A14, SLC39A6, SOX10, STMN1, SULF1, TFF1, TFF3, THY1, TMSB10, TMSB4X, TYMS, XBP1, ZNF571*	5	*ACTA2, CD69, GNG11, KIF23, LAMA1*

Primary cell type	Neighbor cell type	Tissue	Log_2 fold change cutoff	# Detected genes	Detected genes	# Genes with positive log_2 fold change	Genes with positive log_2 fold change	# Genes with negative log_2 fold change	Genes with negative log_2 fold change
Endothelial cells	Tumor cells	100	0.2	57	*ACTA2, ANLN, BCL2, C1QA, CCNB1, CCND1, CD63, CD9, CDH11, CDH3, CDK4, CDK6, CDK7, CRABP2, FASN, FOS, FOXP3, FTL, HLA.A, HLA.B, HSPB1, IFITM3, IGF1R, KRAS, KRT10, KRT8, LAMA1, LGALS1, LILRB1, LYZ, MDM2, MT2A, MYL6, MYL9, NKG7, NOTCH1, PABPC1, PLVAP, POU2AF1, PTPRB, PTPRC, RPL13, RPL18, RPSA, RRM2, S100A9, SDC1, SOX10, SPP1, STMN1, TFF2, TFRC, TMEM45B, TMSB4X, TYMS, XBP1, ZNF571*	57	*ACTA2, ANLN, BCL2, C1QA, CCNB1, CCND1, CD63, CD9, CDH11, CDH3, CDK4, CDK6, CDK7, CRABP2, FASN, FOS, FOXP3, FTL, HLA.A, HLA.B, HSPB1, IFITM3, IGF1R, KRAS, KRT10, KRT8, LAMA1, LGALS1, LILRB1, LYZ, MDM2, MT2A, MYL6, MYL9, NKG7, NOTCH1, PABPC1, PLVAP, POU2AF1, PTPRB, PTPRC, RPL13, RPL18, RPSA, RRM2, S100A9, SDC1, SOX10, SPP1, STMN1, TFF2, TFRC, TMEM45B, TMSB4X, TYMS, XBP1, ZNF571*	0	
Tumor cells	Endothelial cells	100	0.15	39	*ACTA2, APOE, CD69, CDK6, COL3A1, CST3, FASN, FOS, HLA.B, HSPB1, IGKC, KIF23, KRAS, KRT18, KRT19, KRT8, LAMA1, LST1, LTF, LYZ, MAPK3, MT2A, MYL6, MYL9, POU2AF1, PTPRC, RPSA, S100A14, SDC1, TCF4, TFF1, TFF2, TFF3, TFRC, THY1, TMSB4X, TSPAN1, TTC6, XBP1*	32	*ACTA2, CD69, CDK6, CST3, FASN, HLA.B, HSPB1, IGKC, KIF23, KRAS, KRT18, KRT19, KRT8, LAMA1, LYZ, MT2A, MYL6, MYL9, POU2AF1, PTPRC, RPSA, S100A14, SDC1, TCF4, TFF1, TFF2, TFF3, TFRC, TMSB4X, TSPAN1, TTC6, XBP1*	7	*APOE, COL3A1, FOS, LST1, LTF, MAPK3, THY1*
Tumor cells	Macrophages	514	0.25	34	*AHR, AKT1, AR, AURKA, BIRC5, BLVRA, CCL2, CD79A, CDH1, CEACAM1, CR2, CRABP2, CSRP2, CXXC5, ERBB3, FABP7, FTL, HSPB1, ITGAX, KRT17, KRT19, LYPD6B, NCAM1, NDC80, NUF2, PI3, PRLR, PTPRC, RAMP2, S100A4, S100A8, SFRP1, TBX21, TCF4*	7	*AKT1, FABP7, LYPD6B, PRLR, PTPRC, SFRP1, TCF4*	27	*AHR, AR, AURKA, BIRC5, BLVRA, CCL2, CD79A, CDH1, CEACAM1, CR2, CRABP2, CSRP2, CXXC5, ERBB3, FTL, HSPB1, ITGAX, KRT17, KRT19, NCAM1, NDC80, NUF2, PI3, RAMP2, S100A4, S100A8, TBX21*
Tumor cells	Macrophages	58	0.4	32	*BICC1, CD14, CD19, CD36, CD38, CD3G, CD9, EFNA5, FABP7, FCN1, FOS, GRB7, HLA.B, HLA.DRA, IL7R, KIF23, LTF, MAPK3, MMP11, MT2A, MYO10, NAT1, NFKBIA, NR3C1, PLVAP, RPL13, S100A14, SOX10, TCF4, TFF1, TRAC, VIM*	32	*BICC1, CD14, CD19, CD36, CD38, CD3G, CD9, EFNA5, FABP7, FCN1, FOS, GRB7, HLA.B, HLA.DRA, IL7R, KIF23, LTF, MAPK3, MMP11, MT2A, MYO10, NAT1, NFKBIA, NR3C1, PLVAP, RPL13, S100A14, SOX10, TCF4, TFF1, TRAC, VIM*	0	
Endothelial cells	Tumor cells	330	0.25	29	*AR, BGN, C1QA, CAPN13, CD38, CD68, CDC6, CDK6, COL4A5, ELF5, FGFR4, FOXC1, GNLY, HLA.DPA1, KIT, KRT15, KRT5, LYZ, MLPH, MYB, NKG7, PECAM1, S100A9, TFF2, TFRC, TMSB10, TPM2, TYMS, UBE2T*	24	*BGN, C1QA, CAPN13, CDC6, CDK6, COL4A5, ELF5, FGFR4, FOXC1, GNLY, HLA.DPA1, KIT, KRT15, KRT5, LYZ, MYB, PECAM1, S100A9, TFF2, TFRC, TMSB10, TPM2, TYMS, UBE2T*	5	*AR, CD38, CD68, MLPH, NKG7*

Primary cell type	Neighbor cell type	Tissue	Log_2 fold change cutoff	# Detected genes	Detected genes	# Genes with positive log_2 fold change	Genes with positive log_2 fold change	# Genes with negative log_2 fold change	Genes with negative log_2 fold change
Tumor cells	Endothelial cells	59	0.3	28	*BGN, CAPN13, CCNB1, CDH11, CSTB, DCN, ERBB4, FABP7, FTL, GATA3, HLA.C, HLA.DRB5, IGFBP5, ITGAX, JUN, LAMA1, LYZ, MMP12, MYB, MZB1, NF1, NOTCH1, OBP2B, POU2AF1, PRLR, S100A9, SKAP1, SNAI2*	10	*CCNB1, DCN, HLA.DRB5, IGFBP5, JUN, LAMA1, MZB1, NOTCH1, POU2AF1, SKAP1*	18	*BGN, CAPN13, CDH11, CSTB, ERBB4, FABP7, FTL, GATA3, HLA.C, ITGAX, LYZ, MMP12, MYB, NF1, OBP2B, PRLR, S100A9, SNAI2*
Tumor cells	T cells	330	0.25	25	*AR, BANK1, CCNB1, CD38, CD63, CDK6, CDKN2A, GPNMB, GRB7, HLA.DRB5, HLA.E, IGHM, KRT18, LDB2, MAPK13, MELK, MZB1, NUF2, PHGDH, SFRP1, SNAI2, SOX10, TFRC, TMSB10, UBE2T*	18	*AR, BANK1, CCNB1, CD63, CDK6, GPNMB, HLA.DRB5, IGHM, KRT18, LDB2, MAPK13, MELK, NUF2, PHGDH, SFRP1, SNAI2, SOX10, UBE2T*	7	*CD38, CDKN2A, GRB7, HLA.E, MZB1, TFRC, TMSB10*
Tumor cells	Smooth muscle cells	100	0.15	23	*CCNB1, CD24, CD36, CD68, CD69, CDK4, CDK6, FASN, FOS, FOXP3, HIF1A, KIF23, KRT8, LAMA1, LILRB1, LTF, LYZ, MAPK3, MT2A, NKG7, PLVAP, TFRC, ZNF571*	9	*CD24, CD36, FOS, FOXP3, HIF1A, LTF, LYZ, MAPK3, ZNF571*	14	*CCNB1, CD68, CD69, CDK4, CDK6, FASN, KIF23, KRT8, LAMA1, LILRB1, MT2A, NKG7, PLVAP, TFRC*
Tumor cells	CD3+ T cells	330	0.25	19	*AR, BANK1, CCNB1, CD3E, CD63, CDK6, FAT1, HLA.DRB5, IGHM, KRT18, LDB2, MAPK13, MELK, MZB1, NKG7, NUF2, SNAI2, TFRC, TMSB10*	15	*AR, BANK1, CCNB1, CD63, CDK6, FAT1, HLA.DRB5, IGHM, KRT18, LDB2, MAPK13, MELK, NKG7, NUF2, SNAI2*	4	*CD3E, MZB1, TFRC, TMSB10*
Tumor cells	Fibroblasts	58	0.4	17	*C1QA, CD274, CD36, CDKN2A, CSTB, FCN1, FOS, GNLY, HLA.C, HLA.DPA1, HLA.DRA, KRT5, MS4A1, MT2A, PLEK, TCF4, TFRC*	1	*CD274*	16	*C1QA, CD36, CDKN2A, CSTB, FCN1, FOS, GNLY, HLA.C, HLA.DPA1, HLA.DRA, KRT5, MS4A1, MT2A, PLEK, TCF4, TFRC*
Tumor cells	Endothelial cells	330	0.25	17	*CCL4, CD74, CD79A, CDKN2A, DCN, DERL3, ERBB2, FGFR4, FOXC1, GNLY, LGMN, MAPK13, MYB, PHGDH, POU2AF1, RRM2, TP53*	10	*CCL4, CD79A, CDKN2A, ERBB2, FGFR4, FOXC1, GNLY, MYB, RRM2, TP53*	7	*CD74, DCN, DERL3, LGMN, MAPK13, PHGDH, POU2AF1*

Primary cell type	Neighbor cell type	Tissue	Log_2 fold change cutoff	# Detected genes	Detected genes	# Genes with positive log_2 fold change	Genes with positive log_2 fold change	# Genes with negative log_2 fold change	Genes with negative log_2 fold change
T cells	Tumor cells	100	0.2	16	*AR, CD2, CSTB, GATA3, ICOS, IGHG1, IGHM, IGKC, JUN, KRT17, LAMA1, LGALS2, LILRB1, TMSB10, TRAC, XBP1*	2	*LAMA1, LILRB1*	14	*AR, CD2, CSTB, GATA3, ICOS, IGHG1, IGHM, IGKC, JUN, KRT17, LGALS2, TMSB10, TRAC, XBP1*
T cells	Tumor cells	364	0.45	15	*AIF1, CD19, CD38, CDH3, CDKN2A, FOXC1, HLA.E, IGKC, ISG20, LAMA1, MYB, MZB1, S100A4, TFRC, TPM2*	15	*AIF1, CD19, CD38, CDH3, CDKN2A, FOXC1, HLA.E, IGKC, ISG20, LAMA1, MYB, MZB1, S100A4, TFRC, TPM2*	0	
CD3+ T cells	Tumor cells	364	0.45	14	*AIF1, CD38, CDH3, CDKN2A, FOXC1, HLA.E, ISG20, LAMA1, MYB, MYO10, MZB1, S100A4, TFRC, TPM2*	14	*AIF1, CD38, CDH3, CDKN2A, FOXC1, HLA.E, ISG20, LAMA1, MYB, MYO10, MZB1, S100A4, TFRC, TPM2*	0	
Tumor cells	B cells	59	0.3	14	*ANLN, BLVRA, CD24, CDC20, CSTB, FOXP3, FTL, FXYD3, GATA3, HLA.A, KRAS, KRT10, SNAI2, TPM2*	8	*ANLN, CSTB, FOXP3, GATA3, HLA.A, KRAS, SNAI2, TPM2*	6	*BLVRA, CD24, CDC20, FTL, FXYD3, KRT10*
Endothelial cells	Tumor cells	59	0.2	13	*BCL2, BLVRA, BRAF, DCN, FGFR2, GZMB, HLA.DRB5, LAMA1, MZB1, PABPC1, PHGDH, SKAP1, SNAI2*	13	*BCL2, BLVRA, BRAF, DCN, FGFR2, GZMB, HLA.DRB5, LAMA1, MZB1, PABPC1, PHGDH, SKAP1, SNAI2*	0	
CD3+ T cells	Tumor cells	100	0.2	12	*CD2, GATA3, ICOS, IGHG1, IGKC, JUN, KRT17, LAMA1, LILRB1, TMSB10, TRAC, XBP1*	2	*LAMA1, LILRB1*	10	*CD2, GATA3, ICOS, IGHG1, IGKC, JUN, KRT17, TMSB10, TRAC, XBP1*
Tumor cells	Smooth muscle cells	880	0.35	11	*AR, CCNB1, CD19, CDH3, COL4A5, FCRL5, HLA.B, MAPK3, MMP12, PLVAP, TPM2*	6	*AR, CCNB1, CD19, FCRL5, HLA.B, MAPK3*	5	*CDH3, COL4A5, MMP12, PLVAP, TPM2*
Tumor cells	T cells	982	0.3	10	*CDKN2A, FABP7, HLA.E, IFITM3, IGF1R, MMP12, MYB, MYO5B, TCF4, TMEM45B*	10	*CDKN2A, FABP7, HLA.E, IFITM3, IGF1R, MMP12, MYB, MYO5B, TCF4, TMEM45B*	0	
Monocytes	Tumor cells	58	0.25	9	*C1QA, FCN1, FTL, MS4A1, MT2A, PLEK, S100A9, SFRP1, TCL1A*	2	*FCN1, PLEK*	7	*C1QA, FTL, MS4A1, MT2A, S100A9, SFRP1, TCL1A*
Tumor cells	Fibroblasts	330	0.25	9	*CD3E, GPNMB, IGFBP5, ISG20, LGMN, LYZ, MMP12, POU2AF1, TYMS*	8	*CD3E, GPNMB, IGFBP5, LGMN, LYZ, MMP12, POU2AF1, TYMS*	1	*ISG20*
Tumor cells	T cells	364	0.45	9	*CALCRL, CTSL, GNLY, KIT, LYZ, MSR1, NOTCH1, TCL1A, TMSB10*	5	*GNLY, KIT, MSR1, TCL1A, TMSB10*	4	*CALCRL, CTSL, LYZ, NOTCH1*

Primary cell type	Neighbor cell type	Tissue	Log_2 fold change cutoff	# Detected genes	Detected genes	# Genes with positive log_2 fold change	Genes with positive log_2 fold change	# Genes with negative log_2 fold change	Genes with negative log_2 fold change
Tumor cells	CD3+ T cells	364	0.45	9	*BCL2, CALCRL, CTSL, HLA.DRB5, LYZ, MSR1, MZB1, NOTCH1, TCL1A*	2	*MSR1, TCL1A*	7	*BCL2, CALCRL, CTSL, HLA.DRB5, LYZ, MZB1, NOTCH1*
Tumor cells	CD3+ T cells	514	0.25	9	*AKT1, AR, CDKN2A, HLA.E, LYZ, PTPRC, S100A14, S100A9, TFF3*	6	*HLA.E, LYZ, PTPRC, S100A14, S100A9, TFF3*	3	*AKT1, AR, CDKN2A*
Tumor cells	Endothelial cells	880	0.35	9	*CD24, CD36, CD5, FOS, LYZ, S100A14, SIAH2, TFF2, TYMS*	3	*SIAH2, TFF2, TYMS*	6	*CD24, CD36, CD5, FOS, LYZ, S100A14*
Tumor cells	CD3+ T cells	982	0.3	9	*CDKN2A, FABP7, HLA.E, IFITM3, MMP12, MYB, MYO5B, TCF4, TMEM45B*	9	*CDKN2A, FABP7, HLA.E, IFITM3, MMP12, MYB, MYO5B, TCF4, TMEM45B*	0	
CD3+ T cells	Tumor cells	330	0.25	8	*C1QA, CD63, CDKN2A, FABP7, HLA.E, ISG20, TFRC, TYMS*	8	*C1QA, CD63, CDKN2A, FABP7, HLA.E, ISG20, TFRC, TYMS*	0	
Tumor cells	T cells	514	0.25	7	*AKT1, AR, CDKN2A, HLA.E, S100A14, S100A9, TFF3*	4	*HLA.E, S100A14, S100A9, TFF3*	3	*AKT1, AR, CDKN2A*
Tumor cells	Macrophages	783	0.4	6	*HLA.C, MAPK13, MYB, NOTCH1, TFRC, UBE2T*	6	*HLA.C, MAPK13, MYB, NOTCH1, TFRC, UBE2T*	0	
Smooth muscle cells	Tumor cells	100	0.2	5	*FOS, LYZ, SOX10, TMSB4X, ZNF571*	5	*FOS, LYZ, SOX10, TMSB4X, ZNF571*	0	
Tumor cells	Fibroblasts	100	0.15	5	*ANLN, FOS, IGHG1, IGHG4, IGKC*	4	*ANLN, IGHG1, IGHG4, IGKC*	1	*FOS*
Tumor cells	Monocytes	364	0.45	5	*AIF1, FGFR4, KRT5, LYPD6B, TMEM45B*	5	*AIF1, FGFR4, KRT5, LYPD6B, TMEM45B*	0	
Tumor cells	CD8+ T cells	364	0.45	5	*ERBB2, GNLY, KIT, PTPRC, TFRC*	5	*ERBB2, GNLY, KIT, PTPRC, TFRC*	0	
T cells	Tumor cells	514	0.15	4	*CSRP2, GATA3, NOTCH1, SKAP1*	4	*CSRP2, GATA3, NOTCH1, SKAP1*	0	
CD3+ T cells	Tumor cells	514	0.15	4	*CSRP2, GATA3, NOTCH1, SKAP1*	4	*CSRP2, GATA3, NOTCH1, SKAP1*	0	
Macrophages	Tumor cells	783	0.35	4	*BIRC5, CRABP2, MYB, TFRC*	4	*BIRC5, CRABP2, MYB, TFRC*	0	
T cells	Tumor cells	330	0.25	3	*C1QA, FABP7, TYMS*	3	*C1QA, FABP7, TYMS*	0	
Tumor cells	CD8+ T cells	330	0.25	3	*BANK1, MAPK13, SOX10*	3	*BANK1, MAPK13, SOX10*	0	
Fibroblasts	Tumor cells	58	0.25	2	*FTL, RPSA*	1	*RPSA*	1	*FTL*
B cells	Tumor cells	100	0.2	2	*LYZ, MDM2*	2	*LYZ, MDM2*	0	
Fibroblasts	Tumor cells	330	0.25	2	*CD8A, FGFR4*	2	*CD8A, FGFR4*	0	
Endothelial cells	Tumor cells	880	0.35	2	*BCL2, CDH3*	2	*BCL2, CDH3*	0	
Endothelial cells	Tumor cells	514	0.15	1	*GPNMB*	1	*GPNMB*	0	
CD3+ T cells	Tumor cells	982	0.35	1	*PRLR*	1	*PRLR*	0	
Tumor cells	B cells	100	0.15	1	*TFF1*	0		1	*TFF1*
Tumor cells	Endothelial cells	514	0.25	1	*PRLR*	1	*PRLR*	0	

Primary cell type	Neighbor cell type	Tissue	Log_2 fold change cutoff	# Detected genes	Detected genes	# Genes with positive log_2 fold change	Genes with positive log_2 fold change	# Genes with negative log_2 fold change	Genes with negative log_2 fold change
Tumor cells	Smooth muscle cells	514	0.25	1	*THY1*	1	*THY1*	0	
Tumor cells	B cells	783	0.4	1	*TYMS*	1	*TYMS*	0	
Tumor cells	Monocytes	982	0.3	1	*TTC6*	1	*TTC6*	0	
Tumor cells	CD8+ T cells	982	0.3	1	*MYO5B*	1	*MYO5B*	0	

Table S3. Proximity-induced differential expression across tissues and cell-type pairs. Differentially expressed genes identified for each biopsy (tissue) in non-tumor cells as a function of proximity to tumor cells, and in tumor cells as a function of proximity to non-tumor cells. For each tissue, proximity-induced genes were identified by differential expression analysis between primary-type cells spatially proximal (≤1 μm) to neighbor-type cells and those positioned at greater distances (Methods). Primary-neighbor cell-type combinations included non-tumor versus tumor, as well as the reciprocal direction (tumor versus non-tumor). For each tissue and cell-type pair, we report: (**i**) the number of significant proximity-induced genes identified, (**ii**) the corresponding gene set, (**iii**) the number of significant proximity-induced genes with positive $\log_2$ fold change, (**iv**) the corresponding upregulated gene set, (**v**) the number of significant proximity-induced genes with negative $\log_2$ fold change, (**vi**) the corresponding downregulated gene set and (**vii**) the absolute $\log_2$ fold-change cutoff applied in the differential expression analysis. The absolute $\log_2$ fold-change cutoff was applied to increase stringency in detecting differentially expressed genes. This cutoff was slightly adjusted across tissue-cell-type pairs (0.15–0.45) to avoid over-detection of differential expression in individual cases.

Primary cell type	Neighbor cell type	Tissue i	Tissue ii	# Genes in tissue i	# Genes in tissue ii	p-value	q-value	# Intersected genes	Intersected genes
Macrophages	Tumor cells	58	514	79	117	2.E-04	5.E-03	45	*AR, C1QA, CCNE1, CD19, CD3E, CDK6, CDKN2A, CSTB, CXXC5, EFNA5, EIF3E, ELF5, FABP7, FASN, FAT1, FOS, GNLY, IGF1R, KIF23, KRAS, KRT17, KRT5, KRT8, LAMA1, MIA, MMP11, MMP12, MS4A1, MSR1, MT2A, NOTCH1, NR3C1, PABPC1, PLEK, PLVAP, S100A9, SDC1, SFRP1, SKAP1, TCF4, TCL1A, TFRC, THY1, TMEM45B, TPM2*
Endothelial cells	Tumor cells	330	100	44	71	1.E-01	3.E-01	14	*C1QA, CAPN13, CD74, CDK6, HSPB1, LYZ, NKG7, NOTCH1, RRM2, S100A9, TFF2, TFRC, TMSB10, TYMS*
B cells	Tumor cells	100	59	10	71	6.E-02	2.E-01	5	*C1QA, FOXP3, KRAS, LYZ, TMSB4X*
Endothelial cells	Tumor cells	880	100	11	71	9.E-02	3.E-01	5	*ANLN, BCL2, CDH3, FOS, S100A9*
Macrophages	Tumor cells	783	514	8	117	2.E-01	3.E-01	5	*FTL, LAMA1, MYB, POU2AF1, TFRC*
T cells	Tumor cells	364	330	26	19	2.E-02	1.E-01	5	*CDKN2A, HLA.E, ISG20, PHGDH, TFRC*
CD3+ T cells	Tumor cells	364	330	28	20	3.E-02	2.E-01	5	*CDKN2A, HLA.E, ISG20, TFRC, TMSB10*
CD3+ T cells	Tumor cells	364	100	28	35	2.E-01	4.E-01	5	*CD19, IGKC, LAMA1, TCL1A, TMSB10*
Tumor cells	T cells	330	100	25	60	6.E-01	7.E-01	5	*CD63, CDKN2A, KRT18, SOX10, TMSB10*
Tumor cells	CD3+ T cells	330	100	19	64	4.E-01	6.E-01	5	*CCNB1, CD3E, CD63, KRT18, TMSB10*
Tumor cells	CD3+ T cells	100	514	64	9	2.E-02	1.E-01	5	*AKT1, CDKN2A, LYZ, S100A14, TFF3*
Endothelial cells	Tumor cells	880	330	11	44	6.E-02	2.E-01	4	*COL4A5, MZB1, S100A9, TPM2*
Endothelial cells	Tumor cells	100	59	71	17	6.E-01	7.E-01	4	*BCL2, LAMA1, PABPC1, TMEM45B*
Tumor cells	Endothelial cells	880	100	9	39	2.E-02	1.E-01	4	*FOS, LYZ, S100A14, TFF2*
Tumor cells	T cells	364	100	9	60	8.E-02	3.E-01	4	*CTSL, LYZ, NOTCH1, TMSB10*
Tumor cells	T cells	100	514	60	7	3.E-02	1.E-01	4	*AKT1, CDKN2A, S100A14, TFF3*
B cells	Tumor cells	982	59	3	71	1.E-02	1.E-01	3	*FOS, KRAS, SFRP1*
T cells	Tumor cells	364	100	26	34	6.E-01	7.E-01	3	*CDH3, IGKC, LAMA1*
Tumor cells	Endothelial cells	330	59	17	28	2.E-01	4.E-01	3	*DCN, MYB, POU2AF1*
Tumor cells	Endothelial cells	100	59	39	28	7.E-01	8.E-01	3	*LAMA1, LYZ, POU2AF1*
Tumor cells	T cells	330	514	25	7	1.E-02	1.E-01	3	*AR, CDKN2A, HLA.E*
Tumor cells	CD3+ T cells	364	100	9	64	3.E-01	5.E-01	3	*CTSL, LYZ, NOTCH1*
Tumor cells	Smooth muscle cells	880	100	11	23	4.E-02	2.E-01	3	*CCNB1, MAPK3, PLVAP*
B cells	Tumor cells	330	59	7	71	5.E-01	7.E-01	2	*GNLY, LYZ*
Endothelial cells	Tumor cells	880	59	11	17	1.E-01	3.E-01	2	*BCL2, MZB1*
Macrophages	Tumor cells	783	58	8	79	7.E-01	7.E-01	2	*LAMA1, TFRC*
T cells	Tumor cells	982	330	5	19	3.E-02	2.E-01	2	*FABP7, MMP12*
T cells	Tumor cells	330	100	19	34	7.E-01	7.E-01	2	*RRM2, TMSB10*
CD3+ T cells	Tumor cells	330	100	20	35	7.E-01	7.E-01	2	*RRM2, TMSB10*
Tumor cells	Macrophages	58	514	32	34	9.E-01	9.E-01	2	*FABP7, TCF4*
Tumor cells	Monocytes	364	58	5	99	5.E-01	7.E-01	2	*KRT5, LYPD6B*
Tumor cells	T cells	982	330	10	25	2.E-01	4.E-01	2	*CDKN2A, HLA.E*
Tumor cells	T cells	982	100	10	60	6.E-01	7.E-01	2	*CDKN2A, IFITM3*
Tumor cells	T cells	982	514	10	7	2.E-02	1.E-01	2	*CDKN2A, HLA.E*
Tumor cells	CD3+ T cells	982	100	9	64	6.E-01	7.E-01	2	*CDKN2A, IFITM3*
Tumor cells	CD3+ T cells	982	514	9	9	3.E-02	1.E-01	2	*CDKN2A, HLA.E*
Tumor cells	CD3+ T cells	364	330	9	19	1.E-01	3.E-01	2	*HLA.DRB5, MZB1*

Primary cell type	Neighbor cell type	Tissue i	Tissue ii	# Genes in tissue i	# Genes in tissue ii	p-value	q-value	# Intersected genes	Intersected genes
B cells	Tumor cells	982	100	3	10	1.E-01	3.E-01	1	*KRAS*
B cells	Tumor cells	330	100	7	10	2.E-01	4.E-01	1	*LYZ*
Endothelial cells	Tumor cells	330	59	44	17	9.E-01	9.E-01	1	*MZB1*
Endothelial cells	Tumor cells	330	514	44	1	1.E-01	3.E-01	1	*GPNMB*
T cells	Tumor cells	100	514	34	4	4.E-01	5.E-01	1	*GATA3*
CD3+ T cells	Tumor cells	982	330	4	20	2.E-01	4.E-01	1	*FABP7*
CD3+ T cells	Tumor cells	330	514	20	4	2.E-01	4.E-01	1	*NOTCH1*
CD3+ T cells	Tumor cells	100	514	35	4	4.E-01	5.E-01	1	*GATA3*
Tumor cells	Endothelial cells	880	59	9	28	6.E-01	7.E-01	1	*LYZ*
Tumor cells	Endothelial cells	330	100	17	39	9.E-01	9.E-01	1	*POU2AF1*
Tumor cells	Endothelial cells	59	514	28	1	9.E-02	3.E-01	1	*PRLR*
Tumor cells	T cells	364	330	9	25	5.E-01	7.E-01	1	*TMSB10*
Tumor cells	CD3+ T cells	364	514	9	9	2.E-01	5.E-01	1	*LYZ*
Tumor cells	CD3+ T cells	330	514	19	9	4.E-01	7.E-01	1	*AR*
Tumor cells	Fibroblasts	100	58	5	17	3.E-01	5.E-01	1	*FOS*

Table S4. Intersections of proximity-dependent differentially expressed genes across biopsies/tissues. For each tissue, proximity-induced genes were identified by differential expression analysis between primary-type cells spatially proximal (≤1 μm) to neighbor-type cells and those positioned at greater distances (Table S3). To assess whether overlaps of proximity-induced genes between biopsies exceeded random expectation, we performed a permutation-based significance test (Methods). For each biopsy pair (biopsy i and biopsy ii) and cell-type pair (primary and neighbor cell types), we reported: (i) the number of proximity-induced genes identified in each tissue, (ii) the intersecting gene set, (iii) the intersection size, (iv) the empirical permutation-derived *p*-value, and (v) *q*-values.

Primary cell type	Neighbor cell type	ExSeq-MERFISH similarity *p*-value	Receptor status similarity *p*-value	PC1 *explained variance* (*p*-value)	PC2 *explained variance* (*p*-value)
Endothelial cells	Tumor cells	Tissue 982: *p*-value=4.E-02<br><br>Tissue 514: *p*-value=7.E-02	5.E-01	36% (1.E-04)	20% (1.E-04)
Macrophages	Tumor cells	Tissue 514: *p*-value=9.E-01		50% (1.E-04)	20% (1.E-04)
Monocytes	Tumor cells	Tissue 982: *p*-value=2.E-01		34% (1.E-04)	28% (1.E-04)
T cells	Tumor cells	Tissue 982: *p*-value=2.E-02<br><br>Tissue 514: *p*-value=6.E-02	2.E-01	30% (1.E-04)	19% (1.E-04)
CD3+ T cells	Tumor cells		6.E-02	36% (1.E-04)	24% (1.E-04)
Smooth muscle cells	Tumor cells			32% (9.E-04)	28% (3.E-04)
Fibroblasts	Tumor cells			26% (1.E-04)	21% (1.E-04)
Tumor cells	B cells		2.E-01	28% (1.E-04)	24% (1.E-04)
Tumor cells	Endothelial cells	Tissue 514: *p*-value=4.E-01<br><br>Tissue 880: *p*-value=4.E-01	2.E-01	21% (1.E-04)	18% (1.E-04)
Tumor cells	Macrophages	Tissue 982: *p*-value=7.E-02<br><br>Tissue 514: pval=9.E-01	4.E-03	27% (1.E-04)	19% (1.E-04)
Tumor cells	T cells	Tissue 982: *p*-value=4.E-01<br><br>Tissue 514: *p*-value=2.E-01	4.E-01	28% (1.E-04)	16% (1.E-04)
Tumor cells	CD8+ T cells		5.E-01	33% (1.E-04)	28% (6.E-04)
Tumor cells	Fibroblasts			28% (1.E-04)	25% (1.E-04)

Table S5. Cross-platform and cross-patient concordance of proximity-induced expression programs. For each cell-type pair, with cell type *i* designated as the primary and cell type *ii* as the neighbor, proximity-induced genes were identified by differential expression analysis comparing primary-type cells located proximal (≤1 μm) to neighbor-type cells with those located farther away. Analyses were performed across all biopsies (tissues), incorporating both ExSeq and MERFISH datasets from adjacent sections of the same samples when available.
For each dataset, DESeq2 *p*-values of proximity-induced genes were extracted and projected into principal component analysis (PCA) space, positioning each patient-platform combination (ExSeq or MERFISH) within a two-dimensional embedding. For each primary-neighbor cell-type pair, the following values are reported: (i) permutation-based significance of concordance between ExSeq and MERFISH representations of the same tissue; ExSeq and MERFISH proximity-induced programs were significantly closer in two-dimensional PCA space than expected by chance for biopsy 982 in T cells proximal versus distant from tumor cells ($p < 0.02$) and in endothelial cells proximal versus distant from tumor cells ($p < 0.04$); (ii) permutation-based significance of program similarity (clustering in PCA space) across ExSeq datasets from patients sharing the same $HR^{+}/HER2^{-}$ status; specifically, tumor cells proximal versus distant to macrophages exhibited a similar program across patients sharing the same $HR^{+}/HER2^{-}$ status ($p < 0.004$), and (iii–iv) assessment of the significance of the PCA embedding, including (iii) the percentage of variance explained by the first principal component, with its permutation-based significance (in parentheses), and (iv) that of the second principal component.

Cell type pair	Tissue	Gene	# Interacting cells	98th percentile expression	98th percentile distance (µm)	q value	Slope	R^2 (original)	R^2 (Gaussian)	R^2 (permuted mean)	Composite score
Tumor cells → $CD3^+$ T cells	364	*MZB1*	187	27.28	46.85	9.E-11	0.32	0.24	0.95	5.E-03	200
Endothelial → Tumor cells	330	*ERBB2*	597	51.08	56.00	5.E-13	-0.29	0.10	0.59	1.E-03	198
$CD3^+$ T cells → Tumor cells	100	*IGKC*	314	17.48	28.42	9.E-11	0.22	0.15	0.78	3.E-03	192
B cells → Tumor cells	59	*CSTB*	311	41.80	47.71	2.E-11	-0.33	0.16	0.59	3.E-03	192
Fibroblast → Tumor cells	313	*MYLK*	155	41.68	76.78	6.E-05	-0.19	0.13	0.80	7.E-03	183
B cells → Tumor cells	59	*IGKC*	311	19.80	47.71	3.E-06	0.10	0.09	0.64	3.E-03	167
Tumor cells → $CD3^+$ T cells	313	*CTSL*	28	65.82	87.10	2.E-02	-0.38	0.29	0.81	4.E-02	166
Endothelial → Tumor cells	313	*FOS*	33	16.52	75.83	7.E-05	0.21	0.50	0.97	2.E-02	163
$CD3^+$ T cells → Tumor cells	100	*XBP1*	314	20.48	28.42	1.E-04	0.20	0.06	0.63	2.E-03	159
Tumor cells → B cells	982	*ERBB2*	265	17.00	69.97	1.E-05	0.08	0.09	0.57	4.E-03	155
Fibroblast → Tumor cells	313	*SOX18*	155	12.92	76.78	2.E-06	-0.06	0.17	0.67	7.E-03	154
$CD3^+$ T cells → Tumor cells	100	*FTL*	314	14.48	28.42	3.E-05	0.14	0.07	0.64	4.E-03	149
Endothelial → Tumor cells	330	*UBE2T*	597	12.08	56.00	4.E-08	-0.05	0.06	0.57	2.E-03	138
Tumor cells → B cells	364	*GRB7*	30	17.20	88.08	4.E-02	0.09	0.23	0.88	4.E-02	137
$CD3^+$ T cells → Tumor cells	313	*NR3C1*	56	14.90	80.02	1.E-02	-0.07	0.16	0.94	2.E-02	136
Tumor cells → $CD3^+$ T cells	313	*PHGDH*	28	10.92	87.10	1.E-02	-0.07	0.32	0.92	3.E-02	132
Smooth muscle → Tumor cells	100	*COL3A1*	373	15.56	27.16	4.E-05	0.14	0.06	0.51	3.E-03	131
Tumor cells → $CD3^+$ T cells	313	*TFRC*	28	13.38	87.10	3.E-02	-0.08	0.27	0.83	3.E-02	130
Fibroblast → Tumor cells	313	*SFRP1*	155	14.92	76.78	5.E-03	-0.05	0.08	0.62	7.E-03	123
Tumor cells → $CD8^+$ T cells	364	*ERBB2*	71	48.60	63.06	3.E-02	-0.32	0.11	0.55	2.E-02	123
Tumor cells → $CD3^+$ T cells	364	*S100A9*	187	17.56	46.85	7.E-03	-0.10	0.06	0.58	5.E-03	121
B cells → Tumor cells	313	*PECAM1*	51	12.00	86.03	2.E-02	0.06	0.17	0.86	2.E-02	120
Monocyte → Tumor cells	364	*S100A9*	188	21.04	86.88	2.E-02	-0.05	0.05	0.52	4.E-03	117
Fibroblast → Tumor cells	313	*DCN*	155	10.84	76.78	2.E-03	-0.03	0.09	0.73	8.E-03	117
Endothelial → Tumor cells	982	*TCF4*	195	12.24	49.69	4.E-03	-0.06	0.06	0.69	6.E-03	116
Tumor cells → Endothelial	514	*CDKN2A*	114	11.74	67.29	4.E-03	0.05	0.11	0.69	1.E-02	106
Fibroblast → Tumor cells	313	*GPNMB*	155	11.00	76.78	2.E-02	0.03	0.06	0.78	5.E-03	106
Tumor cells → Monocyte	58	*PRLR*	121	16.40	45.90	1.E-02	-0.09	0.08	0.60	9.E-03	105
Monocyte → Tumor cells	364	*LYZ*	188	12.00	86.88	1.E-02	-0.03	0.05	0.57	5.E-03	104
Tumor cells → $CD8^+$ T cells	364	*RRM2*	71	12.60	63.06	3.E-02	-0.06	0.11	0.73	2.E-02	101
Tumor cells → $CD8^+$ T cells	364	*CDKN2A*	71	11.00	63.06	3.E-02	-0.06	0.10	0.70	1.E-02	92
Macrophage → Tumor cells	880	*CD24*	120	13.24	67.52	4.E-02	-0.05	0.06	0.56	8.E-03	70
Tumor cells → $CD8^+$ T cells	982	*GATA3*	105	10.76	71.95	4.E-02	0.03	0.07	0.62	1.E-02	68
Tumor cells → Monocyte	364	*PHGDH*	184	10.34	55.90	2.E-02	-0.03	0.05	0.51	5.E-03	68

Table S6. Genes showing linear dependency of expression on the distance between cell types across biopsies using the ExSeq datasets. Each row represents a gene for which expression in a given cell type (primary; left of the arrow) is significantly associated with its distance to another cell type (neighbor; right of the arrow) within a specific tissue sample. This table lists the significant gene-cell type pair–tissue associations ranked by a composite score reflecting the strength, robustness, and dynamic range of the distance-dependent gene expression relationship. Columns include:

- **Cell type pair**: Directional pair representing the primary (X) and neighbor (Y) cell types used in the distance-expression analysis.
- **Tissue**: Tumor biopsy in which the association was identified.
- **Gene**: Gene symbol.
- **# Interacting cells**: Number of primary cells included in the regression.
- **98th percentile expression**: High-end expression value (98th percentile) in the primary cells, representing the upper dynamic range of the response.
- **98th percentile distance**: Upper range of minimum distances between X and Y cells, representing the dynamic range of the predictor.
- ***q* value**: Global FDR-adjusted *p*-value across all tissues and pairs.
- **Slope**: Regression slope indicating the direction and strength of the association between distance and gene expression.
- **R^2 (original)**: Coefficient of determination for the raw (unsmoothed) regression.
- **R^2 (Gaussian)**: R^2 from regression after applying Gaussian smoothing ($\sigma = 5$) to expression values.
- **R^2 (permuted mean)**: Mean R^2 from 100 permutations with shuffled distances.
- **Composite score**: Final rank-based score integrating eight features (see Methods). Higher scores indicate more reproducible and biologically interpretable linear dependency patterns.

All associations listed passed filtering thresholds, including $q < 0.05$ and Gaussian-smoothed $R^2 \geq 0.5$. To minimize potential segmentation artifacts, genes not expressed in at least 20% of single cells of the corresponding primary cell type were excluded based on scRNA-seq data (see Methods).

Cell type pair	Tissue	Gene	Threshold R^2	# Interacting cells	98th percentile expression	98th percentile distance (µm)	q value	Slope	R^2 (original)	R^2 (Gaussian)	R^2 (permuted mean)	Composite score
Fibroblast → Tumor cells	514	*FOS*	0.5	2581	22.00	128.81	2.E-43	0.06	0.08	0.61	3.E-04	14
Endothelial → Tumor cells	514	*FOS*	0.5	1354	20.00	119.33	9.E-24	0.06	0.08	0.56	8.E-04	8
Endothelial → Tumor cells	982	*VIM*	0.5	673	16.00	45.39	6.E-06	-0.08	0.04	0.51	1.E-03	2
Fibroblast → Tumor cells	514	*FOS*	0.3	2581	22.00	128.81	2.E-43	0.06	0.08	0.61	4.E-04	133
Fibroblast → Tumor cells	514	*FN1*	0.3	2581	44.00	128.81	2.E-23	-0.08	0.04	0.46	4.E-04	130
Endothelial → Tumor cells	514	*FOS*	0.3	1354	20.00	119.33	9.E-24	0.06	0.08	0.56	7.E-04	107
Endothelial → Tumor cells	514	*COL4A1*	0.3	1354	22.00	119.33	2.E-21	-0.06	0.07	0.44	8.E-04	101
Fibroblast → Tumor cells	514	*BGN*	0.3	2581	12.00	128.81	7.E-21	-0.02	0.04	0.39	4.E-04	91
Macrophage → Tumor cells	514	*FOS*	0.3	1867	17.00	116.32	1.E-16	0.04	0.04	0.40	6.E-04	88
Smooth muscle → Tumor cells	514	*FN1*	0.3	1236	26.00	110.27	1.E-12	-0.06	0.04	0.46	1.E-03	86
Hepatocyte → Tumor cells	313	*FN1*	0.3	652	41.00	125.60	2.E-07	-0.07	0.05	0.44	1.E-03	84
Fibroblast → Tumor cells	514	*MMP11*	0.3	2581	14.00	128.81	1.E-19	-0.03	0.03	0.37	4.E-04	84
Macrophage → Tumor cells	514	*VIM*	0.3	1867	17.00	116.32	4.E-15	-0.03	0.04	0.39	5.E-04	79
Fibroblast → Tumor cells	313	*FN1*	0.3	700	36.04	48.51	2.E-06	-0.17	0.04	0.42	1.E-03	78
Fibroblast → Tumor cells	982	*FN1*	0.3	742	32.00	43.09	1.E-06	-0.16	0.04	0.47	1.E-03	77
Macrophage → Tumor cells	514	*ITGAX*	0.3	1867	12.68	116.32	3.E-16	-0.03	0.04	0.38	5.E-04	75
Fibroblast → Tumor cells	514	*JUN*	0.3	2581	11.00	128.81	1.E-14	0.02	0.02	0.33	3.E-04	74
Endothelial → Tumor cells	514	*COL4A2*	0.3	1354	17.00	119.33	5.E-12	-0.03	0.04	0.32	7.E-04	68
Fibroblast → Tumor cells	313	*COL1A2*	0.3	700	21.00	48.51	4.E-06	-0.09	0.04	0.33	1.E-03	57
Endothelial → Tumor cells	982	*VIM*	0.3	673	16.00	45.39	6.E-06	-0.08	0.04	0.51	1.E-03	57
Macrophage → Tumor cells	982	*CD74*	0.3	671	28.00	44.47	3.E-04	-0.11	0.02	0.38	1.E-03	48
Tumor cells → B cells	313	*KRT19*	0.3	406	11.00	142.72	1.E-02	0.01	0.02	0.33	3.E-03	25
B cells → Tumor cells	313	*MZB1*	0.3	287	14.28	110.69	3.E-02	-0.03	0.02	0.32	4.E-03	17

Table S7. Genes showing linear dependency of expression on distance between cell types across biopsies using the MERFISH datasets. Same as Table S6, but for the MERFISH dataset. Note that for the “Threshold R^2”, i.e., the Gaussian-smoothed R^2 threshold applied for inclusion, either 0.5 or 0.3 was utilized. All associations shown passed the specified Gaussian-smoothed R^2 thresholds and global FDR correction ($q < 0.05$). For comparability with ExSeq results, maximum pairwise distances in MERFISH were capped at 145 μm (approximate ExSeq FOV diagonal). To minimize potential segmentation artifacts, genes not expressed in at least 20% of single cells of the corresponding primary cell type were excluded based on scRNA-seq data (see Methods).

Dataset	R^2 threshold	Number of genes detected	Notes
ExSeq	0.5	34	
MERFISH	0.5	3	
MERFISH	0.3	20	Relaxed R^2 threshold for broader sensitivity
Shared ExSeq + MERFISH	0.5	1	FOS, Endothelial → Tumor cells
Shared ExSeq + MERFISH	0.5 (ExSeq) / 0.3 (MERFISH)	1	FOS, Endothelial → Tumor cells

Table S8. Genes showing linear dependency of expression on distance between cell types in ExSeq and MERFISH datasets. This table summarizes the number of genes whose expression exhibited a linear relationship with the distance between two cell types (primary and neighbor) in the ExSeq and MERFISH datasets. For MERFISH, two Gaussian-smoothed R^2 thresholds were evaluated: $R^2 \geq 0.5$ (stringent) and $R^2 \geq 0.3$ (lenient). Results from ExSeq and MERFISH analyses were intersected to identify genes detected on both platforms. One gene, *FOS*, met all criteria across ExSeq and MERFISH datasets at both R^2 thresholds and passed scRNA-seq filtering, specifically in the Endothelial → Tumor cell primary-neighbor pair.

Cell type pair	Tissue	Gene	q value	Slope	R^2	Consensus gene
B cells → Tumor cells	59	*CD74*	8.E-03	-0.12	0.02	+
B cells → Tumor cells	59	*KRT19*	2.E-17	0.17	0.13	+
B cells → Tumor cells	59	*S100A9*	5.E-05	0.07	0.04	
Endothelial → Tumor cells	100	*HSPG2*	3.E-02	-0.44	0.01	+
Endothelial → Tumor cells	330	*CDK6*	4.E-03	0.06	0.02	
Endothelial → Tumor cells	330	*HSPB1*	4.E-03	-0.12	0.02	
Endothelial → Tumor cells	330	*KIT*	1.E-04	0.24	0.04	+
Endothelial → Tumor cells	330	*LDB2*	3.E-02	-0.26	0.01	
Endothelial → Tumor cells	330	*LGMN*	2.E-02	-0.15	0.01	
Endothelial → Tumor cells	330	*TMSB4X*	4.E-03	0.11	0.02	
Endothelial → Tumor cells	330	*ZEB1*	3.E-02	-0.07	0.01	
Tumor cells → Endothelial	100	*SDC1*	9.E-10	0.73	0.04	+
Tumor cells → Endothelial	330	*LGMN*	3.E-02	-0.16	0.01	
Tumor cells → Endothelial	330	*TP53*	4.E-03	0.08	0.01	+
Tumor cells → Endothelial	330	*TYMS*	1.E-02	0.19	0.01	
Tumor cells → Macrophage	514	*AURKA*	3.E-04	-0.09	0.03	+
Tumor cells → Macrophage	514	*CRABP2*	3.E-05	-0.14	0.04	+
Tumor cells → Macrophage	514	*PHGDH*	2.E-03	0.09	0.02	
Tumor cells → Macrophage	514	*PRLR*	3.E-02	0.11	0.01	
Tumor cells → Macrophage	514	*S100A4*	5.E-07	-0.15	0.06	+
Tumor cells → Smooth muscle	100	*CAPN13*	3.E-02	-0.06	0.01	
Tumor cells → Smooth muscle	100	*MYO10*	3.E-02	-0.03	0.01	
Tumor cells → Smooth muscle	880	*TFF1*	3.E-02	0.24	0.02	
Tumor cells → $CD3^+$ T cells	100	*CDKN2A*	2.E-03	0.30	0.01	+
Tumor cells → $CD3^+$ T cells	330	*ERBB3*	4.E-02	0.13	0.01	
Tumor cells → $CD3^+$ T cells	330	*MDM2*	1.E-02	0.23	0.01	
Macrophage → Tumor cells	514	*CCL2*	2.E-02	-0.04	0.01	+
Macrophage → Tumor cells	514	*CD40*	4.E-03	-0.06	0.01	+
Macrophage → Tumor cells	514	*COL4A2*	3.E-02	-0.02	0.01	
Macrophage → Tumor cells	514	*JUNB*	2.E-02	0.09	0.01	
$CD3^+$ T cells → Tumor cells	100	*ICOS*	5.E-03	-1.15	0.04	+
$CD3^+$ T cells → Tumor cells	100	*IGHM*	8.E-03	-0.38	0.04	+
$CD3^+$ T cells → Tumor cells	100	*TIMP1*	2.E-02	-0.18	0.03	+
B cells → Tumor cells	59	* *PHGDH*	2.E-19	1.18	0.15	

Table S9. Genes whose expression levels are significantly associated with the number of neighboring cells. This table lists genes whose expression in a given cell type (left of the arrow) is significantly correlated with the number of neighboring cells of another type (right of the arrow) within a 15 μm cell-boundary distance cutoff. Each row represents a statistically significant gene-cell type pair identified through the iterative sampling approach, which corrects for data imbalance in neighbor-count distributions (Methods). Columns include:

- **Cell type pair**: Directional relationship between the primary (X) and neighbor (Y) cell types.
- **Tissue**: Biopsy in which the association was detected.
- **Gene**: Gene symbol.
- ***q* value**: FDR-adjusted *p*-value from the linear regression model.
- **Slope**: Regression coefficient representing the direction and magnitude of the relationship between gene expression and the number of neighboring cells.
- **R^2**: Coefficient of determination for the fitted linear model.
- **Consensus gene**: "+" indicates genes detected by all methods (see Methods section 'Local cellular density modulates gene expression').

Each regression was performed across 1,000 balanced iterations, each containing 10 randomly sampled cells per neighbor-count bin. The reported slopes and R^2 values correspond to the averaged regression fit. Only genes that remained significant ($q < 0.05$) after iterative sampling are shown. Genes were retained only if detected in at least 10% of cells of the primary cell type based on scRNA-seq data. *The gene *PHGDH* was initially excluded during scRNA-seq expression filtering (removal of genes with very low expression levels within the relevant cell type based on matched scRNA-seq data; see Methods section 'Local cellular density modulates gene expression'). However, it was retained because previous studies reported its expression in activated B cells(4).

Cell type	Tissue	Gene	q value	Slope	R^2	Consensus gene
B cells	58	*KRT8*	1.E-02	1.16	0.18	
B cells	58	*LGALS1*	3.E-02	2.84	0.15	
B cells	59	*S100A9*	1.E-03	0.55	0.03	+
B cells	59	*TCL1A*	1.E-02	0.26	0.02	+
B cells	364	*HLA-B*	2.E-02	0.40	0.07	
B cells	364	*S100A4*	4.E-02	0.28	0.06	
Endothelial	59	*ACTA2*	2.E-04	0.29	0.10	+
Endothelial	59	*BRAF*	3.E-02	0.25	0.04	
Endothelial	59	*DCN*	2.E-02	0.60	0.05	
Endothelial	100	*B2M*	7.E-03	0.10	0.02	+
Endothelial	100	*CALCRL*	5.E-03	0.19	0.02	+
Endothelial	100	*CD40*	5.E-03	0.07	0.02	+
Endothelial	330	*KIT*	2.E-03	0.19	0.03	+
Tumor cells	58	*HLA-DRA*	3.E-04	-0.77	0.08	
Tumor cells	58	*LIPE*	3.E-02	0.05	0.03	+
Tumor cells	58	*LYZ*	2.E-03	-0.68	0.06	+
Tumor cells	58	*MZB1*	1.E-03	0.08	0.06	+
Tumor cells	58	*SPDEF*	2.E-03	0.15	0.06	+
Tumor cells	59	*CCNB1*	6.E-05	0.12	0.02	+
Tumor cells	59	*CD40*	3.E-09	0.37	0.05	+
Tumor cells	59	*CDC20*	2.E-16	0.30	0.09	+
Tumor cells	59	*CRABP2*	3.E-10	0.38	0.05	+
Tumor cells	59	*FTL*	6.E-24	2.89	0.13	+
Tumor cells	59	*SOX4*	7.E-05	0.12	0.02	+
Tumor cells	100	*CAPN13*	3.E-02	-0.04	0.01	
Tumor cells	100	*CDKN2A*	4.E-04	0.06	0.01	+
Tumor cells	330	*CCND1*	6.E-07	-0.13	0.03	+
Tumor cells	330	*LGMN*	2.E-04	-0.17	0.02	+
Tumor cells	330	*NR3C1*	3.E-02	-0.07	0.01	+
Tumor cells	330	*TYMS*	4.E-02	0.07	0.01	
Tumor cells	514	*CR2*	3.E-02	0.12	0.02	
Tumor cells	514	*CRABP2*	3.E-04	0.16	0.03	
Tumor cells	514	*PHGDH*	3.E-02	0.10	0.01	
Tumor cells	514	*S100A4*	1.E-04	0.15	0.04	
Tumor cells	982	*MYO5B*	8.E-06	0.46	0.07	+
Fibroblast	100	*CDKN2A*	4.E-04	0.31	0.06	+
Fibroblast	100	*GATA3*	4.E-05	0.57	0.08	+
Macrophage	514	*CCND1*	1.E-05	0.17	0.03	+
Macrophage	514	*COL4A2*	2.E-02	0.11	0.01	+
Macrophage	514	*SIAH2*	4.E-04	0.27	0.02	+
Monocyte	58	*LGALS2*	6.E-03	0.30	0.05	
Smooth muscle	100	*TCF4*	3.E-04	0.37	0.05	+
$CD3^+$ T cells	100	*CD74*	2.E-04	0.46	0.06	+
$CD3^+$ T cells	100	*HLA-C*	5.E-04	0.24	0.05	+
$CD3^+$ T cells	100	*ICOS*	4.E-06	1.69	0.09	+
$CD3^+$ T cells	100	*IGHM*	2.E-03	0.33	0.04	
$CD3^+$ T cells	330	*CD3D*	3.E-02	-0.15	0.01	
$CD3^+$ T cells	364	*CD8A*	7.E-03	0.64	0.03	+

Table S10. Genes whose expression levels are significantly associated with the number of self neighboring cells. Same as Table S9, but here genes whose expression is significantly correlated with the number of neighboring cells of the same type (self) are presented. The analysis was conducted using the iterative sampling approach to correct for data imbalance in neighbor count distributions. Each slope represents the average of 1,000 regressions performed on balanced subsamples, and R^2 is reported from the final fit. Only gene-cell type associations that passed filtering based on scRNA-seq data (≥10% detection within the relevant cell type) are shown.

Cell type pair	Cell triplet	Gene	DESeq2 log fold change	DESeq2 q value
**Endothelial → B cells**	Endothelial → B cells + Tumor cells	*FOS*	-4.7	4.00E-04
**Endothelial → B cells**	Endothelial → B cells + Tumor cells	*TMSB4X*	-4.94	1.00E-04
**Endothelial → Tumor cells**	Endothelial → Tumor cells + Smooth muscle	*CD63*	2.57	3.00E-02
**Endothelial → Tumor cells**	Endothelial → Tumor cells + Smooth muscle	*IFITM3*	2.08	2.00E-02
**Endothelial → Tumor cells**	Endothelial → Tumor cells + Smooth muscle	*ISG20*	2.45	3.00E-02
**Endothelial → Tumor cells**	Endothelial → Tumor cells + Smooth muscle	*TFF3*	2.7	3.00E-02
**Endothelial → Tumor cells**	Endothelial → Tumor cells + Smooth muscle	*TMSB10*	3.33	1.00E-03
**Endothelial → Tumor cells**	Endothelial → Tumor cells + T cells	*CTSL*	-1.51	2.00E-02
**Endothelial → Tumor cells**	Endothelial → Tumor cells + T cells	*HSPB1*	2	1.00E-02
**Endothelial → Tumor cells**	Endothelial → Tumor cells + T cells	*HSPG2*	-1.77	5.00E-02
**Endothelial → Tumor cells**	Endothelial → Tumor cells + T cells	*PECAM1*	-1.69	1.00E-02
**Endothelial → Tumor cells**	Endothelial → Tumor cells + T cells	*TFF3*	2.38	1.00E-02
**Tumor cells → B cells**	Tumor cells → B cells + Fibroblast	*BLVRA*	4.14	1.00E-02
**Tumor cells → B cells**	Tumor cells → B cells + Fibroblast	*CSTB*	3.59	7.00E-03
**Tumor cells → B cells**	Tumor cells → B cells + Fibroblast	*ERBB4*	3.66	7.00E-03
**Tumor cells → B cells**	Tumor cells → B cells + Fibroblast	*KRT10*	3.4	2.00E-02
**Tumor cells → B cells**	Tumor cells → B cells + Fibroblast	*MYB*	3.24	8.00E-03
**Tumor cells → B cells**	Tumor cells → B cells + Fibroblast	*PRLR*	4.19	3.00E-03
**Tumor cells → B cells**	Tumor cells → B cells + Fibroblast	*RPL18*	3.54	4.00E-02
**Tumor cells → B cells**	Tumor cells → B cells + Fibroblast	*TFF3*	5.8	1.00E-04
**Tumor cells → Endothelial**	Tumor cells → Endothelial + Smooth muscle	*ERBB2*	3.84	4.00E-04
**Macrophage → Tumor cells**	Macrophage → Tumor cells + Smooth muscle	*FTL*	-1.61	4.00E-02
**Macrophage → Tumor cells**	Macrophage → Tumor cells + Smooth muscle	*JUN*	-1.85	4.00E-02
**Macrophage → Tumor cells**	Macrophage → Tumor cells + Smooth muscle	*LYZ*	-2.2	1.00E-02
**Macrophage → Tumor cells**	Macrophage → Tumor cells + T cells	*PTPRC*	-1.15	3.00E-02

Table S11. Genes differentially expressed in triplet proximity configurations across cell-type pairs. For each focal (primary) cell type (*A*), gene expression was compared between cells located near a single other cell type (*B*) and those simultaneously proximal (≤1 μm) to two different cell types (*B* and *C*) (see Methods, 'Triplets analysis'). Differential expression was assessed using DESeq2, with *p*-values adjusted for multiple testing. Genes with very low expected expression, defined as those detected in fewer than 20% of single cells of the corresponding type in scRNA-seq data, were excluded. The table reports, for each primary-neighbor cell-type pair and corresponding triplet configuration ($A \rightarrow B$, $A \rightarrow B + C$): (i) the gene symbol, (ii) DESeq2 $\log_2$ fold change, and (iii) DESeq2 *q*-value.

Cell type	Gene	DESeq2 log fold change	DESeq2 *p-value*	Benjamini-Hochberg *q-value* (DESeq2)	permutation-derived *p-value*	Benjamini-Hochberg *q-value* (Permutation)	Log fold change cutoff
B cells	CD19	0.68	3.E-02	4.E-02	1.E-02	2.E-02	0.45
B cells	IGKC	0.70	3.E-02	4.E-02	2.E-02	3.E-02	0.45
B cells	JUNB	1.06	6.E-04	3.E-03	8.E-03	2.E-02	0.45
B cells	NFKBIA	-0.80	6.E-03	2.E-02	2.E-03	8.E-03	0.45
B cells	PABPC1	0.60	6.E-03	2.E-02	1.E-02	2.E-02	0.45
B cells	S100A4	0.72	3.E-02	4.E-02	1.E-03	8.E-03	0.45
Tumor cells	AHR	-1.25	2.E-02	5.E-02	3.E-02	8.E-02	0.45
Tumor cells	AR	1.69	4.E-07	1.E-05	2.E-03	1.E-02	0.45
Tumor cells	CCND1	1.44	1.E-06	2.E-05	1.E-03	8.E-03	0.45
Tumor cells	CD9	1.29	9.E-06	8.E-05	4.E-03	2.E-02	0.45
Tumor cells	CDK6	-1.24	3.E-02	6.E-02	4.E-02	9.E-02	0.45
Tumor cells	CDK7	0.70	3.E-02	6.E-02	7.E-03	2.E-02	0.45
Tumor cells	COL4A5	1.16	4.E-05	3.E-04	7.E-03	2.E-02	0.45
Tumor cells	EIF3E	0.81	3.E-02	7.E-02	4.E-02	8.E-02	0.45
Tumor cells	LTF	1.07	5.E-03	2.E-02	2.E-02	4.E-02	0.45
Tumor cells	LYZ	2.25	1.E-06	2.E-05	6.E-03	2.E-02	0.45
Tumor cells	MAPK3	1.06	5.E-03	2.E-02	7.E-03	2.E-02	0.45
Tumor cells	MT2A	1.23	4.E-03	2.E-02	2.E-02	5.E-02	0.45
Tumor cells	MUC1	0.82	8.E-03	2.E-02	1.E-02	4.E-02	0.45
Tumor cells	NDRG2	1.01	1.E-02	4.E-02	3.E-02	8.E-02	0.45
Tumor cells	NR3C1	1.09	2.E-03	7.E-03	7.E-03	2.E-02	0.45
Tumor cells	ORC6	1.03	8.E-04	4.E-03	4.E-03	2.E-02	0.45
Tumor cells	PABPC1	1.53	1.E-05	8.E-05	1.E-03	8.E-03	0.45
Tumor cells	RPL13	1.00	1.E-03	5.E-03	1.E-02	3.E-02	0.45
Tumor cells	RPSA	1.06	6.E-03	2.E-02	6.E-03	2.E-02	0.45
Tumor cells	S100A14	1.35	1.E-04	5.E-04	1.E-03	8.E-03	0.45
Tumor cells	S100A9	2.10	5.E-06	6.E-05	1.E-03	8.E-03	0.45
Tumor cells	SDC1	1.96	2.E-05	1.E-04	1.E-03	8.E-03	0.45
Tumor cells	TFF3	1.21	2.E-03	7.E-03	6.E-03	2.E-02	0.45
Tumor cells	TSPAN1	1.84	9.E-06	8.E-05	1.E-03	8.E-03	0.45
Fibroblasts	NFKBIA	1.40	4.E-03	5.E-02	8.E-03	5.E-02	0.45
Macrophages	AZGP1	0.87	1.E-02	2.E-02	4.E-02	5.E-02	0.45
Macrophages	C1QB	1.13	8.E-04	3.E-03	3.E-02	4.E-02	0.45
Macrophages	CCL3	1.20	1.E-04	6.E-04	2.E-03	2.E-02	0.45
Macrophages	CD36	0.74	3.E-02	4.E-02	4.E-02	5.E-02	0.45
Macrophages	CDK6	0.93	5.E-03	1.E-02	4.E-02	5.E-02	0.45
Macrophages	COL3A1	1.09	2.E-03	5.E-03	1.E-02	3.E-02	0.45
Macrophages	CXXC5	1.16	2.E-04	9.E-04	2.E-03	2.E-02	0.45
Macrophages	EIF3E	1.13	1.E-03	4.E-03	6.E-03	2.E-02	0.45

Cell type	Gene	DESeq2 log fold change	DESeq2 *p-value*	Benjamini-Hochberg *q-value* (DESeq2)	permutation-derived *p-value*	Benjamini-Hochberg *q-value* (Permutation)	Log fold change cutoff
Macrophages	FCN1	1.19	1.E-02	2.E-02	8.E-02	9.E-02	0.45
Macrophages	FOS	0.90	1.E-02	3.E-02	3.E-02	5.E-02	0.45
Macrophages	HIF1A	1.19	2.E-04	8.E-04	2.E-03	2.E-02	0.45
Macrophages	IGF1R	0.79	2.E-02	3.E-02	4.E-02	5.E-02	0.45
Macrophages	IGKC	0.95	6.E-03	1.E-02	1.E-03	2.E-02	0.45
Macrophages	KRAS	1.69	8.E-05	5.E-04	2.E-03	2.E-02	0.45
Macrophages	KRT8	0.88	1.E-02	3.E-02	4.E-02	5.E-02	0.45
Macrophages	MAPK3	1.38	2.E-04	1.E-03	1.E-02	3.E-02	0.45
Macrophages	MDM2	1.69	4.E-08	2.E-06	1.E-03	2.E-02	0.45
Macrophages	MT2A	1.33	4.E-04	2.E-03	7.E-03	2.E-02	0.45
Macrophages	NR3C1	0.97	5.E-03	1.E-02	1.E-02	3.E-02	0.45
Macrophages	PABPC1	1.13	6.E-03	1.E-02	4.E-02	5.E-02	0.45
Macrophages	PECAM1	0.82	2.E-02	4.E-02	2.E-02	4.E-02	0.45
Macrophages	PLEK	1.63	4.E-04	2.E-03	2.E-02	4.E-02	0.45
Macrophages	RPL13	0.99	2.E-03	6.E-03	1.E-02	3.E-02	0.45
Macrophages	RPSA	1.46	3.E-06	5.E-05	5.E-03	2.E-02	0.45
Macrophages	TCF4	1.29	2.E-03	6.E-03	3.E-02	4.E-02	0.45
Macrophages	TFRC	1.12	2.E-03	5.E-03	2.E-02	4.E-02	0.45
Macrophages	TPM2	1.23	3.E-03	8.E-03	3.E-02	4.E-02	0.45
Macrophages	ZEB1	0.80	2.E-02	3.E-02	4.E-02	5.E-02	0.45

Table S12. Differentially expressed genes in specific cell types associated with bacterial proximity. For each cell type, gene expression was compared between cells containing bacterial transcripts and cells distant from bacteria (>1μm). Statistical significance was evaluated using DESeq2 combined with a permutation-based framework (Methods). Genes were filtered by $\log_2$ fold change cutoff, and both nominal *p*-values (from DESeq2) and permutation-derived *p*-values were corrected for multiple testing. Shown are genes who reached statistical significance ($q < 0.1$) and were expressed in at least 10% of cells of the respective type using scRNA-seq dataset.

Sample	Proximity module results	Neighborhood module results
S1R1	101	164
S1R2	97	195
S2	93	173
S1R1 + S1R2	79	90
S1R1 + S2	13	55
S1R2 + S2	14	55
S1R2 + S1R2 + S2	13	38

Table S13. Summary of results from the Proximity and Neighborhood modules applied to Xenium datasets. The table reports the number of detected gene-cell type associations showing either linear dependence of gene expression on intercellular distance (Proximity module) or on the number of neighboring cells (Neighborhood module). Note that these counts represent gene-cell type associations rather than unique genes, as the same gene can appear in multiple cell type combinations. Results are shown for each biopsy separately: S1R1 (Sample 1 replicate 1), S1R2 (Sample 1 replicate 2, a consecutive section from the same breast cancer biopsy), and S2 (Sample 2, a breast cancer biopsy from a different patient). Rows containing a “+” indicate the overlap between the corresponding samples. A higher overlap is observed between the two consecutive sections from the same biopsy (S1R1 and S1R2) than between samples from different patients. The full results, including the specific gene and cell type combinations detected by each module, are provided in Tables S14-S15.

Cell type pair	Gene	Sample 1 Replicate 1			Sample 1 Replicate 2			Sample 2		
		q value	Slope	Smoothed R^2	q value	Slope	Smoothed R^2	q value	Slope	Smoothed R^2
Tumor cells → Macrophage	*SCD*	<1.E-305	1.E-01	5.E-01	5.E-234	1.E-01	5.E-01	<1.E-305	2.E-01	8.E-01
Tumor cells → Stromal	*SCD*	<1.E-305	1.E-01	5.E-01	1.E-249	1.E-01	4.E-01	<1.E-305	3.E-01	8.E-01
Tumor cells → T cells	*SCD*	<1.E-305	6.E-02	4.E-01	2.E-174	8.E-02	4.E-01	<1.E-305	2.E-01	8.E-01
Tumor cells → Endothelial	*SCD*	9.E-231	7.E-02	3.E-01	3.E-103	7.E-02	3.E-01	<1.E-305	2.E-01	7.E-01
Tumor cells → B cells	*SCD*	7.E-221	6.E-02	4.E-01	5.E-98	6.E-02	4.E-01	4.E-251	1.E-01	6.E-01
Stromal → Tumor cells	*CCDC80*	<1.E-305	4.E-02	6.E-01	4.E-209	4.E-02	5.E-01	4.E-141	2.E-02	3.E-01
Stromal → Tumor cells	*CXCL12*	1.E-231	3.E-02	5.E-01	1.E-151	3.E-02	4.E-01	<1.E-305	2.E-02	5.E-01
Stromal → Tumor cells	*SFRP4*	3.E-189	2.E-02	4.E-01	8.E-107	2.E-02	4.E-01	9.E-167	3.E-02	3.E-01
Stromal → T cells	*CCDC80*	<1.E-305	6.E-02	5.E-01	<1.E-305	6.E-02	6.E-01	2.E-305	4.E-02	3.E-01
Stromal → Endothelial	*CCDC80*	<1.E-305	7.E-02	7.E-01	<1.E-305	6.E-02	6.E-01	5.E-205	4.E-02	3.E-01
Stromal → Endothelial	*MMP2*	<1.E-305	6.E-02	7.E-01	<1.E-305	4.E-02	7.E-01	8.E-281	5.E-02	4.E-01
Stromal → B cells	*ACTA2*	<1.E-305	3.E-02	4.E-01	8.E-275	2.E-02	4.E-01	5.E-199	2.E-02	3.E-01
Stromal → B cells	*POSTN*	<1.E-305	1.E-01	6.E-01	<1.E-305	1.E-01	5.E-01	1.E-240	6.E-02	4.E-01
Stromal → Tumor cells	*CCND1*	6.E-196	-2.E-02	4.E-01	1.E-149	-2.E-02	4.E-01	-	-	-
Stromal → Tumor cells	*KRT7*	<1.E-305	-3.E-02	4.E-01	2.E-302	-3.E-02	4.E-01	-	-	-
Stromal → Macrophage	*ADH1B*	<1.E-305	3.E-02	4.E-01	<1.E-305	5.E-02	4.E-01	-	-	-
Stromal → Macrophage	*CCDC80*	<1.E-305	9.E-02	6.E-01	<1.E-305	7.E-02	6.E-01	-	-	-
Stromal → Macrophage	*CXCL12*	<1.E-305	7.E-02	5.E-01	<1.E-305	8.E-02	6.E-01	-	-	-
Stromal → Macrophage	*FBLN1*	<1.E-305	3.E-02	5.E-01	<1.E-305	3.E-02	5.E-01	-	-	-
Stromal → Macrophage	*LUM*	<1.E-305	1.E-01	5.E-01	<1.E-305	1.E-01	4.E-01	-	-	-
Stromal → Macrophage	*MMP2*	<1.E-305	7.E-02	7.E-01	<1.E-305	5.E-02	6.E-01	-	-	-
Stromal → Macrophage	*PDK4*	<1.E-305	2.E-02	4.E-01	<1.E-305	3.E-02	5.E-01	-	-	-
Stromal → Macrophage	*SFRP1*	<1.E-305	2.E-02	5.E-01	<1.E-305	2.E-02	4.E-01	-	-	-
Stromal → Macrophage	*TOMM7*	<1.E-305	2.E-02	5.E-01	2.E-274	2.E-02	3.E-01	-	-	-
Stromal → Stromal	*ADH1B*	<1.E-305	1.E-01	4.E-01	<1.E-305	3.E-01	6.E-01	-	-	-
Stromal → Stromal	*CCDC80*	<1.E-305	3.E-01	4.E-01	<1.E-305	3.E-01	5.E-01	-	-	-
Stromal → Stromal	*CCND1*	<1.E-305	2.E-01	6.E-01	<1.E-305	8.E-02	3.E-01	-	-	-
Stromal → Stromal	*CXCL12*	<1.E-305	2.E-01	3.E-01	<1.E-305	3.E-01	5.E-01	-	-	-
Stromal → Stromal	*ERBB2*	<1.E-305	6.E-01	7.E-01	<1.E-305	3.E-01	5.E-01	-	-	-
Stromal → Stromal	*FBLN1*	<1.E-305	1.E-01	3.E-01	<1.E-305	1.E-01	4.E-01	-	-	-

Cell type pair	Gene	Sample 1 Replicate 1			Sample 1 Replicate 2			Sample 2		
		q value	Slope	Smoothed R^2	q value	Slope	Smoothed R^2	q value	Slope	Smoothed R^2
Stromal → Stromal	*IGF1*	<1.E-305	8.E-02	3.E-01	<1.E-305	9.E-02	4.E-01	-	-	-
Stromal → Stromal	*LUM*	<1.E-305	5.E-01	4.E-01	<1.E-305	4.E-01	4.E-01	-	-	-
Stromal → Stromal	*MMP2*	<1.E-305	2.E-01	5.E-01	<1.E-305	2.E-01	5.E-01	-	-	-
Stromal → Stromal	*PDK4*	<1.E-305	1.E-01	4.E-01	<1.E-305	2.E-01	6.E-01	-	-	-
Stromal → Stromal	*SFRP1*	<1.E-305	8.E-02	3.E-01	<1.E-305	9.E-02	4.E-01	-	-	-
Stromal → Stromal	*TOMM7*	<1.E-305	1.E-01	5.E-01	<1.E-305	1.E-01	5.E-01	-	-	-
Stromal → T cells	*CCND1*	<1.E-305	3.E-02	5.E-01	<1.E-305	2.E-02	4.E-01	-	-	-
Stromal → T cells	*CXCL12*	2.E-302	4.E-02	4.E-01	<1.E-305	5.E-02	5.E-01	-	-	-
Stromal → T cells	*ERBB2*	<1.E-305	9.E-02	7.E-01	<1.E-305	5.E-02	5.E-01	-	-	-
Stromal → T cells	*FBLN1*	<1.E-305	2.E-02	5.E-01	<1.E-305	2.E-02	6.E-01	-	-	-
Stromal → T cells	*LUM*	<1.E-305	9.E-02	4.E-01	<1.E-305	1.E-01	5.E-01	-	-	-
Stromal → T cells	*MMP2*	<1.E-305	4.E-02	6.E-01	<1.E-305	3.E-02	6.E-01	-	-	-
Stromal → T cells	*PDGFRA*	2.E-207	1.E-02	3.E-01	2.E-162	1.E-02	3.E-01	-	-	-
Stromal → T cells	*POSTN*	<1.E-305	1.E-01	5.E-01	<1.E-305	1.E-01	5.E-01	-	-	-
Stromal → T cells	*TOMM7*	<1.E-305	2.E-02	4.E-01	<1.E-305	2.E-02	4.E-01	-	-	-
Stromal → Endothelial	*CXCL12*	<1.E-305	5.E-02	5.E-01	<1.E-305	6.E-02	5.E-01	-	-	-
Stromal → Endothelial	*FBLN1*	<1.E-305	2.E-02	5.E-01	<1.E-305	2.E-02	5.E-01	-	-	-
Stromal → Endothelial	*LUM*	<1.E-305	8.E-02	4.E-01	3.E-265	7.E-02	3.E-01	-	-	-
Stromal → Endothelial	*POSTN*	<1.E-305	1.E-01	5.E-01	9.E-283	7.E-02	4.E-01	-	-	-
Stromal → B cells	*CCND1*	<1.E-305	2.E-02	4.E-01	1.E-227	1.E-02	3.E-01	-	-	-
Stromal → B cells	*ERBB2*	<1.E-305	6.E-02	5.E-01	<1.E-305	4.E-02	4.E-01	-	-	-
Stromal → B cells	*LUM*	<1.E-305	8.E-02	4.E-01	<1.E-305	8.E-02	4.E-01	-	-	-
Stromal → B cells	*MMP2*	<1.E-305	3.E-02	4.E-01	4.E-292	2.E-02	4.E-01	-	-	-
Stromal → B cells	*PDGFRB*	<1.E-305	2.E-02	4.E-01	3.E-291	2.E-02	4.E-01	-	-	-
Endothelial → Tumor cells	*AQP1*	1.E-69	3.E-02	4.E-01	6.E-41	3.E-02	5.E-01	-	-	-
Endothelial → Tumor cells	*CCND1*	2.E-82	-3.E-02	4.E-01	7.E-62	-2.E-02	5.E-01	-	-	-
Endothelial → Tumor cells	*TCIM*	3.E-78	-2.E-02	5.E-01	2.E-61	-2.E-02	5.E-01	-	-	-
Endothelial → T cells	*ERBB2*	6.E-141	4.E-02	5.E-01	7.E-60	3.E-02	4.E-01	-	-	-
Endothelial → T cells	*LUM*	3.E-68	3.E-02	3.E-01	5.E-55	4.E-02	3.E-01	-	-	-
Endothelial → Endothelial	*LUM*	3.E-144	1.E-01	3.E-01	6.E-127	1.E-01	4.E-01	-	-	-

Cell type pair	Gene	Sample 1 Replicate 1			Sample 1 Replicate 2			Sample 2		
		q value	Slope	Smoothed R^2	q value	Slope	Smoothed R^2	q value	Slope	Smoothed R^2
**Endothelial → B cells**	*CCND1*	3.E-70	2.E-02	4.E-01	2.E-38	2.E-02	4.E-01	-	-	-
**Endothelial → B cells**	*ERBB2*	9.E-69	3.E-02	4.E-01	1.E-40	2.E-02	4.E-01	-	-	-
**Endothelial → B cells**	*POSTN*	2.E-53	3.E-02	3.E-01	4.E-49	3.E-02	4.E-01	-	-	-
**B cells → Macrophage**	*ERBB2*	7.E-121	1.E-01	6.E-01	6.E-53	5.E-02	5.E-01	-	-	-
**B cells → Macrophage**	*ITM2C*	7.E-65	8.E-02	5.E-01	5.E-47	7.E-02	4.E-01	-	-	-
**B cells → Macrophage**	*MZB1*	4.E-81	7.E-02	6.E-01	1.E-60	5.E-02	5.E-01	-	-	-
**B cells → Macrophage**	*SEC11C*	1.E-60	6.E-02	5.E-01	2.E-49	5.E-02	5.E-01	-	-	-
**B cells → Macrophage**	*SLAMF7*	5.E-59	6.E-02	5.E-01	3.E-51	5.E-02	5.E-01	-	-	-
**B cells → Macrophage**	*TENT5C*	4.E-71	9.E-02	5.E-01	2.E-53	8.E-02	5.E-01	-	-	-
**B cells → T cells**	*ERBB2*	5.E-241	9.E-02	7.E-01	3.E-113	4.E-02	6.E-01	-	-	-
**B cells → T cells**	*ITM2C*	8.E-132	8.E-02	5.E-01	2.E-72	6.E-02	4.E-01	-	-	-
**B cells → T cells**	*MZB1*	2.E-143	6.E-02	5.E-01	2.E-83	4.E-02	4.E-01	-	-	-
**B cells → T cells**	*SEC11C*	1.E-141	6.E-02	5.E-01	4.E-73	4.E-02	4.E-01	-	-	-
**B cells → T cells**	*SLAMF7*	1.E-125	6.E-02	5.E-01	4.E-82	5.E-02	4.E-01	-	-	-
**B cells → T cells**	*TENT5C*	1.E-159	9.E-02	5.E-01	2.E-92	7.E-02	4.E-01	-	-	-
**B cells → B cells**	*ERBB2*	5.E-148	8.E-02	6.E-01	2.E-77	5.E-02	5.E-01	-	-	-
**B cells → B cells**	*MZB1*	1.E-54	4.E-02	3.E-01	1.E-44	4.E-02	4.E-01	-	-	-
**B cells → B cells**	*SEC11C*	5.E-54	4.E-02	3.E-01	1.E-35	3.E-02	3.E-01	-	-	-
**B cells → B cells**	*TENT5C*	3.E-54	6.E-02	3.E-01	3.E-42	6.E-02	4.E-01	-	-	-
**Macrophage → Tumor cells**	**APOC1*	1.E-98	-2.E-02	4.E-01	3.E-52	-2.E-02	4.E-01	-	-	-
**Macrophage → Stromal**	**APOC1*	<1.E-305	8.E-02	7.E-01	<1.E-305	9.E-02	7.E-01	-	-	-
**Macrophage → Endothelial**	**APOC1*	4.E-99	3.E-02	4.E-01	1.E-78	3.E-02	4.E-01	-	-	-
**Stromal → Tumor cells**	*PTGDS*	-	-	-	6.E-233	2.E-02	5.E-01	2.E-219	1.E-02	4.E-01

Table S14. Genes showing linear dependency of expression on distance between cell types across biopsies using the Xenium datasets. Each row represents a gene for which expression in a given cell type (primary; left of the arrow) is significantly associated with its distance to another cell type (neighbor; right of the arrow) within a specific tissue sample (Sample 1 Replicate 1, Sample 1 Replicate 2, Sample 2). Note that this table lists only gene-cell type pair associations detected in at least two of the three samples. Columns include:

- **Cell type pair**: Directional pair representing the primary (X) and neighbor (Y) cell types used in the distance-expression analysis.
- **Gene**: Gene symbol.
- **Sample 1 Replicate 1 / Sample 1 Replicate 2 / Sample 2**: Biopsy in which the association was detected.
    - ***q* value**: Global FDR-adjusted *p*-value across all tissues and pairs.
    - **Slope**: Regression slope indicating the direction and strength of the association between distance and gene expression.
    - **Smoothed R^2**: R^2 from regression after applying Gaussian smoothing ($\sigma = 5$) to expression values.

All associations listed passed filtering thresholds, including $q < 0.05$ and Gaussian-smoothed $R^2 \geq 0.3$. To minimize potential segmentation artifacts, genes not expressed in at least 10% of single cells of the corresponding primary cell type were excluded based on scRNA-seq data (see Methods). *The gene *APOC1* was initially excluded during scRNA-seq expression filtering but was retained because previous studies reported its expression in macrophages in cancer tissues, including breast cancer (5, 6).

Cell type pair	Gene	Sample 1 Replicate 1			Sample 1 Replicate 2			Sample 2		
		q value	Slope	R^2	q value	Slope	R^2	q value	Slope	R^2
B cells → B cells	*KRT7*	5.E-58	-6.E-02	5.E-02	2.E-37	-4.E-02	4.E-02	1.E-08	-4.E-03	4.E-03
B cells → Endothelial	*KRT7*	5.E-03	-2.E-02	2.E-03	2.E-03	-2.E-02	3.E-03	2.E-08	-9.E-03	4.E-03
B cells → Macrophage	*KRT7*	1.E-39	-6.E-02	3.E-02	6.E-24	-4.E-02	3.E-02	9.E-06	-7.E-03	3.E-03
B cells → Macrophage	*TIFA*	3.E-14	-3.E-02	1.E-02	5.E-07	-2.E-02	7.E-03	7.E-04	-5.E-03	1.E-03
B cells → Stromal	*KRT7*	5.E-30	-4.E-02	3.E-02	1.E-10	-2.E-02	1.E-02	2.E-04	-5.E-03	2.E-03
B cells → Tumor cells	*KRT7*	2.E-125	1.E-01	1.E-01	1.E-36	9.E-02	4.E-02	1.E-110	7.E-02	6.E-02
B cells → T cells	*KRT7*	2.E-64	-2.E-02	6.E-02	2.E-33	-1.E-02	4.E-02	3.E-26	-5.E-03	1.E-02
B cells → T cells	*TIFA*	3.E-32	-1.E-02	3.E-02	2.E-16	-9.E-03	2.E-02	1.E-18	-4.E-03	1.E-02
Endothelial → B cells	*ADH1B*	9.E-04	-4.E-02	1.E-03	6.E-13	-1.E-01	8.E-03	1.E-17	-1.E-02	5.E-03
Endothelial → B cells	*MYH11*	9.E-07	-4.E-02	3.E-03	1.E-07	-4.E-02	4.E-03	2.E-20	-4.E-02	6.E-03
Endothelial → Endothelial	*ADH1B*	5.E-09	-4.E-02	4.E-03	7.E-37	-2.E-01	2.E-02	3.E-27	-9.E-03	8.E-03
Endothelial → Endothelial	*MYH11*	6.E-17	4.E-02	8.E-03	2.E-19	5.E-02	1.E-02	9.E-87	4.E-02	3.E-02
Endothelial → Endothelial	*TOP2A*	5.E-09	-2.E-02	4.E-03	4.E-03	-9.E-03	1.E-03	4.E-06	-3.E-03	1.E-03
Endothelial → Macrophage	*ADH1B*	1.E-12	-6.E-02	6.E-03	4.E-34	-2.E-01	2.E-02	7.E-41	-2.E-02	1.E-02
Endothelial → Macrophage	*MYH11*	3.E-11	-4.E-02	5.E-03	1.E-14	-5.E-02	9.E-03	1.E-30	-4.E-02	9.E-03
Endothelial → Stromal	*ADH1B*	5.E-14	-4.E-02	7.E-03	6.E-69	-1.E-01	5.E-02	2.E-25	-1.E-02	8.E-03
Endothelial → Stromal	*TOP2A*	3.E-19	-3.E-02	9.E-03	2.E-08	-1.E-02	5.E-03	1.E-02	-3.E-03	5.E-04
Endothelial → Tumor cells	*ADH1B*	1.E-24	-3.E-02	1.E-02	7.E-23	-7.E-02	1.E-02	1.E-29	-2.E-02	9.E-03
Endothelial → Tumor cells	*MYH11*	1.E-21	-3.E-02	1.E-02	6.E-12	-2.E-02	7.E-03	9.E-11	-3.E-02	3.E-03
Endothelial → Tumor cells	*TOP2A*	8.E-36	2.E-02	2.E-02	2.E-58	3.E-02	4.E-02	2.E-31	2.E-02	1.E-02
Endothelial → T cells	*ADH1B*	9.E-05	-1.E-02	2.E-03	4.E-15	-4.E-02	9.E-03	2.E-35	-5.E-03	1.E-02
Endothelial → T cells	*MYH11*	3.E-09	-1.E-02	4.E-03	5.E-06	-1.E-02	3.E-03	6.E-41	-1.E-02	1.E-02
Endothelial → T cells	*TOP2A*	2.E-11	-1.E-02	5.E-03	1.E-05	-6.E-03	3.E-03	1.E-03	-1.E-03	7.E-04
Stromal → B cells	*ADH1B*	5.E-51	-1.E-01	5.E-03	3.E-91	-3.E-01	1.E-02	1.E-29	-3.E-02	4.E-03
Stromal → B cells	*MYH11*	4.E-31	-2.E-02	3.E-03	8.E-23	-2.E-02	3.E-03	2.E-21	-4.E-02	3.E-03
Stromal → Endothelial	*ADH1B*	4.E-48	-1.E-01	5.E-03	2.E-70	-3.E-01	8.E-03	1.E-06	-9.E-03	8.E-04
Stromal → Macrophage	*ADH1B*	6.E-160	-2.E-01	2.E-02	2.E-194	-4.E-01	2.E-02	8.E-124	-5.E-02	2.E-02
Stromal → Macrophage	*MYH11*	5.E-38	-2.E-02	4.E-03	4.E-34	-2.E-02	4.E-03	7.E-35	-4.E-02	5.E-03
Stromal → Stromal	*ADH1B*	7.E-230	-1.E-01	3.E-02	<1.E-305	-3.E-01	6.E-02	1.E-200	-4.E-02	3.E-02
Stromal → Stromal	*MYH11*	2.E-34	-9.E-03	4.E-03	2.E-23	-7.E-03	3.E-03	6.E-03	-6.E-03	2.E-04

Cell type pair	Gene	Sample 1 Replicate 1			Sample 1 Replicate 2			Sample 2		
		q value	Slope	R^2	q value	Slope	R^2	q value	Slope	R^2
Stromal → Tumor cells	*ADH1B*	4.E-103	-9.E-02	1.E-02	9.E-65	-1.E-01	8.E-03	5.E-119	-5.E-02	2.E-02
Stromal → Tumor cells	*MYH11*	4.E-29	-8.E-03	3.E-03	1.E-19	-9.E-03	2.E-03	7.E-19	-3.E-02	3.E-03
Stromal → T cells	*ADH1B*	7.E-88	-8.E-02	1.E-02	5.E-115	-1.E-01	1.E-02	6.E-116	-2.E-02	2.E-02
Stromal → T cells	*MYH11*	2.E-33	-8.E-03	4.E-03	3.E-30	-9.E-03	3.E-03	1.E-36	-2.E-02	5.E-03
Tumor cells → Macrophage	*APOC1*	1.E-104	4.E-02	1.E-02	2.E-49	3.E-02	1.E-02	2.E-50	2.E-02	1.E-02
Tumor cells → Stromal	*APOC1*	1.E-15	1.E-02	2.E-03	1.E-07	9.E-03	2.E-03	9.E-14	9.E-03	4.E-03
Tumor cells → Tumor cells	*APOC1*	3.E-42	-5.E-03	5.E-03	1.E-23	-5.E-03	6.E-03	3.E-24	-8.E-03	7.E-03
Tumor cells → T cells	*APOC1*	1.E-03	7.E-03	3.E-04	7.E-03	6.E-03	5.E-04	7.E-03	3.E-03	5.E-04
B cells → B cells	*TIFA*	5.E-15	-2.E-02	1.E-02	9.E-09	-2.E-02	9.E-03	-	-	-
B cells → Stromal	*TIFA*	4.E-02	5.E-03	9.E-04	2.E-02	7.E-03	2.E-03	-	-	-
Endothelial → B cells	*CENPF*	2.E-07	-2.E-02	3.E-03	2.E-07	-1.E-02	4.E-03	-	-	-
Endothelial → B cells	*IL7R*	3.E-19	5.E-02	9.E-03	2.E-19	5.E-02	1.E-02	-	-	-
Endothelial → B cells	*LPL*	1.E-08	-3.E-02	4.E-03	1.E-14	-7.E-02	9.E-03	-	-	-
Endothelial → B cells	*MRC1*	4.E-03	-1.E-02	1.E-03	2.E-07	-3.E-02	4.E-03	-	-	-
Endothelial → B cells	*TOP2A*	5.E-09	-4.E-02	4.E-03	6.E-08	-3.E-02	5.E-03	-	-	-
Endothelial → Endothelial	*CENPF*	5.E-11	-1.E-02	5.E-03	5.E-04	-6.E-03	2.E-03	-	-	-
Endothelial → Endothelial	*IL7R*	1.E-29	4.E-02	1.E-02	8.E-16	3.E-02	1.E-02	-	-	-
Endothelial → Endothelial	*KIT*	2.E-03	-5.E-03	1.E-03	2.E-05	-9.E-03	3.E-03	-	-	-
Endothelial → Endothelial	*LPL*	3.E-46	-4.E-02	2.E-02	2.E-64	-1.E-01	4.E-02	-	-	-
Endothelial → Endothelial	*MRC1*	2.E-22	-3.E-02	1.E-02	4.E-31	-4.E-02	2.E-02	-	-	-
Endothelial → Macrophage	*IL7R*	3.E-20	4.E-02	1.E-02	6.E-74	8.E-02	5.E-02	-	-	-
Endothelial → Macrophage	*LPL*	4.E-17	-3.E-02	8.E-03	2.E-25	-7.E-02	2.E-02	-	-	-
Endothelial → Macrophage	*MRC1*	2.E-13	-3.E-02	6.E-03	7.E-13	-3.E-02	8.E-03	-	-	-
Endothelial → Stromal	*CENPF*	1.E-16	-1.E-02	8.E-03	2.E-08	-7.E-03	5.E-03	-	-	-
Endothelial → Stromal	*LPL*	2.E-13	-2.E-02	6.E-03	4.E-50	-6.E-02	3.E-02	-	-	-
Endothelial → Stromal	*MRC1*	6.E-04	-8.E-03	1.E-03	7.E-16	-2.E-02	1.E-02	-	-	-
Endothelial → Tumor cells	*CENPF*	7.E-37	1.E-02	2.E-02	2.E-54	2.E-02	4.E-02	-	-	-
Endothelial → Tumor cells	*IL7R*	1.E-32	-2.E-02	2.E-02	1.E-18	-2.E-02	1.E-02	-	-	-
Endothelial → Tumor cells	*MRC1*	3.E-11	-9.E-03	5.E-03	7.E-13	-1.E-02	8.E-03	-	-	-
Endothelial → T cells	*CENPF*	1.E-08	-4.E-03	4.E-03	9.E-06	-3.E-03	3.E-03	-	-	-

Cell type pair	Gene	Sample 1 Replicate 1			Sample 1 Replicate 2			Sample 2		
		q value	Slope	R^2	q value	Slope	R^2	q value	Slope	R^2
Endothelial → T cells	*IL7R*	2.E-188	4.E-02	9.E-02	4.E-224	4.E-02	1.E-01	-	-	-
Endothelial → T cells	*KIT*	8.E-08	4.E-03	3.E-03	9.E-06	4.E-03	3.E-03	-	-	-
Endothelial → T cells	*LPL*	8.E-19	-1.E-02	9.E-03	1.E-19	-2.E-02	1.E-02	-	-	-
Endothelial → T cells	*MRC1*	1.E-11	-8.E-03	5.E-03	5.E-11	-9.E-03	7.E-03	-	-	-
Stromal → B cells	*ANGPT2*	5.E-27	-1.E-02	3.E-03	1.E-26	-1.E-02	3.E-03	-	-	-
Stromal → B cells	*CAVIN2*	9.E-26	-1.E-02	3.E-03	5.E-31	-1.E-02	3.E-03	-	-	-
Stromal → B cells	*TIMP4*	2.E-27	-1.E-02	3.E-03	3.E-40	-2.E-02	5.E-03	-	-	-
Stromal → Endothelial	*ANGPT2*	2.E-10	8.E-03	1.E-03	7.E-11	8.E-03	1.E-03	-	-	-
Stromal → Endothelial	*MYH11*	7.E-44	2.E-02	5.E-03	6.E-60	3.E-02	7.E-03	-	-	-
Stromal → Endothelial	*TIMP4*	1.E-09	-7.E-03	9.E-04	1.E-25	-2.E-02	3.E-03	-	-	-
Stromal → Macrophage	*ANGPT2*	4.E-22	-1.E-02	2.E-03	4.E-24	-1.E-02	3.E-03	-	-	-
Stromal → Macrophage	*CAVIN2*	2.E-48	-1.E-02	5.E-03	4.E-42	-1.E-02	5.E-03	-	-	-
Stromal → Macrophage	*TIMP4*	5.E-53	-1.E-02	6.E-03	9.E-74	-2.E-02	9.E-03	-	-	-
Stromal → Stromal	*ANGPT2*	1.E-25	-5.E-03	3.E-03	3.E-21	-4.E-03	2.E-03	-	-	-
Stromal → Stromal	*CAVIN2*	1.E-48	-7.E-03	5.E-03	1.E-88	-9.E-03	1.E-02	-	-	-
Stromal → Stromal	*TIMP4*	1.E-118	-1.E-02	1.E-02	2.E-233	-2.E-02	3.E-02	-	-	-
Stromal → Tumor cells	*CAVIN2*	1.E-02	-1.E-03	2.E-04	2.E-04	-2.E-03	4.E-04	-	-	-
Stromal → Tumor cells	*TIMP4*	3.E-09	-3.E-03	9.E-04	6.E-13	-6.E-03	1.E-03	-	-	-
Stromal → T cells	*ANGPT2*	2.E-20	-4.E-03	2.E-03	6.E-19	-4.E-03	2.E-03	-	-	-
Stromal → T cells	*CAVIN2*	4.E-27	-5.E-03	3.E-03	4.E-27	-6.E-03	3.E-03	-	-	-
Stromal → T cells	*TIMP4*	2.E-38	-6.E-03	4.E-03	8.E-46	-9.E-03	5.E-03	-	-	-
Stromal → B cells	*AGR3*	3.E-02	-1.E-02	1.E-04	4.E-06	6.E-02	1.E-03	-	-	-
Stromal → B cells	*APOC1*	3.E-03	2.E-02	3.E-04	4.E-02	2.E-02	3.E-04	-	-	-
Tumor cells → Endothelial	*AGR3*	4.E-03	-6.E-03	2.E-04	1.E-02	1.E-02	4.E-04	-	-	-
Tumor cells → Endothelial	*APOC1*	8.E-33	3.E-02	4.E-03	3.E-18	2.E-02	5.E-03	-	-	-
Tumor cells → Macrophage	*AGR3*	3.E-08	-1.E-02	8.E-04	4.E-04	-1.E-02	8.E-04	-	-	-
Tumor cells → Stromal	*AGR3*	3.E-28	1.E-02	3.E-03	6.E-45	3.E-02	1.E-02	-	-	-
Tumor cells → Tumor cells	*AGR3*	6.E-146	-9.E-03	2.E-02	2.E-78	-1.E-02	2.E-02	-	-	-
Tumor cells → Tumor cells	*CEACAM6*	3.E-295	-3.E-02	3.E-02	2.E-225	-4.E-02	6.E-02	-	-	-
Tumor cells → T cells	*AGR3*	2.E-02	4.E-03	1.E-04	3.E-02	7.E-03	3.E-04	-	-	-

Cell type pair	Gene	Sample 1 Replicate 1			Sample 1 Replicate 2			Sample 2		
		q value	Slope	R^2	q value	Slope	R^2	q value	Slope	R^2
B cells → Endothelial	*TIFA*	2.E-02	-1.E-02	1.E-03	-	-	-	2.E-03	-5.E-03	1.E-03
Endothelial → B cells	*ESM1*	1.E-29	-1.E-01	1.E-02	-	-	-	3.E-04	-7.E-03	9.E-04
Endothelial → Endothelial	*ESM1*	3.E-25	-6.E-02	1.E-02	-	-	-	5.E-36	-1.E-02	1.E-02
Endothelial → Endothelial	*SERPINB9*	2.E-03	-9.E-03	1.E-03	-	-	-	8.E-09	5.E-03	2.E-03
Endothelial → Macrophage	*ESM1*	4.E-16	-6.E-02	8.E-03	-	-	-	3.E-03	-5.E-03	6.E-04
Endothelial → Macrophage	*SERPINB9*	2.E-02	8.E-03	6.E-04	-	-	-	6.E-13	1.E-02	4.E-03
Endothelial → Stromal	*ESM1*	4.E-25	-5.E-02	1.E-02	-	-	-	5.E-06	6.E-03	2.E-03
Endothelial → Stromal	*SERPINB9*	1.E-10	-1.E-02	5.E-03	-	-	-	2.E-03	-4.E-03	7.E-04
Endothelial → Tumor cells	*ESM1*	1.E-221	9.E-02	1.E-01	-	-	-	1.E-210	7.E-02	6.E-02
Endothelial → T cells	*ESM1*	2.E-31	-3.E-02	2.E-02	-	-	-	2.E-16	-4.E-03	5.E-03
Endothelial → T cells	*SERPINB9*	4.E-46	2.E-02	2.E-02	-	-	-	2.E-68	8.E-03	2.E-02
Tumor cells → B cells	*LYZ*	3.E-16	6.E-02	2.E-03	-	-	-	6.E-06	5.E-02	1.E-03
Tumor cells → Endothelial	*CEACAM6*	4.E-03	-1.E-02	2.E-04	-	-	-	1.E-08	-7.E-02	2.E-03
Tumor cells → Macrophage	*LYZ*	1.E-200	6.E-02	2.E-02	-	-	-	1.E-289	2.E-01	8.E-02
Tumor cells → Stromal	*LYZ*	6.E-63	3.E-02	7.E-03	-	-	-	4.E-63	8.E-02	2.E-02
Tumor cells → Tumor cells	*LYZ*	7.E-139	-1.E-02	2.E-02	-	-	-	4.E-99	-6.E-02	3.E-02
Tumor cells → T cells	*LYZ*	2.E-45	3.E-02	5.E-03	-	-	-	3.E-33	5.E-02	1.E-02
B cells → B cells	*DUSP5*	-	-	-	5.E-28	-5.E-02	3.E-02	2.E-08	-4.E-03	4.E-03
B cells → Macrophage	*DUSP5*	-	-	-	4.E-30	-8.E-02	3.E-02	8.E-03	-5.E-03	9.E-04
B cells → T cells	*DUSP5*	-	-	-	1.E-43	-3.E-02	5.E-02	2.E-29	-6.E-03	2.E-02
Tumor cells → B cells	*TOP2A*	-	-	-	2.E-03	-1.E-01	6.E-04	4.E-05	-1.E-01	1.E-03
Tumor cells → Endothelial	*TOP2A*	-	-	-	1.E-03	-4.E-02	7.E-04	4.E-15	-1.E-01	4.E-03
Tumor cells → Macrophage	*CENPF*	-	-	-	6.E-05	-3.E-02	1.E-03	1.E-23	-3.E-02	7.E-03
Tumor cells → Macrophage	*TOP2A*	-	-	-	5.E-06	-5.E-02	1.E-03	3.E-42	-2.E-01	1.E-02
Tumor cells → Stromal	*CENPF*	-	-	-	2.E-02	-1.E-02	3.E-04	9.E-21	-2.E-02	6.E-03
Tumor cells → Stromal	*TOP2A*	-	-	-	8.E-13	-5.E-02	3.E-03	3.E-36	-1.E-01	1.E-02
Tumor cells → Tumor cells	*CENPF*	-	-	-	6.E-05	7.E-03	9.E-04	2.E-03	5.E-03	7.E-04
Tumor cells → Tumor cells	*TOP2A*	-	-	-	5.E-11	1.E-02	2.E-03	2.E-16	5.E-02	4.E-03
Tumor cells → T cells	*CENPF*	-	-	-	7.E-03	-2.E-02	5.E-04	1.E-09	-1.E-02	3.E-03
Tumor cells → T cells	*TOP2A*	-	-	-	2.E-04	-4.E-02	1.E-03	3.E-20	-8.E-02	6.E-03

Cell type pair	Gene	Sample 1 Replicate 1			Sample 1 Replicate 2			Sample 2		
		q value	Slope	R^2	q value	Slope	R^2	q value	Slope	R^2
T cells → B cells	*PRF1*	-	-	-	1.E-08	-7.E-03	3.E-03	1.E-13	-5.E-03	2.E-03
T cells → Endothelial	*PRF1*	-	-	-	2.E-02	-5.E-03	6.E-04	3.E-02	1.E-03	2.E-04
T cells → Macrophage	*PRF1*	-	-	-	3.E-04	-5.E-03	1.E-03	1.E-15	-6.E-03	2.E-03
T cells → T cells	*PRF1*	-	-	-	1.E-13	-2.E-03	5.E-03	6.E-30	-2.E-03	4.E-03

Table S15. Genes whose expression levels are significantly associated with the number of neighboring cells on Xenium datasets. This table lists genes whose expression in a given cell type (left of the arrow) is significantly correlated with the number of neighboring cells of another type (right of the arrow). Each row represents a statistically significant gene-cell type pair. Note that this table lists only gene-cell type pair associations detected in at least two of the three samples. Columns include:

- **Cell type pair**: Directional relationship between the primary (X) and neighbor (Y) cell types.
- **Gene**: Gene symbol.
- **Sample 1 Replicate 1 / Sample 1 Replicate 2 / Sample 2**: Biopsy in which the association was detected.
    - ***q* value**: FDR-adjusted *p*-value from the linear regression model.
    - **Slope**: Regression coefficient representing the direction and magnitude of the relationship between gene expression and the number of neighboring cells.
    - **R^2**: Coefficient of determination for the fitted linear model.

Genes were retained only if detected in at least 10% of cells of the primary cell type based on scRNA-seq data.

Sample	Filtered results
S1R1	174
S1R2	119
S2	184
S1R1 + S1R2	103
S1R1 + S2	75
S1R2 + S2	62
S1R2 + S1R2 + S2	58

Table S16. Summary of results for genes differentially expressed in triplet proximity configurations across cell-type pairs on Xenium datasets. Results are shown for each biopsy separately: S1R1 (Sample 1 replicate 1), S1R2 (Sample 1 replicate 2, a consecutive section from the same breast cancer biopsy), and S2 (Sample 2, a breast cancer biopsy from a different patient). Rows containing a "+" indicate the overlap between the corresponding samples. A higher overlap is observed between the two consecutive sections from the same biopsy (S1R1 and S1R2) than between samples from different patients. The full results, including the specific gene and cell type combinations detected by each module, are provided in Tables S17.

Cell type pair	Cell triplet	Gene	Sample 1 Replicate 1		Sample 1 Replicate 2		Sample 2	
			q value	Log fold change	q value	Log fold change	q value	Log fold change
B cells → Macrophage	B cells → Macrophage + T cells	*ITM2C*	1.E-09	7.E-01	4.E-06	6.E-01	4.E-08	4.E-01
B cells → Macrophage	B cells → Macrophage + T cells	*MZB1*	2.E-11	7.E-01	3.E-05	5.E-01	4.E-05	4.E-01
B cells → Macrophage	B cells → Macrophage + T cells	*PRDM1*	3.E-05	5.E-01	3.E-06	6.E-01	2.E-03	3.E-01
B cells → Macrophage	B cells → Macrophage + T cells	*SEC11C*	3.E-07	5.E-01	5.E-07	6.E-01	1.E-08	5.E-01
B cells → Macrophage	B cells → Macrophage + T cells	*SLAMF7*	2.E-11	7.E-01	1.E-06	6.E-01	4.E-08	4.E-01
B cells → Macrophage	B cells → Macrophage + T cells	*TENT5C*	1.E-13	9.E-01	2.E-07	7.E-01	2.E-10	5.E-01
B cells → Stromal	B cells → Stromal + T cells	*BANK1*	2.E-08	-5.E-01	3.E-22	-8.E-01	4.E-02	-2.E-01
B cells → Stromal	B cells → Stromal + T cells	*CCPG1*	2.E-11	5.E-01	5.E-09	5.E-01	9.E-03	2.E-01
B cells → Stromal	B cells → Stromal + T cells	*ITM2C*	5.E-21	8.E-01	4.E-13	7.E-01	1.E-09	4.E-01
B cells → Stromal	B cells → Stromal + T cells	*MS4A1*	2.E-13	-6.E-01	8.E-16	-7.E-01	1.E-03	-3.E-01
B cells → Stromal	B cells → Stromal + T cells	*MZB1*	1.E-20	8.E-01	9.E-17	8.E-01	4.E-08	4.E-01
B cells → Stromal	B cells → Stromal + T cells	*PECAM1*	1.E-09	5.E-01	4.E-13	7.E-01	2.E-03	3.E-01
B cells → Stromal	B cells → Stromal + T cells	*PTPRC*	3.E-06	-4.E-01	8.E-09	-5.E-01	9.E-07	-3.E-01
B cells → Stromal	B cells → Stromal + T cells	*SEC11C*	3.E-21	8.E-01	1.E-12	7.E-01	2.E-10	4.E-01
B cells → Stromal	B cells → Stromal + T cells	*SLAMF7*	3.E-19	8.E-01	1.E-24	1.E+00	3.E-09	4.E-01
B cells → Stromal	B cells → Stromal + T cells	*TENT5C*	1.E-25	1.E+00	4.E-20	9.E-01	4.E-09	5.E-01
Endothelial → B cells	Endothelial → B cells + T cells	*CAV1*	2.E-02	6.E-01	2.E-02	6.E-01	7.E-06	5.E-01
Endothelial → Macrophage	Endothelial → Macrophage + T cells	*IL7R*	1.E-04	-6.E-01	6.E-06	-8.E-01	1.E-03	-3.E-01
Endothelial → Macrophage	Endothelial → Macrophage + T cells	*POSTN*	5.E-07	9.E-01	2.E-04	7.E-01	1.E-02	3.E-01
Endothelial → Stromal	Endothelial → Stromal + T cells	*ACTA2*	1.E-06	6.E-01	8.E-04	5.E-01	5.E-09	5.E-01
Endothelial → Stromal	Endothelial → Stromal + T cells	*CAV1*	1.E-05	5.E-01	1.E-02	4.E-01	5.E-04	3.E-01
Endothelial → Stromal	Endothelial → Stromal + T cells	*LUM*	4.E-02	3.E-01	3.E-05	6.E-01	6.E-09	4.E-01
Endothelial → Stromal	Endothelial → Stromal + T cells	*POSTN*	8.E-04	5.E-01	2.E-06	8.E-01	2.E-08	5.E-01
Stromal → B cells	Stromal → B cells + T cells	*CCDC80*	7.E-12	5.E-01	2.E-09	5.E-01	2.E-25	6.E-01
Stromal → B cells	Stromal → B cells + T cells	*ERBB2*	7.E-13	6.E-01	6.E-10	6.E-01	3.E-02	2.E-01
Stromal → B cells	Stromal → B cells + T cells	*FBLN1*	9.E-03	3.E-01	1.E-05	4.E-01	3.E-10	4.E-01
Stromal → B cells	Stromal → B cells + T cells	*LUM*	5.E-08	4.E-01	4.E-08	4.E-01	2.E-18	5.E-01
Stromal → B cells	Stromal → B cells + T cells	*MMP2*	5.E-10	5.E-01	2.E-02	3.E-01	6.E-17	5.E-01
Stromal → B cells	Stromal → B cells + T cells	*POSTN*	3.E-14	7.E-01	1.E-15	7.E-01	2.E-19	7.E-01
Stromal → B cells	Stromal → B cells + T cells	*SFRP1*	1.E-04	4.E-01	2.E-06	5.E-01	2.E-10	4.E-01

Cell type pair	Cell triplet	Gene	Sample 1 Replicate 1		Sample 1 Replicate 2		Sample 2	
			q value	Log fold change	q value	Log fold change	q value	Log fold change
**Stromal → B cells**	Stromal → B cells + T cells	*SFRP4*	1.E-06	5.E-01	4.E-09	6.E-01	6.E-18	6.E-01
**Stromal → Endothelial**	Stromal → Endothelial + T cells	*ACTA2*	2.E-04	5.E-01	1.E-05	7.E-01	1.E-11	5.E-01
**Stromal → Endothelial**	Stromal → Endothelial + T cells	*LUM*	1.E-06	5.E-01	7.E-06	5.E-01	1.E-11	5.E-01
**Stromal → Endothelial**	Stromal → Endothelial + T cells	*PDGFRB*	5.E-06	5.E-01	3.E-03	4.E-01	2.E-06	4.E-01
**Stromal → Endothelial**	Stromal → Endothelial + T cells	*POSTN*	5.E-06	5.E-01	6.E-08	7.E-01	3.E-21	9.E-01
**Stromal → Endothelial**	Stromal → Endothelial + T cells	*SFRP4*	2.E-02	4.E-01	2.E-02	5.E-01	2.E-02	3.E-01
**Stromal → Endothelial**	Stromal → Endothelial + Tumor cells	*EPCAM*	2.E-19	-1.E+00	2.E-07	-1.E+00	4.E-02	-7.E-01
**Stromal → Endothelial**	Stromal → Endothelial + Tumor cells	*KRT7*	2.E-19	-1.E+00	3.E-09	-1.E+00	4.E-05	-1.E+00
**Stromal → Macrophage**	Stromal → Macrophage + T cells	*ACTA2*	7.E-11	5.E-01	2.E-10	5.E-01	2.E-17	4.E-01
**Stromal → Macrophage**	Stromal → Macrophage + T cells	*CCDC80*	4.E-12	4.E-01	2.E-07	4.E-01	5.E-29	5.E-01
**Stromal → Macrophage**	Stromal → Macrophage + T cells	*ENAH*	1.E-05	3.E-01	7.E-03	3.E-01	8.E-03	2.E-01
**Stromal → Macrophage**	Stromal → Macrophage + T cells	*ERBB2*	5.E-20	6.E-01	8.E-15	6.E-01	1.E-07	3.E-01
**Stromal → Macrophage**	Stromal → Macrophage + T cells	*KRT7*	9.E-09	4.E-01	3.E-07	4.E-01	4.E-05	3.E-01
**Stromal → Macrophage**	Stromal → Macrophage + T cells	*LUM*	4.E-13	4.E-01	2.E-10	4.E-01	8.E-32	5.E-01
**Stromal → Macrophage**	Stromal → Macrophage + T cells	*MMP2*	9.E-08	3.E-01	3.E-05	3.E-01	1.E-36	6.E-01
**Stromal → Macrophage**	Stromal → Macrophage + T cells	*PDGFRB*	3.E-07	4.E-01	4.E-04	3.E-01	4.E-08	3.E-01
**Stromal → Macrophage**	Stromal → Macrophage + T cells	*POSTN*	4.E-22	6.E-01	2.E-13	5.E-01	6.E-49	9.E-01
**Stromal → Macrophage**	Stromal → Macrophage + T cells	*RUNX1*	4.E-05	3.E-01	4.E-02	2.E-01	4.E-10	3.E-01
**Stromal → Macrophage**	Stromal → Macrophage + T cells	*SFRP1*	3.E-04	3.E-01	5.E-07	4.E-01	6.E-15	4.E-01
**Stromal → Macrophage**	Stromal → Macrophage + T cells	*SFRP4*	2.E-08	5.E-01	1.E-06	4.E-01	8.E-09	3.E-01
**Stromal → Macrophage**	Stromal → Macrophage + Tumor cells	*CCDC80*	8.E-07	9.E-01	6.E-03	9.E-01	1.E-02	7.E-01
**Stromal → Macrophage**	Stromal → Macrophage + Tumor cells	*CXCL12*	5.E-06	9.E-01	5.E-04	1.E+00	1.E-05	1.E+00
**Stromal → Macrophage**	Stromal → Macrophage + Tumor cells	*KRT7*	4.E-15	-1.E+00	1.E-09	-1.E+00	3.E-06	-8.E-01
**Stromal → Macrophage**	Stromal → Macrophage + Tumor cells	*PTGDS*	3.E-07	1.E+00	4.E-04	1.E+00	8.E-05	1.E+00
**Stromal → T cells**	Stromal → T cells + Tumor cells	*CXCL12*	5.E-03	8.E-01	2.E-04	1.E+00	2.E-04	9.E-01
**Stromal → T cells**	Stromal → T cells + Tumor cells	*ERBB2*	2.E-08	-1.E+00	4.E-03	-8.E-01	3.E-03	-6.E-01
**Stromal → T cells**	Stromal → T cells + Tumor cells	*KRT7*	8.E-28	-1.E+00	4.E-07	-1.E+00	4.E-02	-5.E-01
**Stromal → T cells**	Stromal → T cells + Tumor cells	*PTGDS*	1.E-03	1.E+00	7.E-04	2.E+00	1.E-03	8.E-01
**B cells → Macrophage**	B cells → Macrophage + T cells	*BANK1*	7.E-04	-4.E-01	2.E-03	-4.E-01	-	-
**B cells → Macrophage**	B cells → Macrophage + T cells	*CCPG1*	1.E-04	4.E-01	4.E-04	4.E-01	-	-

Cell type pair	Cell triplet	Gene	Sample 1 Replicate 1		Sample 1 Replicate 2		Sample 2	
			q value	Log fold change	q value	Log fold change	q value	Log fold change
**B cells → Macrophage**	B cells → Macrophage + T cells	*ERBB2*	7.E-15	7.E-01	5.E-02	3.E-01	-	-
**B cells → Macrophage**	B cells → Macrophage + T cells	*ERN1*	6.E-07	5.E-01	2.E-02	4.E-01	-	-
**B cells → Macrophage**	B cells → Macrophage + T cells	*PECAM1*	2.E-06	5.E-01	2.E-04	5.E-01	-	-
**B cells → Macrophage**	B cells → Macrophage + T cells	*SEC24A*	2.E-04	4.E-01	2.E-02	3.E-01	-	-
**B cells → Macrophage**	B cells → Macrophage + T cells	*TPD52*	3.E-10	5.E-01	1.E-05	4.E-01	-	-
**B cells → Stromal**	B cells → Stromal + T cells	*DERL3*	1.E-04	4.E-01	5.E-03	4.E-01	-	-
**B cells → Stromal**	B cells → Stromal + T cells	*DUSP5*	5.E-06	4.E-01	4.E-03	4.E-01	-	-
**B cells → Stromal**	B cells → Stromal + T cells	*ERBB2*	5.E-18	7.E-01	1.E-08	5.E-01	-	-
**B cells → Stromal**	B cells → Stromal + T cells	*ERN1*	4.E-13	6.E-01	1.E-12	7.E-01	-	-
**B cells → Stromal**	B cells → Stromal + T cells	*MDM2*	4.E-06	4.E-01	3.E-03	3.E-01	-	-
**B cells → Stromal**	B cells → Stromal + T cells	*PRDM1*	1.E-17	7.E-01	3.E-09	6.E-01	-	-
**B cells → Stromal**	B cells → Stromal + T cells	*RAB30*	3.E-07	4.E-01	3.E-03	3.E-01	-	-
**B cells → Stromal**	B cells → Stromal + T cells	*SEC24A*	5.E-12	6.E-01	3.E-10	6.E-01	-	-
**B cells → Stromal**	B cells → Stromal + T cells	*SELL*	3.E-04	-3.E-01	6.E-04	-4.E-01	-	-
**B cells → Stromal**	B cells → Stromal + T cells	*TPD52*	1.E-10	4.E-01	8.E-10	5.E-01	-	-
**B cells → Stromal**	B cells → Stromal + T cells	*TRIB1*	5.E-05	4.E-01	3.E-05	5.E-01	-	-
**B cells → Stromal**	B cells → Stromal + T cells	*VOPP1*	1.E-06	4.E-01	4.E-04	3.E-01	-	-
**Endothelial → Macrophage**	Endothelial → Macrophage + T cells	*CCND1*	1.E-03	6.E-01	8.E-03	5.E-01	-	-
**Endothelial → Macrophage**	Endothelial → Macrophage + T cells	*ERBB2*	1.E-02	5.E-01	6.E-06	8.E-01	-	-
**Endothelial → Macrophage**	Endothelial → Macrophage + Tumor cells	*ESM1*	3.E-06	-1.E+00	3.E-02	-2.E+00	-	-
**Endothelial → Stromal**	Endothelial → Stromal + T cells	*ERBB2*	2.E-03	4.E-01	3.E-03	5.E-01	-	-
**Endothelial → Stromal**	Endothelial → Stromal + Tumor cells	*ERBB2*	8.E-03	-6.E-01	3.E-02	-7.E-01	-	-
**Stromal → B cells**	Stromal → B cells + T cells	*CCND1*	1.E-06	4.E-01	7.E-04	4.E-01	-	-
**Stromal → B cells**	Stromal → B cells + T cells	*IGF1*	4.E-04	3.E-01	4.E-04	4.E-01	-	-
**Stromal → Endothelial**	Stromal → Endothelial + T cells	*CAV1*	6.E-03	4.E-01	9.E-05	6.E-01	-	-
**Stromal → Endothelial**	Stromal → Endothelial + T cells	*CCND1*	4.E-03	4.E-01	2.E-03	4.E-01	-	-
**Stromal → Endothelial**	Stromal → Endothelial + T cells	*ERBB2*	9.E-07	6.E-01	4.E-06	6.E-01	-	-
**Stromal → Endothelial**	Stromal → Endothelial + Tumor cells	*GATA3*	7.E-06	-7.E-01	3.E-06	-9.E-01	-	-
**Stromal → Endothelial**	Stromal → Endothelial + Tumor cells	*KRT8*	3.E-11	-9.E-01	4.E-03	-8.E-01	-	-
**Stromal → Macrophage**	Stromal → Macrophage + T cells	*ADAM9*	6.E-03	2.E-01	3.E-02	2.E-01	-	-

Cell type pair	Cell triplet	Gene	Sample 1 Replicate 1		Sample 1 Replicate 2		Sample 2	
			q value	Log fold change	q value	Log fold change	q value	Log fold change
Stromal → Macrophage	Stromal → Macrophage + T cells	*ANKRD30A*	3.E-02	2.E-01	2.E-02	3.E-01	-	-
Stromal → Macrophage	Stromal → Macrophage + T cells	*CCND1*	3.E-10	4.E-01	3.E-09	4.E-01	-	-
Stromal → Macrophage	Stromal → Macrophage + T cells	*EPCAM*	1.E-04	3.E-01	3.E-02	3.E-01	-	-
Stromal → Macrophage	Stromal → Macrophage + T cells	*IGF1*	2.E-03	2.E-01	3.E-05	3.E-01	-	-
Stromal → Macrophage	Stromal → Macrophage + T cells	*PDK4*	5.E-08	4.E-01	2.E-07	4.E-01	-	-
Stromal → Macrophage	Stromal → Macrophage + T cells	*ZEB1*	2.E-03	3.E-01	2.E-02	2.E-01	-	-
Stromal → Macrophage	Stromal → Macrophage + Tumor cells	*CCND1*	1.E-04	-6.E-01	9.E-05	-8.E-01	-	-
Stromal → Macrophage	Stromal → Macrophage + Tumor cells	*EPCAM*	1.E-13	-1.E+00	9.E-13	-1.E+00	-	-
Stromal → Macrophage	Stromal → Macrophage + Tumor cells	*GATA3*	6.E-09	-8.E-01	1.E-05	-9.E-01	-	-
Stromal → Macrophage	Stromal → Macrophage + Tumor cells	*KRT8*	7.E-11	-9.E-01	7.E-06	-9.E-01	-	-
Stromal → T cells	Stromal → T cells + Tumor cells	*EPCAM*	6.E-20	-1.E+00	1.E-05	-1.E+00	-	-
Stromal → T cells	Stromal → T cells + Tumor cells	*GATA3*	5.E-12	-1.E+00	2.E-02	-7.E-01	-	-
Stromal → T cells	Stromal → T cells + Tumor cells	*KRT8*	8.E-06	-9.E-01	4.E-02	-8.E-01	-	-
B cells → Macrophage	B cells → Macrophage + T cells	*MS4A1*	1.E-06	-5.E-01	-	-	4.E-07	-4.E-01
B cells → Macrophage	B cells → Macrophage + T cells	*PTPRC*	1.E-05	-4.E-01	-	-	2.E-02	-2.E-01
Endothelial → Macrophage	Endothelial → Macrophage + T cells	*LUM*	3.E-03	5.E-01	-	-	4.E-02	2.E-01
Endothelial → Stromal	Endothelial → Stromal + T cells	*CXCL12*	4.E-02	3.E-01	-	-	4.E-06	4.E-01
Stromal → B cells	Stromal → B cells + Tumor cells	*KRT7*	5.E-06	-2.E+00	-	-	5.E-04	-1.E+00
Stromal → Endothelial	Stromal → Endothelial + T cells	*CCDC80*	1.E-02	3.E-01	-	-	1.E-07	4.E-01
Stromal → Endothelial	Stromal → Endothelial + Tumor cells	*CCDC80*	1.E-05	9.E-01	-	-	3.E-02	9.E-01
Stromal → Endothelial	Stromal → Endothelial + Tumor cells	*CXCL12*	9.E-03	6.E-01	-	-	8.E-04	1.E+00
Stromal → Endothelial	Stromal → Endothelial + Tumor cells	*ERBB2*	7.E-03	-5.E-01	-	-	4.E-03	-8.E-01
Stromal → Endothelial	Stromal → Endothelial + Tumor cells	*PTGDS*	7.E-04	9.E-01	-	-	8.E-03	1.E+00
Stromal → Macrophage	Stromal → Macrophage + T cells	*CTTN*	6.E-04	3.E-01	-	-	7.E-05	2.E-01
Stromal → Macrophage	Stromal → Macrophage + T cells	*FBLN1*	6.E-03	2.E-01	-	-	5.E-12	3.E-01
Stromal → Macrophage	Stromal → Macrophage + T cells	*FGL2*	3.E-02	2.E-01	-	-	9.E-03	2.E-01
Stromal → Macrophage	Stromal → Macrophage + T cells	*LYZ*	3.E-03	2.E-01	-	-	2.E-07	3.E-01
Stromal → Macrophage	Stromal → Macrophage + T cells	*MDM2*	4.E-03	2.E-01	-	-	3.E-02	2.E-01
Stromal → Macrophage	Stromal → Macrophage + T cells	*ZEB2*	5.E-02	2.E-01	-	-	3.E-04	2.E-01
Stromal → T cells	Stromal → T cells + Tumor cells	*CCDC80*	9.E-03	7.E-01	-	-	6.E-04	8.E-01

Cell type pair	Cell triplet	Gene	Sample 1 Replicate 1		Sample 1 Replicate 2		Sample 2	
			q value	Log fold change	q value	Log fold change	q value	Log fold change
**Endothelial → B cells**	Endothelial → B cells + T cells	*CXCL12*	-	-	4.E-02	6.E-01	3.E-07	6.E-01
**Endothelial → Macrophage**	Endothelial → Macrophage + Stromal	*VWF*	-	-	1.E-02	5.E-01	2.E-04	6.E-01
**Endothelial → Macrophage**	Endothelial → Macrophage + T cells	*IL7R*	-	-	6.E-06	-8.E-01	1.E-03	-3.E-01
**Stromal → B cells**	Stromal → B cells + T cells	*ACTA2*	-	-	3.E-03	3.E-01	3.E-08	4.E-01

Table S17. Genes differentially expressed in triplet proximity configurations across cell-type pairs in Xenium datasets. For each focal (primary) cell type (*A*), gene expression was compared between cells located near a single other cell type (*B*) and those simultaneously proximal (≤10 μm) to two different cell types (*B* and *C*) (see Methods, 'Triplets analysis'). Differential expression was assessed using DESeq2. Genes with very low expected expression, defined as those detected in fewer than 10% of single cells of the corresponding type in scRNA-seq data, were excluded. The table reports, for each primary-neighbor cell-type pair and corresponding triplet configuration ($A \rightarrow B$, $A \rightarrow B + C$): (i) the gene symbol, and for each tissue: (ii) *q*-value, and (iii) DESeq2 $\log_2$ fold change.

Cell type pair	Tissue	Gene	# Interacting cells	98th percentile expression	98th percentile distance (μm)	q value	Slope	R^2 (original)	R^2 (Gaussian)	R^2 (permuted mean)
eL2_3 cells → PV cells	Light Rep1	*Egr1*	96	251.30	140.66	9.E-03	-0.74	0.19	0.89	1.E-02
eL2_3 cells → PV cells	Light Rep1	*Nrn1*	96	31.00	140.66	9.E-03	-0.10	0.19	0.92	1.E-02
eL2_3 cells → PV cells	Light Rep1	*Tnfaip6*	96	23.00	140.66	3.E-02	-0.05	0.15	0.68	1.E-02
eL2_3 cells → Endo cells	Light Rep1	*Nov*	147	10.08	108.53	9.E-03	0.04	0.14	0.75	1.E-02
eL2_3 cells → Endo cells	Light Rep1	*Osgin2*	147	19.08	108.53	4.E-02	-0.06	0.09	0.59	6.E-03
eL6 cells → eL2_3 cells	Light Rep1	*Rgs12*	136	21.90	142.69	2.E-02	0.05	0.12	0.71	8.E-03
eL6 cells → eL6 cells	Light Rep1	*Arl4d*	150	12.02	54.26	4.E-02	0.10	0.09	0.49	8.E-03
eL6 cells → eL6 cells	Light Rep1	*Arx*	150	124.14	54.26	9.E-03	0.98	0.14	0.52	6.E-03
eL6 cells → eL6 cells	Light Rep1	*Bcl6*	150	20.08	54.26	3.E-02	0.18	0.10	0.54	8.E-03
eL6 cells → eL6 cells	Light Rep1	*eRNA2*	150	18.06	54.26	2.E-02	0.18	0.11	0.81	7.E-03
eL6 cells → eL6 cells	Light Rep1	*eRNA3*	150	128.32	54.26	2.E-02	1.11	0.11	0.48	6.E-03
eL6 cells → eL6 cells	Light Rep1	*Myl4*	150	19.24	54.26	4.E-05	0.32	0.21	0.83	7.E-03
eL6 cells → eL6 cells	Light Rep1	*Nectin3*	150	15.02	54.26	9.E-03	0.15	0.13	0.69	6.E-03
eL6 cells → eL6 cells	Light Rep1	*Nptx2*	150	10.04	54.26	1.E-02	0.13	0.12	0.62	7.E-03
eL6 cells → eL6 cells	Light Rep1	*Nrn1*	150	29.02	54.26	3.E-02	0.25	0.10	0.53	7.E-03
eL6 cells → eL6 cells	Light Rep1	*Otof*	150	49.00	54.26	3.E-02	0.42	0.10	0.48	6.E-03
eL6 cells → eL6 cells	Light Rep1	*Prok2*	150	90.06	54.26	2.E-02	0.74	0.11	0.69	7.E-03
eL6 cells → eL6 cells	Light Rep1	*Syt17*	150	17.02	54.26	4.E-02	0.14	0.09	0.57	7.E-03
eL5 cells → eL2_3 cells	Light Rep1	*Pcp4*	71	51.80	140.14	4.E-02	0.20	0.18	0.91	1.E-02
eL5 cells → eL4 cells	Light Rep1	*Egr1*	85	227.00	139.67	4.E-02	-0.64	0.15	0.67	1.E-02
eL5 cells → eL4 cells	Light Rep1	*Pcp4*	85	81.24	139.67	3.E-02	0.19	0.16	0.71	1.E-02
Oligo cells → Oligo cells	Light Rep1	*Pcdhgc3*	47	44.08	134.43	4.E-02	0.16	0.27	0.86	2.E-02
eL4 cells → eL2_3 cells	Light Rep1	*Pcp4*	129	11.44	78.04	3.E-02	0.06	0.11	0.68	7.E-03
eL4 cells → NPY cells	Light Rep1	*Ngb*	125	15.00	134.99	2.E-02	0.05	0.13	0.65	9.E-03
eL4 cells → SST cells	Light Rep1	*Enpp2*	126	16.00	114.99	2.E-02	0.06	0.12	0.64	7.E-03
Astro cells → eL2_3 cells	Light Rep1	*Igtp*	46	25.30	138.02	2.E-02	0.11	0.33	0.92	2.E-02
Astro cells → Endo cells	Light Rep1	*Nos1*	56	10.40	117.64	2.E-02	0.04	0.27	0.73	2.E-02
SST cells → eL5 cells	Light Rep1	*Enpp2*	24	46.54	138.26	3.E-02	0.26	0.50	0.69	4.E-02
SST cells → Oligo cells	Light Rep1	*Plcxd2*	26	29.00	109.10	3.E-02	0.22	0.47	0.87	4.E-02
SST cells → Oligo cells	Light Rep1	*Slc5a7*	26	16.00	109.10	3.E-02	0.13	0.47	0.86	4.E-02
SST cells → NPY cells	Light Rep1	*Arx*	22	88.30	135.80	3.E-02	0.46	0.52	0.95	4.E-02
SST cells → SST cells	Light Rep1	*Tnfaip6*	24	17.70	91.38	9.E-03	0.15	0.60	0.97	5.E-02

Table S18. Genes showing linear dependency of expression on distance between cell types in mouse primary visual cortex in the light condition using the STARmap dataset. Same as Table S6, but for the STARmap dataset. All associations shown passed the specified 0.3 Gaussian-smoothed R^2 threshold and global FDR correction (q < 0.05).

Cell type pair	Tissue	Gene	# Interacting cells	98th percentile expression	98th percentile distance (µm)	q value	Slope	R^2 (original)	R^2 (Gaussian)	R^2 (permuted mean)
eL4 cells → eL4 cells	Dark Rep1	*Myl4*	178	19.38	53.76	7.E-03	0.13	0.11	0.46	6.E-03
eL4 cells → eL4 cells	Dark Rep1	*Pcp4*	178	23.84	53.76	3.E-03	0.18	0.13	0.39	6.E-03
eL4 cells → eL5 cells	Dark Rep1	*Ddit4l*	177	25.00	128.47	3.E-03	0.08	0.13	0.81	5.E-03
eL4 cells → eL5 cells	Dark Rep1	*Plcxd2*	177	40.48	128.47	2.E-02	-0.10	0.10	0.54	4.E-03
eL4 cells → eL5 cells	Dark Rep1	*Rasgrf2*	177	16.00	128.47	2.E-02	0.05	0.10	0.83	5.E-03
eL4 cells → SST cells	Dark Rep1	*Pcp4*	151	26.00	140.53	4.E-03	-0.07	0.14	0.80	6.E-03
eL5 cells → eL2_3 cells	Dark Rep1	*Pcp4*	70	119.10	141.51	2.E-02	0.50	0.23	0.92	1.E-02
PV cells → eL5 cells	Dark Rep1	*Pthlh*	33	22.72	112.94	3.E-02	-0.12	0.42	0.96	3.E-02
PV cells → eL6 cells	Dark Rep1	*Mdga1*	33	13.00	127.58	2.E-02	0.07	0.44	0.95	3.E-02
eL6 cells → eL6 cells	Dark Rep1	*Cux2*	108	13.44	76.24	1.E-02	0.06	0.17	0.79	6.E-03
eL6 cells → eL6 cells	Dark Rep1	*Pthlh*	108	26.58	76.24	2.E-03	0.15	0.22	0.72	9.E-03

Table S19. Genes showing linear dependency of expression on distance between cell types in mouse primary visual cortex in the dark condition using the STARmap dataset. Same as Table S6, but for the STARmap dataset. All associations shown passed the specified 0.3 Gaussian-smoothed R^2 threshold and global FDR correction (q < 0.05).

References

1. Danino-Levi,M., Goldberg,T., Keter,M., Akselrod,N., Shprach-Buaron,N., Safra,M., Singer,G. and Alon,S. (2024) Computational analysis of super-resolved in situ sequencing data reveals genes modified by immune-tumor contact events. *RNA*, 30, 749–759.

2. Fang,X., Zheng,P., Tang,J. and Liu,Y. (2010) CD24: from A to Z. *Cell Mol Immunol*, 7, 100–103.

3. Huang,S., Zhang,X., Wei,Y. and Xiao,Y. (2024) Checkpoint CD24 function on tumor and immunotherapy. *Front Immunol*, 15, 1367959.

4. D’Avola,A., Legrave,N., Tajan,M., Chakravarty,P., Shearer,R.L., King,H.W., Kluckova,K., Cheung,E.C., Clear,A.J., Gunawan,A.S., *et al.* (2022) PHGDH is required for germinal center formation and is a therapeutic target in MYC-driven lymphoma. *J Clin Invest*, 132.

5. Ren,L., Yi,J., Yang,Y., Li,W., Zheng,X., Liu,J., Li,S., Yang,H., Zhang,Y., Ge,B., *et al.* (2022) Systematic pan-cancer analysis identifies APOC1 as an immunological biomarker which regulates macrophage polarization and promotes tumor metastasis. *Pharmacol Res*, 183, 106376.

6. Zhang,Y., Zhong,F. and Liu,L. (2024) Single-cell transcriptional atlas of tumor-associated macrophages in breast cancer. *Breast Cancer Res*, 26, 129.
